# Combined computational and classical medicinal chemistry procedure to disclose novel pyrrole-based compounds as potential antituberculosis agents

**DOI:** 10.1007/s10822-026-00794-6

**Published:** 2026-04-06

**Authors:** Rino Ragno, Clemens Zwergel, Sergio Valente, Roberta Astolfi, Chiara Lambona, Eleonora Proia, Lidia Giuliani, Scott G. Franzblau, Rossella Fioravanti, Antonello Mai

**Affiliations:** 1 Rome Center for Molecular Design, Department of Drug Chemistry and Technologies, Sapienza University of Rome, Piazzale Aldo Moro 5, 00185 Rome, Italy; 2 Department of Drug Chemistry and Technologies, Sapienza University of Rome, Piazzale Aldo Moro 5, Rome, 00185 Italy; 3 Institute for Tuberculosis Research, Department of Pharmaceutical Sciences, University of Illinois Chicago, 833 South Wood Street, Chicago, IL 60612 USA

**Keywords:** 3-D QSAR, Molecular Docking, Mycobacterium tuberculosis H37Rv resistant strain, Potential MmpL3 inhibitors, Synthesis of new antitubercular compounds

## Abstract

Tuberculosis (TB) remains a major global health challenge, worsened by the rise of multidrug-resistant (MDR) and extensively drug-resistant (XDR) strains. In this study, we employed a combined computational and medicinal chemistry approach to design, synthesize, and evaluate new pyrrole-based analogues of Sudoterb (**1**, LL-3858) as potential anti-TB agents. Ligand-based quantitative structure–activity relationships (QSAR) and 3-D QSAR models, as well as structure-based docking and COMBINE analyses, were developed and used to analyze the anti-TB structural determinants and investigate the putative targeting to the MmpL3 transporter. Nineteen new analogues, belonging to amide (**2a–j**) and carbamate (**3a–i**) series, were synthesized and tested against *Mycobacterium tuberculosis* H37Rv resistant strain using the microplate Alamar Blue assay. Most of the synthesized analogues showed enhanced potency compared to Sudoterb (MIC = 20.7 µM), with **2i** (MIC = 2.8 µM) and **3 h** (MIC = 2.4 µM) emerging as the most potent and selective derivatives (IC_50_ > 80 µM in Vero cells). Computational predictions aligned well with experimental results, validating the modeling workflow. These findings identify **2i** and **3 h** as promising lead compounds and highlight the utility of integrating computational modeling with rational synthesis to accelerate anti-TB drug discovery.

**Supplementary Information:**

The online version contains supplementary material available at 10.1007/s10822-026-00794-6.

## Introduction

Tuberculosis (TB), caused by *Mycobacterium tuberculosis* (Mtb), is an infectious disease that results in the degeneration and necrosis of various organs, primarily the lungs. Until the mid-1900s, TB was considered fatal if not diagnosed promptly. However, with the advent of chemotherapy, it seemed to have been eradicated, at least in industrialized countries [1].

Due to intense migration flows and an increase in immunodepressive diseases in recent decades, TB must be considered “re-emerging”, even in industrialized countries [1, 2]. Nowadays, TB is once again one of the infectious diseases with a growing mortality rate. In 2023, 1.25 million TB-related deaths were reported. TB has become the world’s leading cause of death from a single infectious agent, surpassing the SARS-CoV-2 virus, which replaced it as the leading cause of death up to 3 years ago. According to the WHO, the infection of HIV-positive individuals is associated with increased lethality, and the spread of drug-resistant strains makes TB one of the most urgent international public health problems, despite some recent progress [1].

**Fig. 1:**
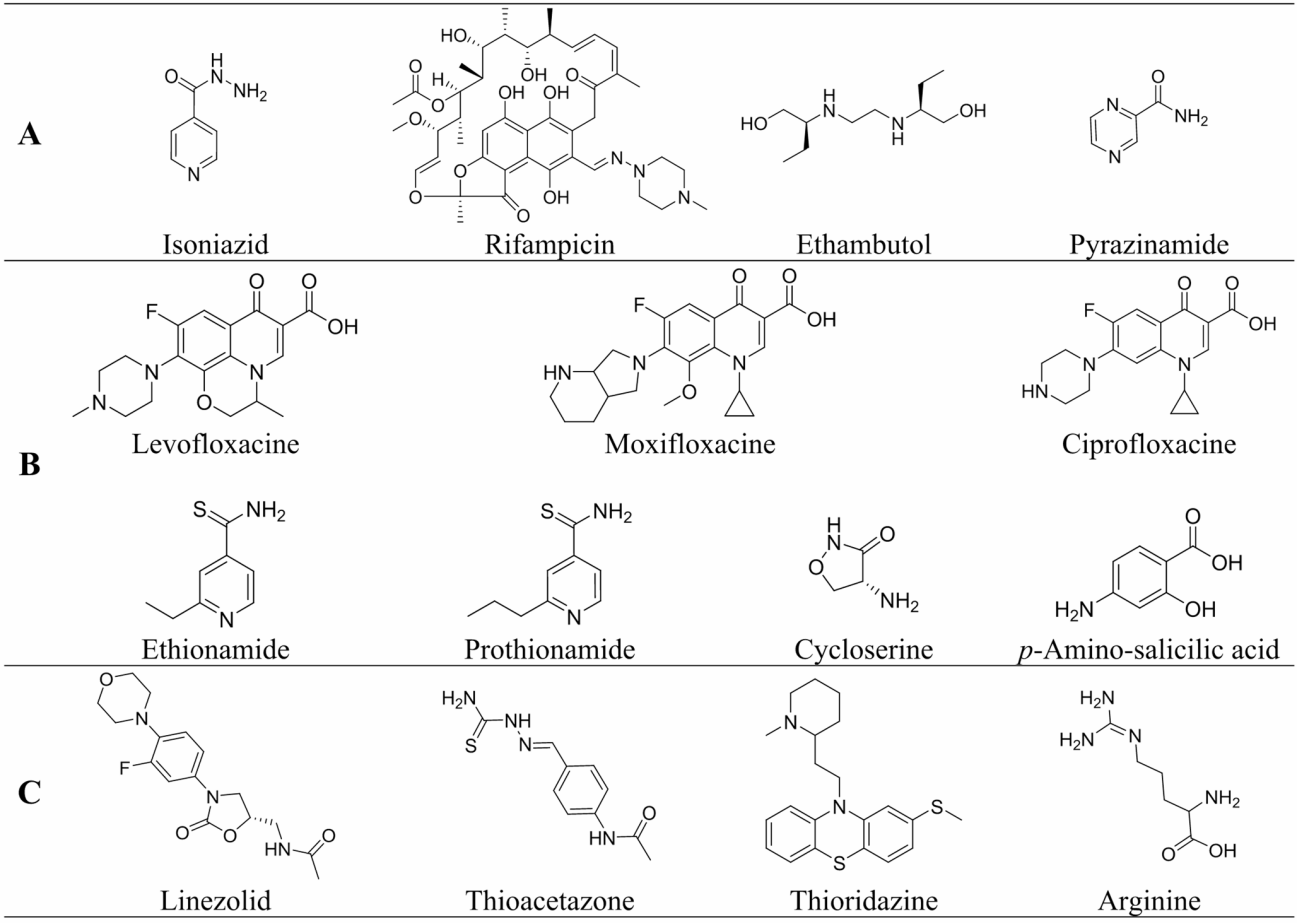
First- (**A**), second- (**B**), and third-line (**C**) approved anti-TB drugs, as classified by WHO

The TB treatment guidelines are intended for settings with low incidence rates, where mycobacterial cultures, molecular and phenotypic drug susceptibility tests, radiographic studies, and other diagnostic tools are routinely available. The most effective strategy for ensuring adherence to treatment is directly observed therapy (DOT). With DOT, a healthcare worker or other designated person watches the TB patient swallow each dose of the prescribed drugs. DOT and integrated case management remain the standard of care [3]. Drug-susceptible TB can be effectively treated within 6 months using a standard treatment regimen that includes a combination of primary anti-TB drugs such as isoniazid, rifampicin, rifabutin, ethambutol, pyrazinamide, and streptomycin (Fig. 1). Conversely, the treatment effectiveness of multidrug-resistant (MDR) and extensively drug-resistant (XDR) strains still remains poor. These strains require treatment with second- and third-line antitubercular drugs for up to two or more years (Fig. 1). There are no evidence-based recommendations for treating XDR-TB. Any treatment resulted in a cure rate as low as 26% [4].

**Fig. 2:**
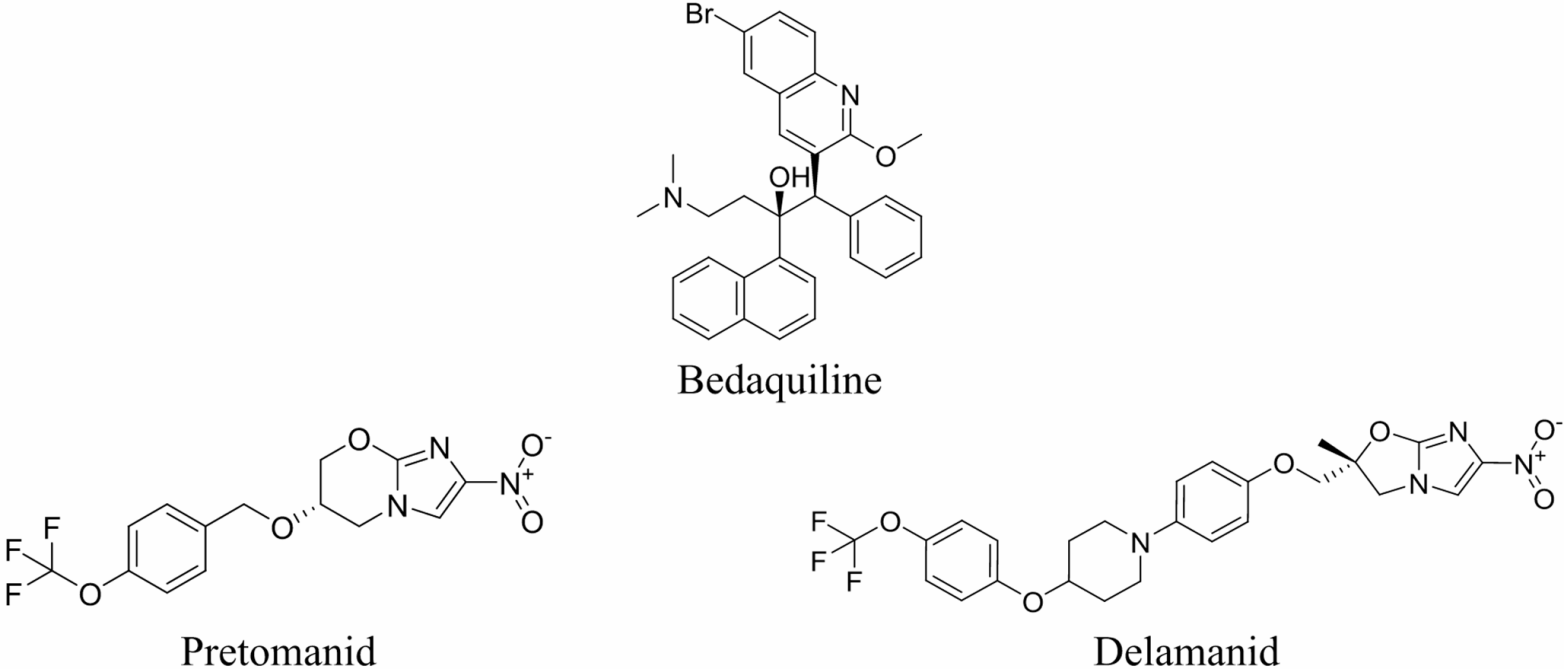
The structures of Bedaquiline, Pretomanid and Delamanid, recently FDA-approved anti-TB drugs

Depending on the patient’s clinical status, the adopted therapy is somewhat complex and long-lasting. This results in treatment cycles that are not respected or are even interrupted, which in turn fosters the development of more resistant forms. Despite DOT’s recommendations, the rapid spread of antibiotic-resistant tuberculosis strains is a major problem, as is the duration of drug treatment necessary to eradicate the disease and the relatively slow development of new antitubercular agents. For isoniazid- and rifampicin-susceptible TB in adults, a 4/6-month rifapentine-moxifloxacin regimen could be effective. However, for rifampicin- and fluoroquinolone-resistant TB, a 6- to 15-month regimen of bedaquiline, pretomanid, and linezolid is necessary for adolescents aged 14 and older and adults with rifampicin-resistant pulmonary TB [3]. Standard antiretroviral therapy for HIV/AIDS patients is incompatible with current tuberculosis treatment due to drug toxicity and interactions. According to the latest WHO report, multidrug-resistant tuberculosis (MDR-TB) continues to rise [2, 5]. Opportunely, TB drug discovery, neglected for decades, is increasing, and new chemical entities are being evaluated in preclinical and clinical settings [6]. As a result, in the past decade the FDA has approved three new anti-TB drugs: Bedaquiline [7, 8], Delamanid [9, 10], and Pretomanid [11, 12] (Fig. 2).

Bedaquiline, introduced as a second-line treatment, is the first anti-TB drug introduced in a gap of more than 40 years [7]. Bedaquiline inhibits mycobacterial ATP (adenosine 5’-triphosphate) synthase by binding to the enzyme c subunit, which is essential for the generation of energy in Mtb [13, 14]. Delamanid and Pretomanid are both prodrugs that are activated by a biotransformation via the mycobacterial F420 coenzyme system. This produces various active metabolites that act as a bacterial respiratory poison under anaerobic conditions [11, 12]. The final effect is the overall inhibition of mycobacterial cell wall mycolic acid biosynthesis, which blocks Mtb replication.

The discovery of novel anti-TB drugs is particularly demanding and complex because TB needs a chronic therapy and new agents should possess good bactericidal activity via novel mechanisms without exhibiting, at the same time, elevated host toxicity and lack of any drug-drug interaction. Although the Mtb genome has been completely sequenced, only a few of its approximately 625 essential genes have been studied in detail. Nevertheless, some of these genes are promising potential anti-TB targets that have yet to be validated [10]. Additional updates on anti-TB drug discovery, including novel hits and repurposed drugs, can be found in comprehensive reviewes [2, 5, 15, 16].

Of the many chemical scaffolds reported as potential novel anti-TB agents [17], pyrrole derivatives have demonstrated activity against Mtb in vitro [18]. Most of these derivatives have been shown to target the mycobacterial membrane protein large 3 (MmpL3), which is an essential transporter in Mtb responsible for exporting trehalose monomycolate. Trehalose monomycolate is a crucial precursor in the biosynthesis of mycolic acids, which are integral to the mycobacterial cell wall [19]. Sudoterb (LL-3858, **1**), a pyrrole-based compound, was developed by Lupin Ltd. (Pune, India) [20, 21], and could be retained as a constrained pyridin-4-yl hydrazone. Compound **1** exhibited potent in vitro anti-TB activity with an MIC range of 0.06–0.5 µg/mL against Mtb, including MDR strains, with a MIC90 of 0.25 µg/mL [22, 23], Compound **1** also shows in vitro synergy with rifampicin. In vivo, a dose of 12.5 mg/kg of **1** reduced the mycobacterial load in a TB mouse model to a greater extent than isoniazid did, exhibiting bactericidal activity at doses well below the toxic threshold. In combination with current anti-TB drugs, **1** was reported to sterilize the lungs and spleens in less time than conventional therapy [24]. It is currently in clinical development for the treatment of TB; however, no data are available about its pharmacokinetics in humans, and its mechanism of action and molecular target have not yet been established [25].

**Fig. 3:**
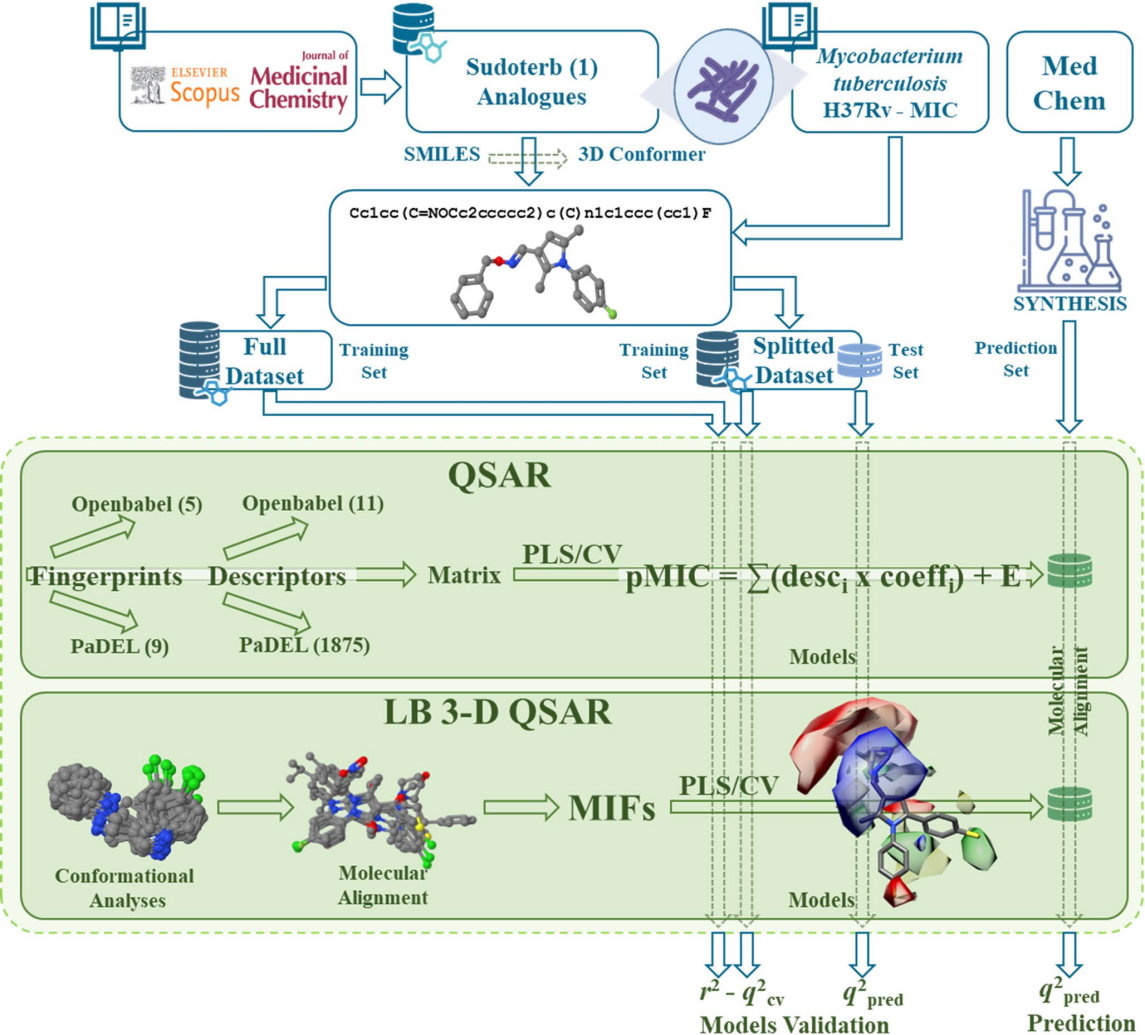
Scheme of the LB procedure (see main text)

**Fig. 4:**
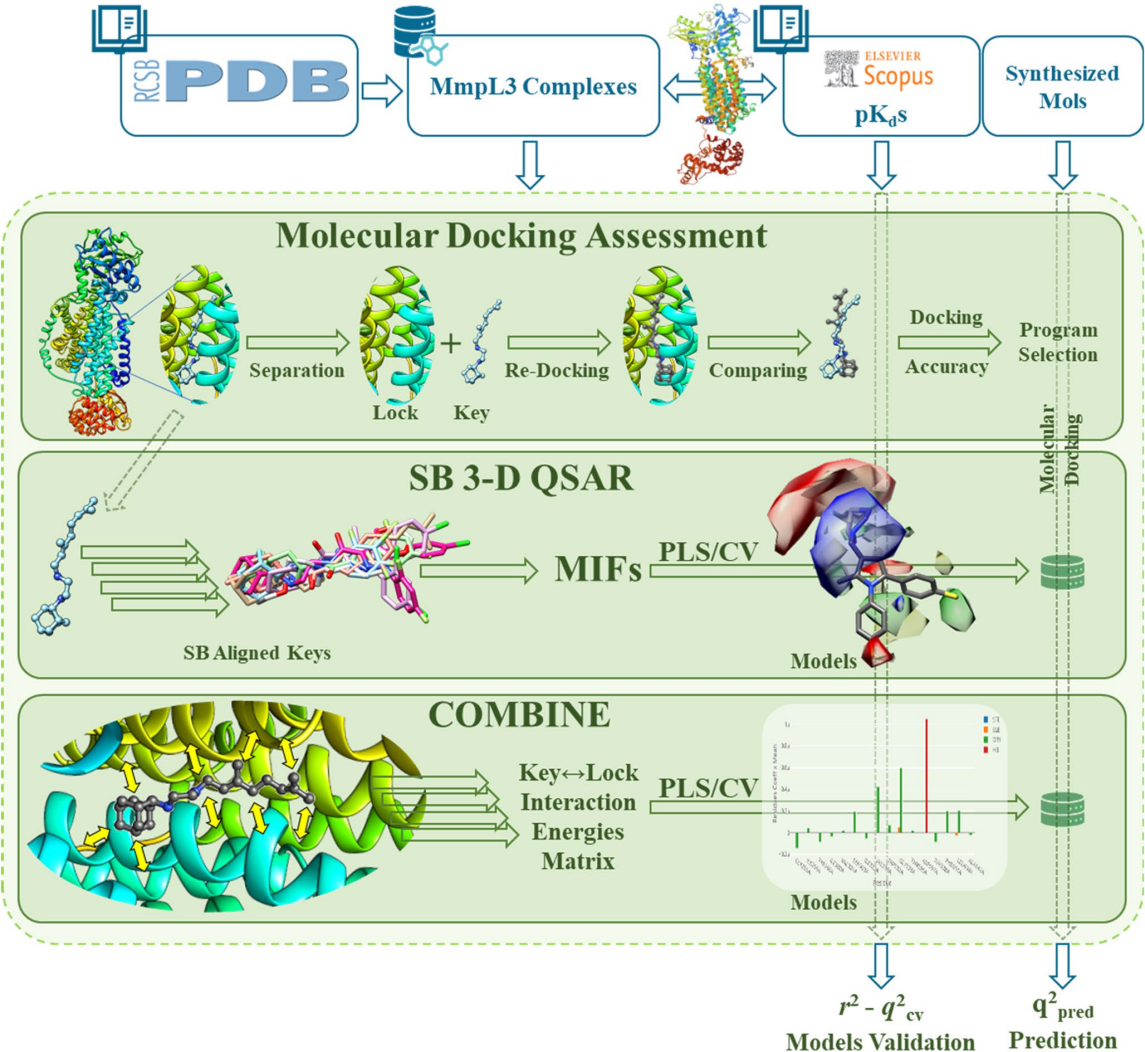
Scheme of the SB procedure (see main text)

**Fig. 5:**
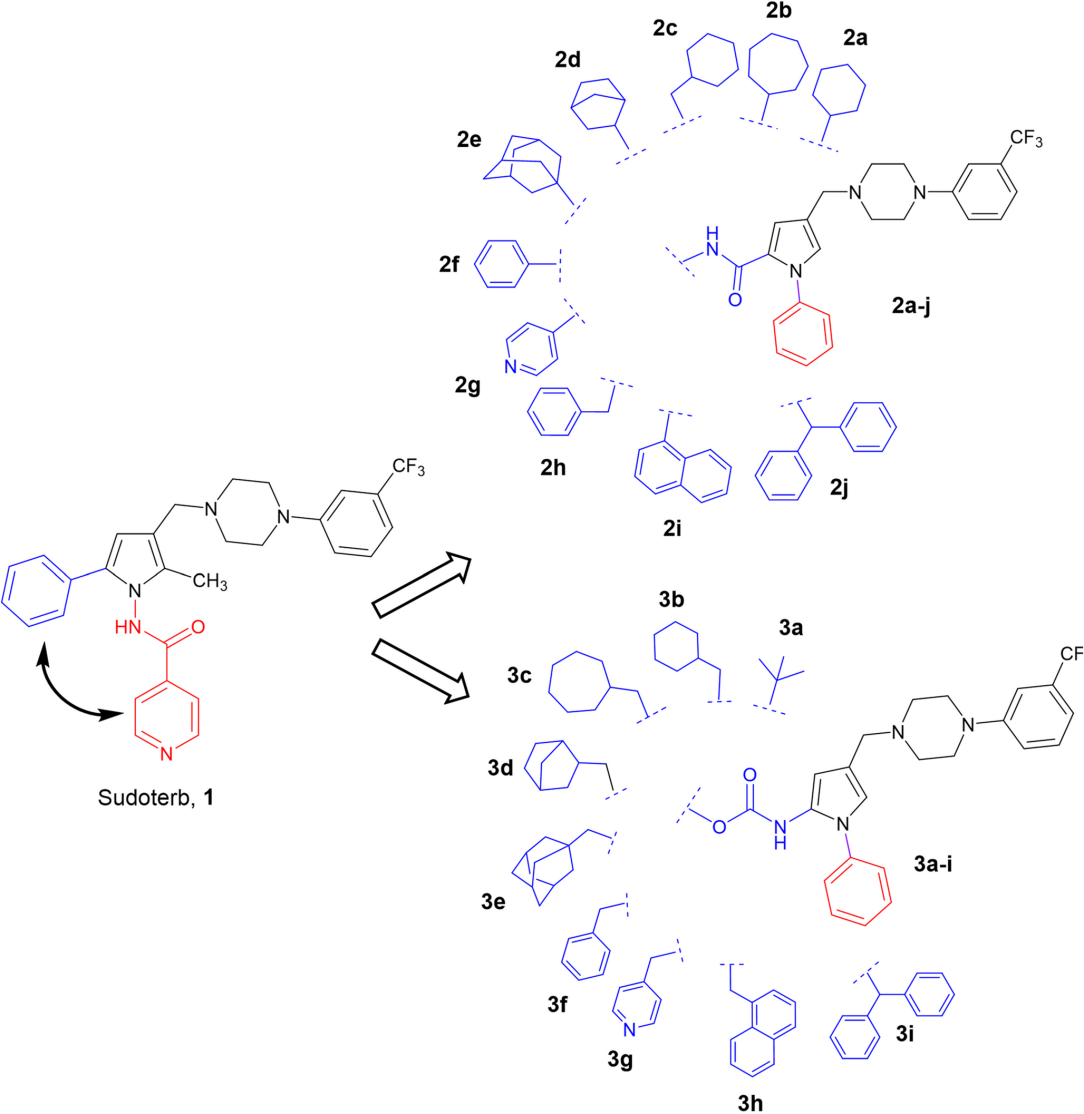
Design of **1** analogues belonging to the amide (**2a-j**) and carbamate (**3a-i**) series

Herein is reported a combination of ligand-based (LB, Fig. 3) and structure-based (SB, Fig. 4) computational methods, joined to classical medicinal chemistry approach (Fig. 5) aimed to disclose a series of new anti-TB agents (**2a-j** and **3a-j**) as chemical chimeras between **1** and other reported pyrrole derivatives.

## Drug design procedure

### Computational medicinal chemistry

LB and SB molecular modeling procedures were set up to develop predictive chemical descriptor Hansch-based [26] (Chem) and structural fingerprints Free-Wilson-based [27] (Struct) quantitative structure-activity relationships (QSARs), molecular interaction fields (MIFs)-based three-dimensional QSARs [28] (MIF 3-D QSAR or CoMFA), and comparative molecular binding energy [29] (COMBINE) models. Sudoterb and compounds **2a-j** and **3a-j**, that were being synthesized in the meantime, were subsequently used as a validation set (VS) for the quantitative models.

The LB and SB procedures were developed through the following points:

#### Dataset composition

A list of molecules structurally related to **1** were retrieved from literature and used to develop either robust or predictive models. To this, the dataset was treated in two different ways:

A. The dataset was directly used as training set (Full).
B. The dataset was splitted (Splitted) into training and test sets.

#### Generation of 3D conformations for each dataset molecule

For each dataset’s molecule the SMILES [30] structure was obtained through Py-MolEdit section of the 3d-qsar.com website [31]. The dataset’s molecules were modeled in local minima conformations directly from SMILES strings by means of a python script which used the openbabel [32, 33] to obtain acceptable 3D conformers [34].

#### LB approaches

In this section QSAR and 3-D QSAR models were built according to the scheme depicted in Fig. 3.

***Structural descriptors:*** different types of fingerprints were calculated by means of the openbabel and PaDEL softwares [32, 35].

***Chemical descriptors:*** two different sets of molecular descriptors were calculated, either simple (through openbabel) or large (by means of PaDEL) one.

***Applicability domain:***

- *AD definition:* the training set AD was calculated with either structural descriptors or chemical descriptors
- *AD application*: defined ADs were used to check the chemical space of the training set sufficiently covered either the scaffold or the physical-chemical character of **1** and the newly synthesized compounds **2a–j** and **3a–i**.

***QSAR modeling:***

- *QSAR modeling*: models for the Full and Splitted datasets were built through a python script using the partial least squares (PLS) regression method [36] and the cross-validation (CV) technique. A series of QSAR models were built using either structural (Struct) or chemical (Chem) descriptors and characterized by classical (*r*^2^), cross-validated (*q*^2^_cv_), and predictive (*q*^2^_pred_) determination coefficients.
- *QSAR model application*: the selected most robust (Full) and predictive (Splitted) QSAR models were applied to predict the validation set compounds.

***3-D QSAR modeling:*** 3-D QSAR models were built using the python implemented CoMFA code (Py-CoMFA) procedure, as previously reported [37]. The dataset molecules were sought for optimal alignment rules through a combination of conformational analyses and automatic molecular alignment methods. The aligned dataset was then subjected to molecular interaction fields (MIFs) calculations and to PLS regression and CV elaborations to obtain *r*^2^, *q*^2^_cv,_ and *q*^2^_pred_ coefficients.

- *Conformational analyses*: using different programs, a series of conformational analyses were run to inspect the molecule’s conformational space.
- *Molecular alignments*: each training set conformational analyses was used directly for the molecular alignments using different automatic methods, various molecule templates, and selecting different conformations for each template.
- *Calculation of MIFs*: the TRIPOS force field, as re-implemented in the Py-CoMFA, was used to calculate the MIF around the aligned molecules using the same settings as described in the original CoMFA method [28].
- *Py-CoMFA best models*: final 3-D QSAR models were selected by means of highest *r*^2^, *q*^2^_cv,_ and *q*^2^_pred_ values and inspected through the average activity contribution (AAC) and activity contribution (AC) plots to delineate a 3-D SAR model.
- *3-D QSAR Model application*: the selected Full and Splitted best Py-CoMFA models were applied to predict the activities of the compounds being synthesized.

#### SB approaches

A structure-based protocol (Fig. 4) was applied to investigate the likely binding mode of **1** and the herein designed derivatives **2a–j** and **3a–i** into MmpL3 as a potential biological target [38–40].

- *SB learning dataset*: All available MmpL3 crystal structures available from PDB [41] were retrieved, and those complexed with a small organic compound for which an affinity value was available were retained.
- *Molecular docking assessment*: the retrieved complexes were used to set-up re-docking and cross-docking experiments to select the most suitable combination of program/scoring function to investigate the binding mode of new potential MmpL3 ligands.
- *SB 3-D QSAR and COMBINE modeling*: the retrieved complexes were used to build SB 3-D QSAR and COMBINE models. These models coupled with molecular docking could be used as external scoring functions to evaluate the putative inhibitory potency of compounds with unknown experimental binding mode [42, 43].
- *SB 3-D QSAR*: the experimental complexes were aligned by means of the matchmaker routine implemented in the UCSF Chimera program [44]. The extracted SB aligned ligands were then used to build and evaluate (*q*^2^_cv_) Py-CoMFA models through the same 3-D QSAR method applied for the LB models.
- *COMBINE*: through the Py-ComBinE code implemented in the 3d-qsar.com, COMBINE models were built and evaluated through the *r*^2^ and *q*^2^_cv_ coefficients.

#### Application of LB and SB approaches to the validation set

***MIC predictions***: The LB models (QSAR and 3-D QSAR) were used to predict the MIC values of the designed compounds through a consensus type application.

***Binding mode***: Putative binding mode and protein affinity prediction of the newly synthesized compounds were investigated through molecular docking and COMBINE models, respectively.

## Synthetic drug design approach

A classical approach was applied to delineate structure-activity relationships (SARs) based on the Sudoterb structure 1 (Fig. 5).

A library of analogs was designed by moving the **1** phenyl ring from pyrrole C5 to N1 position, and replacing the *iso*-nicotinamide group at N1 of the prototype with various amide or carbamate groups inserted at the C5 position of the pyrrole (compounds **2a-j** and **3a-i**, Fig. 5). The group switching C5←→N1 in Sudoterb (**1**) was suggested to expand actual anti-TB SARs. Despite Sudoterb, derivatives such as pyrrole [45] or pyrazole- based [46] with a substituted phenyl moiety at N1 were all bearing in C5 position a substituted phenyl group. Other reported MmpL3 inhibitors such as NITD-304 [47], CRS400393 [48] and THPP [49] contained benzyl, phenyl and cycloaliphatic substituted amides inserted in the proximity of an aromatic nitrogen (pyrrole, pyrazole, indole, benzothiazole, etc.), this observation supported the introduction of a substituted amide group at position C5.

A survey on ChEMBL [50] and PubChem [51] databases indicated that such a list of designed compounds has never been biologically investigated or even synthesized.

## Results

### Computational medicinal chemistry

#### LB dataset composition

**Fig. 6:**
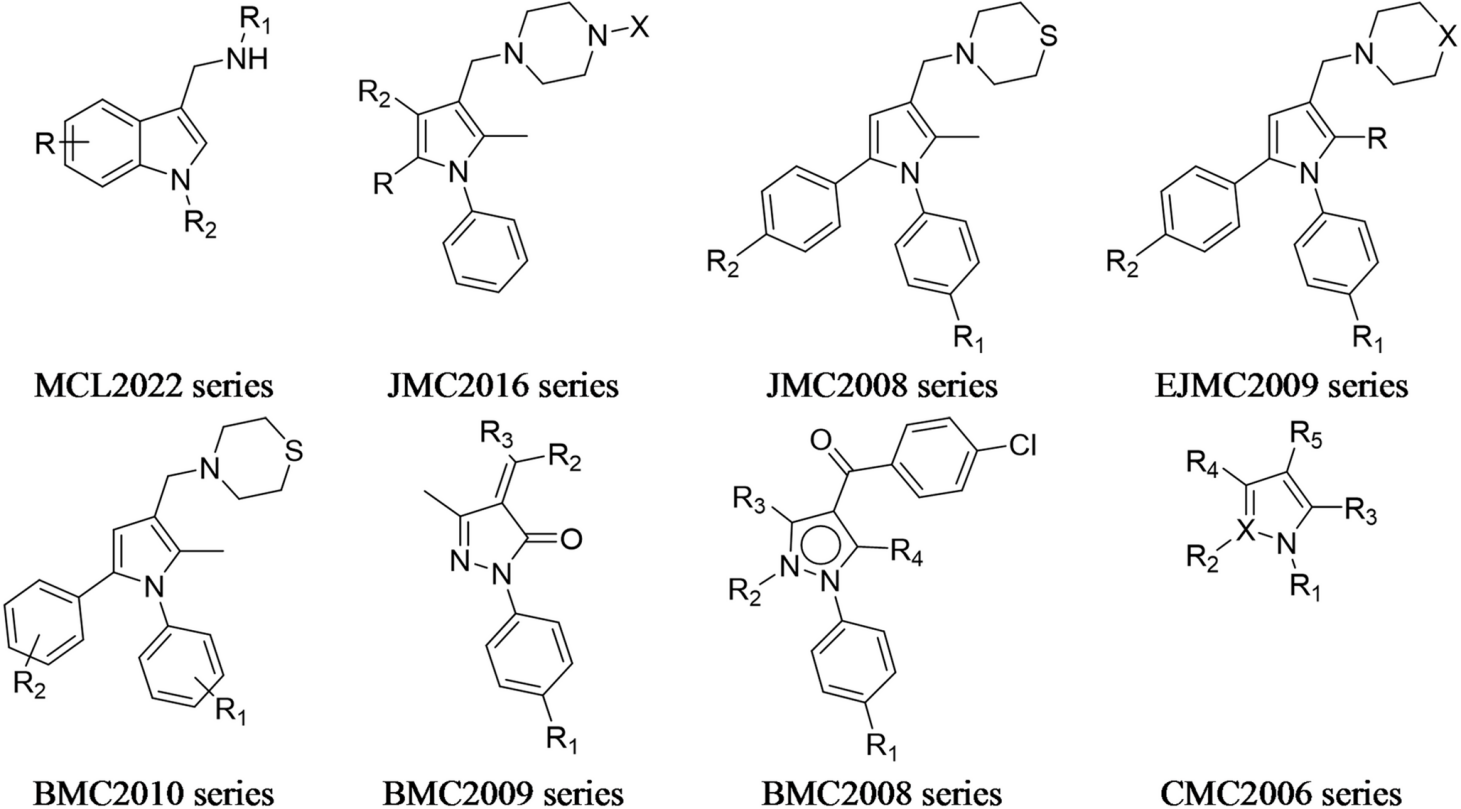
List of molecular scaffolds used to compile the LB dataset (for the references see Supplementary material Tables S1)

A list of 126 molecules reported to be active against H37Rv and belonging to different chemical scaffolds was selected from the literature [45, 52] to compile the final dataset (Fig. 6 and Supplementary Material Tables S1–S9), which comprised molecules similar to **1** or its simplified or complicated analogues. To develop either robust or predictive models, the dataset was used in two different ways:

A. The Full dataset of 126 molecules with MIC values spanning more than 3 log units, with pMIC values ranging from 3.29 to 6.82 (Supplementary Material Tables S1–S9). The Full dataset was subjected to the Py-CoMFA method, leading to the development of a number of 3-D QSAR models.
B. The Splitted dataset was obtained by dividing the Full dataset into training and test sets with 88 (70%) and 38 (30%) compounds, respectively. Training set and test set splitting was performed keeping the pMIC ranges as close as to that of the Full dataset.

#### Generation of 3D conformations for each dataset molecule

Through the Py-MolEdit module, each of the 126 molecule SMILES structures was manually drawn and converted to 126 local minima conformations, which were stored in the associated 3d-qsar.com database and used for the subsequent elaborations.

#### LB approaches

***Applicability domain***: AD for the Chem and Struct dataset were calculated and their application indicated that **1** and the new molecules **2a–j** and **3a–i** were either fully covered or having low probability to be considered out of the AD. The descriptor-based AD (Chem-AD) was defined with a list of descriptors (molecular weight, the logarithm of the calculated octanol/water partition coefficient, the number of hydrogen bond donators, the number of hydrogen bond acceptors, the total polar surface area, the number of rotatable bonds, the number of aromatic rings, the number of heavy atoms, the fraction of sp3 carbon atoms and the total number of rings) calculated with RDKit. The Chem-AD was characterized by an average distance of 2.962, a threshold (90th percentile) of 4.533 and a training distance standard deviation of 1.071. All designed compounds exceeded the strict applicability domain threshold based on distance criteria, several positive observations emerged from the analysis (Supplementary Material Table S10). The relatively low probability values for compounds being outside the Chem-AD (ranging from 0.000 to 0.047) suggested that many molecules were positioned near the domain boundary rather than being considered full outliers. Several compounds showed modest percentage deviations beyond the threshold (7.38% to 29.30%), indicating proximity to the training set’s chemical space.

The fingerprints-based AD (Struct-AD) was defined using Morgan type fingerprints with radius 2 and 2048 bits and was characterized by average distance from center of 12.780 and a threshold (90th percentile) of 18.601. Application of Struct-AD to the titled compounds (Supplementary Material Table S11) indicated that all test set compounds fell within the established applicability domain boundaries.

The structural consistency among the test compounds, featuring related scaffolds with similar functional groups, demonstrated systematic exploration of a focused chemical region. Despite the formal designation as outside the Chem-AD, the compounds exhibited controlled structural variations around the core molecular framework, suggesting the model’s predictions would likely remain reasonably reliable for this chemically coherent series.

***QSAR modeling***: Models for the Full and Splitted datasets were built through a python script using the partial least square (PLS) method [36] as implemented in the Scikit-learn module [53]. Full QSAR models were evaluated by *q*^2^_cv_ values, while the Splitted based models were ranked by the *q*^2^_pred_. Two different sets of structural or chemical descriptors QSAR models were obtained:

- *Models with structural descriptor*: Different fingerprints were calculated. Five of them (FP2, FP3, FP4, MACCS, and Spectrophore) were obtained with openbabel [32] through a python script, and further nine (Fingerprinter, Extended Fingerprinter, Estate Fingerprinter, MACCS Fingerprinter, Pubchem Fingerprinter, Substructure Fingerprinter, KlekotaRoth Fingerprinter, GraphOnly Fingerprinter, and AtomPairs2D Fingerprinter) were calculated through the PaDEL software [35]. These led to obtain 14 Structural QSAR models. Among those elaborated with the Full dataset, the *q*^2^_cv_ top model, obtained with the PaDEL Fingerprinter (M_QSAR_Full_Struct__9, Table 1 and Supplementary Material Tables S12–S13 and Fig. S1), showed a *r*^2^ value of 0.95 and an *q*^2^_cv_ of 0.83 with 7 optimal principal components (PCs). Differently, the best model associated with the Splitted dataset (model M_QSAR_Splitted_Struct__6, Table 1 and Supplementary Material Table S14) revealed a *q*^2^_pred_ value of 0.69 with 4 optimal PCs and corresponding *r*^2^ and *q*^2^_cv_ values of 0.88 and 0.78, respectively (Supplementary Material Table S15 and Fig. S2).
- *Models with chemical descriptors*: Two different sets of molecular descriptors were calculated. One set was calculated using OpenBabel and contained 11 descriptors. The other set was calculated using PaDEL and contained 1,875 descriptors. The openbabel descriptor based QSAR models (Full or Splitted) did not produce any interesting model with *q*^2^ and *r*^2^ (Supplementary Material Table S12). The PaDEL-based descriptors model for the Full dataset was initially not fully satisfactory (Supplementary Material Table S14), and due to the number of descriptors, it was subjected to refinement through a variable selection by means of a python implementation of a simulated annealing algorithm (SA). The SA reduced the number of descriptors, eliminating in an iterative fashion those less important to the regression, consequently the *q*^2^_cv_ rose to 0.80 with an associated *r*^2^ value of 0.89 at 5 PCs (model M_QSAR_Full_Chem__SA, Table 1, Supplementary Material Table S16 and Fig. S3). Differently, the models developed with the Splitted dataset showed interesting *r*^2^ and *q*^2^_pred_ values, but not at the same level of the model based on the fingerprints (Table 1, Supplementary Material Table S14). As for the Full dataset model, the Splitted dataset QSAR model was subjected to refinement through SA. As a result, the number of chemical parameters decreased, leading to *q*^2^_pred_, *q*^2^_cv,_ and *r*^2^ values of 0.67, 0.78, and 0.90, respectively (model M_QSAR_Splitted_Chem__SA, Table 1, Supplementary Material Table S17 and Fig. S4).
- *QSAR-based pMIC predictions for the validation set*: The QSAR selected models M_QSAR_Full_Struct__9, M_QSAR_Splitted_Struct__6, M_QSAR_Full_Chem__SA, and M_QSAR_Splitted_Chem__SA (Table 1) were used to predict **1** and the planned compounds **2a-j** and **3a-i**. The four models predicted compounds with MIC values in the range of 5–100 µM (Supplementary Material Fig. S5). The Struct models, which rely on molecular fingerprints to capture structural similarity, show more variability between compounds compared to the Chem models. In contrast, the Chem models, based on calculated chemical descriptors, exhibited relatively tighter agreement between the Full and Splitted versions, and generally produced higher pMIC estimates. This suggests that the Chem models may be capturing more stable relationships between molecular properties and biological activity. Fingerprint-based models (Struct) produced less accurate predictions, especially in the Full models. Chemical descriptor-based models (Chem) generally produced higher and more consistent predictions, indicating that chemical descriptors capture important features related to biological activity, regardless of whether the model is built through internal or external validation. The four models captured different aspects and therefore returned some variability in pMIC predictivity of the validation set, thus suggesting that they should be applied in a sort of consensus using average predictions

The lower sensitivity of Struct models compared to Chem models may be related to the different adherence of the new compounds to the aforementioned ADs. In fact, the new molecules were recognized to be fully covered by the Struct-AD, interpreting the molecules slightly too structurally related to the training set and therefore the Struct QSAR model was somehow less effective in picking structure differences and thus a lower correlation with the biological activity and slightly higher error of prediction resulted. On the other hands, regarding the Chem QSAR model, as the molecules were slightly outside the Chem-DA boundaries, this reflected the model to apply a limited extrapolation that returned values with lower error of prediction compared to the Struct QSAR model.

**Table 1:** List of QSAR best performing models (Supplementary Material Tables S12 and S14)

Model #	Descriptors	*r* ^2^	q^2^_cv_	q^2^_pred_	ONPC^b^
M_QSAR_Full_Struct__9	PaDEL Fingerprinter	0.95	0.83	NA	7
M_QSAR_Splitted_Struct__6	PaDEL AtomPairs 2D Fingerprinter	0.88	0.78	0.69	4
M_QSAR_Full_Chem__SA^a^	PaDEL Descriptors	0.89 ± 0.01%	0.80 ± 0.01%	NA	5
M_QSAR_Splitted_Chem__SA^a^	PaDEL Descriptors	0.90 ± 0.01%	0.78 ± 0.03%	0.67 ± 0.07% ^c^	4

a. Due to the stochastic nature of the SA optimization three independent optimization were run and therefore the results are reported as mean ± standard deviation from three independent SA optimizations

b. Optimal number of principal components

***3-D QSAR modeling:*** 3-D QSAR models were built using the Py-CoMFA [37]. To this the datasets were sought for optimal alignment rules (conformational analyses and molecular alignments), subjected to molecular interaction fields (MIFs) calculations, and elaborated with PLS and CV to obtain Py-CoMFA models characterized and ranked by *q*^2^_cv_ (Full aligned datasets, FAs) and *q*^2^_pred_ (Splitted aligned datasets, SAs) coefficients.

- *Conformational analyses*: on the initial dataset of 126 molecules, 16 different conformational analyses (CA1-CA16, Supplementary Material Table S18) were run to inspect the molecule’s conformational space with either different and robust search algorithms and/or force fields [34]. The CAs were performed with three different approaches: the free software Balloon [54] and with a python script through the openbabel and RDKit python libraries. By combining the available force fields and the main features of each method, the CAs were run to collect for each a maximum of 30 conformations for each molecule listed in the dataset (Supplementary Material Fig. S6).
- *Molecular alignments*: each of the CAs was used in automatic molecular alignments using 6 different methods (Shaep [55], RDKit [56], Align-it [57], Shape-it [58], InterLig [59], and fkcombu [60]) to generate an aligned dataset to feed the Py-CoMFA module for the MIFs calculations and hence to build the 3-D QSAR models. To this, for each of the 16 CAs, 17 different molecules and 3 unique conformations (Supplementary Material Tables S19 and S20) were selected as templates to generate automatically aligned datasets. Considering also the different available scoring functions for each alignment method (Supplementary Material Table S21), thousands of combinations resulted, and thus too demanding for a systematic approach; therefore, it was decided to randomly select 5% of them to generate the aligned datasets [61]. A total of 431 (Supplementary Material Table S22) and 435 (Supplementary Material Table S23) automatically aligned datasets were obtained for the Full and Splitted datasets, respectively.
- *Py-CoMFA modeling*: from the above aligned datasets, a total of 431 (Supplementary Material Table S24) and 435 (Supplementary Material Table S25) 3-D QSAR models were built for the Full and Splitted datasets, respectively. The best model were selected by means of highest q^2^_cv_ (Full dataset) and q^2^_pred_ (Splitted dataset). The top model for the Full dataset was characterized by a q^2^_cv_ of 0.82 and a r^2^ of 0.95 at 7 PCs (model M_3-D_QSAR_Full__236, Table 2 and Supplementary Material Tables S22 and S23) and was obtained with alignment FA236 using the conformational analysis performed through the RDKit/MMFF94 method and the alignment with RDKit/BestScore combination software and using as template the molecule with the highest number of hydrogen bonding acceptor points (ACS_MCL2022_7m) and with the conformation with coordinates embracing the largest volume (Supplementary Material Table S22).

The best Splitted model had a *q*^2^_pred_ value of 0.82 with an associated *r*^2^ and *q*^2^_cv_ values of 0.92 and 0.64, respectively, at 5 PCs (model M_3-D_QSAR_Splitted__170, Table 2 and Supplementary Material Tables S26 and S27). The dataset alignment rules (Supplementary Material Table S23) were obtained through alignment SA170 using the fkcombu/Volume Rigid pair applied on the conformational analysis from openbabel/Ghemical/Random combination and using as template the conformation of the biggest conformations of the molecules having the lowest number of hydrogen bonding donators groups (BMC2009_9g).

**Table 2:** List of 3-D QSAR best performing models (Supplementary Material Tables S22 and S24)

Model #	*r* ^2^	q^2^_cv_	q^2^_pred_	ONPC^a^ ext
M_3−D_QSAR_Full__236	0.95	0.82	NA	7
M_3−D_QSAR_Splitted__170	0.92	0.64	0.82	5

a. optimal number of principal components

- *Py-CoMFA contour maps analysis*: the above obtained models were graphically inspected through the average activity contribution (AAC) plots. Regarding the Full dataset-based model, the overlap of the most potent molecule (BMC2010_12) with the steric and electrostatic CoMFA-like maps reveals distinct spatial zones where molecular modifications can either enhance or diminish activity. AAC maps were generated and reflect average contributions derived from the product of the molecular interaction fields and the PLS regression coefficients. The resulting polyhedral, green and yellow for steric, blue and red for electrostatic, indicate where structural changes are likely to have a positive or negative impact on anti-TB potency.

Starting with the steric (STE) map, green regions represent areas where increasing steric bulk enhances activity, while yellow regions highlight zones where bulky substituents are detrimental for the activity. A prominent green region surrounds the nitrogen atom of the thiomorpholine group, suggesting that introducing bulky substituents in this area could enhance activity. This aligns with the molecule’s current structure, which already positions the nitrogen atom optimally. Another significant green region is observed near the aromatic rings, particularly on one side of the ring system. This indicates that extending bulky groups from this side, such as phenyl, cycloalkyl, or other hydrophobic moieties, could improve potency by engaging favorably with the surrounding space. A yellow region is consistently present near the thiomorpholine sulfur atom, indicating that adding bulky groups in this area would lead to steric hindrance and reduce activity. Therefore, it is advisable to avoid introducing large substituents near the sulfur atom. The *N*-phenyl group is also near a yellow region. This suggests that inserting bulky groups on the benzene ring could reduce activity; therefore, keeping this region minimal or avoiding bulky additions is crucial. The last part refers to the phenyl moiety bonded to position C5 of the pyrrole ring, specifically, there are polyhedron that extend away from the central ring system. These regions are marked as unfavorable in both the steric and electrostatic maps, indicating that introducing bulky or charged groups here would likely reduce activity due to steric hindrance or electrostatic repulsion.

Regarding the electrostatic (ELE) maps, blue polyhedron indicate favorable regions where appropriately charged groups enhance activity, while red polyhedron represent unfavorable regions where charges reduce activity. A strong blue region surrounds the thiomorpholine nitrogen atom, consistent with its role as a positively ionizable center. This supports the idea that preserving or enhancing the basicity of this site contributes positively to activity. A blue region extends along one edge of the pyrrole ring, suggesting that electron-rich substituents, placed in this area, may further improve potency. A red region is observed near the sulfur atom, indicating that introducing negatively charged groups in this area could destabilize interactions and reduce activity. Similar to the steric map, the phenyl attached to the N1 pyrrole nitrogen is near a red region. Placing positively charged groups in this area could also reduce activity due to unfavorable electrostatic interactions. To avoid any redundancy, the AAC map analysis for the Splitted dataset-based model M_3-D_QSAR_Splitted__170 is reported in Supplementary Material.

**Fig. 7:**
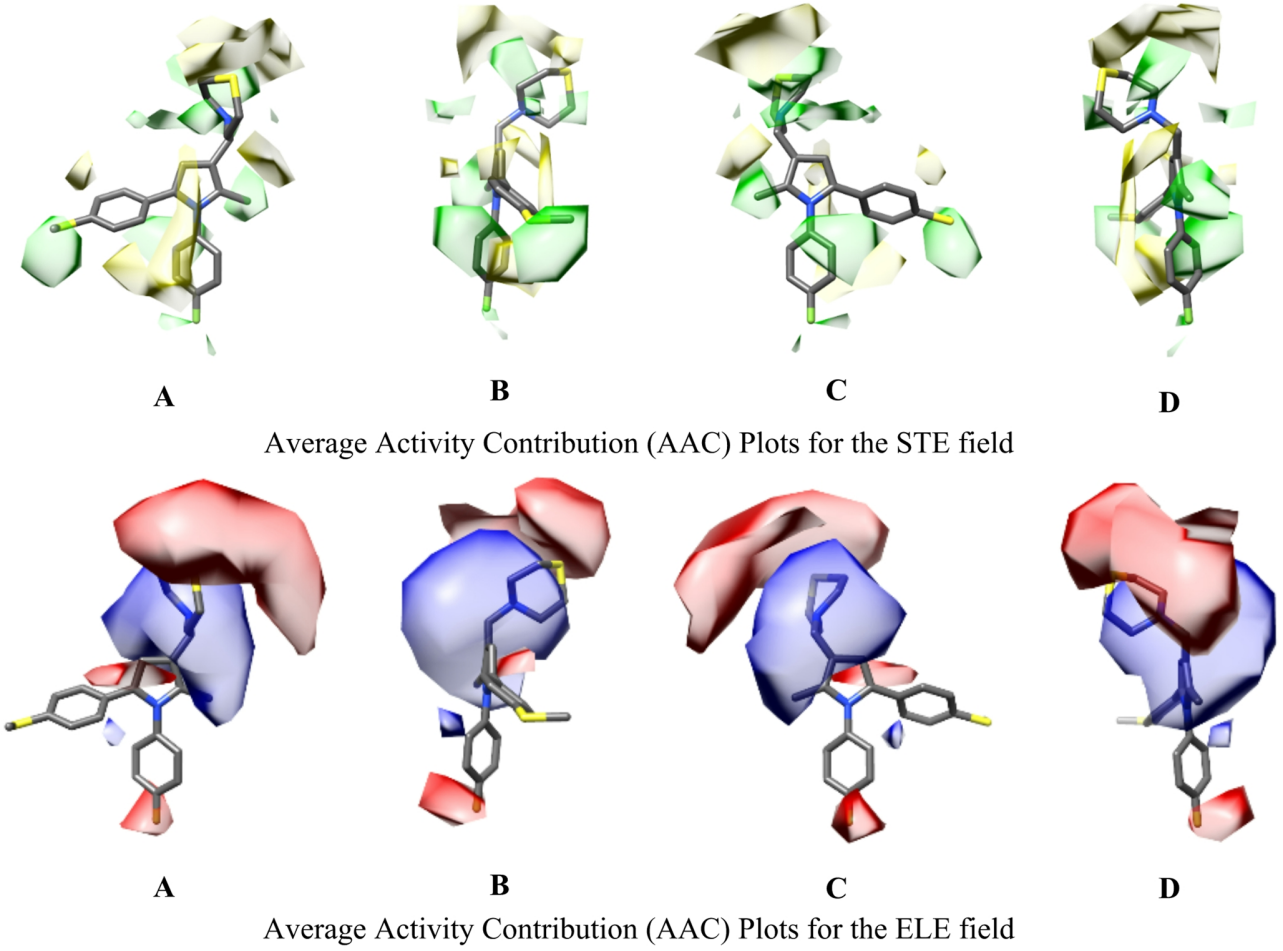
AAC plots relative to the M_3-D_QSAR_Full__236 model. Four images (**A**–**D**) are given for the STE and ELE fields. From left (**A**) to right (**D**), each image has a 90° rotated view of the same object. BMC2010_12, the most potent compound of the dataset, is depicted along with the AAC. Images were generated with the 1.18 version of UCSF Chimera software [62]

- *3-D QSAR prediction of the validation set*: Sudoterb (**1**) and the designed new compounds **2a-j** and **3a-i** were subjected to the same alignment rules as described above, and their pMIC was predicted through the M_3-D_QSAR_Full__236 and M_3-D_QSAR_Splitted__170 models. The application of the two selected Py-CoMFA models to the under synthesizing compounds predicted their MIC values to range between 2 and 50 µM (Fig. S10) (Fig. 7).

#### SB approaches

Recently, the membrane transporter MmpL3 has been proposed as a biological target for several anti-tuberculosis (TB) compounds [63–67] and co-crystallized complexes with compounds similar to Sudoterb and other analogues have also been reported [38, 39]. In particular, 17 *M. smegmatis* MmpL3 and one Mtb MmpL3 crystalized structures (6AJF, 6AJG, 6AJH, 6AJI, 6AJJ, 6N40, 6OR2, 7C2M, 7C2N, 7K7M, 7K8A, 7K8B, 7K8C, 7K8D, 7N6B, 7WNX, 8QKK, 7NVH) were found in the PDB, and among them, 7 (6AJG, 6AJH, 6AJI, 6AJJ, 7C2M, 7C2N and 7WNX) were complexed with small molecules with reported *K*_d_s (Table 3).

**Table 3:** The dataset composition for the SB investigations

PDB ID^a^ (mol name)^b^	Mol ID^c^	Chemical structure	K_d_ (µM)	pK_d_^d^
7WNX (ST004)	1I2		9.58 [39]	5.02
7C2M (NITD-349)	FFU		0.05 [68]	7.30
7C2N (SPIRO)	FG0		0.80 [68]	6.10
6AJJ (ICA38)	J9E		0.11 [38]	6.96
6AJH (AU1235)	9ZF		0.20 [38]	6.70
6AJG (SQ109)	3RX		1.80 [38]	5.75
6AJI (Rimonabant)	AY6		41.40 [38]	4.38

a: PDB entry code from https://www.rcsb.org/

b: molecule name given in the reference

c: molecule name as found in the PDB file

d: -log_10_(K_d_) for K_d_ expressed in molar unit

Based on these data and considering that the potency of the co-crystallized MmpL3 spanned almost three log units [69], a structure-based protocol was established to investigate the likely binding mode of **1** and the derivatives **2aj** and **3aj** designed herein. All ligand-co-crystallized MmpL3 proteins were retrieved from the PDB to develop SB-based 3-D QSAR models using the Py-CoMFA and COMBINE paradigms [42, 43].

***SB learning dataset***: the MmpL3 crystal structure complexed with small organic compounds were downloaded, cleaned up, added the hydrogens and subjected to a single point short energy minimization to relax any introduced steric hindrance. Each protein (lock) and ligand (key) of the minimized complexes were then separated and stored into different files and associated with their p*K*_d_ values.

***Molecular docking assessment***: Starting with either the experimental (EC) or randomized (RC) conformation, re-docking (RD) and cross-docking (CD) experiments [70] were performed to select the most suitable docking program among Smina [71] and PLANTS [72, 73]. All possible combinations with the available scoring functions [74, 75] were considered (Table 4 and Supplementary Material Table S28). The four levels of docking assessment clearly indicated that the PLANTS/PLP pair was the most effective, showing the best profile with ECRD, RCRD, ECCD, and RCCD DA% values of 100.00%, 85.71%, 85.71%, and 71.43%, respectively. The PLANTS/PLP combination achieved high accuracy in re-docking (100%) but showed a drop in random conformation cross-docking (71.43%). From the authors best knowledge any CD application reveal a drop of performance if compared to direct RD [70]. This is due to changes in binding site conformations. Nevertheless, although a DA% drop of about 30%, a value of DA% as high as 71.43% should be considered a very good performance. In the investigation of an active molecule, the cross-docking can be considered a measure of the likely correct binding conformation that can be obtained with the validated docking program. Herein, assuming an active compound as a ligand of MmpL3 then the DA% would indicate that the docked conformation would have about 71% of possibility to be the correct one. In particular, as different experimental conformations of the target are used, the cross-docking gives insights on which target conformation the under-investigation molecule might bind. Therefore, in case of the design of novel anti-TB each new compound could be evaluated in the most likely binding site as suggested by the cross-docking experiment.

**Table 4:** Re-docking and cross-docking assessment on the seven MmpL3 complexes

Program^a^	SF^b^	DA %^c^				Average DA%
		ECRD^d^	RCRC^e^	ECCD^f^	RCCD^g^	
PLANTS	chemplp	92.86	92.86	85.71	64.29	83.93
	PLP95	100.00	85.71	85.71	85.71	89.28
	PLP	100.00	85.71	85.71	71.43	85.71
Smina	AD4	50.00	42.86	57.14	64.29	53.57
	Vina	78.57	71.43	71.43	64.29	71.43
	Vinardo	85.71	85.71	71.43	85.71	82.14

a: program used for the docking

b: Scoring Function available within the docking program

c: docking accuracy espressed as percentage [70]

d: experimental conformation re-docking

e: random conformation re-docking

f: experimental conformation cross-docking

g: random conformation cross-docking

***SB 3-D QSAR and COMBINE modeling***: the published *K*_d_s of the co-crystallized molecules (7WNX, 7C2M, 7C2N, 6AJJ, 6AJH, 6AJG, 6AJI) against MmpL3 were collected (Table 3). Despite the limited number of lock/key pairs, the experimental complexes and the associated biological data were used to build preliminary SB 3-D QSAR and COMBINE models to be used as external focused scoring functions to evaluate the putative inhibitory potency of the titled compounds with an unavailable experimental binding mode. The same procedure previously reported was applied [42, 43].

**Fig. 8:**
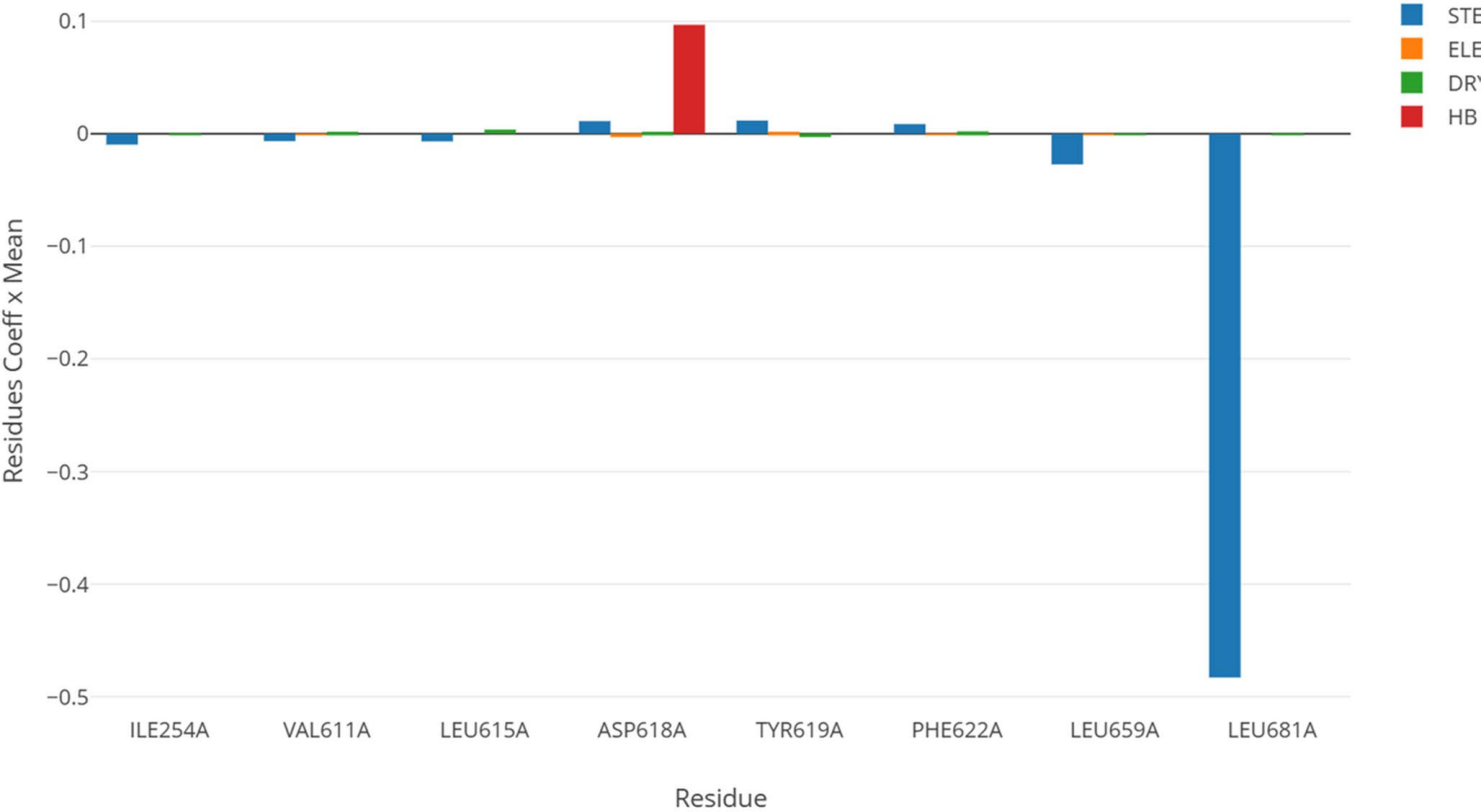
AAC plots relative to the M_COMBINE__4

**Fig. 9:**
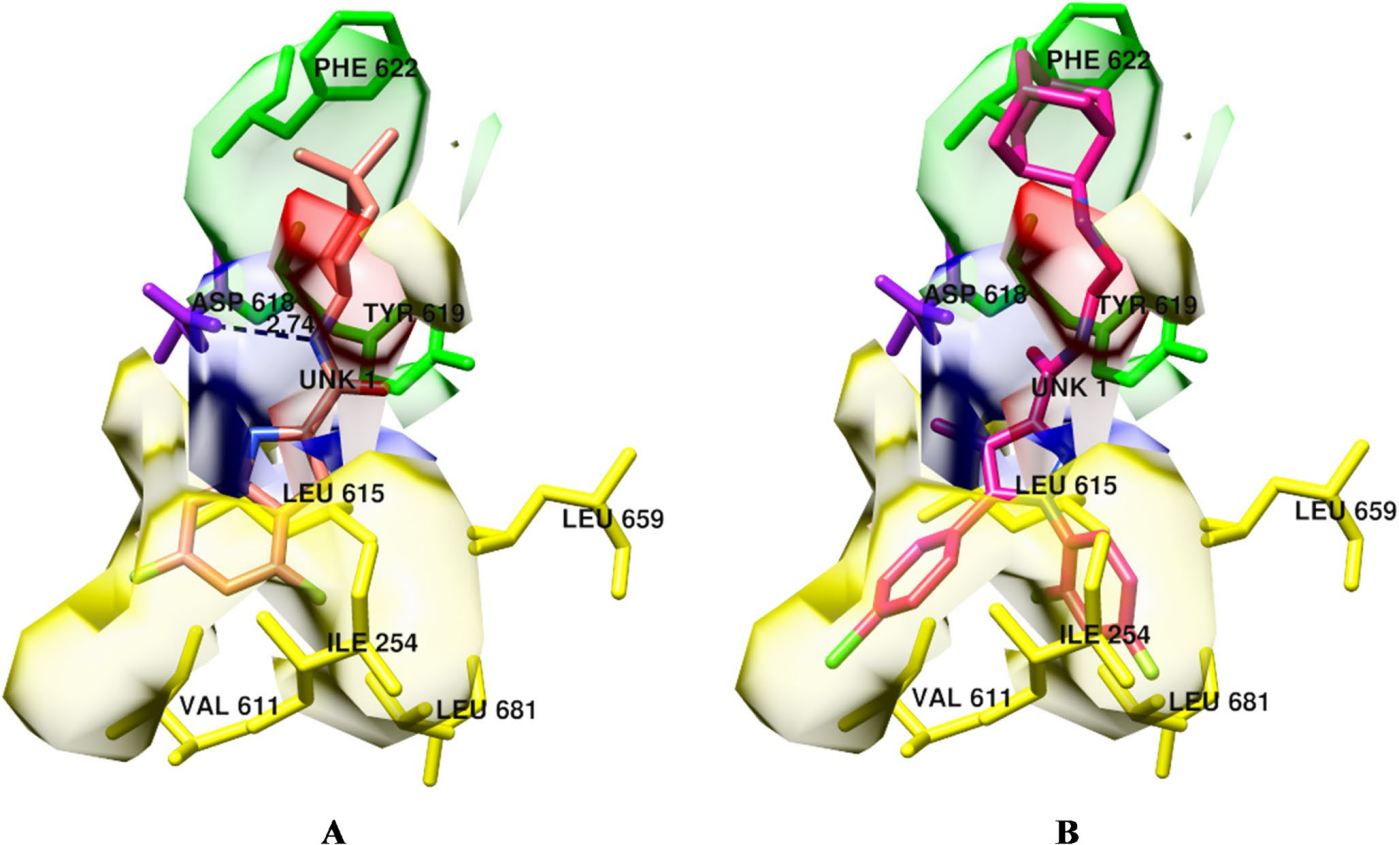
Overlap of the AAC values from CoMFA model M_SB_3-D_QSAR__97_MIN_ and COMBINE M_COMBINE__4 model. Residues are colored as described in the main text. AAC_COMBINE_ values for the hydrogen bonding (HB), the residues were colored in purple. The most potent compound (6C2M) is also reported in panel A, and one of the less potent (7WNX) in panel B. The hydrogen bonding distance of 2.74 Å between 6C2M amide nitrogen and Asp619 carboxylic side chain is also depicted in panel A

- *SB 3-D QSAR*: the molecular alignment was achieved through an SB alignment of all complexes using the matchmaker routine implemented in the UCSF Chimera program [44]. The structure corresponding to the PDB entry code 6AJG was used as a reference because it had the best resolution (2.60 Å). The SB-aligned ligands were then subjected to the same procedure described above for the LB 3-D QSAR modeling. The base model revealed *r*^2^ and *q*^2^_cv_ coefficients of 0.90 and 0.52, respectively, at two PCs (model M_SB_3-D_QSAR__1, model Supplementary Material Tables S29 and S30). To refine the model, 200 random variable pretreatment optimizations (VPO) were also run. The best model was selected based on the highest *q*^2^_cv_ values. Of the 200 randomly generated models through the VPO, M_SB_3-D_QSAR__59 (model Supplementary Material Tables S29 and S31, and Fig. S11) had the highest *q*²_cv_ value (0.73) and was selected for evaluating the MmpL3 affinity of compounds under synthesis.
- *COMBINE*: either the original COMBINE or the actual version of the Py-ComBinE code implemented on 3d-qsar.com require locks with the same length and perfectly aligned residues’ sequences. To overcome this limitation, each of the co-crystallized keys was iteratively merged into each of the locks, and the resulting complex was subjected to a short energy minimization. To prevent any protein unfolding, the complex minimization was performed with the command line version of openbabel [35]. A 5Å shell of explicit water molecules solvation was achieved through the tleap program included in the AmberTools [76] suite (version 19.0). A dataset of minimized complexes was obtained for each lock, and the Py-ComBinE procedure implemented in 3d-qsar.com was applied. A series of COMBINE models was built and evaluated with the *q*^2^_cv_ coefficient. Inspection of the *q*^2^_cv_ values revealed that model M_COMBINE__4, obtained with the training set molecules minimized into the 6AJJ protein, showed the highest *q*^2^_cv_ value (0.62), which was associated with an *r*^2^ of 0.91 (Supplementary Material Table S32). Though the M_COMBINE__4 model was quite good, SA feature selection was applied to achieve a more robust model (M_COMBINE__4_SA_ in Supplementary Material Table S32) characterized by *r*^2^ of and *q*^2^_cv_ values of 0.97 and 0.91, respectively (Supplementary Material Fig. S12). Differently from AAC_SB 3-D QSAR_ values that can be visualized as colored polyhedral, the average activity contribution (AAC_COMBINE_) for the M_COMBINE__4 model and associated with each protein residue could only be bar-plotted and AAC_COMBINE_ plot was generated for the M_COMBINE__4_SA_ model and indicated that the main driving interaction would be addressed to residue Leu681 (panel A of Supplementary Material Fig. S13). Although the SA feature selection led to better coefficients, the resulting model was somehow depleted of information; therefore, AAC plot for the M_COMBINE__4 was also generated (Fig. 8), which confirmed that inhibitory potency is highly dependent on steric interaction with Leu681, while the formation of a hydrogen bond with the side chain of Asp618 should be kept. Modulatory supplemental average activity contributions were also indicated by the M_COMBINE__4 model for Ile254, Val611, Leu615, Tyr619, Phe622, and Leu659. Interestingly, activity contribution plots (AC_COMBINE_) obtained for the most potent training set compound (7CM2) were also investigated and compared to that of one of 7WNX (a low potent co-crystallized inhibitor). These plots give detailed information on the interactions between a given compound and the surrounding residues. For the most active compound, a slightly positive contribution was associated with van der Waals (STE) with Leu618, while other sterical modulatory interactions were recorded for the residues Ile254, Ile258, Asp261, Ile302, and Gly614. Differently, Ser298, Val611, and Tyr619 modulated the 7CM2 potency through desolvation energy (DRY), and only Tyr619 showed a very slight electrostatic modulation power. No hydrogen bonding was shown to have modulating importance (panel A of Supplementary Material Fig. S13). Regarding the compound 7WNX, the AC_COMBINE_ plot indeed highlights that its low potency is mainly due to a steric interaction with Leu681.
- *Combined analysis of SB 3-D QSAR and COMBINE*: The training set molecules’ conformations obtained upon minimization in the 6AJJ protein from which the best COMBINE model was obtained (M_COMBINE__4) was used to train a SB 3-D QSAR model using the same procedure to obtain the above described model M_SB_3-D_QSAR__59 (Supplementary Material Table S33). The VPO optimization led to the selection of model *M*_*SB_3-D_QSAR_*_*97*_*MIN*,_ *for which the AAC were analyzed and superimposed to the binding site extracted from* 6AJJ. Although for the COMBINE, yet it is not possible to easily graphically inspect the AAC and AC data, a sort of workaround was applied to obtain a graphical output by coloring the residues with the absolute highest AAC_COMBINE_ values [77]. Therefore, similar to the classical CoMFA color coding and accordingly with the AAC_COMBINE_ positive or negative values, the residues were colored in green or blue and yellow or red for the steric and electrostatic interactions, respectively. For the hydrogen bonding and desolvation AAC_COMBINE_ values, other colors were used (Fig. 9 and Supplementary Material Fig. S14). In particular, looking at the protein from a side-view with the extracellular portion at the top and the intracellular in the bottom, the negative influencing residues Ile254, Val611, Leu615, Leu659, and Leu681 (colored in yellow in Fig. 9) form a bottom pocket wall preventing the molecules from going further deep into the transmembrane channel. The most potent compound 6C2M perfectly fulfills the pocket with the difluoroindolyl portion, likely avoiding any possible steric clash. Differently, the low active compound 7WNX has the 2,4-difluorophenyl and the 4-chloro-phenyl moieties deep bonded in the pocket, almost reaching a steric clash. Interestingly, negative AAC-STE CoMFA polyhedron are fully overlapped to the negative binding pocket, indicating that any further steric increase would result in a potency decrease.

Differently, the steric positive COMBINE and CoMFA AAC are again in good agreement. In fact, Tyr619 and Phe622 (colored in green in Fig. 9), recognized as van der Waals anchoring points, delineate a small volume perfectly containing a CoMFA AAC green polyhedron and overlapping with 4,4-dimethylcyclohexane and adamantly groups of 6C2M and 7WNX ligands, respectively. Lastly, correctly, the COMBINE AAC highlighted the importance of a hydrogen bonding interaction with Asp618 (colored in purple in Fig. 9). The potent inhibitor 6C2M placed the amide nitrogen at a very strong hydrogen bonding distance (2.74 Å) from the Asp618 carboxylic side chain. On the contrary, compound 7WNX showed a geometry inverted amide group where the amide oxygen points toward the Asp618 carboxylic side chain. These two states are perfectly highlighted by the CoMFA AAC-ELE polyhedron. A blue one (positive contribution) overlapped with both the Asp618 carboxylic side chain and 6C2M amide nitrogen. While a red one (negative contribution) overlaps with 7WNX amide nitrogen, which had no oxygen or basic nitrogen within 3.5 Å.

- *Putative binding mode of the new compounds*: Although no experimental data were available on the molecular mechanism of action, due to some similarity to Mmpl3 co-crystallized compounds (6AJI and 7WNX), the putative binding mode of **1** and that of the newly synthesized compounds **2a-j** and **3a-i** were investigated through molecular docking. In particular, in agreement with the Py-COMBINE results (Table S30) and the docking assessment, the molecules were docked by means of the PLANTS/PLP combination into the MmpL3 using as lock the protein from the 6AJJ PDB entry code. In general, the binding mode of either **1** or any of the newly designed compounds (Supplementary Material Fig. S15) was quite different from that of 7WNX, although they shared some structural similarities (compare the structure of 7WNX with those of **1**, **2a-j**, and **3a-i**).

Compared to the pyrazole derivatives 6AJI and 7WNX, the **1** docked conformation displayed a 180° flipped conformation displaying the N-phenylpyrrole overlapping to the piperidine of 6AJI and the ethylaminoadamantane group of 7WNX, while the substituent in position 3 of the pyrrole overlapped the piperazine ring overlapped to the 6AJI and 7WNX pyrazole rings and the phenyl group bonded to the distal piperazine nitrogen placed the trifluoromethyl group superimposing the chlorine in meta position of the phenyl group bonded in position 5 of both 6AJI and 7WNX. This peculiar binding mode could be due to the greater steric hindrance exerted by the phenylaminocarbonyl substituent in the pyrrole C-5 position of **1**. This resulted in a unique binding mode of the designed compound differently from what expected from a simple structural comparison of **1**, **2a-j**, and **3a-i** with the 6AJI and 7WNX structures.

**Fig. 10:**
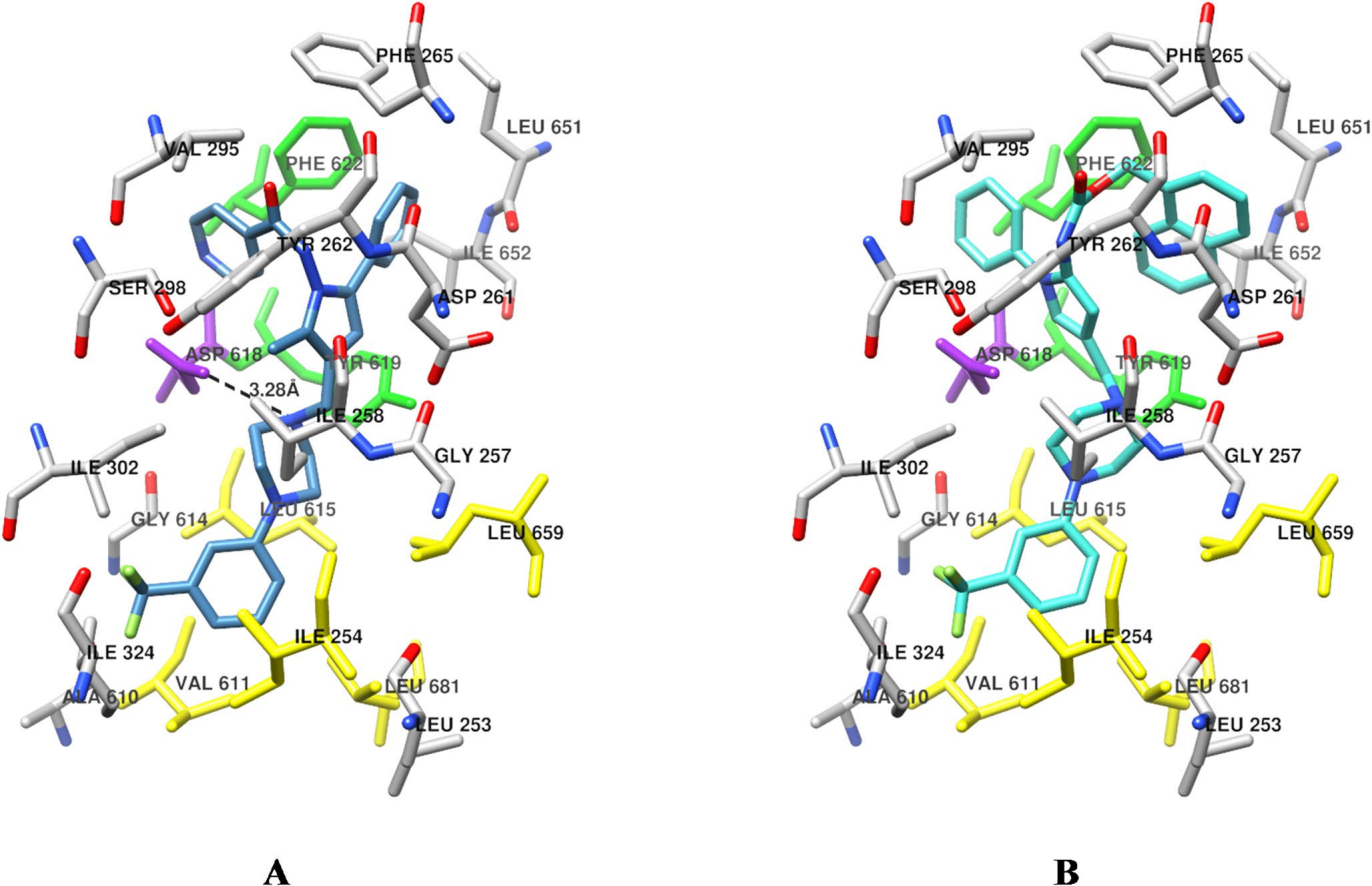
**A** Docked conformation of Sudoterb **1** (carbon atoms colored in Steel Blue). **B** Docked conformation of **3 h** (MC3536, carbon atoms colored in Turquoise)

Interestingly, indicating a docking self-consistency, compound **1** and the designed compounds **2a-j**, and **3a-i** showed an overall similar putative binding mode, placing the 3-trifluorimethylpiperazinyl group in the bottom part overlapping the difluoroindolyl moiety of 6C2M and slightly interacting with the COMBINE negatively designed residues. Depending on the particular docked conformation of each compound, a weak hydrogen bonding distance could be observed between the Asp618 carboxylic side chain and the piperazine nitrogen proximal to the pyrrole ring, as in the case of **1** (panel A of Fig. 10). Differently, the most potent derivative **3 h** (MC3536), used as representative molecule, did not show any producing hydrogen bonding distance (panel B of Fig. 10). Regarding the substituted pyrrole ring, a different binding interaction was observed among **1** and compound **3 h**. In particular, likely due to a direct connection to the piperazinyl, a flexibility reduction resulted for compound **1** so that the phenylaminocarbonyl and the phenyl groups were somehow forced to bind in the top part of the binding site and specifically with the Tyr619 and Phe622 aromatic side chains, likely accounting for a positive binding affinity. Differently, in **3 h**, the introduction of a methylene bridge enhanced the overall molecule flexibility so that the docked conformation displayed a different position of the pyrrole substituents. In particular, the naphthyl group seemed to establish a strong π-π interaction with Phe622. Likely, these structural differences could in part explain the higher microbiological potency of **3 h**.

As all the complexes were related to *M. smegmatis* it was considered to perform molecular dockings also into the available Mtb MmpL3 structure [40] (PDB entry code 7NVH). The 7NVH structure was cleaned and was lacking of any ligand in the site corresponding to that of the SB training set structures. Analysis of the binding site surrounding residues revealed that it was not suitable to perform any plausible docking simulation. Compared to *M. smegmatis* complexes, the Mtb MmpL3 binding site was observed collapsed, leaving no room to host any of the seven ligands extracted from the SB structural dataset (Supplementary Material Fig. S16). Interestingly, comparing the MmpL3 from the two *Mycobacterium* strains share almost the same binding site and notably the *M. smegmatis* unbound proteins binding sites (6AJF, 6N40, 6OR2, 7K7M, 7K8A, 7K8B, 7K8C, 7K8D, 7N6B, 8QKK) well overlapped to that of 7NVH displayed the same collapsed binding site (Supplementary Material Fig. S17). These observations are in good agreement that molecular docking simulation into binding site that haven’t hosted any small molecule can be misleading. Molecular docking using ligand-bound (holo) structures usually outperforms docking using ligand-free (apo) structures being the holo binding pocket geometries ready to bind a molecule [78]. Best practices in docking recommend selecting holo structures and discarding apo structures when possible [79].

**Fig. 11:**
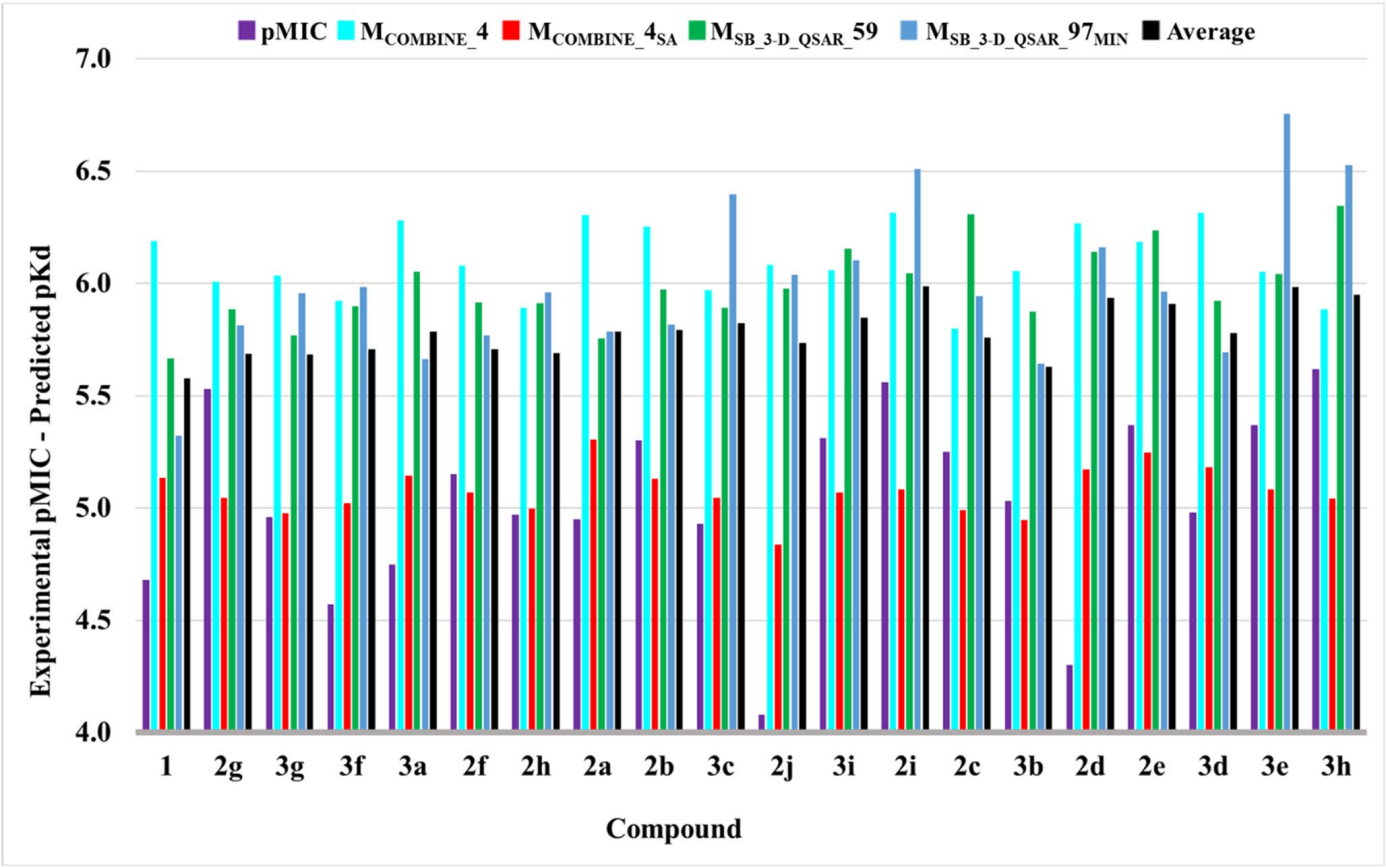
pKd prediction of **1**, **2a-j**, and **3a-i** with the four SB models. For comparison purposes, the pMIC values are also reported. The average predicted p*K*_d_ is also included (black bars)

- *Prediction of protein affinity of new compounds*: the main biological target of **1** and the designed compounds **2a-j** and **3a-i** was considered to be the MmpL3 protein. Due to the SB studies (3-D QSAR and COMBINE), the M_SB_3-D_QSAR__59, M_COMBINE__4, M_COMBINE__4_SA,_ and M_SB_3-D_QSAR__97_MIN_ models were applied to the docked conformations of **1**, **2a-j**, and **3a-i** to predict their biochemical affinities. Of the four models, M_SB_3-D_QSAR__97_MIN_ predicted the pK_d_ profile that showed the best agreement with the pMICs (Fig. 11). Attempts to use all the models via a consensus method by averaging the predicted pK_d_s did not lead to better results.

### Synthesis of 2a-j and 3a-i

**Scheme 1:**
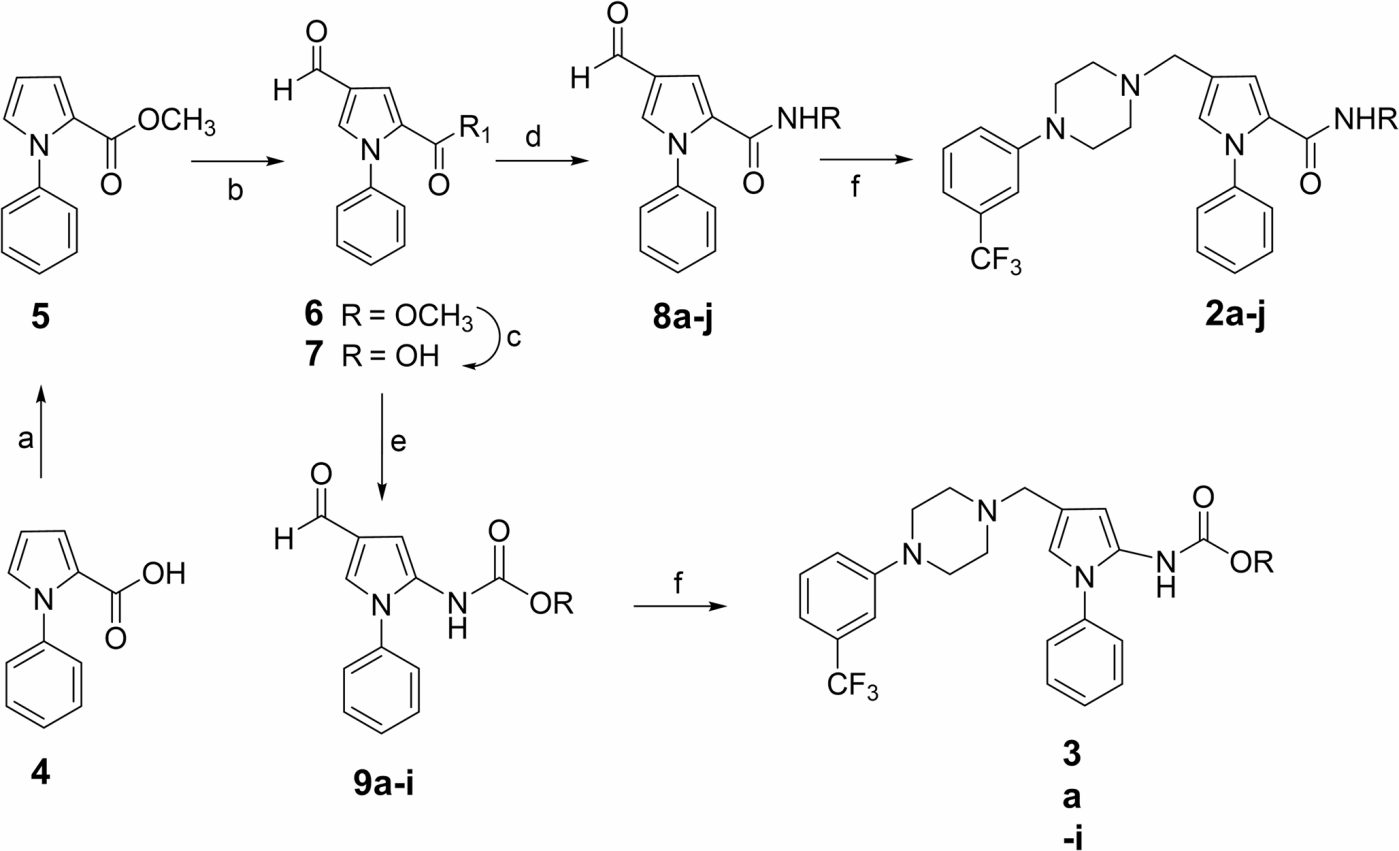
Reagents and conditions: **a** CDI, dry THF, MeOH, rt, 24 h; **b** DMF, POCl_3_, DCE, reflux, 48 h; **c** THF/H_2_O, LiOH, rt, 15 h; **d** (1) SOCl_2_, dry benzene; reflux (2) appropriate amine, dry THF, TEA; **e** DPPA, TEA, dry benzene, appropriate alcohol, *N*_*2*_, reflux 12 h; **f** 1-(3-trifluoromethylphenyl)piperazine, dry DCM, NaBH(O_2_CCH_3_)_3_, rt

The compound **1** analogs **2a-j** and **3a-i** were prepared as outlined in Scheme 1.

In detail, the commercial 1-phenyl-1*H*-pyrrole-2-carboxylic acid **4** was converted into its methyl ester **5** (step a) by treatment with *N*, *N-*carbonyldiimidazole (CDI) in methanol, in mild conditions and at room temperature. The ester **5** underwent a Vielsmeier-Haack reaction (step b), yielding the methyl 4-formyl-1-phenyl-1*H*-pyrrole-2-carboxylate **6**, which was then hydrolyzed in tetrahydrofuran/water using lithium hydroxide (step c), giving the carboxylic acid **7**.

The intermediate **7** was activated to the corresponding acyl chloride with thionyl chloride in dry benzene, and then treated with the appropriate amine in the presence of triethylamine to furnish the amide derivatives **8a-l** (step d).

Alternatively, the carboxylic acid **7** was converted into the carbamates **9a-i** (step e) by applying the Curtius transposition in dry benzene under N_2_, using diphenylphosphoryl azide (DPPA), trimethylamine, and the appropriate alcohol. The reaction of **7** with DPPA furnished the corresponding acyl-azide, not isolated, which, in the presence of the alcohol, underwent transposition giving the required carbamates.

The intermediates **8a-l** and **9a-i** underwent reductive amination (step f) in dry dichloromethane in the presence of 1-(3-trifluoromethylphenyl)piperazine and the reducing agent triacetoxy sodium borohydride, giving the final compounds **2a-l** and **3a-i**, respectively (Scheme 1). Chemical and physical data of the intermediates **5–7**, **8a-j**, **9a-j**, and the final compounds **2a-j**, **3a-i**, as well as elemental analyses for the final compounds, are reported in Tables S1–S6 in Supplementary Material.

### Biology

#### Anti-TB activity of 2a-j and 3a-i

The new pyrrole analogs **2a-j** and **3a-i**, as well as the prototype **1**, were tested in Microplate Alamar Blue Assay (MABA) with a serial dilution starting from 200 µM to determine the minimum inhibitory concentration (MIC) against the Mtb H37Rv strain (Table 5). Rifampicin, Isoniazid, and TMC207 were used as positive controls.

The data (Table 5) showed that nearly all the new amide (**2a-j)** and carbamate (**3a-i)** derivatives, although less potent than the reference drugs, outperformed reference compound **1**, except for **2d**, **2j**, and **3f**, which demonstrated reduced potency (**2d**, **3f**) or were inactive (**2j**). In detail, within the amide series, the antimicrobial activity varied considerably, with MIC values ranging from 2.8 to > 84 µM. The most potent compound was **2i** (MIC = 2.8 µM, 7.4-fold lower than **1**), carrying a 1-naphthyl group at the C2 amide side chain, followed by **2b**, **2c**, and **2e**–**g**, all effective at single-digit micromolar level.

Since **2i**, with its large 1-naphthyl group, exhibited the highest potency, and other amides, such as the *N*-cycloheptyl (**2b**), the *N*-1-adamantanyl (**2e**), and the *N*-4-pyridyl (**2 g**) analogs, also displayed strong anti-TB potency in the range 3.0–5.6 µM, we can speculate that the presence of larger aliphatic or aromatic substituents, in the last case better if heteroaromatic (compare **2 g** with **2f**), could confer a favorable interaction with the putative target consistently enhancing their inhibition. On the other hand, compounds such as the *N*-2-bicyclo[2.2.1]heptanyl-substituted **2d** and the *N*-benzhydryl analog **2j**, with MIC values of 50.6 and > 84 µM, respectively, were significantly less potent or inactive.

The carbamate group (**3a-i**) showed MIC values ranging from 2.4 to 26.8 µM (Table 5). Compounds with 1-naphthylmethyl (**3 h**) and 1-adamantanyl (**3e**) substituents at the oxygen atom of the carbamate function exhibited the highest potency, with MIC values of 2.4 and 4.3 µM, respectively (8.6- and 4.8-fold lower than **1**). Surprisingly, the benzhydryl carbamate **3i** also showed nice inhibitory activity (MIC = 5.0 µM), while its amide counterpart **2j** was inactive. The benzyl-substituted analog **3f**, with a MIC of 26.8 µM, represented the least potent member of the carbamate series.

#### Toxicity of 2a-j and 3a-i in vero cells

Compounds **2a-j** and **3a-i** were screened in Vero cells in 10-dose IC_50_ mode with serial dilution starting from 200 µM solutions to assess their cytotoxicity. Rifampicin and TMC207 were also tested as reference drugs. Looking at the data in Table 5, both the amide (**2a-j**) and carbamate (**3a-i**) series are less cytotoxic than their parent compound **1** and the reference TMC207, but more toxic than rifampicin.

In Vero cells, the amide compounds **2a**–**j** showed generally low cytotoxicity, with IC_50_ values between 33.2 and > 98 µM. Compounds **2i** and **2e**, in addition to being the most potent, also exhibited very low cytotoxicity (IC_50_ around > 90 µM), thus making them the most selective derivatives (selectivity indexes > 33 (**2i**) and > 21 (**2e**).

Carbamate derivatives **3a**–**i** in terms of cytotoxicity generally performed slightly better than the amides, with most of them exhibiting IC_50_ values exceeding 80 µM, indicating low toxicity and a potentially favorable therapeutic profile. As in the case of the amide series, the two most potent anti-TB agents, **3 h** and **3f**, were also among the less toxic, thus being the most selective, with selectivity indexes of > 36 (**3 h**) and > 20 (**3e**).

#### General SAR considerations of 2a-j and 3a-i

The structure–activity relationship analysis revealed that both series contain derivatives with potent antimicrobial activity and low cytotoxicity.

In summary, the detailed SAR analysis above indicates that both series are structurally tunable, as several compounds in both series demonstrated sub-5 µM activity, indicating promising antimicrobial potential combined with a favorable toxicity profile, possessing improved potency and reduced cytotoxicity compared to the reference compound **1**. Overall, the SAR findings underscore compounds **2i** and **3 h** as promising leads due to their low MICs and high IC_50_s.

The SARs herein depicted are in good agreement with the 3-D QSAR and COMBINE models, nevertheless most analogues showed improved potency over the parent compound Sudoterb (**1**) (Table 5) while a few were less potent (**2d** and **3f**).

**Table 5:** Activity of **2a-i** and **3a-i** against *M. tuberculosis* H37Rv and toxicity in Vero cells^a, b^

Lab code	Compd	MIC (µM) MABA^a^	IC_50_ (µM) Vero cell^b^	Selectivity index
MC 3172 (*Sudoterb)*	1	20.7 ± 1.5^c^	33.2 ± 4.1	1.6
MC 3494	2a	11.3 ± 0.05	> 98 (45%)	> 9
MC 3504	2b	5.0 ± 0.07	> 95 (41%)	> 19
MC 3525	2c	5.6 ± 0.1	47.1 ± 5.7	8.4
MC 3528	2d	50.6 ± 8.7	49.9 ± 6.1	1
MC 3529	2e	4.3 ± 0.9	> 89	> 21
MC 3491	2f	7.1 ± 0.8	> 99 (44%)	> 14
MC 3203	2 g	3.0 ± 0.4	38.8 ± 2.9	13.2
MC 3492	2 h	10.6 ± 0.8	41.9 ± 7.4	4.0
MC 3515	2i	2.8 ± 0.04	> 90 (45%)	> 33
MC 3511	2j	> 84	> 84 (40%)	**–**
MC 3270	3a	17.9 ± 0.4	> 100 (40%)	> 6
MC 3526	3b	9.4 ± 0.3	> 92 (42%)	> 10
MC 3505	3c	11.7 ± 1.2	> 90 (40%)	> 8
MC 3533	3d	10.6 ± 0.2	> 90 (45%)	> 9
MC 3535	3e	4.3 ± 0.4	> 84 (47%)	> 20
MC 3233	3f	26.8 ± 10.3	46.7 ± 7.9	1.7
MC 3227	3g	11.0 ± 0.2	39.2 ± 5.0	3.6
MC 3536	3h	2.4 ± 0.1	> 86 (47%)	> 36
MC 3512	3i	5.0 ± 0.1	> 82 (45%)	> 17
**–**	Rifampicin	0.03 ± 0.01	> 122	> 4067
**–**	Isoniazid	0.24 ± 0.06	–	
**–**	TMC207	0.01 ± 0.005	> 28	> 2800

^a^Compounds were tested in an Alamar blue (MABA) assay to evaluate the growth inhibition of Mtb H37Rv strain

^b^Compounds were tested in Vero cells to assess their cytotoxicity

^c^Data were obtained from triplicate test and are reported as average ± standard deviation

## Conclusions

In this study, we tackled the pressing issue of drug-resistant tuberculosis by combining computational modeling with traditional medicinal chemistry to design, synthesize, and assess novel **1** (Sudoterb) analogues. We employed a dual approach, utilizing ligand-based (quantitative structure-activity relationship (QSAR), 3-D QSAR) and structure-based (molecular docking, 3D-structure-based quantitative structure-activity relationship (SB-3-D QSAR), and COMBINE) computational methods to build predictive models for antitubercular activity and prioritize candidate structures. These models revealed key molecular features that influence MIC values and predict binding to the MmpL3 transporter, a validated target for tuberculosis TB.

The computational predictions were then used to assess the designed synthesis of two focused series of analogues: amide (**2a–j**) and carbamate (**3a–i**) derivatives. In more detail, we evaluated 19 of these new **1** analogs to determine how the different substitution patterns on the pyrrole-C2 position, via an amide or carbamate linker, could influence their anti-TB activity against the H37Rv strain, as measured by the minimum inhibitory concentration (MIC) via the MABA test. Many of these compounds, despite being less effective than Rifampicin, Isoniazide, and TMC-207, used as reference drugs, exhibited improved in vitro activity against Mtb H37Rv with respect to the parent compound **1**, with the amides **2i** (MIC: 2.8 µM, 7.4-fold lower than **1**) and **2 g** (MIC: 3.0 µM, 6.9-fold lower), and the carbamates **3 h** (MIC: 2.4 µM, 8.6-fold lower) and **3e** (MIC: 4.3 µM, 4.8-fold lower) being the most potent. These gains in potency were consistent with predictions from the top-performing QSAR and Py-CoMFA models. About cytotoxicity, the majority of compounds of both series displayed low cytotoxicity in Vero cells (IC_50_ >80 µM), indicating a favorable selectivity index and improved safety profile relative to **1**. Moreover, although no experimental data are available on the biochemical mechanism of inhibition, it is assumed that the new compounds would act as inhibitors of the MmpL3 enzyme. SB approaches revealed that the putative target could host the docked conformations of **1**, **2a–j** and **3a–i** with a binding mode different from similar compounds co-crystallized with MmpL3. This alternative binding mode is supported by the affinity prediction (p*K*_d_s) profile, which is in good agreement with that of pMICs.

Overall, this study demonstrates the effectiveness of combining in silico modeling with rational synthetic design.

The herein obtained results will be used for further design by refining the chemical series using the established modeling pipeline and to assess the most potent compounds against a panel of resistant Mtb strains. This iterative design strategy represents a valuable tool in the continued search for more effective and selective antitubercular agents. Both **2a–j** and **3a–i** series offer reliable templates for further optimization, and the SAR-driven refinement of the pyrrole-C2 side chain, with the help of our herein presented computer models, could enhance both efficacy and selectivity of next analogues.

## Experimental section

### Computational medicinal chemistry

The LB and SB procedure was developed through the following points:

#### Dataset composition

The ligand-based training and test sets were compiled from literature (Supplementary Material Tables S1–S9) and the molecules were drawn in the Py-MolEdit module of the 3d-qsar.com portal using the embedded the free for academics Molsoft HTML5 Molecule Editor (https://www.molsoft.com/moledit.html).

#### Generation of 3D conformations for each dataset molecule

Each of the training and test set molecules were stored as SMILES and transformed into 3D local minima conformations by means of the obmin command available in the openbabel [34, 35] software.

#### LB approaches

***QSAR modeling:***

- *Structural descriptors*: several type of FPs were calculated by means of openbabel [34, 35] and PaDEL using a python script.
- *Chemical descriptors*: a number of chemical descriptor were calculated by means of openbabel [34, 35] and PaDEL using a python script.
- *QSAR Modeling*: by means of the python library SciKit-learn [56] PLS-based regression models were developed and validated through cross-validation and external predictions. The most predictive models were used to predict the potencies of the designed compounds.

***3-D QSAR modeling:***

- *Conformational analyses:* Each of the training and test set molecules was subjected to 16 different conformational analyses by means of three different procedures. In particular with the software Balloon and two python scripts, all included in the Py-ConfSearch module of the 3d-qsar.com portal. For each procedure all the available force fields were used (Supplementary Material Table S18).
- *Molecular alignments*: through a list of 17 pre-selected templates from each conformational analysis and the six available molecular alignment software (Tables S19–S23) as embedded in the 3d-qsar.com portal, thousands of combinations were possible. To reduce CPU time 5% of the possible alignments were randomly selected to obtain an equal number of aligned datasets.
- *Calculation of MIFs and Py-CoMFA modeling*: Calculation of MIFs was achieved using the default settings using the Py-CoMFA [33] module of 3d-qsar.com. PLS-based 3-D QSAR models were built for all the aligned datasets and evaluated by *r*^2^, *q*^2^_cv_ and *q*^2^_pred_ values. Since good coefficients level were obtained optimization of the top model was not necessary. The best model was used to predict the MIC of the designed compounds.

#### SB approaches

A structure-based protocol was applied to investigate the likely binding mode of Sudoterb and the herein designed derivatives into MmpL3 as potential biological target [40, 41].

***SB learning dataset***: Through a Blast search run on the PDB database all MmpL3 3D structure complexed with an inhibitor were retrieved and cleaned by mean of the Py-PDB module of 3d-qsar.com. Briefly only one chain was kept and all non-important molecules (solvent and crystallization adjuvants) were deleted. The cleaned complex was the subjected to a single point minimization using the UFF force field available in the openbabel software. The complexes were finally SB aligned using the match-maker routine available in the UCSF Chimera program and stored in separated key and lock files.

***Molecular docking assessment:*** through exhaustive re-docking and cross-docking procedures the programs Smina [71] and PLANTS [72] were used with their available scoring functions to select the combination showing the lowest error in the reproduction of the experimental complexes (Supplementary Material Table S28).

***SB 3-D QSAR and COMBINE modeling***.

- *SB 3-D QSAR*: the ligand extracted from the minimized and aligned complexes were directly used to derive a SB 3-D QSAR model. protein affinities (*K*_d_s) for each ligand were obtained from a literature survey. The model was then subjected to a random VPO procedure in which *r*^2^, *q*^2^_cv_ were optimized by randomly changing the Probe, Charge Model, Min Sigma, Grid Spacing, Grid Extension, Diel Const, CutOff and # Level (Supplementary Material Table S29). The SB 3-D QSAR models was used to evaluate the *K*_d_s of the designed compounds.
- *COMBINE:* the COMBINE procedure, as originally described [31], requires that all proteins should be composed of the same number and positions of the residues. To achieve this condition all SB training set molecules were in turn minimized in all proteins obtained from the minimized and aligned complexes. To this the minimization feature available in the Smina program were used to selectively minimize only the ligands. Through a python script the per residue energy interactions were computed for each ligand, associated to the experimental *K*_d_s and subjected to PLS analysis and cross-validation to build the COMBINE models. The best model was finally used to predict the *K*_d_s of the designed compounds. All the procedure was run through the Py-ComBinE module of the 3d-qsar.com portal.

### Synthesis and characterization

#### General methods

Melting points were determined on a Büchi 530 melting point apparatus and are uncorrected. ^1^H-NMR spectra were recorded at 400 MHz on a Bruker AC 400 spectrometer; chemical shifts are reported in δ (ppm) units relative to the internal reference tetramethylsilane (Me_4_Si). All compounds were routinely checked by TLC and ^1^H-NMR. TLC was performed on aluminum-backed silica gel plates (Merck DC, Alufolien Kieselgel 60 F254) with spots visualized by U.V. light. All solvents were reagent grade and, when necessary, were purified and dried by standard methods. Concentration of solutions after reactions and extractions involved using a rotary evaporator operating at a reduced pressure of ca. 20 Torr. Organic solutions were dried over anhydrous sodium sulfate. Elemental analysis has been used to determine the purity of the described unknown final compounds, which is > 95%. Analytical results are within ± 0.40% of the theoretical values. All chemicals were purchased from Sigma Aldrich s.r.l, Milan (Italy), or TCI Europe N.V., Zwijndrecht (Belgium), and were of the highest purity.

#### Procedure for the preparation of methyl 1-phenyl-1H-pyrrole-2-carboxylate 5 *(step a)*

1-Phenyl-1*H*-pyrrole-2-carboxylic acid **4** (1.21 g) is dissolved in dry THF, and then CDI (1.57 g; 9.69 mmol; 1.5 eq.) was added under magnetic stirring at room temperature. After 30 min, methanol (20 mL) was added, and the reaction was stirred for 3 days. At the end of the reaction, confirmed by TLC using AcOEt as an eluent phase, the solvent was removed under reduced pressure. Water was added to the crude, which was extracted with CHCl_3_ (4 × 25mL). The combined organic layers were washed with brine (1 × 30 mL) and subsequently dried with Na_2_SO_4_, filtered, and concentrated under vacuum, giving the pure compound **5**.

#### Procedure for the preparation of methyl 4-formyl-1-phenyl-1H-pyrrol-2-carboxylate 6 (step b)

*N*,* N*-Dimethylformamide (2.04 mL; 26.42 mmol; 2.25 equiv.) was placed in a reaction flask in the presence of 1,2-dichloroethane (10 mL) with a magnetic stirrer. To the resulting mixture, phosphorus oxychloride (2.4 ml; 26.42 mmol; 2.25 equiv.), dissolved in 1,2-dichloroethane, was added dropwise at 0 °C. Next, methyl 1-phenyl-1*H*-pyrrole-2-carboxylate **7** (5 g), dissolved in dichloroethane, was added dropwise as well. The resulting mixture was refluxed for 2 h and monitored via TLC using 1:5 AcOEt/*n*-hexane as eluent system. Upon completion, 2 N KOH solution (40 mL) was added at 0 °C, followed by extraction with AcOEt (4 × 25 mL). The combined organic layers were washed with brine (1 × 30 mL), dried over Na_2_SO_4_, filtered, and concentrated under vacuum. The crude was then purified using a chromatographic column with 1:5 AcOEt/*n*-hexane as eluent phase, yielding pure compound **6**.

#### Procedure for the preparation of 4-formyl-1-phenyl-1H-pyrrole-2-carboxylic acid 7 (step c)

Methyl 4-formyl-1-phenyl-1*H*-pyrrol-2-carboxylate (700 mg) was hydrolyzed in THF/H_2_O (1:1) in the presence of LiOH (515 mg; 12.24 mmol; 4 equiv.) for 12 h at room temperature. The reaction was monitored by TLC using AcOEt as an eluent phase. Upon completion, the organic solvent was removed by reduced pressure. A 2 N HCl solution was added dropwise to the aqueous layer at 0 °C, resulting in a precipitate that was filtered off, yielding pure compound **7**.

#### General procedure for the synthesis of 4-formyl-1-phenyl-1H-pyrrole-2-carboxamide derivatives (8a-j) (step d)

*Example: synthesis of 4-formyl-N*,*1-diphenyl-1*H*-pyrrol-2-carboxyamide (****8f****)*.

4-Formyl-1-phenyl-1*H*-pyrrole-2-carboxylic acid (70 mg) is dissolved in dry benzene (4 mL) in the presence of SOCl_2_ (0.05 mL; 0.651 mmol; 2 equiv.). After stirring for 45 min, SOCl_2_ is evaporated under vacuum, giving the relative acyl chloride, and without further purification was dissolved in dry THF, and aniline (0.03 mL; 0.357 mmol; 1.1 equiv.) was added in the presence of TEA (0.07 mL; 0.488 mmol; 1.5 equiv.). The reaction is stirred for 30 min and monitored by TLC using 1:2 AcOEt/*n*-hexane as eluent phase. Upon completion, the mixture is quenched with water, and the aqueous phase was extracted with DCM (4 × 25 mL). The combined organic layers were washed with brine (1 × 30mL) and subsequently dried over Na_2_SO_4_, filtered, and concentrated under vacuum. The crude obtained from the extraction was purified over a chromatographic column using 1:2 AcOEt/*n*-hexane as eluent phase, yielding the pure compound **8f**.

#### General procedure for the (4-formyl-1-phenyl-1H-pyrrol-2-yl)carbamate derivatives (9a-i) (step e)

*Example: synthesis of benzyl (4-formyl-1-phenyl-1*H*-pyrrol-2-yl)carbamate* (***9f***).

4-Formyl-1-phenyl-1*H*-pyrrole-2-carboxylic acid (200 mg), TEA (0.15 mL; 1.12 mmol; 1.2 equiv.), DPPA (0.22 mL; 1.02 mmol; 1.1 equiv.), and benzyl alcohol (0.14 mL; 1.39 mmol; 1.5 equiv.) were refluxed for 12 h in dry benzene (5 mL) under an inert atmosphere (N_2_). At the end of the reaction, confirmed by TLC using 1:2 AcOEt/*n*-hexane as eluent phase, the solvent is evaporated under reduced pressure, and the obtained residue was purified over a chromatographic column using 1:2 AcOEt/*n*-hexane as eluent phase, giving the pure compound **9f**.

#### General procedure for the synthesis of the amides 2a-j and the carbamates 3a-i (step h)

*Example: synthesis of* N*-(bicyclo[2.2.1]heptan-2-yl)-1-phenyl-4-((4-(3-(trifluoromethyl)phenyl)piperazin-1-yl)methyl)-1*H*-pyrrole-2-carboxamide (****2d****)*.

The aldehyde **8d** (59 mg) and 1-(3-trifluoromethylphenyl)piperazine (0.04 mL; 0.191 mmol; 1 eq.) were dissolved in dry DCM. After 5 min, NaBH(O_2_CCH_3_)_3_ (80.9 mg; 0.38 mmol; 2 eq.) was added, and the reaction was left overnight and was monitored by TLC using 5:1 AcOEt/*n*-hexane as eluent phase. Upon completion, water was added, and the aqueous phase was extracted with DCM (4 × 25 mL). The combined organic phases are washed with brine (1 × 30 mL) and then dried over Na_2_SO_4_, filtered, and concentrated under vacuum to obtain compound **2d**, which is purified over a chromatographic column using AcOEt/*n*-hexane 5:1 as eluent phase.

*Chemical and physical data of compounds* ***5****–****7***, ***8a****-****j***, ***9a****-****i***, ***2a****-****j***, ***3a****-****i***, *are in Supplementary Material* (Tables S34–S39) while ^1^H NMR descriptions are reported in a separate paragraph.

### Biology

#### Microplate alamar blue assay (MABA) for the evaluation of growth inhibition of *M. tuberculosis* H37Rv

The minimum inhibitory concentration (MIC) for each tested compound was measured in 96-well, flat-bottomed polystyrene microplates using an adapted protocol previously described [80–82]. Briefly, compounds **2a**–**j** and **3a**–**i**, including the parent compound Sudoterb (**1**), were solubilized in DMSO at a concentration of 200 µmol/L and assayed at ten different concentrations (1:2 elution) using the standard limit dilution method. To 95 µL of Middlebrook 7H9 liquid culture medium, 5 µL of the diluted drug was added. Isoniazid, Rifampicin, and TMC-207 were used as positive controls and assayed at eight different concentrations (1:2 elution) from 160 µg/mL, and 5 µL of this control curve was added to 95 µL of Middlebrook 7H9 medium (Difco). 5 µL of DMSO was added to wells containing only 95 µL of Middlebrook 7H9 medium (blank) and to wells containing only the medium with the mycobacteria in the absence of drug, to monitor their growth. The inoculum was standardized to approximately 1 × 107 cfu/mL and diluted 1 in 100 in Middlebrook 7H9 medium to produce the final inoculum of strain H37Rv (ATCC25618). 100 µL of the inoculum was placed in all plate wells except the wells representing the blank. All plates were placed in a sealed box to prevent dehydration of the wells and incubated for 6 days at 37 °C. A resazurin solution was prepared by dissolving one resazurin tablet (resazurin tablets for milk analysis; VWR International Ltd) in 30 mL of sterile PBS (phosphate buffer). Of this solution, 25 µL was added to each well. Fluorescence was measured (Molecular Devices Spectramax M5, excitation 530 nm, emission 590 nm) after 48 h to determine the MIC value. Experiments were done in triplicate.

#### Cytotoxicity in mammalian cells

The cytotoxic effects of the newly synthesized compounds were determined using Vero cells (ATCC CRL-81) as described [82]. The cytotoxicity assessment using Vero cells was designed as a preliminary, standardized host-cell safety screening. As the measured toxicity endpoints are not immune-cell specific, Vero cells are widely accepted for early-stage cytotoxicity testing. Notably, Vero cells are recommended and widely used in WHO- or NIH-guided drug screening frameworks to evaluate mammalian cell toxicity and selectivity indices as reported in “Drugs against parasitic diseases: R&D methodologies and issues“ (TDR/PRD/03.1; WHO, 2003 https://tdr.who.int/docs/librariesprovider10/meeting-reports/drugs-against-diseases-pdf.pdf), by Lucile White et al. [83] and more recently reports [84–86]. Indeed, Oliveira et al. [87] in a systematic review, described the use of Vero cell cytotoxicity as a common initial metric in drug discovery prioritization.

In detail, cells were cultured in Eagle’s minimum essential medium (MEM) containing 10% fetal bovine serum (FBS) plus penicillin and streptomycin. Vero cells were prepared and washed with 0.25% trypsin-EDTA 1× solution in Hanks’ balanced salt solution (HBSS; pH 7.4). After verifying the morphology by microscopy and adjusting the density to 3–5 × 105 cells/mL in MEM, 100 µL of the cell suspension was incubated with the test compounds at 37 °C for 72 h. Then 20 µL of 0.6 mM resazurin was added to each well and incubated for 4 h. The fluorescence was measured at excitation/emission wavelengths of 530/590 nm. The concentration of the test compound effecting a reduction in fluorescence of 50% relative to untreated cells (IC_50_) was calculated. Experiments were done in triplicate.

## Supplementary Information

Below is the link to the electronic supplementary material.

Supplementary Material 1

Supplementary Material 2

Supplementary Material 3

Supplementary Material 4

Supplementary Material 5

Supplementary Material 6

## Data Availability

Upon request to [rino.ragno@uniroma1.it] all data used for the developing the 3-D QSAR, COMBINE models and molecular docking results can be requested. With the data, any non profit user will be enabled to run 3-D QSAR and COMBINE predictions as well as perform molecular docking on the [www.3d-qsar.com] portal.
