# Supplementary material for: Combined computational and classical medicinal chemistry procedure to disclose novel pyrrole-based compounds as potential antituberculosis agents
Source: J Comput Aided Mol Des. 2026 Apr 6;40(1):92. doi: 10.1007/s10822-026-00794-6 (PMC13053371; doi:10.1007/s10822-026-00794-6)
Supplement: Supplementary file 6 — Supplementary Material 6 [file 10822_2026_794_MOESM6_ESM.pdf]

Supplementary Material

Combined Computational and Classical Medicinal Chemistry Procedure to Disclose Novel Pyrrole-Based Compounds as Potential Antituberculosis Agents

Rino Ragno,[1]* Clemens Zwergel,[2] Sergio Valente,[2] Roberta Astolfi,[1] Chiara Lambona,[2] Eleonora Proia,[2] Lidia Giuliani,[2] Scott G. Franzblau,[3] Rossella Fioravanti,[2]* and Antonello Mai[2]

[1]Rome Center for Molecular Design, Department of Drug Chemistry and Technologies, Sapienza University of Rome, Piazzale Aldo Moro 5, 00185 Rome, Italy
[2]Department of Drug Chemistry and Technologies, Sapienza University of Rome, Piazzale Aldo Moro 5, 00185 Rome, Italy
[3]Institute for Tuberculosis Research, Department of Pharmaceutical Sciences, University of Illinois Chicago, 833 South Wood Street, Chicago, IL 60612, United States

Table S1. Dataset composition for the LB elaborations. Starred pMIC values were used as Test Set for the Splitted dataset.

	Molecule ID	SMILES	pMIC
**MCL2022 series** {Semenya, 2022 #88}	**MCL2022_7m**	[NH2+](Cc1cn(c2cc(ccc12)Cl)C/C=C(\C)/CCC=C(C)C)C1CCCCCC1	5.13
	**MCL2022_7l**	[NH2+](Cc1cn(c2cc(ccc12)Cl)CCCCCCCC)[C@H]1CC[C@H](CC1)C	5.40
	**MCL2022_7k**	[NH2+](Cc1cn(c2cc(ccc12)Cl)CCCCCCCC)c1ccccc1	4.11*
	**MCL2022_7j**	[NH2+](Cc1cn(c2cc(ccc12)Cl)CCCCCCCC)C1CCCCCC1	5.63*
	**MCL2022_7i**	[NH2+](Cc1cn(c2cc(ccc12)Cl)C(C)C)C1CCCCCCC1	4.36
	**MCL2022_7h**	[NH2+](Cc1cn(c2cc(ccc12)Cl)C(C)C)C1CCCCCC1	4.27
	**MCL2022_7g**	[NH2+](Cc1cn(c2cc(ccc12)Cl)C(C)C)[C@H]1CC[C@H](CC1)C	4.74
	**MCL2022_7f**	[NH2+](Cc1cn(c2cc(ccc12)Cl)C(C)C)C1CCCCC1	4.06
	**MCL2022_7d**	[NH2+](Cc1cn(c2cc(ccc12)Cl)C)[C@@H]1[C@H]2C[C@H]3C[C@@H]1C[C@H](C3)C2	4.47
	**MCL2022_7c**	[NH2+](Cc1cn(c2cc(ccc12)Cl)C)C1CCCCCCC1	4.04
	**MCL2022_7b**	[NH2+](Cc1cn(c2cc(ccc12)Cl)C)[C@H]1CC[C@H](CC1)C	4.18
	**MCL2022_5l**	[NH2+](Cc1c[nH]c2cc(ccc12)Cl)c1ccccc1	4.14*
	**MCL2022_5k**	[NH2+](Cc1c[nH]c2cc(ccc12)Cl)[C@@H]1[C@H]2C[C@H]3C[C@@H]1C[C@H](C3)C2	4.81
	**MCL2022_5g**	C1CCC(CCC1)[NH2+]Cc1c[nH]c2ccc(cc12)C	4.03
	**MCL2022_5f**	C1CCC(CCC1)[NH2+]Cc1c[nH]c2cc(ccc12)Cl	4.25
	**MCL2022_5d**	[C@H]1(CC[C@H](CC1)[NH2+]Cc1c[nH]c2cc(ccc12)Cl)C	4.06
	**MCL2022_5b**	C1CCC(CC1)[NH2+]Cc1c[nH]c2ccc(cc12)Cl	4.30
**JMC2016 series**{Bhakta, 2016 #89}	**JMC2016_9h**	c1(n(c(cc1C[NH2+]Cc1ccccc1)C)c1ccc(F)cc1)C	5.33*
	**JMC2016_9f**	c1(n(c(cc1C[NH2+]Cc1ccc(cc1)Cl)C)c1ccc(Cl)cc1)C	5.33
	**JMC2016_9e**	c1(n(c(cc1C[NH2+]Cc1ccc(cc1)C)C)c1ccc(Cl)cc1)C	5.33
	**JMC2016_9d**	c1(n(c(cc1C[NH2+][C@@H]1[C@@H]2C[C@@H]3C[C@H]1C[C@H](C2)C3)C)c1ccc(Cl)cc1)C	5.33
	**JMC2016_9c**	c1(n(c(cc1C[NH2+]C1CCCCC1)C)c1ccc(Cl)cc1)C	5.33
	**JMC2016_9b**	c1(n(c(cc1C[NH2+]Cc1ccccc1)C)c1ccc(Cl)cc1)C	5.33*
	**JMC2016_9a**	c1(n(c(cc1C[NH2+]CCc1ccccc1)C)c1ccc(Cl)cc1)C	5.33*
	**JMC2016_7l**	c1(n(c(cc1C[N@H+]1CC[N@@H+](CC1)[C@]12C[C@H]3C[C@@H](C2)C[C@@H](C1)C3)C)c1ccc(Cl)cc1)c1ccc(cc1)Cl	5.33*
	**JMC2016_7k**	c1(n(c(cc1C[N@H+]1CC[N@@H+](CC1)[C@@H]1[C@H]2C[C@H]3C[C@@H]1C[C@@H](C2)C3)c1ccc(cc1)Cl)c1ccc(Cl)cc1)C	5.33
	**JMC2016_7j**	c1(n(c(cc1C[NH+]1CC[NH+](CC1)c1ccccc1)c1ccc(cc1)Cl)c1ccc(Cl)cc1)C	5.33
	**JMC2016_7i**	c1(n(c(cc1C[N@H+]1CC[N@@H+](CC1)[C@]12C[C@H]3C[C@@H](C2)C[C@@H](C1)C3)C)c1ccc(Cl)cc1)C	5.33
	**JMC2016_7h**	c1(n(c(cc1C[N@H+]1CC[N@@H+](CC1)C1CCCCC1)C)c1ccc(Cl)cc1)C	5.33
	**JMC2016_7f**	c1(n(c(c(c1C[NH+]1CC[NH+](CC1)c1ccccc1)C[NH+]1CCN(CC1)c1ccccc1)C)c1ccc(Cl)cc1)C	5.33
	**JMC2016_7e**	c1(n(c(cc1C[NH+]1CC[NH+](CC1)c1ccccc1)C)c1ccc(Cl)cc1)C	5.33*

	**JMC2016_7d**	c1(n(c(cc1C[N@H+]1CC[N@@H+](CC1)[C@@H]1[C@H]2C[C@H]3C[C@@H]1C[C@@H](C2)C3)C)c1cccc(c1)C)C	5.33
	**JMC2016_7c**	c1(n(c(cc1C[N@H+]1CC[N@@H+](CC1)[C@@H]1[C@H]2C[C@H]3C[C@@H]1C[C@@H](C2)C3)C)c1ccc(C(C)C)cc1)C	5.33*
	**JMC2016_7b**	c1(n(c(cc1C[N@H+]1CC[N@@H+](CC1)[C@@H]1[C@H]2C[C@H]3C[C@@H]1C[C@@H](C2)C3)C)c1ccccc1F)C	5.33
	**JMC2016_7a**	c1(n(c(cc1C[N@H+]1CC[N@@H+](CC1)[C@@H]1[C@H]2C[C@H]3C[C@@H]1C[C@@H](C2)C3)C)c1ccc(Cl)cc1)C	5.33
	**JMC2016_2**	CC(=CCC/C(=C\C[NH2+]CC[NH2+][C@H]1[C@H]2C[C@@H]3C[C@H](C2)C[C@H]1C3)/C)C	5.81*
	**JMC2016_1**	c1(n(c(cc1C[N@H+]1CC[N@@H+](CC1)C)c1ccc(Cl)cc1)c1ccc(Cl)cc1)C	5.33
**JMC2008 series**{Biava, [illegible]	**JMC2008_9**	c1(n(c(c(c1)C[NH+]1CCSCC1)C)c1ccc(Cl)cc1)c1ccc(cc1)CC	6.22*
	**JMC2008_8**	c1(n(c(c(c1)C[NH+]1CCSCC1)C)c1ccc(Cl)cc1)c1ccc(cc1)C	5.90*
	**JMC2008_4**	c1(n(c(c(c1)C[NH+]1CCSCC1)C)c1ccc(F)cc1)c1ccc(cc1)C(C)C	6.21
	**JMC2008_15**	c1(n(c(c(c1)C[NH+]1CCSCC1)C)c1ccc(C(C)C)cc1)c1ccc(cc1)Cl	6.23
	**JMC2008_14**	c1(n(c(c(c1)C[NH+]1CCSCC1)C)c1ccc(CCC)cc1)c1ccc(cc1)Cl	6.23*
	**JMC2008_13**	c1(n(c(c(c1)C[NH+]1CCSCC1)C)c1ccc(CC)cc1)c1ccc(cc1)Cl	5.92
	**JMC2008_12**	c1(n(c(c(c1)C[NH+]1CCSCC1)C)c1ccc(C)cc1)c1ccc(cc1)Cl	5.90
	**JMC2008_11**	c1(n(c(c(c1)C[NH+]1CCSCC1)C)c1ccc(Cl)cc1)c1ccc(cc1)C(C)C	6.53*
	**JMC2008_10**	c1(n(c(c(c1)C[NH+]1CCSCC1)C)c1ccc(Cl)cc1)c1ccc(cc1)CCC	6.53
	**JMC2008_1**	c1(n(c(c(c1)C[NH+]1CCSCC1)C)c1ccc(F)cc1)c1ccc(cc1)C	6.48
**EJMC2009** [illegible]	**EJMC2009_2h**	c1(n(c(cc1C[NH+]1CCSCC1)c1c(F)cccc1)c1ccccc1F)CC	4.70
	**EJMC2009_2g**	c1(n(c(cc1C[NH+]1CCSCC1)c1ccc(F)cc1)c1ccccc1F)CC	5.30
	**EJMC2009_2e**	c1(n(c(cc1C[NH+]1CCSCC1)c1ccccc1)c1ccc(F)cc1)CC	4.98*
	**EJMC2009_2d**	c1(n(c(cc1C[NH+]1CCSCC1)c1ccc(F)cc1)c1ccccc1)CC	5.58
	**EJMC2009_2c**	c1(n(c(cc1C[NH+]1CCSCC1)c1ccccc1)c1ccccc1)CC	4.96
	**EJMC2009_2b**	c1(n(c(cc1C[NH+]1CCSCC1)c1ccc(C)cc1)c1ccc(F)cc1)CC	6.20
	**EJMC2009_2a**	c1(n(c(cc1C[N@H+]1CC[N@@H+](CC1)C)c1ccc(Cl)cc1)c1ccc(Cl)cc1)CC	5.03*
	**EJMC2009_1a**	c1(n(c(cc1C[N@H+]1CC[N@@H+](CC1)C)c1ccc(Cl)cc1)c1ccc(Cl)cc1)C	5.62
**CMC2006 series**{Manetti, 2006 #91}	**CMC2006_III**	c1(n(nc(c1C(=O)c1ccccc1)C)c1ccc(Cl)cc1)O	4.10
	**CMC2006_5g**	c1(n(c(cc1/C=N/OCc1ccncc1)C)c1ccc(cc1)F)C	4.11
	**CMC2006_5c**	c1(n(c(cc1/C=N/OCc1cccc(c1)Cl)C)c1ccc(cc1)F)C	3.55
	**CMC2006_5b**	c1(n(c(cc1/C=N/OCc1ccc(cc1)Cl)C)c1ccc(cc1)F)C	3.85
	**CMC2006_3c**	c1(n(c(cc1/C=N/OCc1ccccc1)C)c1ccc(cc1)Cl)C	3.53
	**CMC2006_3b**	c1(n(c(cc1/C=N/OCc1ccccc1)C)c1ccc(cc1)F)C	4.11
	**CMC2006_12h**	c1(n(nc(c1/C(=N/OC)/c1ccc(cc1)Cl)C)c1ccc(cc1)Cl)O	4.18*
	**CMC2006_12d**	c1(n(nc(c1/C(=N/OC)/c1ccc(cc1)Cl)C)c1ccccc1)O	4.13
	**CMC2006_11o**	c1(n(nc(c1C(=O)/C=C/c1ccccc1)C)c1ccccc1)O	3.78
	**CMC2006_11n**	c1(n(nc(c1C(=O)c1ccc2c(c1)cccc2)C)c1ccccc1)O	4.12*
	**CMC2006_11m**	c1(n(nc(c1C(=O)c1cccs1)C)c1ccccc1)O	3.75*
	**CMC2006_11k**	c1(n(nc(c1C(=O)c1ccc(cc1)c1ccccc1)C)c1ccccc1)O	3.85
	**CMC2006_11i**	c1(n(nc(c1C(=O)c1ccccc1)C)c1ccc(cc1)F)OC(=O)c1ccccc1	4.20
	**CMC2006_11h**	c1(n(nc(c1C(=O)c1ccc(cc1)C)C)c1ccccc1)O	3.77
	**CMC2006_11g**	c1(n(nc(c1C(=O)c1ccccc1)C)c1ccccc1)O	3.75*
	**CMC2006_11f**	c1(n(nc(c1C(=O)c1ccc(cc1)Cl)C)c1ccc(cc1)F)O	4.42
	**CMC2006_11e**	c1(n(nc(c1C(=O)c1ccc(cc1)Cl)C)c1ccc(cc1)Cl)O	4.74
	**CMC2006_11d**	c1(n(nc(c1C(=O)c1cc(ccc1)F)C)c1ccccc1)O	4.07*
	**CMC2006_11c**	c1(n(nc(c1C(=O)c1c(cccc1)C)C)c1ccccc1)O	3.77*
	**CMC2006_11a**	c1(n(nc(c1C(=O)c1ccc(cc1)Cl)C)c1ccccc1)O	4.70
**BMC2010** [illegible]	**BMC2010_29**	c1(n(c(c(c1)C[NH+]1CCSCC1)C)c1ccc(cc1)C(C)C)c1ccc(cc1)SC	5.94
	**BMC2010_28**	c1(n(c(c(c1)C[NH+]1CCSCC1)C)c1ccc(cc1)SC)c1ccc(cc1)C(C)C	5.94
	**BMC2010_27**	c1(n(c(c(c1)C[NH+]1CCSCC1)C)c1ccc(cc1)CCC)c1ccc(cc1)SC	5.94
	**BMC2010_26**	c1(n(c(c(c1)C[NH+]1CCSCC1)C)c1ccc(cc1)SC)c1ccc(cc1)CCC	5.94*
	**BMC2010_25**	c1(n(c(c(c1)C[NH+]1CCSCC1)C)c1ccc(cc1)CC)c1ccc(cc1)SC	6.23
	**BMC2010_24**	c1(n(c(c(c1)C[NH+]1CCSCC1)C)c1ccc(cc1)SC)c1ccc(cc1)CC	5.63
	**BMC2010_23**	c1(n(c(c(c1)C[NH+]1CCSCC1)C)c1ccc(cc1)C)c1ccc(cc1)SC	6.21

	**BMC2010_22**	c1(n(c(c(c1)C[NH+]1CCSCC1)C)c1ccc(cc1)SC)c1ccc(cc1)C	5.61
	**BMC2010_21**	c1(n(c(c(c1)C[NH+]1CCSCC1)C)c1ccc(cc1)C(C)C)c1ccc(cc1)OC	6.23*
	**BMC2010_20**	c1(n(c(c(c1)C[NH+]1CCSCC1)C)c1ccc(cc1)OC)c1ccc(cc1)C(C)C	5.93
	**BMC2010_19**	c1(n(c(c(c1)C[NH+]1CCSCC1)C)c1ccc(cc1)CCC)c1ccc(cc1)OC	6.23
	**BMC2010_18**	c1(n(c(c(c1)C[NH+]1CCSCC1)C)c1ccc(cc1)OC)c1ccc(cc1)CCC	5.93*
	**BMC2010_17**	c1(n(c(c(c1)C[NH+]1CCSCC1)C)c1ccc(cc1)CC)c1ccc(cc1)OC	5.91
	**BMC2010_16**	c1(n(c(c(c1)C[NH+]1CCSCC1)C)c1ccc(cc1)OC)c1ccc(cc1)CC	5.91
	**BMC2010_15**	c1(n(c(c(c1)C[NH+]1CCSCC1)C)c1ccc(cc1)C)c1ccc(cc1)OC	5.90
	**BMC2010_14**	c1(n(c(c(c1)C[NH+]1CCSCC1)C)c1ccc(cc1)OC)c1ccc(cc1)C	5.59
	**BMC2010_13**	c1(n(c(c(c1)C[NH+]1CCSCC1)C)c1ccc(cc1)SC)c1ccc(cc1)F	5.62
	**BMC2010_12**	c1(n(c(c(c1)C[NH+]1CCSCC1)C)c1ccc(cc1)F)c1ccc(cc1)SC	6.82
	**BMC2010_11**	c1(n(c(c(c1)C[NH+]1CCSCC1)C)c1c(cccc1)SC)c1ccc(cc1)F	4.71*
	**BMC2010_10**	c1(n(c(c(c1)C[NH+]1CCSCC1)C)c1ccc(cc1)F)c1ccccc1SC	4.41*
	**BMC2010_1**	c1(n(c(c(c1)C[NH+]1CCSCC1)C)c1ccc(F)cc1)c1ccc(cc1)C	6.48*
**BMC2009 series**{Castagnolo, 2009 #93}	**BMC2009_9g**	C1(=O)N(N=C(/C/1=C\N1CCN(CC1)c1occc1)C)c1ccc(Cl)cc1	4.06
	**BMC2009_9f**	C1(=O)N(N=C(/C/1=C\N1CC[N@@H+](CC1)CCO)C)c1ccc(Cl)cc1	3.74
	**BMC2009_9e**	C1(=O)N(N=C(/C/1=C\Nc1ccccc1)C)c1ccc(Cl)cc1	3.69*
	**BMC2009_9d**	C1(=O)N(N=C(/C/1=C\N1CCN(CC1)C(=O)C)C)c1ccc(Cl)cc1	3.73
	**BMC2009_9c**	C1(=O)N(N=C(/C/1=C\N1CCOCC1)C)c1ccc(Cl)cc1	3.68
	**BMC2009_9b**	C1(=O)N(N=C(/C/1=C\N1CC[N@@H+](CC1)C)C)c1ccc(Cl)cc1	3.70
	**BMC2009_9a**	C1(=O)N(N=C(/C/1=C\N1CCN(CC1)c1ccccc1)C)c1ccc(Cl)cc1	3.77*
	**BMC2009_5k**	C1(=O)N(N=C(/C/1=C(/c1ccc(cc1)Cl)\N(C)CCC)C)c1ccc(cc1)Cl	3.80
	**BMC2009_5j**	C1(=O)N(N=C(/C/1=C(/c1ccc(cc1)Cl)\N1CC[C@@H](CC1)C)C)c1ccc(Cl)cc1	3.82*
	**BMC2009_5h**	C1(=O)N(N=C(/C/1=C(/c1ccc(cc1)Cl)\N(C)C)C)c1ccc(cc1)Cl	4.37
	**BMC2009_5g**	C1(=O)N(N=C(/C/1=C(/c1ccc(cc1)Cl)\N1CCOCC1)C)c1ccc(Cl)cc1	5.02
	**BMC2009_5f**	C1(=O)N(N=C(/C/1=C(/c1ccc(cc1)Cl)\N1CC[N@@H+](CC1)C)C)c1ccc(Cl)cc1	5.03*
	**BMC2009_5e**	C1(=O)N(N=C(/C/1=C(/c1ccc(cc1)Cl)\N1CCSCC1)C)c1ccc(Cl)cc1	4.73
	**BMC2009_5d**	C1(=O)N(N=C(/C/1=C(/c1ccc(cc1)Cl)\N(C)C)C)c1ccccc1	4.03
	**BMC2009_5c**	C1(=O)N(N=C(/C/1=C(/c1ccc(cc1)Cl)\N1CCOCC1)C)c1ccccc1	4.38
	**BMC2009_5b**	C1(=O)N(N=C(/C/1=C(/c1ccc(cc1)Cl)\N1CC[N@@H+](CC1)C)C)c1ccccc1	4.69
	**BMC2009_5a**	C1(=O)N(N=C(/C/1=C(/c1ccc(cc1)Cl)\N1CCSCC1)C)c1ccccc1	4.70
**BMC2008 series.**{Castagnolo, 2008 [illegible]	**BMC2008_9f**	c1(=O)n(n(c(c1C(=O)c1ccc(cc1)Cl)C)Cc1ccc(cc1)N(=O)=O)c1ccc(cc1)Cl	4.18*
	**BMC2008_9e**	c1(=O)n(n(c(c1C(=O)c1ccc(cc1)Cl)C)Cc1ccccc1)c1ccc(cc1)Cl	4.44*
	**BMC2008_9d**	c1(=O)n(n(c(c1C(=O)c1ccc(cc1)Cl)C)Cc1ccc(cc1)F)c1ccc(cc1)Cl	4.15*
	**BMC2008_9a**	c1(=O)n(n(c(c1C(=O)c1ccc(cc1)Cl)C)Cc1ccc(cc1)F)c1ccccc1	4.12*
	**BMC2008_8k**	c1(n(nc(c1C(=O)c1ccc(cc1)Cl)c1ccccc1)c1ccc(cc1)C(C)C)O	4.11
	**BMC2008_8j**	c1(n(nc(c1C(=O)c1ccc(cc1)Cl)C)c1ccc(cc1)C(C)C)O	4.35
	**BMC2008_8i**	c1(n(nc(c1C(=O)c1ccc(cc1)Cl)c1ccccc1)c1ccc(cc1)C)O	4.08
	**BMC2008_8h**	c1(n(nc(c1C(=O)c1ccc(cc1)Cl)C)c1ccc(cc1)C)O	4.31
	**BMC2008_8g**	c1(n(nc(c1C(=O)c1ccc(cc1)Cl)C)c1ccc(cc1)Br)O	4.99*
	**BMC2008_8f**	c1(n(nc(c1C(=O)c1ccc(cc1)Cl)c1ccccc1)c1ccc(cc1)Cl)O	4.41
	**BMC2008_8e**	c1(n(nc(c1C(=O)c1ccc(cc1)Cl)C(C)C)c1ccc(cc1)Cl)O	4.07
	**BMC2008_8c**	c1(n(nc(c1C(=O)c1ccc(cc1)Cl)c1ccccc1)c1ccccc1)O	4.07
	**BMC2008_8b**	c1(n(nc(c1C(=O)c1ccc(cc1)Cl)C(C)C)c1ccccc1)O	3.73*

Table S2. Structure of compounds from MCL2022 series[1].

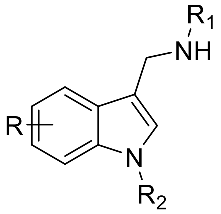

Molecule ID	R	R2	R1
**MCL2022_5b**	5-Cl	H	cyclohexyl
**MCL2022_5d**	6-Cl	H	cyclohexanemethyl
**MCL2022_5f**	6-Cl	H	cycloheptyl
**MCL2022_5g**	5-Me	H	cycloheptyl
**MCL2022_5k**	6-Cl	H	2-adamantyl
**MCL2022_5l**	6-Cl	H	benzyl
**MCL2022_7b**	6-Cl	Me	cyclohexanemethyl
**MCL2022_7c**	6-Cl	Me	cyclooctyl
**MCL2022_7d**	6-Cl	Me	2-adamantyl
**MCL2022_7f**	6-Cl	iPr	cyclohexyl
**MCL2022_7g**	6-Cl	iPr	cyclohexanemethyl
**MCL2022_7h**	6-Cl	iPr	cycloheptyl
**MCL2022_7i**	6-Cl	iPr	cyclooctyl
**MCL2022_7j**	6-Cl	Octyl	cycloheptyl
**MCL2022_7k**	6-Cl	Octyl	benzyl
**MCL2022_7l**	6-Cl	Octyl	cyclohexanemethyl
**MCL2022_7m**	6-Cl	Geranyl	cycloheptyl

Table S3. Structure of compounds from JMC2016 series[2].

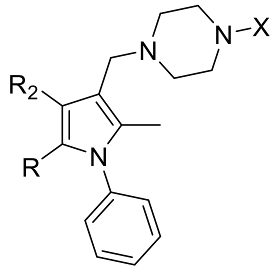

Molecule ID	R	R1	R2	X
**JMC2016_7a**	Me	4-Cl	H	N-(2-adamantyl)piperazinyl
**JMC2016_7b**	Me	2-F	H	N-(2-adamantyl)piperazinyl
**JMC2016_7c**	Me	4-iPr	H	N-(2-adamantyl)piperazinyl
**JMC2016_7d**	Me	3-Me	H	N-(2-adamantyl)piperazinyl
**JMC2016_7e**	Me	4-Cl	H	N-phenylpiperazinyl
**JMC2016_7f**	Me	4-Cl	N-phenylpiperazin-N-ylmethyl	N-phenylpiperazinyl
**JMC2016_7h**	Me	4-Cl	H	N-phenylpiperazin-N-ylmethyl
**JMC2016_7i**	Me	4-Cl	H	N-(1-adamantyl)piperazinyl
**JMC2016_7j**	4-Cl-phenyl	4-Cl	H	N-phenylpiperazinyl
**JMC2016_7k**	4-Cl-phenyl	4-Cl	H	N-(2-adamantyl)piperazinyl
**JMC2016_7l**	4-Cl-phenyl	4-Cl	H	N-(1-adamantyl)piperazinyl
**JMC2016_9a**	Me	4-Cl	H	PhCH2CH2NH-
**JMC2016_9b**	Me	4-Cl	H	PhCH2N-
**JMC2016_9c**	Me	4-Cl	H	cyclohexylNH-
**JMC2016_9d**	Me	4-Cl	H	2-adamantylNH-
**JMC2016_9e**	Me	4-Cl	H	4-Me-BnNH-
**JMC2016_9f**	Me	4-Cl	H	4-Cl-BnNH-
**JMC2016_9h**	Me	4-F	H	PhCH2NH-

Table S4. Structure of compounds from JMC2008 series.

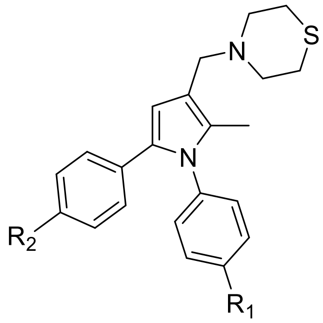

Molecule ID	R1	R2	MICb
**JMC2008_1**	F	methyl	0.25
**JMC2008_4**	F	isopropyl	0.25
**JMC2008_8**	Cl	methyl	0.5
**JMC2008_9**	Cl	ethyl	0.25
**JMC2008_10**	Cl	propyl	0.25
**JMC2008_11**	Cl	isopropyl	0.125
**JMC2008_12**	methyl	Cl	0.5
**JMC2008_13**	ethyl	Cl	0.5
**JMC2008_14**	propyl	Cl	0.25
**JMC2008_15**	isopropyl	Cl	0.25

Table S5. Structure of compounds from EJMC2009 series.

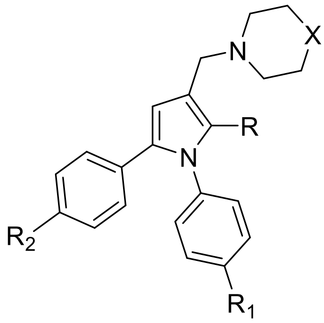

Molecule ID	R	R1	R2	X
**EJMC2009_1a**	CH3	4-Cl	4-Cl	N-CH3
**EJMC2009_2a**	C2H5	4-Cl	4-Cl	N-CH3
**EJMC2009_2b**	C2H5	4-F	4-CH3	S
**EJMC2009_2c**	C2H5	H	H	S
**EJMC2009_2d**	C2H5	H	4-F	S
**EJMC2009_2e**	C2H5	4-F	H	S
**EJMC2009_2f**	C2H5	4-F	4-F	S
**EJMC2009_2g**	C2H5	2-F	4-F	S
**EJMC2009_2h**	C2H5	2-F	2-F	S

Table S6. Structure of compounds from BMC2010 series[3].

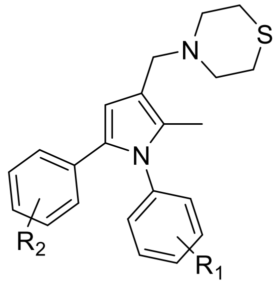

Molecule ID	R1	R2
**BMC2010_1**	4-F	4-CH3
**BMC2010_10**	4-F	2-SCH3
**BMC2010_11**	2-SCH3	4-F
**BMC2010_12**	4-F	4-SCH3
**BMC2010_13**	4-SCH3	4-F
**BMC2010_14**	4-OCH3	4-CH3
**BMC2010_15**	4-CH3	4-OCH3
**BMC2010_16**	4-OCH3	4-C2H5
**BMC2010_17**	4-C2H5	4-OCH3
**BMC2010_18**	4-OCH3	4-C3H7
**BMC2010_19**	4-C3H7	4-OCH3
**BMC2010_20**	4-OCH3	4-i-C3H7
**BMC2010_21**	4-i-C3H7	4-OCH3
**BMC2010_22**	4-SCH3	4-CH3
**BMC2010_23**	4-CH3	4-SCH3
**BMC2010_24**	4-SCH3	4-C2H5
**BMC2010_25**	4-C2H5	4-SCH3
**BMC2010_26**	4-SCH3	4-C3H7
**BMC2010_27**	4-C3H7	4-SCH3
**BMC2010_28**	4-SCH3	4-i-C3H7
**BMC2010_29**	4-i-C3H7	4-SCH3

Table S7. Structure of compounds from BMC2009 series[4].

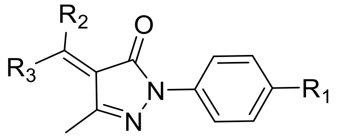

Compound	R	R1
**BMC2009_5a**	H	Thiomorpholine
**BMC2009_5b**	H	*N*-Me-piperazine
**BMC2009_5c**	H	Morpholine
**BMC2009_5d**	H	N(Me)2
**BMC2009_5e**	Cl	Thiomorpholine
**BMC2009_5f**	Cl	*N*-Me-piperazine
**BMC2009_5g**	Cl	Morpholine
**BMC2009_5h**	Cl	N(Me)2
**BMC2009_5i**	Cl	Piperidine
**BMC2009_5j**	Cl	4-Me-piperidine
**BMC2009_5k**	Cl	PrNMe
**BMC2009_7**	Cl	
**BMC2009_9a**	Cl	*N*-Ph-piperazine
**BMC2009_9b**	Cl	*N*-Me-piperazine
**BMC2009_9c**	Cl	Morpholine
**BMC2009_9d**	Cl	*N*-Ac-piperazine
**BMC2009_9e**	Cl	NH-Ph
**BMC2009_9f**	Cl	*N*-(2-Hydroxyethyl)-piperazine
**BMC2009_9g**	Cl	*N*-(2-Furoyl)-piperazine

Table S8. Structure of compounds from BMC2008 series[5].

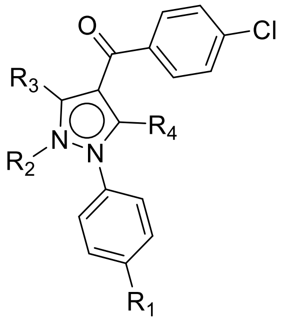

Compound	R1	R2	R3	R4
**BMC2008_8b**	H	-	Isopropyl	OH
**BMC2008_8c**	H	-	Ph	OH
**BMC2008_8e**	Cl	-	Isopropyl	OH
**BMC2008_8f**	Cl	-	Ph	OH
**BMC2008_8g**	Br	-	CH3	OH
**BMC2008_8h**	CH3	-	CH3	OH
**BMC2008_8i**	CH3	-	Ph	OH
**BMC2008_8j**	Isopropyl	-	CH3	OH
**BMC2008_8k**	Isopropyl	-	Ph	OH
**BMC2008_9a**	H	4-F-Bn	CH3	=O
**BMC2008_9d**	Cl	4-F-Bn	CH3	=O
**BMC2008_9e**	Cl	Bn	CH3	=O
**BMC2008_9f**	Cl	4-NO2-Bn	CH3	=O

Table S9. Structure of compounds from CMC2006 series[6].

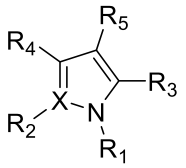

Molecule ID	R1	R2	R3	R4	R5	X
**CMC2006_3b**	4-F-Ph	CH3	CH3	H	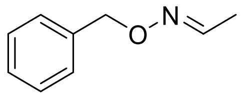	C
**CMC2006_3c**	4-Cl-Ph	CH3	CH3	H	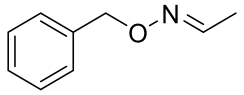	C
**CMC2006_5b**	4-F-Ph	CH3	CH3	H	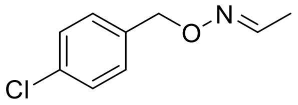	C
**CMC2006_5c**	4-F-Ph	CH3	CH3	H	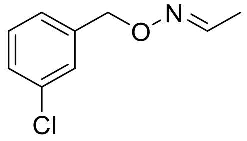	C
**CMC2006_5g**	4-F-Ph	CH3	CH3	H	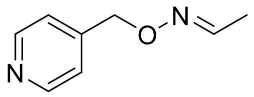	C
**CMC2006_III**	4-Cl-Ph	-	OH	CH3	Benzoyl	N
**CMC2006_11a**	Ph	-	OH	CH3	4-Cl-benzoyl	N
**CMC2006_11c**	2-CH3-Ph	-	OH	CH3	2-CH3-benzoyl	N
**CMC2006_11d**	3-F-Ph	-	OH	CH3	3-F-benzoyl	N
**CMC2006_11e**	4-Cl-Ph	-	OH	CH3	4-Cl-benzoyl	N
**CMC2006_11f**	4-F-Ph	-	OH	CH3	4-Cl-benzoyl	N
**CMC2006_11g**	Ph	-	OH	CH3	benzoyl	N
**CMC2006_11h**	Ph	-	OH	CH3	4-CH3-benzoyl	N
**CMC2006_11j**	Ph	-	4-CH3-benzoyl-O	CH3	4-CH3-benzoyl	N
**CMC2006_11k**	Ph	-		CH3	4-Ph-benzoyl	N
**CMC2006_11m**	Ph	-	H	CH3	2-thienyl-benzoyl	N
**CMC2006_11n**	Ph	-	H	CH3	2-naphthyl-benzoyl	N
**CMC2006_11o**	Ph	-	H	CH3	2-styryl-benzoyl	N
**CMC2006_12d**	Ph	-	H	CH3	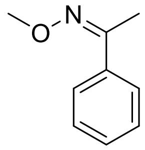	N
**CMC2006_12h**	4-Cl-Ph	-	H	CH3	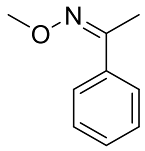	N

Supplementary Material Table S10. Applicability domain outlierness analysis for compounds **2a–j** and **3a–i** using chemical descriptors.

Molecule Name	Distance	Outlierness Score	% Beyond Threshold	Prob Outside
MC3270	7.30	0.94	61.11	0.0000
MC3536	7.40	0.97	63.22	0.0000
MC3505	7.95	1.16	75.47	0.0000
MC3233	5.78	0.42	27.63	0.0042
MC3529	6.23	0.57	37.44	0.0011
MC3512	8.08	1.20	78.23	0.0000
MC3227	6.02	0.50	32.87	0.0021
MC3491	4.87	0.11	7.38	0.0377
MC3492	4.75	0.07	4.88	0.0472
MC3504	6.34	0.61	39.78	0.0008
MC3511	7.14	0.88	57.43	0.0000
MC3533	6.59	0.70	45.48	0.0003
MC3535	7.76	1.09	71.12	0.0000
MC3172	5.86	0.45	29.30	0.0034
MC3203	4.88	0.12	7.71	0.0366
MC3494	5.45	0.31	20.17	0.0102
MC3515	6.70	0.73	47.93	0.0002
MC3525	5.61	0.36	23.74	0.0068
MC3526	7.08	0.86	56.29	0.0001
MC3528	4.98	0.15	9.82	0.0299

Calculated multiple quantitative measures to assess how much a molecule fell outside the applicability domain. The outlierness score provided a normalized measure of deviation from the training set's chemical space, while the percentage beyond threshold indicated the relative extent of deviation compared to the established boundary. The probability estimate gave researchers a likelihood measure of whether a compound truly belonged outside the reliable prediction region. These quantitative measures enabled ranking of compounds by their degree of outlierness, allowing for nuanced decision-making regarding model applicability rather than simple binary classification. The continuous measures provided more detailed information about the reliability of predictions for individual compounds, supporting better risk assessment in modeling applications

Supplementary Material Table S11. Fingerprint-based applicability domain outlierness analysis for compounds **2a–j** and **3a–i**

Molecule Name	Distance from Training Set Center	Outlierness Score	% Beyond Threshold	Minimum Distance Training Set	Maximum Tanimoto Similarity Training
MC3270	3.7213	0	0	7.84	0.20
MC3536	3.6858	0	0	7.06	0.17
MC3505	3.4213	0	0	6.97	0.17
MC3233	4.6326	0	0	6.21	0.19
MC3529	6.1483	0	0	6.63	0.18
MC3512	3.0294	0	0	7.20	0.18
MC3227	9.5684	0	0	8.01	0.20
MC3491	4.0357	0	0	7.52	0.19
MC3492	3.4976	0	0	7.22	0.19
MC3504	3.6841	0	0	7.32	0.18
MC3511	3.1032	0	0	7.45	0.19
MC3533	3.7416	0	0	6.14	0.17
MC3535	6.7124	0	0	5.05	0.18
MC3172	5.7705	0	0	6.61	0.32
MC3203	6.5882	0	0	7.31	0.20
MC3494	3.6841	0	0	7.32	0.18
MC3515	3.9027	0	0	7.03	0.18
MC3525	3.6841	0	0	7.32	0.18
MC3526	3.4213	0	0	6.97	0.17
MC3528	3.6425	0	0	6.67	0.17

Calculated quantitative outlierness measures using molecular fingerprints as the chemical representation. The approach combined fingerprint-based similarity calculations with PCA dimensionality reduction to establish the applicability domain boundaries in a computationally efficient manner. Multiple quantitative measures were provided including distance from the training set center, minimum distance to training compounds, and maximum Tanimoto similarity values. The outlierness score offered a normalized measure of how far each test compound deviated from the established chemical space, while the percentage beyond threshold provided a relative measure of deviation magnitude. The Tanimoto similarity metric specifically addressed the fingerprint-based nature of the approach, giving researchers direct insight into structural similarity relationships between test and training compounds. These quantitative measures enabled precise ranking of compounds by their degree of outlierness and supported evidence-based decisions regarding model applicability for specific molecular structures.

Table S12. QSAR models with the Full dataset

Model #	Descriptor Method	r^2	q^2_{cv}	ONPC
$M_{QSAR_Full_Struct}$_1	Openbabel FP2	0.75	0.70	2
$M_{QSAR_Full_Struct}$_2	Openbabel FP3	0.69	0.66	2
$M_{QSAR_Full_Struct}$_3	Openbabel FP4	0.78	0.73	3
$M_{QSAR_Full_Struct}$_4	Openbabel MACCS	0.74	0.70	2
$M_{QSAR_Full_Struct}$_5	Openbabel Spectrophore	0.44	0.39	3
$M_{QSAR_Full_Struct}$_6	PaDEL AtomPairs2D Fingerprinter	0.85	0.77	4
$M_{QSAR_Full_Struct}$_7	PaDEL Estate Fingerprinter	0.73	0.68	2
$M_{QSAR_Full_Struct}$_8	PaDEL Extended Fingerprinter	0.94	0.80	7
$M_{QSAR_Full_Struct}$_9	PaDEL Fingerprinter	0.95	0.83	7
$M_{QSAR_Full_Struct}$_10	PaDEL GraphOnly Fingerprinter	0.78	0.73	3
$M_{QSAR_Full_Struct}$_11	PaDEL Klekota Roth Fingerprinter	0.80	0.72	3
$M_{QSAR_Full_Struct}$_12	PaDEL MACCS Fingerprinter	0.73	0.69	2
$M_{QSAR_Full_Struct}$_13	PaDEL Pubchem Fingerprinter	0.80	0.74	3
$M_{QSAR_Full_Struct}$_14	PaDEL Substructure Fingerprinter	0.76	0.70	3
$M_{QSAR_Full_Chem_}$1	Openbabel_Descs	0.47	0.45	1
$M_{QSAR_Full_Chem_}$2	PaDEL_Descs	0.57	-0.03	1

Table S13. Details of $M_{QSAR\_Full\_Struct\_9}$ model

PC	r^2	SDEC	q^2_{cv}	SDEP $_{cv}$
1	0.72	0.46	0.69	0.48
2	0.82	0.37	0.74	0.44
3	0.85	0.34	0.77	0.42
4	0.88	0.30	0.78	0.40
5	0.91	0.27	0.80	0.39
6	0.92	0.24	0.80	0.39
7*	0.95	0.20	0.83	0.36
8	0.96	0.22	0.82	0.37

The star indicates the optimal number of PCs selected by the q^2_{cv} max value.

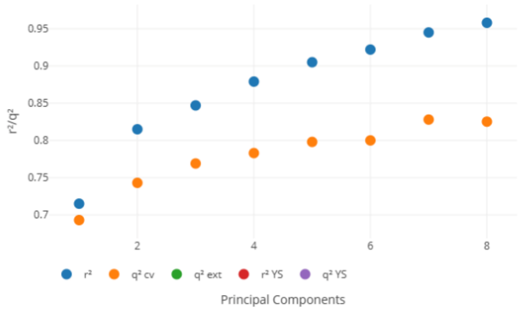

A

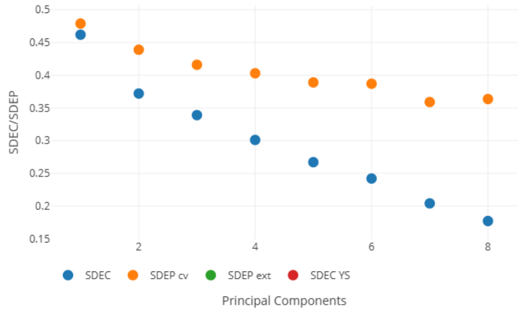

B

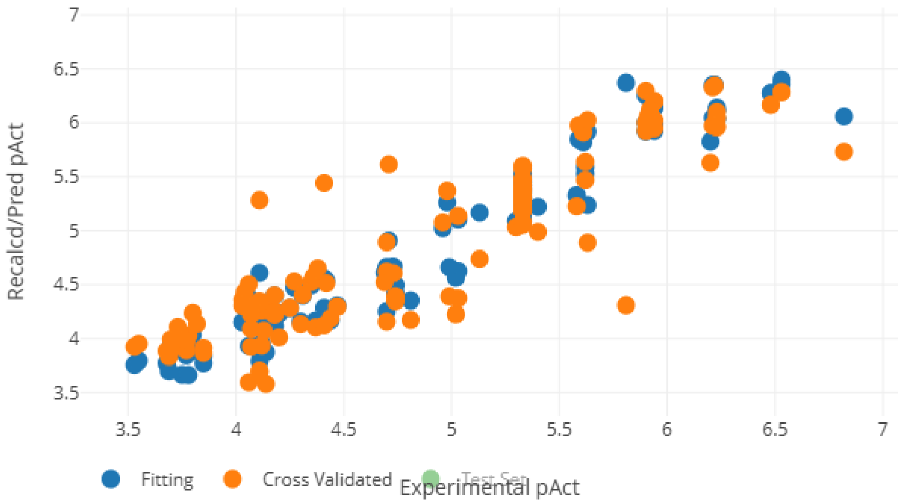

C

Figure S1. Full dataset $M_{QSAR\_Full\_Struct\_9}$ model: (A) r^2 and q^2_{cv} values in relation to number of PCs. (B) SDEC and SDEPcv in relation with the number of PC and the (C) experimental versus the recalculated/CV-predicted pMIC (pAct) values at 7 PCs. In blue the r^2, SDEC and experimental fitted values. In orange the q^2_{cv}, $SDEP_{cv}$ and crossvalidated values.

Table S14. QSAR models with the Splitted dataset

Model #	Descriptor Method	r^2_{cv}	q^2_{cv}	ONPC cv	r^2_{pred}	q^2_{pred}	ONPC ext
$M_{QSAR\_Splitted\_Struct\_}1$	Openbabel FP2	0.83	0.78	3	0.86	0.64	4
$M_{QSAR\_Splitted\_Struct\_}2$	Openbabel FP3	0.71	0.67	2	0.56	0.64	1
$M_{QSAR\_Splitted\_Struct\_}3$	Openbabel FP4	0.82	0.78	3	0.63	0.63	1
$M_{QSAR\_Splitted\_Struct\_}4$	Openbabel MACCS	0.84	0.77	3	0.64	0.64	1
$M_{QSAR\_Splitted\_Struct\_}5$	Openbabel Spectrophore	0.53	0.46	3	0.53	0.23	3
$M_{QSAR\_Splitted\_Struct\_}6$	PaDEL AtomPairs2DFingerprinter	0.92	0.81	6	0.88	0.69	4
$M_{QSAR\_Splitted\_Struct\_}7$	PaDEL EStateFingerprinter	0.77	0.72	2	0.70	0.60	1
$M_{QSAR\_Splitted\_Struct\_}8$	PaDEL ExtendedFingerprinter	0.87	0.81	3	0.89	0.67	4
$M_{QSAR\_Splitted\_Struct\_}9$	PaDEL Fingerprinter	0.92	0.84	5	0.76	0.62	1
$M_{QSAR\_Splitted\_Struct\_}10$	PaDEL GraphOnlyFingerprinter	0.89	0.81	5	0.65	0.60	1
$M_{QSAR\_Splitted\_Struct\_}11$	PaDEL KlekotaRothFingerprinter	0.83	0.75	3	0.83	0.68	3
$M_{QSAR\_Splitted\_Struct\_}12$	PaDEL MACCSFingerprinter	0.84	0.77	3	0.62	0.64	1
$M_{QSAR\_Splitted\_Struct\_}13$	PaDEL PubchemFingerprinter	0.84	0.79	3	0.84	0.63	3
$M_{QSAR\_Splitted\_Struct\_}14$	PaDEL SubstructureFingerprinter	0.79	0.72	3	0.65	0.61	1
$M_{QSAR\_Splitted\_Chem}$	Openbabel_Descs	0.66	0.59	4	0.62	0.40	3

Table S15. Details of $M_{QSAR\_Splitted\_Struct\_6}$ model.

PC	r^2_{cv}	SDEC	q^2_{cv}	$SDEP_{cv}$	q^2_{pred}	$SDEP_{pred}$
1	0.60	0.54	0.56	0.57	0.44	0.67
2	0.76	0.42	0.70	0.47	0.58	0.58
3	0.81	0.37	0.73	0.44	0.61	0.56
4*	0.88	0.29	0.78	0.40	0.69	0.50
5	0.90	0.27	0.79	0.39	0.64	0.54
6	0.92	0.24	0.81	0.37	0.67	0.52
7	0.93	0.23	0.81	0.37	0.67	0.52
8	0.94	0.22	0.82	0.37	0.68	0.51

The star indicates the optimal number of PCs selected by the max q^2_{pred} value.

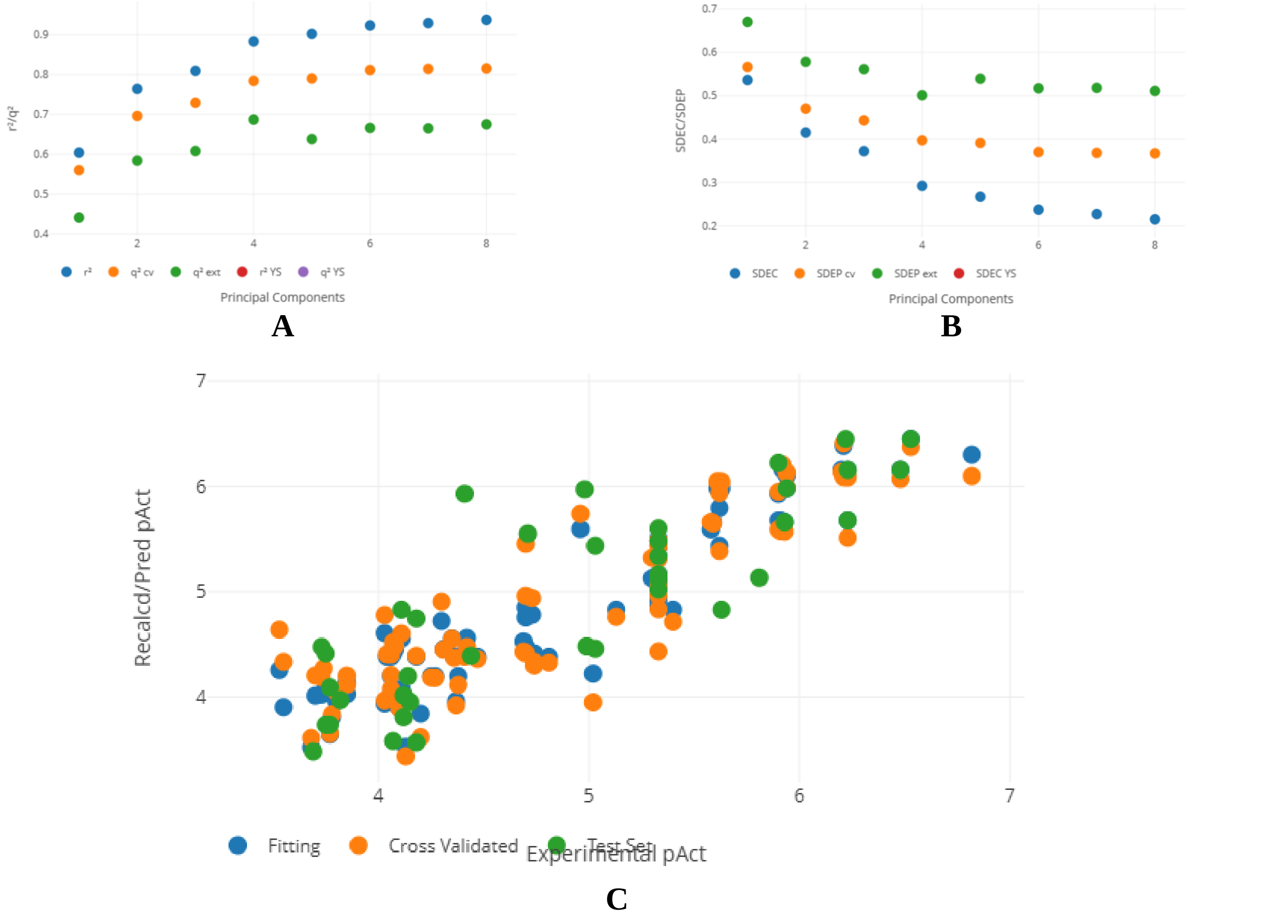

Figure S2. Splitted dataset $M_{QSAR\_Splitted\_Struct\_6}$ model: (A) r^2, q^2_{cv} and q^2_{pred} values in relation to number of PCs. (B) SDEC, $SDEP_{cv}$ and $SDEP_{pred}$ in relation with the number of PC and the (C) experimental versus the recalculated/CV-predicted/Test Set predicted pMIC (pAct) values at 4 PCs. In blue the r^2, SDEC and experimental fitted values. In orange the q^2_{cv}, $SDEP_{cv}$ and crossvalidated values. In green the q^2_{pred}, $SDEP_{pred}$ and Test Set predicted values.

Table S16. Details of $M_{QSAR\_Full\_Chem\_SA}$ model

PC	r^2	SDEC	q^2_{cv}	$SDEP_{cv}$
1	0.57 ± 0.02%	0.57 ± 0.01%	0.26 ± 0.48%	0.75 ± 0.08%
2	0.75 ± 0.02%	0.43 ± 0.04%	0.58 ± 0.43%	0.55 ± 0.28%
3	0.79 ± 0.01%	0.39 ± 0.01%	0.74 ± 0.01%	1.01 ± 0.97%
4	0.85 ± 0.01%	0.34 ± 0.01%	0.78 ± 0.01%	0.41 ± 0.02%
5*	0.89 ± 0.01%	0.29 ± 0.01%	0.80 ± 0.01%	0.38 ± 0.02%

The star indicates the optimal number of PCs selected by the max q^2_{pred} value. The values are averages of three indipendent SA runs.

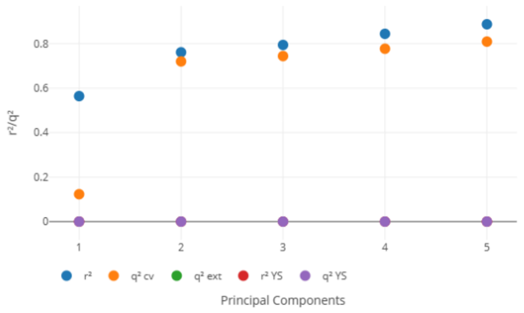

A

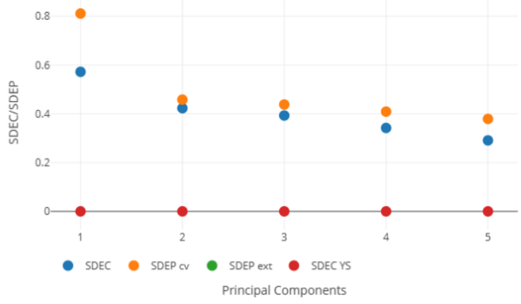

B

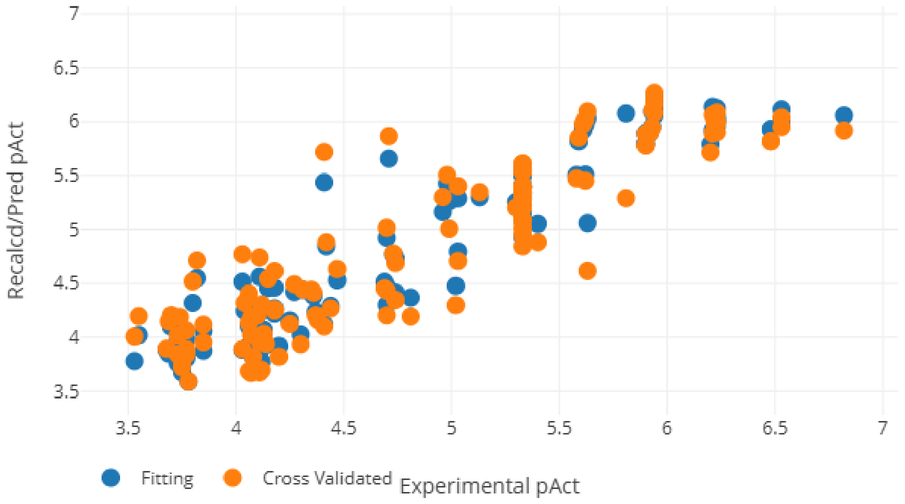

C

Figure S3. Full dataset $M_{QSAR\_Full\_Chem\_SA}$ model: (A) r^2 and q^2_{cv} values in relation to number of PCs. (B) SDEC and SDEPcv in relation with the number of PC and the (C) experimental versus the recalculated/CV-predicted pMIC (pAct) values at 7 PCs. In blue, the r^2, SDEC, and experimental fitted values. In orange, the q^2_{cv}, $SDEP_{cv,}$ and crossvalidated values.

Table S17. Details of $M_{QSAR\_Splitted\_Chem\_SA}$ model.

PC	r^2 cv	SDEC	q^2_{cv}	SDEP cv	q^2_{pred}	SDEP pred
1	0.61 ± 0.01%	0.53 ± 0.01%	0.58 ± 0.01%	0.55 ± 0.01%	0.45 ± 0.02%	0.66 ± 0.01%
2	0.82 ± 0.01%	0.36 ± 0.03%	0.77 ± 0.01%	0.41 ± 0.02%	0.65 ± 0.01%	0.53 ± 0.01%
3	0.84 ± 0.01%	0.34 ± 0.02%	0.78 ± 0.01%	0.40 ± 0.01%	0.61 ± 0.03%	0.56 ± 0.02%
4*	0.90 ± 0.01%	0.28 ± 0.03%	0.78 ± 0.03%	0.40 ± 0.05%	0.67 ± 0.07%	0.51 ± 0.07%
5	0.92 ± 0.00%	0.24 ± 0.02%	0.73 ± 0.17%	0.43 ± 0.23%	0.65 ± 0.07%	0.53 ± 0.06%

The star indicates the optimal number of PCs selected by the max q^2_{pred} value. The values are averages of three indipendent SA runs.

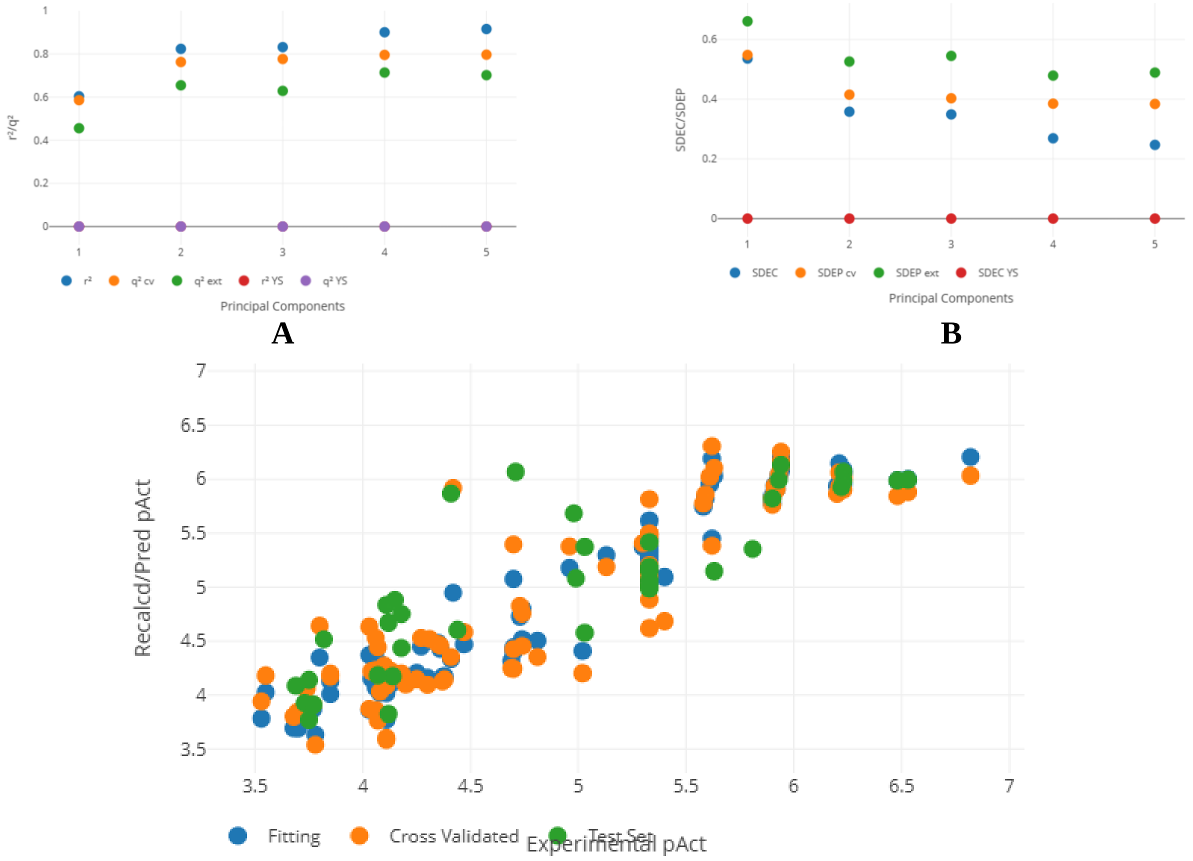

A B

C

Figure S4. Splitted dataset $M_{QSAR\_Splitted\_Chem\_SA}$ model: (A) r^2, q^2_{cv} and q^2_{pred} values in relation to number of PCs. (B) SDEC, $SDEP_{cv}$ and $SDEP_{pred}$ in relation with the number of PC and the (C) experimental versus the recalculated/CV-predicted/Test Set predicted pMIC (pAct) values at 4 PCs. In blue, the r^2, SDEC, and experimental fitted values. In orange, the q^2_{cv}, $SDEP_{cv}$, and crossvalidated values. In green the q^2_{pred}, $SDEP_{pred}$ and Test Set predicted values.

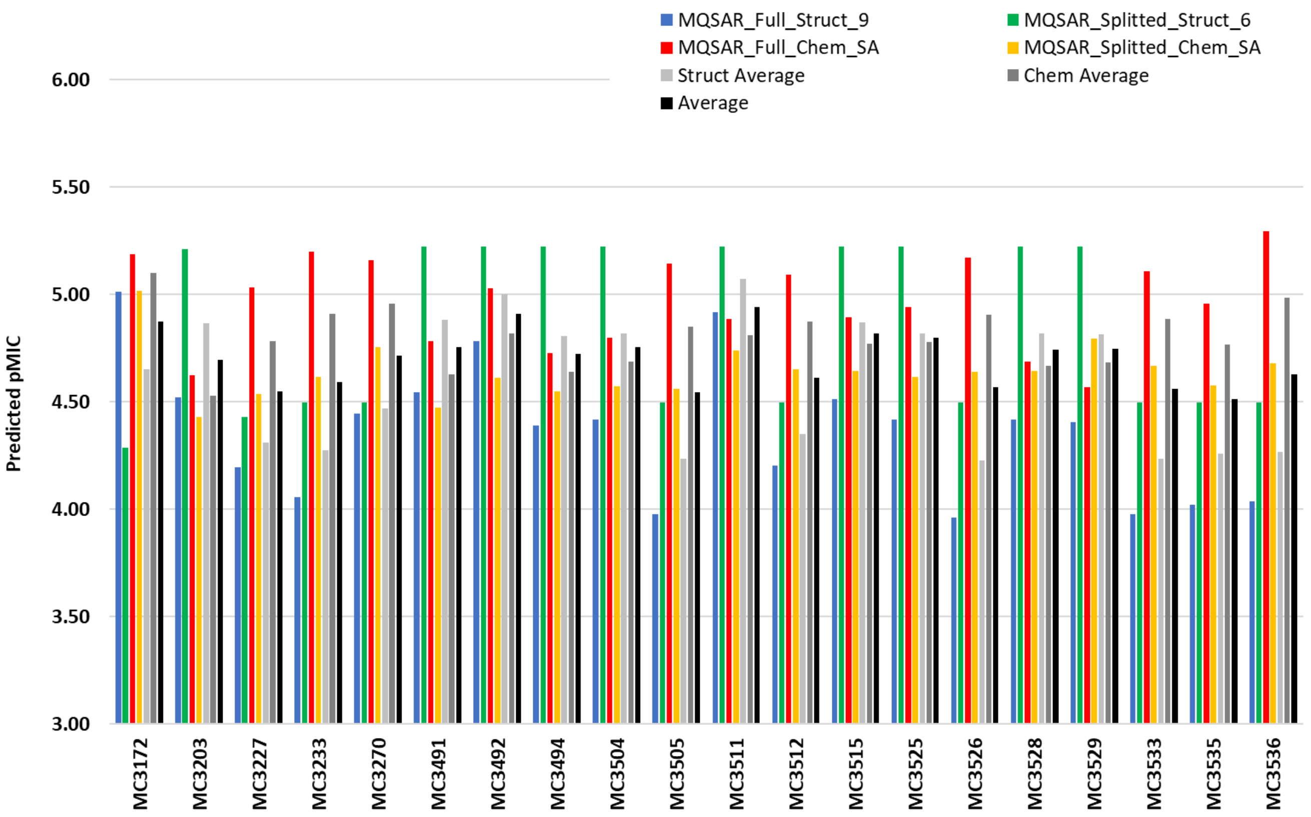

C

Figure S5. pMIC prediction on the under synthesizing compounds with the four final QSAR models.

Table S18. List of conformational analyses performed on the molecules.

#	Method	Force Field	OB Search Method
CA_1	balloon	EEM	NA
CA_2	balloon	MMFF94	NA
CA_3	balloon	SFKEEM	NA
CA_4	RDKit	UFF	NA
CA_5	RDKit	MMFF94	NA
CA_6	RDKit	MMFF94s	NA
CA_7	openbabel	GAFF	Weighted
CA_8	openbabel	GAFF	Random
CA_9	openbabel	UFF	Weighted
CA_10	openbabel	UFF	Random
CA_11	openbabel	MMFF94	Weighted
CA_12	openbabel	MMFF94	Random
CA_13	openbabel	MMFF94s	Weighted
CA_14	openbabel	MMFF94s	Random
CA_15	openbabel	Ghemical	Weighted
CA_16	openbabel	Ghemical	Random

Figure S6. Comparison of the different conformational analyses obtained for the compound MCL2022_7l, the one with the highest number of rotatable bonds takes as an example of conformational variability.

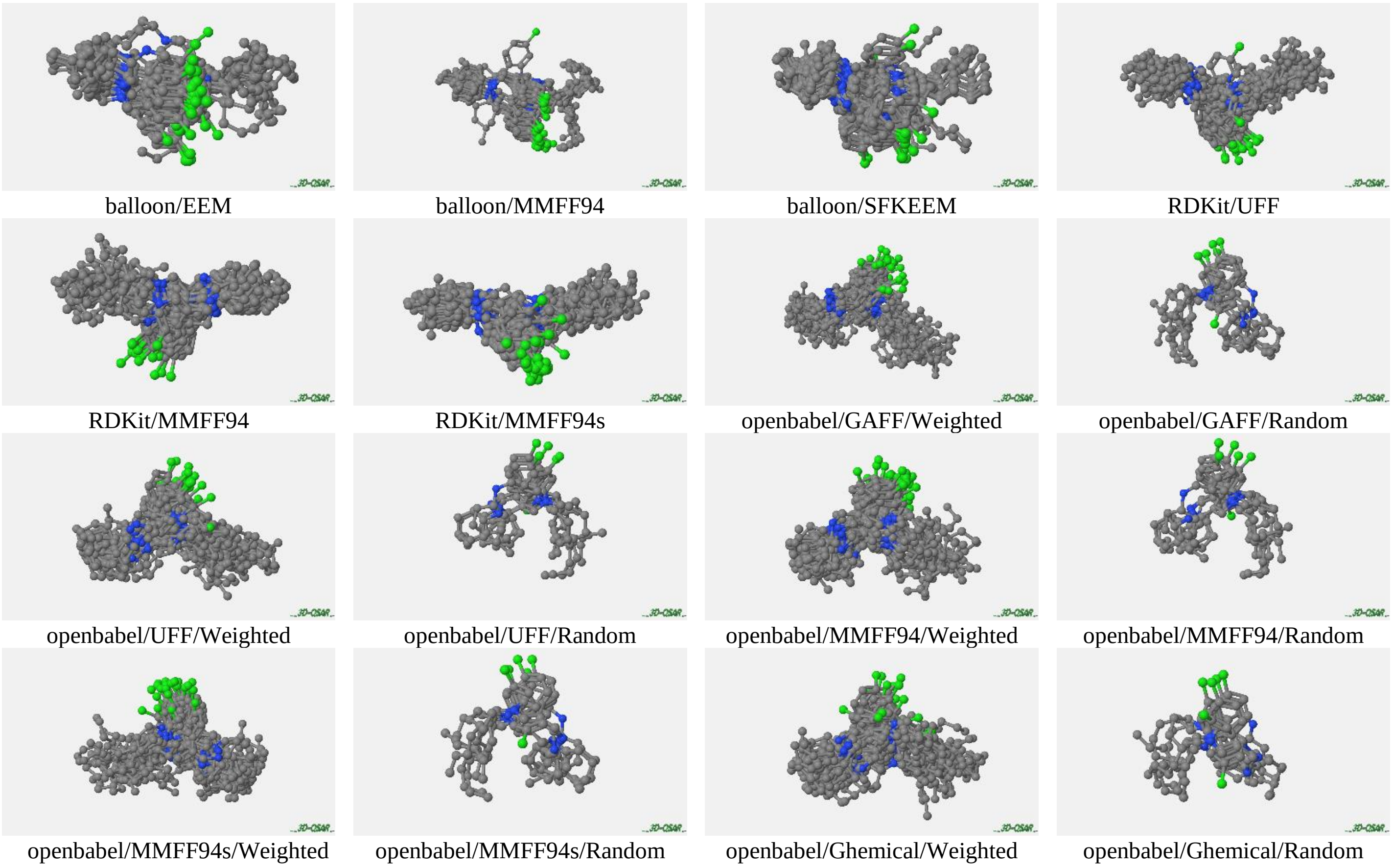

Table S19. Example of list of templates and types of conformations used for the automatic alignments taken from the Full dataset and obtained with the method Balloon and the EMM associated force field.

Property	GM Molecule ID	GM Conf	Longest Molecule ID	Longest Conf	Biggest Molecule ID	Biggest Conf
Least Active	CMC2006_03c	26	CMC2006_03c	13	CMC2006_03c	27
Most Active	BMC2010_12	6	BMC2010_12	24	BMC2010_12	16
Heaviest	JMC2016_07f	5	JMC2016_07f	10	JMC2016_07f	17
Longest	JMC2016_07c	27	ACS_MCL2022_7l	29	JMC2016_07c	18
Most Flexible	JMC2016_02	26	ACS_MCL2022_7l	29	ACS_MCL2022_7l	17
Most Rigid	BMC2009_9c	28	BMC2009_9c	0	BMC2009_9c	28
Least Polar	JMC2008_14	29	JMC2008_14	7	BMC2010_27	0
Most Polar	BMC2008_9f	4	BMC2008_9f	24	BMC2008_9f	12
Biggest	JMC2016_07f	5	JMC2016_07f	10	JMC2016_07f	17
Highest MR	JMC2016_07f	5	JMC2016_07f	10	JMC2016_07f	17
Lowest MR	ACS_MCL2022_5b	26	ACS_MCL2022_5b	17	ACS_MCL2022_5b	8
Highest HA	BMC2008_9f	4	BMC2008_9f	24	BMC2008_9f	12
Lowest HA	JMC2016_02	26	JMC2016_02	11	JMC2016_02	16
Highest HD	JMC2016_07c	27	JMC2016_07c	22	JMC2016_07c	18
Lowest HD	BMC2009_9a	1	BMC2009_9a	8	BMC2009_9a	20
Highest LogP	ACS_MCL2022_7k	22	ACS_MCL2022_7k	16	ACS_MCL2022_7k	17
Lowest LogP	BMC2009_9f	18	BMC2009_9f	8	BMC2009_9f	16

Table S20. List of datasets obtained by the random combination alignment for the Full dataset.

Property	GM Molecule ID	GM Conf	Longest Molecule ID	Longest Conf	Biggest Molecule ID	Biggest Conf
Least Active	CMC2006_03c	26	CMC2006_03c	13	CMC2006_03c	27
Most Active	BMC2010_12	3	BMC2010_12	3	BMC2010_12	22
Heaviest	JMC201s6_07f	1	JMC2016_07f	7	JMC2016_07f	9
Longest	ACS_MCL2022_7l	18	ACS_MCL2022_7m	18	ACS_MCL2022_7l	20
Most Flexible	ACS_MCL2022_7l	18	ACS_MCL2022_7l	16	ACS_MCL2022_7l	20
Most Rigid	BMC2009_9c	5	BMC2009_9c	1	BMC2009_9c	29
Least Polar	JMC2008_10	8	JMC2008_10	13	JMC2008_10	11
Most Polar	CMC2006_11i	26	CMC2006_11i	11	CMC2006_11i	20
Biggest	JMC2016_07f	1	JMC2016_07f	7	JMC2016_07f	9
Highest MR	JMC2016_07f	1	JMC2016_07f	7	JMC2016_07f	9
Lowest MR	ACS_MCL2022_5b	24	ACS_MCL2022_5b	20	ACS_MCL2022_5b	17
Highest HA	BMC2009_9g	14	BMC2009_9g	24	BMC2009_9g	29
Lowest HA	ACS_MCL2022_5k	3	ACS_MCL2022_5f	16	ACS_MCL2022_5k	11
Highest HD	JMC2016_07a	18	BMC2009_9f	19	BMC2009_9f	19
Lowest HD	CMC2006_03c	26	CMC2006_03c	13	CMC2006_03c	27
Highest LogP	ACS_MCL2022_7m	8	ACS_MCL2022_7m	18	ACS_MCL2022_7m	7
Lowest LogP	BMC2009_9f	1	BMC2009_9f	19	BMC2009_9f	19

Table S21. List of alignment methods and available scoring function/settings varies during automatic alignment combination.

Alignment Method	Scoring Function/Settings			
AlignIt[7]	Tanimoto	Tversky		
fkcombu[8]	Atom Flex	Atom Rigid	Volume Flex	Volume Rigid
InterLig[9]				
RDKit[10]	Best Score	Lowest RMSD	Protrude Distance	Tanimoto Distance
Shaep[11]	OnlyShape	Similarity		
ShapeIt[12]	Tanimoto	Tversky		

Table S22. List of alignments for the Full dataset

Align ID	Align Method	SF	Conf Search Method	FF	Ref.Par.	Ref.Mol.	Ref. Conf.	Use Long	Use Big
FA1	fkcombu	Volume Rigid	balloon	MMFF94	Least Active	CMC2006_03c	18	T	F
FA2	Shaep	Similarity	balloon	SFKEEM	Most Active	BMC2010_12	3	F	T
FA3	fkcombu	Volume Rigid	RDKit	UFF	Least Polar	BMC2010_27	17	F	F
FA4	RDKit	Lowest RMSD	RDKit	MMFF94	Lowest HA	JMC2016_02	11	F	T
FA5	AlignIt	Tanimoto	balloon	MMFF94	Highest HD	JMC2016_07c	20	F	T
FA6	RDKit	Best Score	balloon	SFKEEM	Highest MR	JMC2016_07f	28	F	T
FA7	fkcombu	Atom Flex	RDKit	UFF	Most Flexible	ACS_MCL2022_7l	6	T	F
FA8	fkcombu	Atom Flex	RDKit	MMFF94	Most Rigid	BMC2009_9c	22	T	F
FA9	Shaep	OnlyShape	RDKit	UFF	Most Active	BMC2010_12	5	F	T
FA10	AlignIt	Tanimoto	balloon	MMFF94	Highest MR	JMC2016_07f	5	T	F
FA11	fkcombu	Volume Rigid	balloon	SFKEEM	Most Flexible	ACS_MCL2022_7l	12	F	F
FA12	fkcombu	Atom Rigid	RDKit	MMFF94	Most Rigid	BMC2009_9c	20	F	F
FA13	fkcombu	Volume Rigid	RDKit	UFF	Lowest HA	JMC2016_02	25	F	F
FA14	fkcombu	Atom Rigid	balloon	MMFF94	Least Active	CMC2006_03c	17	F	T
FA15	fkcombu	Atom Rigid	balloon	SFKEEM	Least Active	CMC2006_03c	20	F	T
FA16	AlignIt	Tanimoto	RDKit	MMFF94	Most Flexible	ACS_MCL2022_7l	0	T	F
FA17	Shaep	OnlyShape	RDKit	UFF	Lowest LogP	BMC2009_9f	13	F	F
FA18	RDKit	Tanimoto Distance	balloon	MMFF94	Lowest LogP	BMC2009_9f	11	F	T
FA19	AlignIt	Tanimoto	RDKit	MMFF94	Lowest LogP	BMC2009_9f	23	T	F
FA20	AlignIt	Tversky	balloon	SFKEEM	Most Active	BMC2010_12	27	F	F
FA21	RDKit	Lowest RMSD	RDKit	UFF	Highest HD	JMC2016_07f	20	T	F
FA22	AlignIt	Tanimoto	balloon	MMFF94	Most Rigid	BMC2009_9c	13	T	F
FA23	fkcombu	Atom Rigid	RDKit	MMFF94	Highest HA	BMC2008_9f	12	F	F
FA24	RDKit	Tanimoto Distance	balloon	SFKEEM	Least Active	CMC2006_03c	7	F	F
FA25	RDKit	Tanimoto Distance	RDKit	UFF	Lowest MR	ACS_MCL2022_5l	3	F	F
FA26	RDKit	Protrude Distance	balloon	MMFF94	Least Polar	JMC2008_10	8	F	F
FA27	fkcombu	Atom Flex	RDKit	MMFF94	Lowest HD	BMC2009_9a	1	T	F
FA28	Shaep	Similarity	balloon	SFKEEM	Least Active	CMC2006_03c	22	T	F
FA29	AlignIt	Tanimoto	RDKit	UFF	Most Active	BMC2010_12	4	T	F
FA30	RDKit	Tanimoto Distance	balloon	MMFF94	Least Active	CMC2006_03c	18	T	F
FA31	fkcombu	Atom Rigid	RDKit	MMFF94	Most Rigid	BMC2009_9c	22	T	F
FA32	fkcombu	Atom Flex	balloon	SFKEEM	Least Active	CMC2006_03c	20	F	T
FA33	fkcombu	Atom Flex	RDKit	UFF	Lowest HA	JMC2016_02	25	F	F
FA34	AlignIt	Tversky	balloon	MMFF94	Lowest LogP	BMC2009_9f	11	F	T
FA35	RDKit	Best Score	RDKit	MMFF94	Lowest MR	ACS_MCL2022_5l	22	F	T
FA36	ShapeIt	Tversky	balloon	SFKEEM	Highest MR	JMC2016_07f	25	F	F
FA37	RDKit	Best Score	RDKit	UFF	Most Rigid	BMC2009_9c	6	F	T
FA38	RDKit	Tanimoto Distance	RDKit	MMFF94	Lowest LogP	BMC2009_9f	1	F	F
FA39	AlignIt	Tversky	balloon	MMFF94	Least Active	CMC2006_03c	24	F	F
FA40	RDKit	Lowest RMSD	RDKit	UFF	Lowest HA	JMC2016_02	1	T	F
FA41	RDKit	Tanimoto Distance	balloon	SFKEEM	Lowest MR	ACS_MCL2022_5b	20	T	F
FA42	RDKit	Best Score	RDKit	MMFF94	Most Active	BMC2010_12	4	F	F
FA43	AlignIt	Tversky	balloon	MMFF94	Lowest LogP	BMC2009_9f	14	T	F
FA44	Shaep	Similarity	RDKit	UFF	Most Flexible	ACS_MCL2022_7l	6	F	F
FA45	RDKit	Best Score	balloon	SFKEEM	Least Active	CMC2006_03c	7	F	F
FA46	RDKit	Tanimoto Distance	RDKit	MMFF94	Lowest HD	BMC2009_9a	1	T	F
FA47	fkcombu	Atom Flex	RDKit	UFF	Least Active	CMC2006_03c	0	T	F
FA48	Shaep	OnlyShape	balloon	MMFF94	Most Active	BMC2010_12	22	T	F
FA49	fkcombu	Volume Rigid	balloon	SFKEEM	Most Active	BMC2010_12	27	F	F
FA50	RDKit	Lowest RMSD	RDKit	MMFF94	Least Polar	JMC2008_14	7	T	F
FA51	fkcombu	Atom Flex	RDKit	UFF	Lowest HD	BMC2009_9a	17	F	T
FA52	AlignIt	Tanimoto	balloon	MMFF94	Highest LogP	ACS_MCL2022_7k	11	T	F
FA53	fkcombu	Volume Rigid	balloon	SFKEEM	Most Rigid	BMC2009_9c	7	T	F
FA54	fkcombu	Volume Flex	RDKit	UFF	Lowest LogP	BMC2009_9f	26	F	T
FA55	InterLig		RDKit	MMFF94	Lowest LogP	BMC2009_9f	23	T	F
FA56	RDKit	Best Score	balloon	MMFF94	Most Flexible	ACS_MCL2022_7l	26	T	F
FA57	RDKit	Best Score	balloon	SFKEEM	Highest LogP	ACS_MCL2022_7k	27	F	F
FA58	fkcombu	Volume Rigid	RDKit	MMFF94	Lowest LogP	BMC2009_9f	23	T	F

Table S22. List of alignments for the Full dataset

Align ID	Align Method	SF	Conf Search Method	FF	Ref.Par.	Ref.Mol.	Ref. Conf.	Use Long	Use Big
FA59	RDKit	Best Score	RDKit	UFF	Most Rigid	BMC2009_9c	29	T	F
FA60	RDKit	Best Score	balloon	MMFF94	Most Active	BMC2010_12	22	T	F
FA61	Shaep	Similarity	balloon	SFKEEM	Lowest HD	BMC2009_9a	11	F	F
FA62	fkcombu	Volume Flex	RDKit	MMFF94	Least Active	CMC2006_03c	13	F	T
FA63	fkcombu	Atom Flex	RDKit	UFF	Most Rigid	BMC2009_9c	29	T	F
FA64	ShapeIt	Tanimoto	balloon	MMFF94	Highest HA	BMC2008_9f	2	T	F
FA65	AlignIt	Tanimoto	RDKit	MMFF94	Highest LogP	ACS_MCL2022_7m	2	F	F
FA66	RDKit	Lowest RMSD	RDKit	UFF	Least Active	CMC2006_03c	19	F	T
FA67	AlignIt	Tanimoto	balloon	SFKEEM	Highest HA	BMC2008_9f	25	F	T
FA68	ShapeIt	Tanimoto	balloon	MMFF94	Lowest MR	ACS_MCL2022_5b	29	F	T
FA69	Shaep	Similarity	RDKit	MMFF94	Highest LogP	ACS_MCL2022_7m	2	F	F
FA70	ShapeIt	Tanimoto	RDKit	UFF	Highest HA	BMC2008_9f	21	F	F
FA71	fkcombu	Atom Rigid	balloon	SFKEEM	Most Flexible	ACS_MCL2022_7l	17	T	F
FA72	fkcombu	Atom Flex	balloon	MMFF94	Highest HA	BMC2008_9f	8	F	T
FA73	Shaep	Similarity	RDKit	UFF	Highest HD	JMC2016_07f	18	F	T
FA74	Shaep	OnlyShape	RDKit	MMFF94	Least Active	CMC2006_03c	6	F	F
FA75	RDKit	Best Score	openbabel	MMFF94/Weighted	Highest HA	BMC2008_9f	4	F	F
FA76	RDKit	Best Score	balloon	SFKEEM	Lowest LogP	BMC2009_9f	24	T	F
FA77	RDKit	Protrude Distance	balloon	MMFF94	Most Rigid	BMC2009_9c	13	F	F
FA78	InterLig		RDKit	UFF	Lowest HA	JMC2016_02	25	F	F
FA79	fkcombu	Atom Flex	RDKit	MMFF94	Most Active	BMC2010_12	6	T	F
FA80	RDKit	Protrude Distance	openbabel	MMFF94/Weighted	Least Active	CMC2006_03c	5	F	F
FA81	RDKit	Lowest RMSD	balloon	SFKEEM	Most Rigid	BMC2009_9c	10	F	F
FA82	RDKit	Protrude Distance	balloon	MMFF94	Highest HD	JMC2016_07c	3	F	F
FA83	AlignIt	Tversky	RDKit	UFF	Longest	JMC2016_07c	11	T	F
FA84	RDKit	Tanimoto Distance	balloon	SFKEEM	Most Rigid	BMC2009_9c	10	F	F
FA85	InterLig		openbabel	MMFF94/Weighted	Lowest MR	ACS_MCL2022_5l	7	F	F
FA86	ShapeIt	Tanimoto	RDKit	UFF	Highest HA	BMC2008_9f	22	F	T
FA87	fkcombu	Atom Rigid	RDKit	MMFF94	Highest HA	BMC2008_9f	2	T	F
FA88	fkcombu	Atom Flex	balloon	SFKEEM	Highest MR	JMC2016_07f	6	T	F
FA89	fkcombu	Atom Rigid	RDKit	UFF	Highest LogP	ACS_MCL2022_7m	2	F	F
FA90	RDKit	Lowest RMSD	openbabel	MMFF94/Weighted	Lowest LogP	BMC2009_9f	8	F	F
FA91	ShapeIt	Tanimoto	RDKit	MMFF94	Highest LogP	ACS_MCL2022_7m	2	F	F
FA92	RDKit	Best Score	balloon	MMFF94	Lowest HD	BMC2009_9a	4	F	F
FA93	RDKit	Lowest RMSD	balloon	SFKEEM	Lowest MR	ACS_MCL2022_5b	20	T	F
FA94	RDKit	Protrude Distance	RDKit	UFF	Lowest MR	ACS_MCL2022_5l	3	F	F
FA95	fkcombu	Atom Flex	RDKit	MMFF94	Lowest HD	BMC2009_9a	6	F	F
FA96	fkcombu	Volume Flex	openbabel	MMFF94/Weighted	Highest HA	BMC2008_9f	7	T	F
FA97	ShapeIt	Tversky	balloon	MMFF94	Highest MR	JMC2016_07f	25	F	F
FA98	RDKit	Tanimoto Distance	RDKit	UFF	Highest HD	JMC2016_07f	20	T	F
FA99	RDKit	Best Score	RDKit	MMFF94	Lowest LogP	BMC2009_9f	1	F	F
FA100	InterLig		balloon	SFKEEM	Least Polar	BMC2010_27	27	F	F
FA101	fkcombu	Atom Rigid	openbabel	MMFF94/Weighted	Highest HD	JMC2016_07f	3	T	F
FA102	InterLig		balloon	MMFF94	Lowest MR	ACS_MCL2022_5b	1	F	F
FA103	fkcombu	Atom Rigid	RDKit	UFF	Lowest MR	ACS_MCL2022_5l	7	F	T
FA104	RDKit	Lowest RMSD	RDKit	MMFF94	Lowest HA	JMC2016_02	24	F	F
FA105	ShapeIt	Tversky	balloon	SFKEEM	Lowest HD	BMC2009_9a	11	F	F
FA106	Shaep	OnlyShape	openbabel	MMFF94/Weighted	Lowest HA	ACS_MCL2022_5f	16	F	F
FA107	fkcombu	Atom Flex	balloon	MMFF94	Highest HA	BMC2008_9f	21	F	F
FA108	RDKit	Tanimoto Distance	balloon	MMFF94	Lowest MR	ACS_MCL2022_5b	22	T	F
FA109	ShapeIt	Tanimoto	balloon	SFKEEM	Highest HA	BMC2008_9f	18	F	F
FA110	fkcombu	Atom Flex	openbabel	MMFF94/Weighted	Lowest MR	ACS_MCL2022_5l	7	F	F
FA111	AlignIt	Tversky	balloon	MMFF94	Lowest MR	ACS_MCL2022_5b	1	F	F
FA112	AlignIt	Tversky	openbabel	MMFF94/Weighted	Lowest HD	BMC2009_9a	1	T	F
FA113	ShapeIt	Tversky	balloon	SFKEEM	Most Rigid	BMC2009_9c	27	F	T
FA114	RDKit	Lowest RMSD	balloon	SFKEEM	Highest LogP	ACS_MCL2022_7k	27	F	F
FA115	fkcombu	Atom Flex	openbabel	MMFF94/Weighted	Lowest HD	BMC2009_9a	9	F	T
FA116	RDKit	Tanimoto Distance	balloon	SFKEEM	Most Rigid	BMC2009_9c	27	F	T

Table S22. List of alignments for the Full dataset

Align ID	Align Method	SF	Conf Search Method	FF	Ref.Par.	Ref.Mol.	Ref. Conf.	Use Long	Use Big
FA117	RDKit	Best Score	openbabel	MMFF94/Weighted	Lowest MR	ACS_MCL2022_5l	7	F	F
FA118	InterLig		balloon	SFKEEM	Lowest MR	ACS_MCL2022_5b	2	F	F
FA119	fkcombu	Atom Rigid	openbabel	MMFF94/Weighted	Longest	JMC2016_07c	14	F	T
FA120	InterLig		openbabel	MMFF94s/Random	Lowest HA	ACS_MCL2022_5d	27	T	F
FA121	ShapeIt	Tanimoto	openbabel	MMFF94/Weighted	Highest HA	BMC2008_9f	30	F	T
FA122	AlignIt	Tanimoto	openbabel	MMFF94s/Random	Most Rigid	BMC2009_9c	13	F	F
FA123	Shaep	OnlyShape	openbabel	MMFF94/Weighted	Highest HD	JMC2016_07f	13	F	T
FA124	fkcombu	Atom Rigid	openbabel	MMFF94s/Random	Most Rigid	BMC2009_9c	29	F	T
FA125	fkcombu	Volume Rigid	openbabel	MMFF94/Weighted	Lowest HA	JMC2016_02	19	T	F
FA126	Shaep	OnlyShape	openbabel	MMFF94s/Random	Most Flexible	ACS_MCL2022_7l	27	F	T
FA127	AlignIt	Tversky	openbabel	Ghemical/Random	Most Flexible	ACS_MCL2022_7l	25	T	F
FA128	RDKit	Lowest RMSD	openbabel	MMFF94/Weighted	Least Active	CMC2006_03c	5	F	F
FA129	fkcombu	Atom Rigid	openbabel	MMFF94s/Random	Lowest LogP	BMC2009_9f	11	F	T
FA130	RDKit	Best Score	openbabel	Ghemical/Random	Lowest MR	ACS_MCL2022_5l	26	T	F
FA131	ShapeIt	Tversky	openbabel	MMFF94/Weighted	Lowest LogP	BMC2009_9f	16	F	T
FA132	ShapeIt	Tanimoto	openbabel	MMFF94s/Random	Lowest LogP	BMC2009_9f	8	F	F
FA133	fkcombu	Atom Flex	openbabel	Ghemical/Random	Lowest LogP	BMC2009_9f	25	F	F
FA134	Shaep	Similarity	openbabel	MMFF94/Weighted	Least Active	CMC2006_03c	10	T	F
FA135	AlignIt	Tanimoto	openbabel	MMFF94s/Random	Least Active	CMC2006_03c	26	T	F
FA136	fkcombu	Volume Rigid	openbabel	Ghemical/Random	Least Active	CMC2006_03c	8	F	F
FA137	AlignIt	Tversky	openbabel	MMFF94s/Random	Least Active	CMC2006_03c	28	F	T
FA138	Shaep	OnlyShape	openbabel	MMFF94/Weighted	Lowest MR	ACS_MCL2022_5l	8	T	F
FA139	RDKit	Protrude Distance	openbabel	MMFF94s/Random	Highest HD	JMC2016_07f	19	F	T
FA140	RDKit	Best Score	openbabel	Ghemical/Random	Least Active	CMC2006_03c	8	F	F
FA141	fkcombu	Atom Flex	openbabel	MMFF94/Weighted	Most Active	BMC2010_12	7	F	F
FA142	fkcombu	Atom Rigid	openbabel	MMFF94s/Random	Lowest MR	ACS_MCL2022_5l	26	T	F
FA143	AlignIt	Tversky	openbabel	Ghemical/Random	Lowest HA	ACS_MCL2022_5l	26	F	T
FA144	RDKit	Best Score	openbabel	MMFF94/Weighted	Lowest MR	ACS_MCL2022_5l	8	T	F
FA145	RDKit	Protrude Distance	openbabel	MMFF94s/Random	Highest HD	JMC2016_07f	7	F	F
FA146	Shaep	OnlyShape	openbabel	Ghemical/Random	Least Active	CMC2006_03c	20	F	T
FA147	RDKit	Tanimoto Distance	openbabel	MMFF94/Weighted	Least Polar	BMC2010_26	23	T	F
FA148	fkcombu	Atom Flex	openbabel	MMFF94s/Random	Longest	JMC2016_07c	16	T	F
FA149	fkcombu	Volume Rigid	openbabel	Ghemical/Random	Lowest LogP	BMC2009_9f	21	F	T
FA150	Shaep	OnlyShape	openbabel	MMFF94/Weighted	Longest	ACS_MCL2022_7j	1	F	F
FA151	ShapeIt	Tversky	openbabel	Ghemical/Random	Highest HA	BMC2008_9f	28	F	T
FA152	Shaep	OnlyShape	openbabel	MMFF94s/Random	Highest HA	BMC2008_9f	2	F	F
FA153	Shaep	OnlyShape	openbabel	Ghemical/Random	Lowest HA	ACS_MCL2022_5l	26	F	T
FA154	fkcombu	Volume Rigid	openbabel	MMFF94/Weighted	Highest LogP	ACS_MCL2022_7m	8	T	F
FA155	RDKit	Protrude Distance	openbabel	MMFF94s/Random	Most Active	BMC2010_12	6	F	F
FA156	Shaep	Similarity	openbabel	Ghemical/Random	Most Active	BMC2010_12	11	F	F
FA157	fkcombu	Atom Rigid	openbabel	MMFF94s/Random	Highest HA	BMC2008_9f	12	T	F
FA158	InterLig		openbabel	MMFF94/Weighted	Most Rigid	BMC2009_9c	25	F	F
FA159	ShapeIt	Tversky	openbabel	Ghemical/Random	Lowest MR	ACS_MCL2022_5l	26	T	F
FA160	AlignIt	Tversky	openbabel	MMFF94s/Random	Most Active	BMC2010_12	6	F	F
FA161	ShapeIt	Tanimoto	openbabel	MMFF94/Weighted	Lowest MR	ACS_MCL2022_5l	29	F	T
FA162	fkcombu	Volume Rigid	openbabel	Ghemical/Random	Highest HD	JMC2016_07f	28	T	F
FA163	fkcombu	Atom Flex	openbabel	MMFF94s/Random	Lowest HA	ACS_MCL2022_5d	27	T	F
FA164	ShapeIt	Tversky	openbabel	MMFF94/Weighted	Lowest LogP	BMC2009_9f	1	T	F
FA165	ShapeIt	Tanimoto	openbabel	MMFF94s/Random	Longest	JMC2016_07c	16	T	F
FA166	fkcombu	Volume Rigid	openbabel	Ghemical/Random	Most Rigid	BMC2009_9c	23	F	F
FA167	RDKit	Best Score	openbabel	MMFF94s/Random	Highest HA	BMC2008_9f	23	F	T
FA168	InterLig		openbabel	Ghemical/Random	Most Active	BMC2010_12	24	F	T
FA169	RDKit	Best Score	openbabel	MMFF94s/Random	Lowest LogP	BMC2009_9f	11	F	T
FA170	fkcombu	Atom Flex	openbabel	Ghemical/Random	Lowest HD	BMC2009_9a	15	F	T
FA171	fkcombu	Volume Rigid	openbabel	MMFF94s/Random	Lowest HA	ACS_MCL2022_5d	29	F	T
FA172	ShapeIt	Tversky	openbabel	Ghemical/Random	Lowest HD	BMC2009_9a	15	F	F
FA173	RDKit	Best Score	openbabel	MMFF94s/Random	Lowest HD	BMC2009_9a	19	F	F
FA174	fkcombu	Volume Flex	openbabel	Ghemical/Random	Highest HA	BMC2008_9f	1	F	F

Table S22. List of alignments for the Full dataset

Align ID	Align Method	SF	Conf Search Method	FF	Ref.Par.	Ref.Mol.	Ref. Conf.	Use Long	Use Big
FA175	fkcombu	Volume Rigid	openbabel	MMFF94s/Random	Lowest HA	ACS_MCL2022_5d	27	T	F
FA176	fkcombu	Atom Flex	openbabel	Ghemical/Random	Highest LogP	ACS_MCL2022_7m	29	F	T
FA177	fkcombu	Volume Rigid	openbabel	MMFF94s/Random	Least Active	CMC2006_03c	28	F	T
FA178	fkcombu	Atom Flex	openbabel	Ghemical/Random	Least Polar	JMC2008_14	0	F	F
FA179	AlignIt	Tanimoto	openbabel	MMFF94s/Random	Most Active	BMC2010_12	6	F	F
FA180	ShapeIt	Tversky	openbabel	Ghemical/Random	Highest HD	JMC2016_07f	28	T	F
FA181	AlignIt	Tversky	openbabel	MMFF94s/Random	Highest LogP	ACS_MCL2022_7m	28	F	T
FA182	RDKit	Protrude Distance	openbabel	Ghemical/Random	Longest	JMC2016_07k	3	F	T
FA183	ShapeIt	Tversky	openbabel	MMFF94s/Random	Lowest MR	ACS_MCL2022_5l	1	F	F
FA184	InterLig		openbabel	Ghemical/Random	Most Active	BMC2010_12	20	T	F
FA185	ShapeIt	Tversky	openbabel	Ghemical/Random	Most Rigid	BMC2009_9c	23	F	F
FA186	fkcombu	Volume Rigid	openbabel	Ghemical/Random	Highest HD	JMC2016_07f	4	F	F
FA187	fkcombu	Atom Rigid	openbabel	Ghemical/Random	Highest HD	JMC2016_07f	28	T	F
FA188	InterLig		openbabel	UFF/Random	Lowest HD	BMC2009_9a	14	F	T
FA189	AlignIt	Tanimoto	openbabel	UFF/Random	Most Rigid	BMC2009_9c	25	T	F
FA190	fkcombu	Atom Flex	openbabel	UFF/Random	Lowest MR	ACS_MCL2022_5l	27	F	T
FA191	AlignIt	Tversky	openbabel	UFF/Random	Least Polar	BMC2010_26	20	T	F
FA192	ShapeIt	Tanimoto	openbabel	UFF/Random	Lowest MR	ACS_MCL2022_5l	2	F	F
FA193	Shaep	OnlyShape	openbabel	UFF/Random	Highest HA	BMC2008_9f	0	F	F
FA194	fkcombu	Volume Rigid	openbabel	UFF/Random	Least Polar	BMC2010_26	20	F	T
FA195	AlignIt	Tversky	openbabel	UFF/Random	Lowest LogP	BMC2009_9f	25	F	T
FA196	InterLig		openbabel	UFF/Random	Lowest HA	JMC2016_02	1	F	F
FA197	fkcombu	Atom Rigid	openbabel	UFF/Random	Lowest LogP	BMC2009_9f	20	F	F
FA198	fkcombu	Atom Flex	openbabel	UFF/Random	Most Rigid	BMC2009_9c	19	F	T
FA199	ShapeIt	Tversky	openbabel	UFF/Random	Lowest MR	ACS_MCL2022_5l	27	T	F
FA200	fkcombu	Volume Flex	openbabel	UFF/Random	Most Active	BMC2010_12	25	F	T
FA201	RDKit	Protrude Distance	openbabel	UFF/Random	Most Rigid	BMC2009_9c	19	F	T
FA202	AlignIt	Tanimoto	openbabel	UFF/Random	Highest HD	JMC2016_07f	0	F	F
FA203	ShapeIt	Tanimoto	openbabel	UFF/Random	Most Active	BMC2010_12	25	F	T
FA204	RDKit	Lowest RMSD	openbabel	UFF/Random	Least Polar	BMC2010_26	20	T	F
FA205	AlignIt	Tanimoto	openbabel	UFF/Random	Least Polar	BMC2010_29	0	F	F
FA206	ShapeIt	Tanimoto	openbabel	UFF/Random	Lowest HD	BMC2009_9a	12	F	F
FA207	AlignIt	Tanimoto	openbabel	UFF/Random	Highest HD	JMC2016_07f	24	T	F
FA208	AlignIt	Tanimoto	openbabel	UFF/Random	Highest LogP	ACS_MCL2022_7m	27	T	F
FA209	AlignIt	Tanimoto	openbabel	UFF/Random	Least Active	CMC2006_03c	0	F	F
FA210	AlignIt	Tversky	openbabel	UFF/Random	Highest HD	JMC2016_07f	0	F	F
FA211	RDKit	Tanimoto Distance	openbabel	UFF/Random	Highest HD	JMC2016_07f	0	F	F
FA212	RDKit	Best Score	openbabel	UFF/Random	Least Polar	BMC2010_29	0	F	F
FA213	RDKit	Lowest RMSD	openbabel	UFF/Random	Highest HA	BMC2008_9f	0	F	F
FA214	ShapeIt	Tversky	RDKit	MMFF94s	Most Flexible	ACS_MCL2022_7l	19	F	F
FA215	AlignIt	Tanimoto	RDKit	MMFF94s	Lowest HA	JMC2016_02	17	F	T
FA216	RDKit	Protrude Distance	RDKit	MMFF94s	Most Active	BMC2010_12	15	F	F
FA217	fkcombu	Atom Flex	RDKit	MMFF94s	Most Active	BMC2010_12	15	F	F
FA218	AlignIt	Tversky	RDKit	MMFF94s	Lowest HA	JMC2016_02	13	F	F
FA219	Shaep	Similarity	RDKit	MMFF94s	Least Active	CMC2006_03c	13	F	T
FA220	fkcombu	Atom Rigid	RDKit	MMFF94s	Most Rigid	BMC2009_9c	18	T	F
FA221	fkcombu	Volume Flex	RDKit	MMFF94s	Most Active	BMC2010_12	27	T	F
FA222	RDKit	Protrude Distance	RDKit	MMFF94s	Least Active	CMC2006_03c	4	F	F
FA223	fkcombu	Volume Flex	RDKit	MMFF94s	Lowest HD	BMC2009_9a	13	F	F
FA224	fkcombu	Volume Flex	RDKit	MMFF94s	Highest LogP	ACS_MCL2022_7m	20	F	F
FA225	fkcombu	Volume Rigid	RDKit	MMFF94s	Lowest MR	ACS_MCL2022_5l	10	T	F
FA226	RDKit	Protrude Distance	RDKit	MMFF94s	Lowest HD	BMC2009_9a	13	F	F
FA227	RDKit	Tanimoto Distance	RDKit	MMFF94s	Highest LogP	ACS_MCL2022_7m	20	F	F
FA228	fkcombu	Atom Flex	RDKit	MMFF94s	Lowest HA	JMC2016_02	13	F	F
FA229	ShapeIt	Tversky	RDKit	MMFF94s	Most Flexible	ACS_MCL2022_7l	3	F	T
FA230	fkcombu	Atom Flex	RDKit	MMFF94s	Highest HA	BMC2008_9f	1	T	F
FA231	AlignIt	Tanimoto	RDKit	MMFF94s	Lowest LogP	BMC2009_9f	17	F	T
FA232	Shaep	OnlyShape	RDKit	MMFF94s	Lowest LogP	BMC2009_9f	24	F	F

Table S22. List of alignments for the Full dataset

Align ID	Align Method	SF	Conf Search Method	FF	Ref.Par.	Ref.Mol.	Ref. Conf.	Use Long	Use Big
FA233	fkcombu	Volume Rigid	RDKit	MMFF94s	Most Flexible	ACS_MCL2022_7l	6	T	F
FA234	RDKit	Protrude Distance	RDKit	MMFF94s	Highest HD	JMC2016_07f	25	F	T
FA235	RDKit	Best Score	RDKit	MMFF94s	Highest LogP	ACS_MCL2022_7m	20	F	F
FA236	RDKit	Best Score	RDKit	MMFF94s	Highest HA	BMC2008_9f	28	F	T
FA237	RDKit	Tanimoto Distance	RDKit	MMFF94s	Lowest LogP	BMC2009_9f	11	T	F
FA238	RDKit	Protrude Distance	RDKit	MMFF94s	Most Rigid	BMC2009_9c	23	F	F
FA239	AlignIt	Tanimoto	RDKit	MMFF94s	Most Active	BMC2010_12	27	T	F
FA240	fkcombu	Volume Rigid	RDKit	MMFF94s	Most Rigid	BMC2009_9c	18	T	F
FA241	AlignIt	Tanimoto	openbabel	Ghemical/Weighted	Highest LogP	ACS_MCL2022_7m	23	T	F
FA242	fkcombu	Atom Rigid	openbabel	Ghemical/Weighted	Lowest HD	BMC2009_9a	2	F	T
FA243	InterLig		openbabel	Ghemical/Weighted	Lowest HD	BMC2009_9a	2	F	T
FA244	RDKit	Protrude Distance	openbabel	Ghemical/Weighted	Most Active	BMC2010_12	4	T	F
FA245	Shaep	OnlyShape	openbabel	Ghemical/Weighted	Highest HA	BMC2008_9f	11	F	T
FA246	AlignIt	Tanimoto	openbabel	Ghemical/Weighted	Most Rigid	BMC2009_9c	0	F	F
FA247	Shaep	OnlyShape	openbabel	Ghemical/Weighted	Lowest LogP	BMC2009_9f	29	F	T
FA248	RDKit	Tanimoto Distance	openbabel	Ghemical/Weighted	Highest HA	BMC2008_9f	6	F	F
FA249	fkcombu	Volume Flex	openbabel	Ghemical/Weighted	Most Rigid	BMC2009_9c	21	F	T
FA250	RDKit	Tanimoto Distance	openbabel	Ghemical/Weighted	Least Polar	JMC2008_04	2	F	F
FA251	fkcombu	Atom Flex	openbabel	Ghemical/Weighted	Lowest HA	JMC2016_02	19	F	T
FA252	RDKit	Protrude Distance	openbabel	Ghemical/Weighted	Highest HD	JMC2016_07f	22	F	T
FA253	RDKit	Protrude Distance	openbabel	Ghemical/Weighted	Highest HD	JMC2016_07f	22	T	F
FA254	ShapeIt	Tanimoto	openbabel	Ghemical/Weighted	Highest HA	BMC2008_9f	1	T	F
FA255	InterLig		openbabel	Ghemical/Weighted	Least Polar	BMC2010_26	29	F	T
FA256	RDKit	Best Score	openbabel	Ghemical/Weighted	Lowest MR	ACS_MCL2022_5l	25	F	T
FA257	fkcombu	Volume Flex	openbabel	Ghemical/Weighted	Lowest HD	BMC2009_9a	2	F	T
FA258	RDKit	Lowest RMSD	openbabel	Ghemical/Weighted	Least Polar	BMC2010_26	29	F	T
FA259	RDKit	Lowest RMSD	openbabel	Ghemical/Weighted	Least Active	CMC2006_03c	3	F	F
FA260	Shaep	OnlyShape	openbabel	Ghemical/Weighted	Longest	ACS_MCL2022_7k	1	F	F
FA261	InterLig		openbabel	Ghemical/Weighted	Lowest MR	ACS_MCL2022_5l	21	F	F
FA262	Shaep	Similarity	openbabel	Ghemical/Weighted	Lowest HA	JMC2016_02	20	T	F
FA263	Shaep	Similarity	openbabel	Ghemical/Weighted	Highest LogP	ACS_MCL2022_7m	23	T	F
FA264	AlignIt	Tversky	openbabel	Ghemical/Weighted	Least Polar	JMC2008_04	2	F	F
FA265	fkcombu	Atom Flex	openbabel	Ghemical/Weighted	Highest HD	JMC2016_07f	22	T	F
FA266	Shaep	Similarity	openbabel	Ghemical/Weighted	Least Active	CMC2006_03c	2	F	T
FA267	fkcombu	Atom Rigid	openbabel	Ghemical/Weighted	Highest HA	BMC2008_9f	1	T	F
FA268	ShapeIt	Tanimoto	openbabel	Ghemical/Weighted	Least Active	CMC2006_03c	5	T	F
FA269	ShapeIt	Tversky	openbabel	UFF/Weighted	Lowest HD	BMC2009_9a	25	F	F
FA270	RDKit	Lowest RMSD	openbabel	UFF/Weighted	Least Polar	JMC2008_14	3	F	F
FA271	fkcombu	Atom Flex	openbabel	UFF/Weighted	Lowest HD	BMC2009_9a	25	F	F
FA272	InterLig		openbabel	UFF/Weighted	Most Flexible	ACS_MCL2022_7l	2	T	F
FA273	fkcombu	Atom Flex	openbabel	UFF/Weighted	Most Rigid	BMC2009_9c	20	F	F
FA274	fkcombu	Atom Flex	openbabel	UFF/Weighted	Most Flexible	ACS_MCL2022_7l	1	F	F
FA275	ShapeIt	Tversky	openbabel	UFF/Weighted	Most Active	BMC2010_12	6	F	T
FA276	InterLig		openbabel	UFF/Weighted	Least Active	CMC2006_03c	21	F	T
FA277	ShapeIt	Tversky	openbabel	UFF/Weighted	Highest HA	BMC2008_9f	16	F	F
FA278	fkcombu	Atom Flex	openbabel	UFF/Weighted	Lowest HD	BMC2009_9a	30	T	F
FA279	RDKit	Best Score	openbabel	UFF/Weighted	Least Polar	JMC2008_14	3	F	F
FA280	fkcombu	Volume Rigid	openbabel	UFF/Weighted	Highest HA	BMC2008_9f	18	T	F
FA281	fkcombu	Volume Rigid	openbabel	UFF/Weighted	Lowest LogP	BMC2009_9f	30	F	T
FA282	fkcombu	Atom Rigid	openbabel	UFF/Weighted	Lowest MR	ACS_MCL2022_5l	30	T	F
FA283	RDKit	Best Score	openbabel	UFF/Weighted	Most Rigid	BMC2009_9c	1	T	F
FA284	AlignIt	Tanimoto	openbabel	UFF/Weighted	Least Polar	JMC2008_14	16	F	T
FA285	InterLig		openbabel	GAFF/Random	Most Rigid	BMC2009_9c	11	F	T
FA286	ShapeIt	Tanimoto	openbabel	UFF/Weighted	Lowest MR	ACS_MCL2022_5l	19	F	T
FA287	fkcombu	Atom Flex	openbabel	GAFF/Random	Highest LogP	ACS_MCL2022_7m	23	T	F
FA288	ShapeIt	Tanimoto	openbabel	UFF/Weighted	Most Flexible	ACS_MCL2022_7l	2	T	F
FA289	Shaep	OnlyShape	openbabel	GAFF/Random	Highest LogP	ACS_MCL2022_7m	2	F	T
FA290	ShapeIt	Tversky	openbabel	UFF/Weighted	Least Polar	BMC2010_27	30	T	F

Table S22. List of alignments for the Full dataset

Align ID	Align Method	SF	Conf Search Method	FF	Ref.Par.	Ref.Mol.	Ref. Conf.	Use Long	Use Big
FA291	AlignIt	Tanimoto	openbabel	GAFF/Random	Lowest MR	ACS_MCL2022_5l	0	F	F
FA292	fkcombu	Atom Rigid	openbabel	UFF/Weighted	Lowest HD	BMC2009_9a	8	F	T
FA293	RDKit	Tanimoto Distance	openbabel	GAFF/Random	Least Active	CMC2006_03c	0	F	F
FA294	RDKit	Best Score	openbabel	UFF/Weighted	Least Active	CMC2006_03c	21	F	T
FA295	Shaep	OnlyShape	openbabel	GAFF/Random	Most Rigid	BMC2009_9c	25	T	F
FA296	fkcombu	Volume Flex	openbabel	UFF/Weighted	Lowest LogP	BMC2009_9f	12	T	F
FA297	ShapeIt	Tversky	openbabel	GAFF/Random	Least Active	CMC2006_03c	0	F	F
FA298	fkcombu	Atom Flex	openbabel	UFF/Weighted	Most Active	BMC2010_12	14	T	F
FA299	fkcombu	Atom Flex	openbabel	GAFF/Random	Lowest LogP	BMC2009_9f	21	F	T
FA300	Shaep	Similarity	openbabel	UFF/Weighted	Lowest MR	ACS_MCL2022_5l	19	F	T
FA301	fkcombu	Volume Flex	openbabel	GAFF/Random	Lowest LogP	BMC2009_9f	19	T	F
FA302	RDKit	Protrude Distance	openbabel	UFF/Weighted	Most Active	BMC2010_12	14	T	F
FA303	Shaep	Similarity	openbabel	GAFF/Random	Most Flexible	ACS_MCL2022_7l	24	F	T
FA304	RDKit	Protrude Distance	openbabel	UFF/Weighted	Highest LogP	ACS_MCL2022_7m	6	F	F
FA305	InterLig		openbabel	GAFF/Random	Lowest HA	ACS_MCL2022_5d	27	T	F
FA306	AlignIt	Tanimoto	openbabel	GAFF/Random	Least Polar	BMC2010_26	27	T	F
FA307	RDKit	Best Score	openbabel	GAFF/Random	Highest HA	BMC2008_9f	5	T	F
FA308	AlignIt	Tanimoto	openbabel	GAFF/Random	Least Polar	JMC2008_14	8	F	F
FA309	fkcombu	Atom Rigid	openbabel	GAFF/Random	Most Rigid	BMC2009_9c	11	F	T
FA310	AlignIt	Tversky	openbabel	GAFF/Random	Most Active	BMC2010_12	21	F	T
FA311	fkcombu	Atom Rigid	openbabel	GAFF/Random	Lowest HA	ACS_MCL2022_5g	21	F	F
FA312	fkcombu	Volume Rigid	openbabel	GAFF/Random	Least Polar	BMC2010_26	27	T	F
FA313	RDKit	Best Score	openbabel	GAFF/Random	Longest	JMC2016_07c	10	T	F
FA314	fkcombu	Atom Rigid	openbabel	GAFF/Random	Lowest HD	BMC2009_9a	0	T	F
FA315	AlignIt	Tversky	openbabel	GAFF/Random	Most Rigid	BMC2009_9c	3	F	F
FA316	InterLig		openbabel	GAFF/Random	Most Active	BMC2010_12	21	F	T
FA317	fkcombu	Atom Rigid	openbabel	GAFF/Random	Most Active	BMC2010_12	18	T	F
FA318	Shaep	OnlyShape	openbabel	GAFF/Random	Highest HA	BMC2008_9f	10	F	T
FA319	fkcombu	Volume Flex	openbabel	GAFF/Random	Highest LogP	ACS_MCL2022_7m	2	F	T
FA320	InterLig		openbabel	GAFF/Random	Lowest HA	ACS_MCL2022_5g	21	F	F
FA321	ShapeIt	Tanimoto	openbabel	GAFF/Random	Highest HD	JMC2016_07f	5	F	F
FA322	ShapeIt	Tversky	openbabel	GAFF/Random	Lowest LogP	BMC2009_9f	9	F	F
FA323	Shaep	OnlyShape	openbabel	MMFF94/Random	Most Rigid	BMC2009_9c	1	F	F
FA324	fkcombu	Volume Flex	openbabel	MMFF94/Random	Most Active	BMC2010_12	25	T	F
FA325	RDKit	Tanimoto Distance	openbabel	MMFF94/Random	Lowest HD	BMC2009_9a	8	T	F
FA326	fkcombu	Atom Flex	openbabel	MMFF94/Random	Most Active	BMC2010_12	5	F	F
FA327	ShapeIt	Tversky	openbabel	MMFF94/Random	Lowest HD	BMC2009_9a	1	F	F
FA328	Shaep	Similarity	openbabel	MMFF94/Random	Least Active	CMC2006_03c	25	T	F
FA329	RDKit	Lowest RMSD	openbabel	MMFF94/Random	Highest HA	BMC2008_9f	10	F	F
FA330	fkcombu	Atom Flex	openbabel	MMFF94/Random	Lowest HD	BMC2009_9a	8	T	F
FA331	RDKit	Tanimoto Distance	openbabel	MMFF94/Random	Most Active	BMC2010_12	25	T	F
FA332	AlignIt	Tversky	openbabel	MMFF94/Random	Highest HA	BMC2008_9f	28	T	F
FA333	ShapeIt	Tanimoto	openbabel	MMFF94/Random	Most Rigid	BMC2009_9c	16	F	T
FA334	fkcombu	Atom Flex	openbabel	MMFF94/Random	Most Rigid	BMC2009_9c	16	F	T
FA335	fkcombu	Atom Rigid	openbabel	MMFF94/Random	Lowest HD	BMC2009_9a	5	F	T
FA336	RDKit	Best Score	openbabel	MMFF94/Random	Longest	JMC2016_07c	10	T	F
FA337	RDKit	Protrude Distance	openbabel	MMFF94/Random	Least Active	CMC2006_03c	25	T	F
FA338	AlignIt	Tanimoto	openbabel	MMFF94/Random	Lowest LogP	BMC2009_9f	15	T	F
FA339	AlignIt	Tanimoto	openbabel	MMFF94/Random	Lowest MR	ACS_MCL2022_5l	27	T	F
FA340	Shaep	Similarity	openbabel	MMFF94/Random	Least Polar	JMC2008_10	0	F	F
FA341	AlignIt	Tanimoto	openbabel	MMFF94/Random	Highest HA	BMC2008_9f	10	F	F
FA342	RDKit	Lowest RMSD	openbabel	MMFF94/Random	Least Polar	JMC2008_10	0	F	F
FA343	InterLig		openbabel	MMFF94/Random	Lowest HD	BMC2009_9a	5	F	T
FA344	Shaep	Similarity	openbabel	MMFF94/Random	Lowest LogP	BMC2009_9f	15	T	F
FA345	InterLig		openbabel	MMFF94/Random	Longest	JMC2016_07c	10	T	F
FA346	InterLig		openbabel	MMFF94/Random	Highest HD	JMC2016_07f	28	F	T
FA347	fkcombu	Volume Rigid	openbabel	MMFF94/Random	Lowest HD	BMC2009_9a	5	F	T
FA348	AlignIt	Tversky	openbabel	MMFF94/Random	Highest HA	BMC2008_9f	10	F	F

Table S22. List of alignments for the Full dataset

Align ID	Align Method	SF	Conf Search Method	FF	Ref.Par.	Ref.Mol.	Ref. Conf.	Use Long	Use Big
FA349	RDKit	Tanimoto Distance	openbabel	GAFF/Weighted	Lowest LogP	BMC2009_9f	7	F	F
FA350	ShapeIt	Tanimoto	openbabel	GAFF/Weighted	Most Flexible	ACS_MCL2022_7l	1	F	F
FA351	RDKit	Protrude Distance	openbabel	GAFF/Weighted	Lowest HA	JMC2016_02	15	F	T
FA352	InterLig		openbabel	GAFF/Weighted	Highest LogP	ACS_MCL2022_7m	13	F	T
FA353	Shaep	Similarity	openbabel	GAFF/Weighted	Least Polar	JMC2008_10	14	F	F
FA354	fkcombu	Volume Rigid	openbabel	GAFF/Weighted	Lowest HD	BMC2009_9a	30	T	F
FA355	AlignIt	Tversky	openbabel	GAFF/Weighted	Highest HA	BMC2008_9f	5	T	F
FA356	RDKit	Tanimoto Distance	openbabel	GAFF/Weighted	Lowest MR	ACS_MCL2022_5l	4	T	F
FA357	fkcombu	Volume Rigid	openbabel	GAFF/Weighted	Lowest LogP	BMC2009_9f	4	F	T
FA358	Shaep	OnlyShape	openbabel	GAFF/Weighted	Lowest LogP	BMC2009_9f	18	T	F
FA359	fkcombu	Atom Flex	openbabel	GAFF/Weighted	Highest HD	JMC2016_07f	13	T	F
FA360	fkcombu	Atom Rigid	openbabel	GAFF/Weighted	Least Active	CMC2006_03c	7	T	F
FA361	fkcombu	Volume Rigid	openbabel	GAFF/Weighted	Most Active	BMC2010_12	18	F	F
FA362	fkcombu	Volume Rigid	openbabel	GAFF/Weighted	Lowest MR	ACS_MCL2022_5l	4	T	F
FA363	RDKit	Lowest RMSD	openbabel	GAFF/Weighted	Least Active	CMC2006_03c	3	F	T
FA364	ShapeIt	Tversky	openbabel	GAFF/Weighted	Most Flexible	ACS_MCL2022_7l	1	F	F
FA365	AlignIt	Tversky	openbabel	GAFF/Weighted	Highest LogP	ACS_MCL2022_7m	22	T	F
FA366	InterLig		openbabel	GAFF/Weighted	Lowest HA	JMC2016_02	15	F	T
FA367	fkcombu	Atom Rigid	openbabel	GAFF/Weighted	Highest HA	BMC2008_9f	6	F	F
FA368	fkcombu	Atom Flex	openbabel	GAFF/Weighted	Most Active	BMC2010_12	5	F	T
FA369	fkcombu	Volume Flex	openbabel	GAFF/Weighted	Longest	JMC2016_07c	29	T	F
FA370	RDKit	Lowest RMSD	openbabel	GAFF/Weighted	Lowest HA	JMC2016_02	4	T	F
FA371	RDKit	Best Score	openbabel	GAFF/Weighted	Longest	JMC2016_07c	25	F	T
FA372	fkcombu	Volume Flex	openbabel	GAFF/Weighted	Lowest HA	JMC2016_02	4	T	F
FA373	InterLig		openbabel	GAFF/Weighted	Most Active	BMC2010_12	20	T	F
FA374	AlignIt	Tanimoto	openbabel	GAFF/Weighted	Most Rigid	BMC2009_9c	2	T	F
FA375	AlignIt	Tversky	openbabel	GAFF/Weighted	Highest LogP	ACS_MCL2022_7m	6	F	F
FA376	RDKit	Lowest RMSD	balloon	EEM	Highest HD	JMC2016_07c	27	F	F
FA377	Shaep	OnlyShape	balloon	EEM	Highest HD	JMC2016_07c	22	T	F
FA378	AlignIt	Tversky	balloon	EEM	Highest HD	JMC2016_07c	22	T	F
FA379	RDKit	Protrude Distance	balloon	EEM	Least Active	CMC2006_03c	27	F	T
FA380	ShapeIt	Tversky	balloon	EEM	Least Polar	JMC2008_14	7	T	F
FA381	RDKit	Lowest RMSD	balloon	EEM	Most Flexible	ACS_MCL2022_7l	17	F	T
FA382	fkcombu	Atom Rigid	balloon	EEM	Lowest LogP	BMC2009_9f	18	F	F
FA383	fkcombu	Atom Rigid	balloon	EEM	Lowest HD	BMC2009_9a	8	T	F
FA384	AlignIt	Tversky	balloon	EEM	Lowest LogP	BMC2009_9f	8	T	F
FA385	fkcombu	Volume Flex	balloon	EEM	Highest HD	JMC2016_07c	27	F	F
FA386	fkcombu	Atom Flex	balloon	EEM	Lowest HD	BMC2009_9a	20	F	T
FA387	RDKit	Best Score	balloon	EEM	Lowest LogP	BMC2009_9f	16	F	T
FA388	RDKit	Tanimoto Distance	balloon	EEM	Lowest HA	JMC2016_02	28	F	T
FA389	Shaep	OnlyShape	balloon	EEM	Lowest MR	ACS_MCL2022_5b	26	F	F
FA390	AlignIt	Tanimoto	balloon	EEM	Highest HA	BMC2008_9f	4	F	F
FA391	Shaep	Similarity	balloon	EEM	Least Active	CMC2006_03c	27	F	T
FA392	RDKit	Best Score	balloon	EEM	Least Active	CMC2006_03c	26	F	F
FA393	RDKit	Tanimoto Distance	balloon	EEM	Highest LogP	ACS_MCL2022_7k	22	F	F
FA394	fkcombu	Atom Rigid	balloon	EEM	Highest MR	JMC2016_07f	10	T	F
FA395	fkcombu	Volume Flex	balloon	EEM	Lowest HD	BMC2009_9a	1	F	F
FA396	fkcombu	Atom Flex	balloon	EEM	Lowest MR	ACS_MCL2022_5b	8	F	T
FA397	RDKit	Best Score	balloon	EEM	Highest HA	BMC2008_9f	4	F	F
FA398	RDKit	Best Score	balloon	EEM	Least Polar	BMC2010_27	0	F	T
FA399	fkcombu	Volume Flex	balloon	EEM	Most Active	BMC2010_12	6	F	F
FA400	fkcombu	Atom Rigid	balloon	EEM	Highest MR	JMC2016_07f	5	F	F
FA401	AlignIt	Tanimoto	balloon	EEM	Lowest HD	BMC2009_9a	8	T	F
FA402	RDKit	Protrude Distance	balloon	EEM	Most Rigid	BMC2009_9c	0	T	F
FA403	Shaep	OnlyShape	balloon	EEM	Most Flexible	ACS_MCL2022_7l	29	T	F
FA404	ShapeIt	Tanimoto	openbabel	MMFF94s/Weighted	Longest	JMC2016_07c	25	T	F
FA405	RDKit	Lowest RMSD	openbabel	MMFF94s/Weighted	Lowest LogP	BMC2009_9f	3	F	F
FA406	RDKit	Protrude Distance	openbabel	MMFF94s/Weighted	Highest HD	JMC2016_07f	25	F	T

Table S22. List of alignments for the Full dataset

Align ID	Align Method	SF	Conf Search Method	FF	Ref.Par.	Ref.Mol.	Ref. Conf.	Use Long	Use Big
FA407	RDKit	Tanimoto Distance	openbabel	MMFF94s/Weighted	Lowest MR	ACS_MCL2022_5l	21	F	T
FA408	ShapeIt	Tversky	openbabel	MMFF94s/Weighted	Most Flexible	ACS_MCL2022_7l	2	T	F
FA409	fkcombu	Atom Flex	openbabel	MMFF94s/Weighted	Least Active	CMC2006_03c	28	T	F
FA410	InterLig		openbabel	MMFF94s/Weighted	Lowest HD	BMC2009_9a	2	F	T
FA411	RDKit	Lowest RMSD	openbabel	MMFF94s/Weighted	Most Rigid	BMC2009_9c	26	F	F
FA412	RDKit	Lowest RMSD	openbabel	MMFF94s/Weighted	Highest HD	JMC2016_07f	25	F	T
FA413	Shaep	Similarity	openbabel	MMFF94s/Weighted	Lowest MR	ACS_MCL2022_5l	6	T	F
FA414	InterLig		openbabel	MMFF94s/Weighted	Highest LogP	ACS_MCL2022_7m	26	T	F
FA415	fkcombu	Volume Rigid	openbabel	MMFF94s/Weighted	Lowest HD	BMC2009_9a	2	F	T
FA416	AlignIt	Tanimoto	openbabel	MMFF94s/Weighted	Lowest MR	ACS_MCL2022_5l	21	F	T
FA417	ShapeIt	Tanimoto	openbabel	MMFF94s/Weighted	Highest HA	BMC2008_9f	21	T	F
FA418	AlignIt	Tanimoto	openbabel	MMFF94s/Weighted	Lowest HA	JMC2016_02	11	T	F
FA419	RDKit	Best Score	openbabel	MMFF94s/Weighted	Lowest HA	JMC2016_02	11	T	F
FA420	fkcombu	Atom Rigid	openbabel	MMFF94s/Weighted	Least Polar	BMC2010_27	11	F	T
FA421	fkcombu	Volume Flex	openbabel	MMFF94s/Weighted	Highest LogP	ACS_MCL2022_7m	26	T	F
FA422	ShapeIt	Tversky	openbabel	MMFF94s/Weighted	Most Rigid	BMC2009_9c	14	F	T
FA423	RDKit	Tanimoto Distance	openbabel	MMFF94s/Weighted	Highest HA	BMC2008_9f	6	F	F
FA424	AlignIt	Tversky	openbabel	MMFF94s/Weighted	Most Active	BMC2010_12	27	T	F
FA425	fkcombu	Atom Flex	openbabel	MMFF94s/Weighted	Lowest HA	JMC2016_02	11	T	F
FA426	RDKit	Best Score	openbabel	MMFF94s/Weighted	Least Polar	BMC2010_22	1	F	F
FA427	Shaep	OnlyShape	openbabel	MMFF94s/Weighted	Lowest HA	JMC2016_02	11	T	F
FA428	RDKit	Protrude Distance	openbabel	MMFF94s/Weighted	Highest HA	BMC2008_9f	21	T	F
FA429	AlignIt	Tanimoto	openbabel	MMFF94s/Weighted	Lowest HA	JMC2016_02	12	F	T
FA430	Shaep	Similarity	openbabel	MMFF94s/Weighted	Least Polar	BMC2010_26	24	T	F

Table S23. List of alignments for the Splitted dataset.

Align ID	Align Method	SF	Conf Search Method	FF	Ref.Par.	Ref.Mol.	Ref. Conf.	Use Long	Use Big
SA1	fkcombu	Volume Rigid	RDKit	MMFF94	Lowest MR	ACS_MCL2022_5b	22	T	F
SA2	RDKit	Lowest RMSD	balloon	SFKEEM	Highest HD	JMC2016_07f	12	T	F
SA3	ShapeIt	Tanimoto	RDKit	UFF	Highest LogP	ACS_MCL2022_7m	22	F	T
SA4	Shaep	Similarity	balloon	MMFF94	Most Active	BMC2010_12	17	F	T
SA5	ShapeIt	Tversky	RDKit	MMFF94s	Lowest LogP	BMC2009_9f	26	T	F
SA6	RDKit	Tanimoto Distance	openbabel	GAFF/Weighted	Lowest HD	BMC2009_9g	18	F	F
SA7	RDKit	Best Score	RDKit	MMFF94s	Lowest HD	BMC2009_9g	2	F	F
SA8	ShapeIt	Tversky	RDKit	UFF	Lowest HD	BMC2009_9g	1	F	T
SA9	fkcombu	Volume Rigid	balloon	SFKEEM	Lowest MR	ACS_MCL2022_5b	27	T	F
SA10	fkcombu	Atom Flex	RDKit	MMFF94	Lowest HA	ACS_MCL2022_5g	10	F	F
SA11	fkcombu	Atom Rigid	balloon	MMFF94	Highest MR	JMC2016_07f	9	T	F
SA12	fkcombu	Atom Flex	openbabel	GAFF/Weighted	Most Polar	CMC2006_11i	3	F	T
SA13	fkcombu	Atom Rigid	RDKit	MMFF94s	Most Polar	CMC2006_11i	8	F	T
SA14	RDKit	Lowest RMSD	balloon	SFKEEM	Least Polar	BMC2010_27	22	T	F
SA15	ShapeIt	Tversky	RDKit	MMFF94	Least Active	CMC2006_03c	19	F	T
SA16	ShapeIt	Tanimoto	RDKit	UFF	Lowest MR	ACS_MCL2022_5b	18	F	T
SA17	fkcombu	Volume Rigid	RDKit	MMFF94s	Lowest HD	BMC2009_9g	14	F	T
SA18	RDKit	Lowest RMSD	balloon	MMFF94	Most Polar	CMC2006_11i	21	F	T
SA19	RDKit	Protrude Distance	balloon	SFKEEM	Lowest MR	ACS_MCL2022_5b	27	T	F
SA20	RDKit	Best Score	RDKit	MMFF94	Highest HD	JMC2016_07f	12	T	F
SA21	fkcombu	Volume Rigid	RDKit	UFF	Lowest LogP	BMC2009_9f	14	F	T
SA22	Shaep	OnlyShape	RDKit	MMFF94s	Highest LogP	ACS_MCL2022_7m	19	F	F
SA23	AlignIt	Tanimoto	RDKit	UFF	Highest LogP	ACS_MCL2022_7m	13	F	F
SA24	fkcombu	Volume Rigid	openbabel	GAFF/Weighted	Least Active	CMC2006_03c	5	F	F
SA25	InterLig		balloon	SFKEEM	Lowest HA	ACS_MCL2022_5k	16	F	T
SA26	AlignIt	Tversky	RDKit	MMFF94	Most Polar	CMC2006_11i	25	F	F
SA27	fkcombu	Atom Rigid	RDKit	UFF	Most Flexible	ACS_MCL2022_7l	3	F	T
SA28	Shaep	OnlyShape	RDKit	MMFF94s	Least Polar	BMC2010_27	26	T	F
SA29	fkcombu	Volume Rigid	balloon	MMFF94	Lowest HD	CMC2006_03c	22	F	F
SA30	fkcombu	Volume Flex	RDKit	MMFF94	Lowest HD	BMC2009_9g	1	T	F
SA31	ShapeIt	Tversky	balloon	SFKEEM	Highest HA	BMC2009_9g	15	F	F
SA32	Shaep	Similarity	RDKit	UFF	Lowest MR	ACS_MCL2022_5b	18	F	T
SA33	ShapeIt	Tanimoto	RDKit	MMFF94s	Highest LogP	ACS_MCL2022_7m	19	F	F
SA34	fkcombu	Volume Rigid	openbabel	GAFF/Weighted	Highest HD	JMC2016_07f	22	F	F
SA35	Shaep	Similarity	RDKit	MMFF94	Lowest HA	ACS_MCL2022_5g	10	F	F
SA36	Shaep	OnlyShape	balloon	SFKEEM	Lowest MR	ACS_MCL2022_5b	27	T	F
SA37	RDKit	Best Score	RDKit	MMFF94s	Lowest HA	ACS_MCL2022_5d	10	F	F
SA38	ShapeIt	Tanimoto	RDKit	UFF	Least Active	CMC2006_03c	22	T	F
SA39	Shaep	Similarity	RDKit	MMFF94	Lowest MR	ACS_MCL2022_5b	13	F	F
SA40	fkcombu	Atom Flex	balloon	MMFF94	Most Polar	CMC2006_11i	1	T	F
SA41	Shaep	OnlyShape	balloon	SFKEEM	Highest HA	BMC2009_9g	15	F	F
SA42	RDKit	Tanimoto Distance	RDKit	MMFF94s	Lowest HD	BMC2009_9g	2	F	F
SA43	RDKit	Tanimoto Distance	RDKit	UFF	Lowest MR	ACS_MCL2022_5b	27	F	F
SA44	RDKit	Tanimoto Distance	RDKit	MMFF94	Highest LogP	ACS_MCL2022_7m	23	T	F
SA45	RDKit	Protrude Distance	openbabel	GAFF/Weighted	Highest LogP	ACS_MCL2022_7m	16	F	F
SA46	RDKit	Protrude Distance	RDKit	UFF	Most Rigid	BMC2009_9c	20	F	F
SA47	RDKit	Best Score	RDKit	MMFF94s	Most Flexible	ACS_MCL2022_7l	29	F	F
SA48	fkcombu	Atom Flex	balloon	SFKEEM	Lowest HD	CMC2006_05b	19	F	F
SA49	fkcombu	Volume Rigid	balloon	MMFF94	Lowest HA	ACS_MCL2022_5d	24	T	F
SA50	RDKit	Lowest RMSD	RDKit	MMFF94	Highest LogP	ACS_MCL2022_7m	23	T	F
SA51	ShapeIt	Tanimoto	RDKit	MMFF94s	Lowest HA	ACS_MCL2022_5d	22	T	F
SA52	fkcombu	Atom Flex	RDKit	UFF	Most Polar	CMC2006_11i	15	T	F
SA53	fkcombu	Volume Flex	openbabel	GAFF/Weighted	Least Polar	BMC2010_27	9	F	T
SA54	RDKit	Protrude Distance	balloon	SFKEEM	Least Polar	BMC2010_27	22	T	F
SA55	InterLig		RDKit	MMFF94	Lowest HA	ACS_MCL2022_5g	10	F	F
SA56	RDKit	Lowest RMSD	openbabel	UFF/Random	Lowest LogP	BMC2009_9f	9	F	F
SA57	Shaep	OnlyShape	balloon	MMFF94	Lowest HA	ACS_MCL2022_5d	24	T	F
SA58	ShapeIt	Tanimoto	RDKit	UFF	Lowest MR	ACS_MCL2022_5b	27	F	F

Table S23. List of alignments for the Splitted dataset.

Align ID	Align Method	SF	Conf Search Method	FF	Ref.Par.	Ref.Mol.	Ref. Conf.	Use Long	Use Big
SA59	fkcombu	Atom Rigid	RDKit	MMFF94s	Least Active	CMC2006_03c	11	F	F
SA60	fkcombu	Atom Flex	RDKit	MMFF94	Least Polar	BMC2010_27	22	T	F
SA61	RDKit	Protrude Distance	balloon	SFKEEM	Lowest HA	ACS_MCL2022_5k	16	F	T
SA62	RDKit	Lowest RMSD	openbabel	UFF/Random	Most Rigid	BMC2009_9c	6	F	T
SA63	AlignIt	Tanimoto	openbabel	GAFF/Weighted	Highest HD	JMC2016_07f	21	F	T
SA64	ShapeIt	Tanimoto	RDKit	MMFF94s	Most Polar	CMC2006_11i	8	F	T
SA65	fkcombu	Volume Rigid	RDKit	UFF	Most Active	BMC2010_12	1	T	F
SA66	fkcombu	Volume Rigid	RDKit	MMFF94	Most Polar	CMC2006_11i	25	F	F
SA67	RDKit	Best Score	balloon	MMFF94	Most Rigid	BMC2009_9c	13	T	F
SA68	AlignIt	Tversky	balloon	SFKEEM	Lowest HD	BMC2009_9g	5	F	T
SA69	RDKit	Lowest RMSD	openbabel	UFF/Random	Most Polar	CMC2006_11i	29	F	T
SA70	Shaep	Similarity	RDKit	MMFF94s	Lowest HD	BMC2009_9g	8	T	F
SA71	ShapeIt	Tanimoto	openbabel	GAFF/Weighted	Lowest HD	BMC2009_9g	1	T	F
SA72	fkcombu	Volume Flex	RDKit	UFF	Most Rigid	BMC2009_9c	20	F	F
SA73	AlignIt	Tanimoto	balloon	MMFF94	Lowest LogP	BMC2009_9f	28	T	F
SA74	fkcombu	Atom Flex	RDKit	MMFF94	Most Polar	CMC2006_11i	19	T	F
SA75	RDKit	Lowest RMSD	openbabel	UFF/Random	Most Active	BMC2010_12	22	F	T
SA76	Shaep	Similarity	balloon	SFKEEM	Lowest MR	ACS_MCL2022_5b	29	F	F
SA77	fkcombu	Volume Rigid	RDKit	MMFF94s	Least Polar	JMC2008_10	27	F	T
SA78	fkcombu	Volume Flex	RDKit	MMFF94	Highest HD	JMC2016_07f	12	T	F
SA79	RDKit	Best Score	RDKit	UFF	Lowest LogP	BMC2009_9f	14	F	T
SA80	ShapeIt	Tversky	RDKit	MMFF94s	Most Polar	CMC2006_11i	2	T	F
SA81	RDKit	Protrude Distance	openbabel	GAFF/Weighted	Least Polar	BMC2010_27	9	F	T
SA82	RDKit	Protrude Distance	openbabel	UFF/Random	Lowest MR	ACS_MCL2022_5b	2	F	F
SA83	ShapeIt	Tversky	RDKit	MMFF94	Most Polar	CMC2006_11i	9	F	T
SA84	fkcombu	Atom Rigid	balloon	SFKEEM	Lowest MR	ACS_MCL2022_5b	29	F	F
SA85	RDKit	Lowest RMSD	RDKit	UFF	Lowest LogP	BMC2009_9f	24	T	F
SA86	fkcombu	Atom Flex	balloon	MMFF94	Least Active	CMC2006_03c	7	F	T
SA87	fkcombu	Volume Flex	RDKit	MMFF94	Most Active	BMC2010_12	23	F	F
SA88	fkcombu	Atom Rigid	openbabel	GAFF/Weighted	Lowest MR	ACS_MCL2022_5b	18	T	F
SA89	Shaep	OnlyShape	openbabel	UFF/Random	Highest HA	BMC2009_9g	29	T	F
SA90	RDKit	Best Score	RDKit	MMFF94s	Lowest MR	ACS_MCL2022_5b	6	T	F
SA91	RDKit	Protrude Distance	openbabel	GAFF/Weighted	Lowest HA	ACS_MCL2022_5f	8	F	F
SA92	Shaep	Similarity	balloon	SFKEEM	Highest LogP	ACS_MCL2022_7m	18	T	F
SA93	RDKit	Protrude Distance	RDKit	UFF	Most Flexible	ACS_MCL2022_7l	3	F	T
SA94	AlignIt	Tanimoto	openbabel	UFF/Random	Lowest MR	ACS_MCL2022_5b	28	F	T
SA95	RDKit	Lowest RMSD	balloon	MMFF94	Lowest HA	ACS_MCL2022_5d	28	F	T
SA96	RDKit	Tanimoto Distance	RDKit	MMFF94	Highest HD	JMC2016_07f	22	F	T
SA97	fkcombu	Atom Rigid	balloon	SFKEEM	Highest HD	JMC2016_07i	27	F	T
SA98	fkcombu	Volume Rigid	RDKit	UFF	Highest LogP	ACS_MCL2022_7m	2	T	F
SA99	RDKit	Tanimoto Distance	openbabel	UFF/Random	Least Active	CMC2006_03c	11	F	F
SA100	RDKit	Best Score	RDKit	MMFF94s	Least Active	CMC2006_03c	10	F	T
SA101	fkcombu	Volume Rigid	openbabel	GAFF/Weighted	Lowest HA	ACS_MCL2022_5d	26	T	F
SA102	fkcombu	Atom Rigid	balloon	MMFF94	Highest LogP	ACS_MCL2022_7m	16	T	F
SA103	RDKit	Best Score	RDKit	UFF	Highest LogP	ACS_MCL2022_7m	13	F	F
SA104	AlignIt	Tanimoto	RDKit	MMFF94	Least Polar	JMC2008_10	18	F	F
SA105	ShapeIt	Tversky	openbabel	UFF/Random	Highest LogP	ACS_MCL2022_7m	27	T	F
SA106	AlignIt	Tanimoto	openbabel	GAFF/Weighted	Lowest LogP	BMC2009_9f	2	F	T
SA107	RDKit	Protrude Distance	balloon	SFKEEM	Lowest HD	CMC2006_05b	28	T	F
SA108	fkcombu	Atom Rigid	RDKit	MMFF94s	Lowest HD	BMC2009_9g	8	T	F
SA109	fkcombu	Volume Flex	balloon	MMFF94	Lowest LogP	BMC2009_9f	2	F	T
SA110	Shaep	OnlyShape	RDKit	UFF	Least Polar	BMC2010_24	26	F	F
SA111	Shaep	OnlyShape	RDKit	MMFF94	Highest LogP	ACS_MCL2022_7m	16	F	F
SA112	fkcombu	Volume Flex	openbabel	UFF/Random	Highest LogP	ACS_MCL2022_7m	27	F	T
SA113	AlignIt	Tversky	RDKit	MMFF94s	Lowest LogP	BMC2009_9f	12	F	F
SA114	ShapeIt	Tversky	balloon	SFKEEM	Least Active	CMC2006_03c	14	F	T
SA115	Shaep	Similarity	openbabel	GAFF/Weighted	Most Flexible	ACS_MCL2022_7l	4	T	F
SA116	AlignIt	Tversky	RDKit	UFF	Highest HD	JMC2016_07f	22	F	F

Table S23. List of alignments for the Splitted dataset.

Align ID	Align Method	SF	Conf Search Method	FF	Ref.Par.	Ref.Mol.	Ref. Conf.	Use Long	Use Big
SA117	ShapeIt	Tversky	RDKit	MMFF94	Least Active	CMC2006_03c	21	F	F
SA118	InterLig		openbabel	UFF/Random	Most Polar	CMC2006_11i	28	T	F
SA119	ShapeIt	Tanimoto	balloon	MMFF94	Most Polar	CMC2006_11i	1	F	F
SA120	ShapeIt	Tversky	balloon	SFKEEM	Most Flexible	ACS_MCL2022_7l	26	F	T
SA121	Shaep	OnlyShape	openbabel	GAFF/Weighted	Lowest HD	BMC2009_9g	1	T	F
SA122	RDKit	Best Score	RDKit	MMFF94s	Lowest HA	ACS_MCL2022_5d	22	T	F
SA123	Shaep	OnlyShape	openbabel	UFF/Random	Lowest HA	ACS_MCL2022_5f	3	F	F
SA124	fkcombu	Volume Rigid	balloon	MMFF94	Lowest HD	CMC2006_05b	26	F	T
SA125	ShapeIt	Tversky	balloon	SFKEEM	Highest LogP	ACS_MCL2022_7m	27	F	F
SA126	RDKit	Protrude Distance	RDKit	UFF	Lowest HD	BMC2009_9g	25	F	F
SA127	Shaep	OnlyShape	RDKit	MMFF94	Least Polar	BMC2010_27	22	T	F
SA128	RDKit	Lowest RMSD	balloon	MMFF94	Lowest HD	CMC2006_03c	23	T	F
SA129	ShapeIt	Tanimoto	openbabel	GAFF/Weighted	Lowest LogP	BMC2009_9f	2	F	F
SA130	Shaep	Similarity	balloon	SFKEEM	Lowest LogP	BMC2009_9f	29	F	F
SA131	InterLig		openbabel	UFF/Random	Least Polar	BMC2010_27	18	T	F
SA132	ShapeIt	Tversky	RDKit	MMFF94s	Most Active	BMC2010_12	14	F	F
SA133	Shaep	Similarity	RDKit	UFF	Most Flexible	ACS_MCL2022_7l	0	T	F
SA134	fkcombu	Atom Flex	openbabel	GAFF/Weighted	Highest HD	JMC2016_07f	22	F	F
SA135	RDKit	Lowest RMSD	balloon	SFKEEM	Lowest MR	ACS_MCL2022_5b	10	F	T
SA136	fkcombu	Atom Rigid	openbabel	UFF/Random	Least Polar	JMC2008_15	3	F	F
SA137	Shaep	Similarity	balloon	MMFF94	Most Active	BMC2010_12	13	T	F
SA138	AlignIt	Tanimoto	RDKit	MMFF94	Most Flexible	ACS_MCL2022_7l	15	F	T
SA139	fkcombu	Volume Rigid	RDKit	UFF	Lowest HA	ACS_MCL2022_5d	27	F	F
SA140	RDKit	Lowest RMSD	openbabel	GAFF/Weighted	Least Active	CMC2006_03c	5	F	F
SA141	fkcombu	Atom Flex	balloon	MMFF94	Most Rigid	BMC2009_9c	13	F	F
SA142	Shaep	OnlyShape	balloon	SFKEEM	Lowest HA	ACS_MCL2022_5k	16	F	T
SA143	RDKit	Best Score	openbabel	UFF/Random	Lowest MR	ACS_MCL2022_5b	28	T	F
SA144	fkcombu	Atom Rigid	RDKit	MMFF94s	Lowest MR	ACS_MCL2022_5b	6	T	F
SA145	ShapeIt	Tversky	RDKit	MMFF94	Lowest LogP	BMC2009_9f	27	F	T
SA146	RDKit	Tanimoto Distance	RDKit	UFF	Lowest HD	BMC2009_9g	1	F	T
SA147	InterLig		openbabel	GAFF/Weighted	Most Flexible	ACS_MCL2022_7l	10	F	T
SA148	AlignIt	Tversky	balloon	MMFF94	Most Flexible	ACS_MCL2022_7l	29	T	F
SA149	Shaep	OnlyShape	balloon	SFKEEM	Most Active	BMC2010_12	25	F	T
SA150	InterLig		openbabel	UFF/Random	Most Rigid	BMC2009_9c	8	T	F
SA151	Shaep	OnlyShape	balloon	MMFF94	Highest MR	JMC2016_07f	9	T	F
SA152	Shaep	Similarity	RDKit	MMFF94	Most Flexible	ACS_MCL2022_7l	15	F	T
SA153	AlignIt	Tversky	RDKit	MMFF94s	Lowest HD	BMC2009_9g	14	F	T
SA154	Shaep	OnlyShape	balloon	SFKEEM	Least Active	CMC2006_03c	11	T	F
SA155	RDKit	Tanimoto Distance	RDKit	UFF	Highest HD	JMC2016_07f	22	F	F
SA156	RDKit	Protrude Distance	openbabel	GAFF/Weighted	Most Rigid	BMC2009_9c	25	T	F
SA157	Shaep	OnlyShape	openbabel	UFF/Random	Least Active	CMC2006_03c	20	F	T
SA158	fkcombu	Volume Flex	balloon	MMFF94	Highest LogP	ACS_MCL2022_7m	0	F	F
SA159	AlignIt	Tversky	RDKit	MMFF94	Most Active	BMC2010_12	23	T	F
SA160	ShapeIt	Tversky	balloon	SFKEEM	Lowest HD	CMC2006_05b	19	F	F
SA161	RDKit	Lowest RMSD	RDKit	MMFF94s	Lowest MR	ACS_MCL2022_5b	11	F	F
SA162	fkcombu	Atom Rigid	RDKit	UFF	Most Active	BMC2010_12	1	T	F
SA163	RDKit	Protrude Distance	openbabel	GAFF/Weighted	Lowest LogP	BMC2009_9f	2	F	F
SA164	ShapeIt	Tanimoto	balloon	MMFF94	Lowest MR	ACS_MCL2022_5b	29	T	F
SA165	RDKit	Tanimoto Distance	openbabel	UFF/Random	Most Flexible	ACS_MCL2022_7l	29	T	F
SA166	RDKit	Best Score	balloon	SFKEEM	Most Active	BMC2010_12	20	F	F
SA167	AlignIt	Tanimoto	RDKit	MMFF94	Least Active	CMC2006_03c	10	T	F
SA168	AlignIt	Tanimoto	openbabel	GAFF/Weighted	Least Polar	BMC2010_27	9	F	T
SA169	ShapeIt	Tanimoto	RDKit	MMFF94s	Lowest LogP	BMC2009_9f	12	F	F
SA170	fkcombu	Volume Rigid	openbabel	Ghemical/Random	Lowest HD	BMC2009_9g	27	F	T
SA171	Shaep	Similarity	balloon	MMFF94	Lowest HA	ACS_MCL2022_5d	24	T	F
SA172	fkcombu	Atom Flex	balloon	SFKEEM	Highest MR	JMC2016_07f	14	F	T
SA173	RDKit	Tanimoto Distance	openbabel	GAFF/Weighted	Highest LogP	ACS_MCL2022_7m	10	F	T
SA174	InterLig		openbabel	UFF/Random	Most Rigid	BMC2009_9c	1	F	F

Table S23. List of alignments for the Splitted dataset.

Align ID	Align Method	SF	Conf Search Method	FF	Ref.Par.	Ref.Mol.	Ref. Conf.	Use Long	Use Big
SA175	ShapeIt	Tanimoto	RDKit	MMFF94s	Most Flexible	ACS_MCL2022_7l	4	T	F
SA176	Shaep	Similarity	balloon	SFKEEM	Most Rigid	BMC2009_9c	1	F	T
SA177	ShapeIt	Tversky	balloon	MMFF94	Highest HD	JMC2016_07a	19	T	F
SA178	RDKit	Protrude Distance	openbabel	Ghemical/Random	Most Flexible	ACS_MCL2022_7l	27	F	T
SA179	fkcombu	Volume Flex	balloon	EEM	Most Polar	CMC2006_11i	20	F	T
SA180	AlignIt	Tanimoto	openbabel	UFF/Random	Least Active	CMC2006_03c	20	F	T
SA181	RDKit	Tanimoto Distance	openbabel	GAFF/Weighted	Lowest LogP	BMC2009_9f	23	T	F
SA182	RDKit	Lowest RMSD	balloon	SFKEEM	Most Active	BMC2010_12	20	F	F
SA183	AlignIt	Tversky	openbabel	Ghemical/Random	Highest LogP	ACS_MCL2022_7m	27	T	F
SA184	AlignIt	Tversky	balloon	EEM	Highest LogP	ACS_MCL2022_7m	7	F	T
SA185	InterLig		balloon	MMFF94	Highest MR	JMC2016_07f	21	F	T
SA186	RDKit	Lowest RMSD	openbabel	Ghemical/Random	Most Rigid	BMC2009_9c	27	F	T
SA187	ShapeIt	Tversky	openbabel	GAFF/Weighted	Highest HD	JMC2016_07f	2	T	F
SA188	Shaep	OnlyShape	openbabel	UFF/Random	Highest LogP	ACS_MCL2022_7m	27	F	T
SA189	RDKit	Tanimoto Distance	balloon	MMFF94	Least Polar	BMC2010_28	1	F	F
SA190	fkcombu	Atom Flex	balloon	EEM	Lowest HD	CMC2006_03c	26	F	F
SA191	fkcombu	Volume Rigid	openbabel	GAFF/Weighted	Least Polar	BMC2010_12	14	F	F
SA192	fkcombu	Atom Rigid	openbabel	Ghemical/Random	Most Active	BMC2010_12	18	F	T
SA193	RDKit	Tanimoto Distance	openbabel	UFF/Random	Longest	JMC2016_07b	22	F	T
SA194	ShapeIt	Tanimoto	balloon	EEM	Most Flexible	ACS_MCL2022_7l	20	F	T
SA195	AlignIt	Tversky	balloon	MMFF94	Highest MR	JMC2016_07f	9	T	F
SA196	RDKit	Protrude Distance	openbabel	Ghemical/Random	Most Rigid	BMC2009_9c	7	T	F
SA197	fkcombu	Atom Rigid	balloon	EEM	Most Polar	CMC2006_11i	20	F	T
SA198	RDKit	Lowest RMSD	balloon	MMFF94	Highest HD	JMC2016_07k	27	F	F
SA199	fkcombu	Atom Rigid	openbabel	UFF/Random	Highest LogP	ACS_MCL2022_7m	27	F	T
SA200	AlignIt	Tversky	balloon	EEM	Lowest MR	ACS_MCL2022_5b	24	F	F
SA201	RDKit	Best Score	openbabel	Ghemical/Random	Most Flexible	ACS_MCL2022_7l	4	F	F
SA202	RDKit	Lowest RMSD	balloon	MMFF94	Highest LogP	ACS_MCL2022_7m	16	T	F
SA203	ShapeIt	Tanimoto	balloon	EEM	Highest HA	BMC2009_9g	24	T	F
SA204	fkcombu	Atom Flex	openbabel	UFF/Random	Lowest MR	ACS_MCL2022_5b	2	F	F
SA205	ShapeIt	Tanimoto	openbabel	Ghemical/Random	Lowest HD	BMC2009_9g	15	T	F
SA206	AlignIt	Tversky	balloon	MMFF94	Most Rigid	BMC2009_9c	13	F	F
SA207	Shaep	OnlyShape	balloon	EEM	Most Active	BMC2010_12	22	F	T
SA208	RDKit	Protrude Distance	openbabel	Ghemical/Random	Lowest HA	ACS_MCL2022_5k	27	T	F
SA209	fkcombu	Volume Flex	openbabel	UFF/Random	Lowest MR	ACS_MCL2022_5b	28	T	F
SA210	RDKit	Lowest RMSD	balloon	EEM	Lowest MR	ACS_MCL2022_5b	17	F	T
SA211	InterLig		openbabel	UFF/Random	Lowest LogP	BMC2009_9f	12	T	F
SA212	RDKit	Best Score	balloon	EEM	Least Polar	JMC2008_10	8	F	F
SA213	RDKit	Tanimoto Distance	openbabel	Ghemical/Random	Lowest MR	ACS_MCL2022_5b	27	T	F
SA214	Shaep	OnlyShape	openbabel	MMFF94s/Random	Lowest HD	CMC2006_03c	10	F	F
SA215	AlignIt	Tanimoto	balloon	EEM	Highest HA	BMC2009_9g	29	F	T
SA216	fkcombu	Volume Rigid	openbabel	UFF/Random	Lowest HD	BMC2009_9g	29	F	T
SA217	fkcombu	Atom Rigid	openbabel	Ghemical/Random	Most Polar	CMC2006_11i	0	F	F
SA218	Shaep	OnlyShape	balloon	EEM	Most Flexible	ACS_MCL2022_7l	16	T	F
SA219	Shaep	Similarity	openbabel	MMFF94s/Random	Highest LogP	ACS_MCL2022_7m	0	F	F
SA220	ShapeIt	Tversky	openbabel	UFF/Random	Most Active	BMC2010_12	22	F	T
SA221	RDKit	Tanimoto Distance	openbabel	Ghemical/Random	Lowest HD	BMC2009_9g	27	F	T
SA222	fkcombu	Volume Flex	balloon	EEM	Highest LogP	ACS_MCL2022_7m	18	T	F
SA223	RDKit	Protrude Distance	openbabel	MMFF94s/Random	Most Rigid	BMC2009_9c	13	F	F
SA224	fkcombu	Atom Flex	openbabel	Ghemical/Random	Least Active	CMC2006_03c	20	F	T
SA225	fkcombu	Atom Flex	openbabel	MMFF94s/Random	Highest HA	BMC2009_9g	13	T	F
SA226	RDKit	Lowest RMSD	balloon	EEM	Highest LogP	ACS_MCL2022_7m	8	F	F
SA227	RDKit	Protrude Distance	balloon	EEM	Lowest MR	ACS_MCL2022_5b	24	F	F
SA228	RDKit	Lowest RMSD	openbabel	MMFF94s/Random	Highest LogP	ACS_MCL2022_7m	0	F	F
SA229	ShapeIt	Tanimoto	openbabel	Ghemical/Random	Most Flexible	ACS_MCL2022_7l	26	T	F
SA230	RDKit	Protrude Distance	openbabel	MMFF94s/Random	Most Rigid	BMC2009_9c	28	F	T
SA231	fkcombu	Atom Rigid	openbabel	MMFF94s/Random	Lowest MR	ACS_MCL2022_5b	26	F	T
SA232	AlignIt	Tversky	openbabel	Ghemical/Random	Lowest HD	BMC2009_9g	15	T	F

Table S23. List of alignments for the Splitted dataset.

Align ID	Align Method	SF	Conf Search Method	FF	Ref.Par.	Ref.Mol.	Ref. Conf.	Use Long	Use Big
SA233	RDKit	Best Score	balloon	EEM	Lowest LogP	BMC2009_9f	19	T	F
SA234	Shaep	OnlyShape	openbabel	MMFF94s/Random	Most Rigid	BMC2009_9c	13	F	F
SA235	RDKit	Protrude Distance	openbabel	Ghemical/Random	Most Polar	CMC2006_11i	27	F	T
SA236	RDKit	Tanimoto Distance	balloon	EEM	Highest HA	BMC2009_9g	14	F	F
SA237	ShapeIt	Tanimoto	openbabel	Ghemical/Random	Most Rigid	BMC2009_9c	7	T	F
SA238	ShapeIt	Tversky	balloon	EEM	Highest MR	JMC2016_07f	1	F	F
SA239	AlignIt	Tversky	openbabel	MMFF94s/Random	Lowest LogP	BMC2009_9f	1	F	T
SA240	RDKit	Protrude Distance	openbabel	Ghemical/Random	Most Flexible	ACS_MCL2022_7l	26	T	F
SA241	ShapeIt	Tversky	balloon	EEM	Most Flexible	ACS_MCL2022_7l	18	F	F
SA242	ShapeIt	Tversky	openbabel	Ghemical/Random	Lowest LogP	BMC2009_9f	25	F	T
SA243	AlignIt	Tversky	openbabel	MMFF94s/Random	Most Active	BMC2010_12	5	F	F
SA244	ShapeIt	Tanimoto	balloon	EEM	Lowest MR	ACS_MCL2022_5b	20	T	F
SA245	fkcombu	Volume Rigid	openbabel	Ghemical/Random	Least Active	CMC2006_03c	20	F	T
SA246	RDKit	Tanimoto Distance	balloon	EEM	Most Rigid	BMC2009_9c	1	T	F
SA247	ShapeIt	Tanimoto	openbabel	MMFF94s/Random	Highest LogP	ACS_MCL2022_7m	27	F	T
SA248	AlignIt	Tversky	openbabel	Ghemical/Random	Least Active	CMC2006_03c	8	F	F
SA249	ShapeIt	Tversky	balloon	EEM	Most Flexible	ACS_MCL2022_7l	20	F	T
SA250	InterLig		openbabel	Ghemical/Random	Most Rigid	BMC2009_9c	7	F	F
SA251	ShapeIt	Tversky	balloon	EEM	Highest HD	JMC2016_07a	18	F	F
SA252	ShapeIt	Tversky	openbabel	MMFF94s/Random	Most Polar	CMC2006_11i	27	F	T
SA253	ShapeIt	Tanimoto	balloon	EEM	Most Rigid	BMC2009_9c	29	F	T
SA254	InterLig		openbabel	Ghemical/Random	Lowest LogP	BMC2009_9f	25	F	T
SA255	AlignIt	Tanimoto	openbabel	MMFF94s/Random	Most Active	BMC2010_12	5	F	F
SA256	fkcombu	Volume Rigid	balloon	EEM	Least Polar	JMC2008_10	8	F	F
SA257	RDKit	Best Score	openbabel	Ghemical/Random	Most Polar	CMC2006_11i	27	F	T
SA258	fkcombu	Volume Rigid	openbabel	MMFF94s/Random	Least Polar	BMC2010_24	1	F	F
SA259	Shaep	Similarity	balloon	EEM	Lowest HD	CMC2006_03c	27	F	T
SA260	RDKit	Lowest RMSD	openbabel	Ghemical/Random	Lowest HA	ACS_MCL2022_5k	27	T	F
SA261	fkcombu	Atom Rigid	balloon	EEM	Lowest HA	ACS_MCL2022_5k	11	F	T
SA262	RDKit	Protrude Distance	openbabel	Ghemical/Random	Lowest HA	ACS_MCL2022_5f	29	F	T
SA263	Shaep	OnlyShape	openbabel	MMFF94s/Random	Most Rigid	BMC2009_9c	28	F	T
SA264	RDKit	Tanimoto Distance	openbabel	Ghemical/Random	Least Active	CMC2006_03c	22	T	F
SA265	fkcombu	Atom Rigid	openbabel	MMFF94s/Random	Most Active	BMC2010_12	29	F	T
SA266	InterLig		openbabel	Ghemical/Weighted	Least Polar	JMC2008_04	9	F	T
SA267	Shaep	OnlyShape	openbabel	MMFF94s/Random	Most Rigid	BMC2009_9c	23	T	F
SA268	InterLig		openbabel	Ghemical/Weighted	Lowest HA	ACS_MCL2022_5d	24	F	T
SA269	InterLig		openbabel	Ghemical/Weighted	Lowest HA	ACS_MCL2022_5d	24	T	F
SA270	RDKit	Tanimoto Distance	openbabel	MMFF94s/Random	Highest HD	JMC2016_07f	21	T	F
SA271	RDKit	Lowest RMSD	openbabel	MMFF94s/Random	Lowest LogP	BMC2009_9f	28	F	F
SA272	ShapeIt	Tanimoto	openbabel	MMFF94s/Random	Most Flexible	ACS_MCL2022_7l	28	T	F
SA273	Shaep	OnlyShape	openbabel	Ghemical/Weighted	Lowest LogP	BMC2009_9f	19	F	T
SA274	AlignIt	Tversky	openbabel	MMFF94s/Random	Lowest HA	ACS_MCL2022_5k	2	F	F
SA275	AlignIt	Tanimoto	openbabel	Ghemical/Weighted	Most Active	BMC2010_12	24	F	T
SA276	Shaep	Similarity	openbabel	MMFF94s/Random	Least Polar	BMC2010_27	29	T	F
SA277	fkcombu	Volume Flex	openbabel	MMFF94s/Random	Highest LogP	ACS_MCL2022_7m	27	T	F
SA278	fkcombu	Volume Flex	openbabel	MMFF94s/Random	Highest HA	BMC2009_9g	13	T	F
SA279	Shaep	OnlyShape	openbabel	MMFF94s/Random	Most Flexible	ACS_MCL2022_7l	26	F	T
SA280	Shaep	Similarity	openbabel	Ghemical/Weighted	Least Polar	JMC2008_04	9	F	T
SA281	RDKit	Tanimoto Distance	openbabel	MMFF94s/Random	Most Polar	CMC2006_11i	27	F	T
SA282	fkcombu	Atom Rigid	openbabel	MMFF94s/Random	Most Flexible	ACS_MCL2022_7l	26	F	T
SA283	AlignIt	Tversky	openbabel	Ghemical/Weighted	Most Polar	CMC2006_11i	21	T	F
SA284	RDKit	Lowest RMSD	openbabel	Ghemical/Weighted	Most Polar	CMC2006_11i	27	F	T
SA285	fkcombu	Volume Flex	openbabel	Ghemical/Weighted	Lowest HA	ACS_MCL2022_5d	24	F	T
SA286	RDKit	Lowest RMSD	openbabel	Ghemical/Weighted	Highest HD	JMC2016_07f	1	T	F
SA287	Shaep	Similarity	openbabel	Ghemical/Weighted	Lowest HA	ACS_MCL2022_5d	24	T	F
SA288	Shaep	Similarity	openbabel	Ghemical/Weighted	Highest LogP	ACS_MCL2022_7m	22	F	T
SA289	fkcombu	Volume Rigid	openbabel	Ghemical/Weighted	Highest HD	JMC2016_07f	10	F	T
SA290	ShapeIt	Tanimoto	openbabel	Ghemical/Weighted	Most Rigid	BMC2009_9c	20	F	T

Table S23. List of alignments for the Splitted dataset.

Align ID	Align Method	SF	Conf Search Method	FF	Ref.Par.	Ref.Mol.	Ref. Conf.	Use Long	Use Big
SA291	fkcombu	Atom Flex	openbabel	Ghemical/Weighted	Least Polar	JMC2008_04	9	F	T
SA292	InterLig		openbabel	Ghemical/Weighted	Most Rigid	BMC2009_9c	22	T	F
SA293	InterLig		openbabel	Ghemical/Weighted	Most Flexible	ACS_MCL2022_7l	22	F	T
SA294	InterLig		openbabel	Ghemical/Weighted	Lowest HD	BMC2009_9g	24	T	F
SA295	RDKit	Tanimoto Distance	openbabel	Ghemical/Weighted	Lowest LogP	BMC2009_9f	19	F	T
SA296	fkcombu	Volume Flex	openbabel	GAFF/Random	Most Polar	CMC2006_11i	23	F	T
SA297	fkcombu	Atom Flex	openbabel	Ghemical/Weighted	Highest LogP	ACS_MCL2022_7m	16	T	F
SA298	AlignIt	Tversky	openbabel	GAFF/Random	Lowest MR	ACS_MCL2022_5b	3	F	F
SA299	Shaep	OnlyShape	openbabel	GAFF/Random	Least Active	CMC2006_03c	23	T	F
SA300	ShapeIt	Tanimoto	openbabel	GAFF/Random	Lowest LogP	BMC2009_9f	11	F	F
SA301	fkcombu	Volume Rigid	openbabel	GAFF/Random	Lowest HA	ACS_MCL2022_5f	1	F	F
SA302	InterLig		openbabel	GAFF/Random	Most Rigid	BMC2009_9c	19	F	F
SA303	ShapeIt	Tversky	openbabel	GAFF/Random	Highest HD	JMC2016_07f	19	F	F
SA304	fkcombu	Atom Rigid	openbabel	GAFF/Random	Most Rigid	BMC2009_9c	13	T	F
SA305	Shaep	Similarity	openbabel	GAFF/Random	Lowest LogP	BMC2009_9f	3	T	F
SA306	RDKit	Best Score	openbabel	GAFF/Random	Highest LogP	ACS_MCL2022_7m	17	F	F
SA307	fkcombu	Atom Rigid	openbabel	GAFF/Random	Most Active	BMC2010_12	18	T	F
SA308	fkcombu	Atom Flex	openbabel	GAFF/Random	Least Polar	BMC2010_27	9	T	F
SA309	Shaep	OnlyShape	openbabel	GAFF/Random	Highest HD	JMC2016_07f	19	F	F
SA310	ShapeIt	Tversky	openbabel	GAFF/Random	Lowest LogP	BMC2009_9f	3	T	F
SA311	AlignIt	Tversky	openbabel	GAFF/Random	Highest LogP	ACS_MCL2022_7m	8	T	F
SA312	AlignIt	Tversky	openbabel	GAFF/Random	Highest LogP	ACS_MCL2022_7m	17	F	T
SA313	fkcombu	Atom Flex	openbabel	GAFF/Random	Lowest HD	BMC2009_9g	16	F	F
SA314	ShapeIt	Tversky	openbabel	GAFF/Random	Least Polar	BMC2010_29	29	F	T
SA315	AlignIt	Tversky	openbabel	GAFF/Random	Lowest LogP	BMC2009_9f	22	F	T
SA316	ShapeIt	Tanimoto	openbabel	GAFF/Random	Least Active	CMC2006_03c	23	T	F
SA317	RDKit	Protrude Distance	openbabel	GAFF/Random	Least Active	CMC2006_03c	9	F	F
SA318	Shaep	Similarity	openbabel	GAFF/Random	Lowest LogP	BMC2009_9f	11	F	F
SA319	RDKit	Tanimoto Distance	openbabel	GAFF/Random	Highest HD	JMC2016_07f	19	F	F
SA320	ShapeIt	Tversky	openbabel	GAFF/Random	Lowest MR	ACS_MCL2022_5b	3	F	F
SA321	Shaep	OnlyShape	openbabel	GAFF/Random	Least Polar	JMC2008_15	8	F	F
SA322	fkcombu	Atom Rigid	openbabel	GAFF/Random	Least Polar	BMC2010_29	29	F	T
SA323	fkcombu	Volume Rigid	openbabel	GAFF/Random	Lowest HD	BMC2009_9g	16	F	F
SA324	fkcombu	Volume Flex	openbabel	MMFF94s/Weighted	Lowest HA	ACS_MCL2022_5d	0	F	F
SA325	Shaep	Similarity	openbabel	MMFF94s/Weighted	Most Polar	CMC2006_11i	22	F	T
SA326	AlignIt	Tanimoto	openbabel	MMFF94s/Weighted	Lowest HD	CMC2006_05b	2	F	T
SA327	Shaep	OnlyShape	openbabel	MMFF94s/Weighted	Highest LogP	ACS_MCL2022_7m	4	F	F
SA328	fkcombu	Atom Rigid	openbabel	MMFF94s/Weighted	Lowest HA	ACS_MCL2022_5d	15	T	F
SA329	ShapeIt	Tversky	openbabel	MMFF94s/Weighted	Lowest LogP	BMC2009_9f	9	F	T
SA330	ShapeIt	Tversky	openbabel	MMFF94s/Weighted	Most Active	BMC2010_12	10	F	F
SA331	RDKit	Best Score	openbabel	MMFF94s/Weighted	Lowest HA	ACS_MCL2022_5d	27	F	T
SA332	RDKit	Lowest RMSD	openbabel	MMFF94s/Weighted	Most Polar	CMC2006_11i	22	F	T
SA333	fkcombu	Atom Rigid	openbabel	MMFF94s/Weighted	Most Active	BMC2010_12	7	F	T
SA334	RDKit	Best Score	openbabel	MMFF94s/Weighted	Most Flexible	ACS_MCL2022_7l	13	F	F
SA335	Shaep	Similarity	openbabel	MMFF94s/Weighted	Highest LogP	ACS_MCL2022_7m	20	F	T
SA336	fkcombu	Atom Rigid	openbabel	MMFF94s/Weighted	Highest HA	BMC2009_9g	21	F	F
SA337	RDKit	Lowest RMSD	openbabel	MMFF94s/Weighted	Most Flexible	ACS_MCL2022_7l	13	F	F
SA338	RDKit	Protrude Distance	openbabel	MMFF94s/Weighted	Lowest LogP	BMC2009_9f	9	F	T
SA339	RDKit	Lowest RMSD	openbabel	MMFF94s/Weighted	Highest HD	JMC2016_07f	21	F	T
SA340	Shaep	Similarity	openbabel	MMFF94s/Weighted	Lowest HD	CMC2006_05b	2	F	T
SA341	InterLig		openbabel	MMFF94s/Weighted	Lowest HA	ACS_MCL2022_5d	27	F	T
SA342	ShapeIt	Tversky	openbabel	MMFF94s/Weighted	Lowest HA	ACS_MCL2022_5d	0	F	F
SA343	ShapeIt	Tanimoto	openbabel	MMFF94s/Weighted	Most Flexible	ACS_MCL2022_7l	13	F	F
SA344	fkcombu	Volume Rigid	openbabel	MMFF94s/Weighted	Least Active	CMC2006_03c	1	F	F
SA345	fkcombu	Atom Flex	openbabel	MMFF94s/Weighted	Longest	JMC2016_07k	5	F	F
SA346	InterLig		openbabel	MMFF94s/Weighted	Lowest HD	CMC2006_05b	9	T	F
SA347	fkcombu	Atom Flex	openbabel	MMFF94s/Weighted	Lowest MR	ACS_MCL2022_5b	25	F	T
SA348	ShapeIt	Tanimoto	openbabel	MMFF94s/Weighted	Most Active	BMC2010_12	26	T	F

Table S23. List of alignments for the Splitted dataset.

Align ID	Align Method	SF	Conf Search Method	FF	Ref.Par.	Ref.Mol.	Ref. Conf.	Use Long	Use Big
SA349	ShapeIt	Tversky	openbabel	MMFF94s/Weighted	Longest	JMC2016_07k	5	F	F
SA350	ShapeIt	Tversky	openbabel	MMFF94s/Weighted	Lowest HA	ACS_MCL2022_5d	15	T	F
SA351	Shaep	OnlyShape	openbabel	MMFF94s/Weighted	Least Polar	BMC2010_27	3	F	F
SA352	RDKit	Best Score	openbabel	MMFF94s/Weighted	Lowest HD	CMC2006_05b	2	F	T
SA353	fkcombu	Volume Rigid	openbabel	MMFF94s/Weighted	Most Polar	CMC2006_11i	4	F	F
SA354	fkcombu	Atom Flex	openbabel	MMFF94/Weighted	Most Active	BMC2010_12	3	F	F
SA355	fkcombu	Volume Rigid	openbabel	MMFF94/Weighted	Lowest HD	CMC2006_03c	2	F	T
SA356	InterLig		openbabel	MMFF94/Weighted	Highest HD	JMC2016_07f	5	T	F
SA357	Shaep	Similarity	openbabel	MMFF94/Weighted	Lowest LogP	BMC2009_9f	23	F	T
SA358	RDKit	Best Score	openbabel	MMFF94/Weighted	Most Polar	CMC2006_11i	10	F	T
SA359	AlignIt	Tversky	openbabel	MMFF94/Weighted	Lowest MR	ACS_MCL2022_5b	19	T	F
SA360	fkcombu	Volume Flex	openbabel	MMFF94/Weighted	Lowest HA	ACS_MCL2022_5d	30	F	T
SA361	AlignIt	Tversky	openbabel	MMFF94/Weighted	Lowest LogP	BMC2009_9f	23	F	T
SA362	RDKit	Best Score	openbabel	MMFF94/Weighted	Lowest MR	ACS_MCL2022_5b	29	F	T
SA363	fkcombu	Atom Flex	openbabel	MMFF94/Weighted	Most Active	BMC2010_12	15	T	F
SA364	InterLig		openbabel	MMFF94/Weighted	Lowest HA	ACS_MCL2022_5d	29	T	F
SA365	ShapeIt	Tanimoto	openbabel	MMFF94/Weighted	Most Polar	CMC2006_11i	13	F	F
SA366	fkcombu	Atom Rigid	openbabel	MMFF94/Weighted	Most Active	BMC2010_12	3	F	F
SA367	RDKit	Best Score	openbabel	MMFF94/Weighted	Highest HD	JMC2016_07f	12	F	T
SA368	AlignIt	Tversky	openbabel	MMFF94/Weighted	Least Polar	BMC2010_12	24	F	T
SA369	Shaep	Similarity	openbabel	MMFF94/Weighted	Highest LogP	ACS_MCL2022_7m	1	F	F
SA370	ShapeIt	Tanimoto	openbabel	MMFF94/Weighted	Highest HD	JMC2016_07f	12	F	T
SA371	fkcombu	Atom Rigid	openbabel	MMFF94/Weighted	Least Active	CMC2006_03c	17	T	F
SA372	Shaep	OnlyShape	openbabel	MMFF94/Weighted	Highest LogP	ACS_MCL2022_7m	27	T	F
SA373	fkcombu	Atom Rigid	openbabel	MMFF94/Weighted	Lowest HA	ACS_MCL2022_5d	29	T	F
SA374	ShapeIt	Tanimoto	openbabel	MMFF94/Weighted	Lowest MR	ACS_MCL2022_5b	19	T	F
SA375	Shaep	Similarity	openbabel	MMFF94/Weighted	Lowest LogP	BMC2009_9f	7	F	F
SA376	ShapeIt	Tanimoto	openbabel	MMFF94/Weighted	Lowest LogP	BMC2009_9f	23	F	T
SA377	ShapeIt	Tversky	openbabel	MMFF94/Weighted	Lowest HD	CMC2006_03c	2	F	T
SA378	AlignIt	Tanimoto	openbabel	MMFF94/Weighted	Highest LogP	ACS_MCL2022_7m	1	F	T
SA379	AlignIt	Tanimoto	openbabel	MMFF94/Weighted	Lowest HA	ACS_MCL2022_5d	30	F	T
SA380	InterLig		openbabel	MMFF94/Weighted	Lowest LogP	BMC2009_9f	2	T	F
SA381	AlignIt	Tanimoto	openbabel	MMFF94/Random	Highest HD	JMC2016_07f	27	F	T
SA382	ShapeIt	Tversky	openbabel	MMFF94/Random	Highest HD	JMC2016_07f	6	F	F
SA383	Shaep	OnlyShape	openbabel	UFF/Weighted	Most Flexible	ACS_MCL2022_7l	10	T	F
SA384	RDKit	Protrude Distance	openbabel	MMFF94/Random	Least Active	CMC2006_03c	0	F	F
SA385	RDKit	Tanimoto Distance	openbabel	UFF/Weighted	Lowest MR	ACS_MCL2022_5b	11	F	F
SA386	RDKit	Protrude Distance	openbabel	MMFF94/Random	Most Polar	CMC2006_11i	29	T	F
SA387	fkcombu	Volume Rigid	openbabel	UFF/Weighted	Most Active	BMC2010_12	25	F	F
SA388	RDKit	Best Score	openbabel	MMFF94/Random	Highest LogP	ACS_MCL2022_7m	0	F	F
SA389	AlignIt	Tversky	openbabel	UFF/Weighted	Most Rigid	BMC2009_9c	1	T	F
SA390	RDKit	Protrude Distance	openbabel	MMFF94/Random	Most Rigid	BMC2009_9c	24	F	T
SA391	fkcombu	Atom Flex	openbabel	UFF/Weighted	Most Polar	CMC2006_11i	11	F	T
SA392	Shaep	OnlyShape	openbabel	MMFF94/Random	Lowest HA	ACS_MCL2022_5g	27	F	T
SA393	InterLig		openbabel	UFF/Weighted	Most Flexible	ACS_MCL2022_7l	10	T	F
SA394	fkcombu	Atom Rigid	openbabel	MMFF94/Random	Least Polar	BMC2010_25	0	F	F
SA395	RDKit	Lowest RMSD	openbabel	MMFF94/Random	Lowest HA	ACS_MCL2022_5g	27	F	T
SA396	fkcombu	Atom Flex	openbabel	UFF/Weighted	Lowest HA	ACS_MCL2022_5d	9	T	F
SA397	ShapeIt	Tanimoto	openbabel	MMFF94/Random	Most Flexible	ACS_MCL2022_7l	24	F	T
SA398	AlignIt	Tanimoto	openbabel	UFF/Weighted	Lowest HA	ACS_MCL2022_5d	23	F	F
SA399	fkcombu	Atom Flex	openbabel	MMFF94/Random	Lowest HD	CMC2006_03b	6	F	F
SA400	Shaep	Similarity	openbabel	UFF/Weighted	Most Rigid	BMC2009_9c	3	F	T
SA401	ShapeIt	Tversky	openbabel	MMFF94/Random	Lowest HA	ACS_MCL2022_5g	27	F	T
SA402	fkcombu	Volume Rigid	openbabel	UFF/Weighted	Lowest LogP	BMC2009_9f	5	T	F
SA403	RDKit	Tanimoto Distance	openbabel	MMFF94/Random	Most Flexible	ACS_MCL2022_7l	27	T	F
SA404	RDKit	Tanimoto Distance	openbabel	UFF/Weighted	Highest LogP	ACS_MCL2022_7m	25	F	T
SA405	AlignIt	Tanimoto	openbabel	MMFF94/Random	Most Flexible	ACS_MCL2022_7l	27	T	F
SA406	AlignIt	Tanimoto	openbabel	UFF/Weighted	Lowest HD	BMC2009_9g	8	T	F

Table S23. List of alignments for the Splitted dataset.

Align ID	Align Method	SF	Conf Search Method	FF	Ref.Par.	Ref.Mol.	Ref. Conf.	Use Long	Use Big
SA407	fkcombu	Atom Flex	openbabel	MMFF94/Random	Most Flexible	ACS_MCL2022_7l	24	F	T
SA408	RDKit	Lowest RMSD	openbabel	UFF/Weighted	Lowest HD	CMC2006_05b	8	F	F
SA409	ShapeIt	Tanimoto	openbabel	MMFF94/Random	Most Polar	CMC2006_11i	29	T	F
SA410	RDKit	Tanimoto Distance	openbabel	MMFF94/Random	Lowest MR	ACS_MCL2022_5b	27	F	T
SA411	ShapeIt	Tanimoto	openbabel	UFF/Weighted	Highest HD	JMC2016_07f	18	F	T
SA412	fkcombu	Atom Flex	openbabel	MMFF94/Random	Lowest LogP	BMC2009_9f	8	T	F
SA413	fkcombu	Volume Flex	openbabel	UFF/Weighted	Least Active	CMC2006_03c	16	F	T
SA414	ShapeIt	Tversky	openbabel	MMFF94/Random	Highest HD	JMC2016_07f	18	T	F
SA415	fkcombu	Atom Rigid	openbabel	UFF/Weighted	Lowest MR	ACS_MCL2022_5b	25	F	T
SA416	Shaep	OnlyShape	openbabel	MMFF94/Random	Highest LogP	ACS_MCL2022_7m	0	F	F
SA417	RDKit	Lowest RMSD	openbabel	UFF/Weighted	Most Active	BMC2010_12	25	F	F
SA418	fkcombu	Atom Flex	openbabel	MMFF94/Random	Lowest HA	ACS_MCL2022_5k	26	T	F
SA419	RDKit	Best Score	openbabel	UFF/Weighted	Most Flexible	ACS_MCL2022_7l	9	F	T
SA420	RDKit	Lowest RMSD	openbabel	MMFF94/Random	Most Active	BMC2010_12	27	F	T
SA421	InterLig		openbabel	UFF/Weighted	Least Polar	BMC2010_27	1	F	F
SA422	RDKit	Best Score	openbabel	MMFF94/Random	Most Active	BMC2010_12	27	F	T
SA423	Shaep	Similarity	openbabel	UFF/Weighted	Highest LogP	ACS_MCL2022_7m	4	F	F
SA424	fkcombu	Atom Flex	openbabel	MMFF94/Random	Most Flexible	ACS_MCL2022_7l	5	F	F
SA425	fkcombu	Atom Flex	openbabel	UFF/Weighted	Highest LogP	ACS_MCL2022_7m	25	F	T
SA426	Shaep	Similarity	openbabel	MMFF94/Random	Lowest HD	CMC2006_03b	6	F	F
SA427	fkcombu	Atom Flex	openbabel	UFF/Weighted	Lowest HA	ACS_MCL2022_5d	10	F	T
SA428	fkcombu	Volume Flex	openbabel	MMFF94/Random	Most Active	BMC2010_12	23	T	F
SA429	RDKit	Tanimoto Distance	openbabel	MMFF94/Random	Lowest LogP	BMC2009_9f	16	F	F
SA430	RDKit	Lowest RMSD	openbabel	UFF/Weighted	Most Flexible	ACS_MCL2022_7l	9	F	T
SA431	AlignIt	Tanimoto	openbabel	MMFF94/Random	Highest HA	BMC2009_9g	28	F	F
SA432	fkcombu	Volume Flex	openbabel	UFF/Weighted	Lowest LogP	BMC2009_9f	1	F	T
SA433	Shaep	Similarity	openbabel	UFF/Weighted	Most Flexible	ACS_MCL2022_7l	10	T	F
SA434	AlignIt	Tanimoto	openbabel	UFF/Weighted	Least Active	CMC2006_03c	20	T	F
SA435	RDKit	Tanimoto Distance	openbabel	UFF/Weighted	Most Active	BMC2010_12	23	F	T

Table S24. 3-D QSAR models coefficients obtained with the Full dataset.

**Model ID**	$\mathbf{r^2_{cv}}$	$\mathbf{q^2_{cv}}$	$\mathbf{ONPC_{cv}}$
$M_{3\text{-}D\_QSAR\_Full\_}1$	0.834	0.698	4
$M_{3\text{-}D\_QSAR\_Full\_}2$	0.855	0.751	4
$M_{3\text{-}D\_QSAR\_Full\_}3$	0.852	0.726	4
$M_{3\text{-}D\_QSAR\_Full\_}4$	0.966	0.639	8
$M_{3\text{-}D\_QSAR\_Full\_}5$	0.974	0.712	8
$M_{3\text{-}D\_QSAR\_Full\_}6$	0.918	0.804	5
$M_{3\text{-}D\_QSAR\_Full\_}7$	0.864	0.708	4
$M_{3\text{-}D\_QSAR\_Full\_}8$	0.768	0.704	3
$M_{3\text{-}D\_QSAR\_Full\_}9$	0.96	0.775	8
$M_{3\text{-}D\_QSAR\_Full\_}10$	0.807	0.607	4
$M_{3\text{-}D\_QSAR\_Full\_}11$	0.749	0.656	3
$M_{3\text{-}D\_QSAR\_Full\_}12$	0.769	0.705	3
$M_{3\text{-}D\_QSAR\_Full\_}13$	0.843	0.654	4
$M_{3\text{-}D\_QSAR\_Full\_}14$	0.834	0.698	4
$M_{3\text{-}D\_QSAR\_Full\_}15$	0.908	0.715	6
$M_{3\text{-}D\_QSAR\_Full\_}16$	0.951	0.728	7
$M_{3\text{-}D\_QSAR\_Full\_}17$	0.96	0.743	8
$M_{3\text{-}D\_QSAR\_Full\_}18$	0.778	0.637	3
$M_{3\text{-}D\_QSAR\_Full\_}19$	0.9	0.745	5
$M_{3\text{-}D\_QSAR\_Full\_}20$	0.913	0.75	5
$M_{3\text{-}D\_QSAR\_Full\_}21$	0.893	0.759	5
$M_{3\text{-}D\_QSAR\_Full\_}22$	0.957	0.737	8
$M_{3\text{-}D\_QSAR\_Full\_}23$	0.924	0.704	7
$M_{3\text{-}D\_QSAR\_Full\_}24$	0.757	0.715	2
$M_{3\text{-}D\_QSAR\_Full\_}25$	0.738	0.703	2
$M_{3\text{-}D\_QSAR\_Full\_}26$	0.874	0.746	4
$M_{3\text{-}D\_QSAR\_Full\_}27$	0.75	0.659	3
$M_{3\text{-}D\_QSAR\_Full\_}28$	0.859	0.707	5
$M_{3\text{-}D\_QSAR\_Full\_}29$	0.82	0.737	3
$M_{3\text{-}D\_QSAR\_Full\_}30$	0.807	0.724	3
$M_{3\text{-}D\_QSAR\_Full\_}31$	0.768	0.704	3
$M_{3\text{-}D\_QSAR\_Full\_}32$	0.908	0.715	6
$M_{3\text{-}D\_QSAR\_Full\_}33$	0.843	0.654	4
$M_{3\text{-}D\_QSAR\_Full\_}34$	0.933	0.669	6
$M_{3\text{-}D\_QSAR\_Full\_}35$	0.927	0.731	6
$M_{3\text{-}D\_QSAR\_Full\_}36$	0.788	0.694	3
$M_{3\text{-}D\_QSAR\_Full\_}37$	0.945	0.806	6
$M_{3\text{-}D\_QSAR\_Full\_}38$	0.951	0.786	6
$M_{3\text{-}D\_QSAR\_Full\_}39$	0.898	0.746	5
$M_{3\text{-}D\_QSAR\_Full\_}40$	0.891	0.681	5
$M_{3\text{-}D\_QSAR\_Full\_}41$	0.843	0.719	3
$M_{3\text{-}D\_QSAR\_Full\_}42$	0.737	0.7	2
$M_{3\text{-}D\_QSAR\_Full\_}43$	0.933	0.669	6
$M_{3\text{-}D\_QSAR\_Full\_}44$	0.898	0.73	5
$M_{3\text{-}D\_QSAR\_Full\_}45$	0.757	0.715	2
$M_{3\text{-}D\_QSAR\_Full\_}46$	0.938	0.758	6
$M_{3\text{-}D\_QSAR\_Full\_}47$	0.842	0.708	4
$M_{3\text{-}D\_QSAR\_Full\_}48$	0.877	0.791	4
$M_{3\text{-}D\_QSAR\_Full\_}49$	0.786	0.7	3
$M_{3\text{-}D\_QSAR\_Full\_}50$	0.885	0.751	4
$M_{3\text{-}D\_QSAR\_Full\_}51$	0.945	0.766	7
$M_{3\text{-}D\_QSAR\_Full\_}52$	0.826	0.628	4
$M_{3\text{-}D\_QSAR\_Full\_}53$	0.748	0.66	3
$M_{3\text{-}D\_QSAR\_Full\_}54$	0.911	0.702	5

Table S24. 3-D QSAR models coefficients obtained with the Full dataset.

Model ID	r^2_{cv}	q^2_{cv}	$ONPC_{cv}$
$M_{3\text{-}D\_QSAR\_Full\_}55$	0.617	0.442	2
$M_{3\text{-}D\_QSAR\_Full\_}56$	0.927	0.728	6
$M_{3\text{-}D\_QSAR\_Full\_}57$	0.878	0.677	5
$M_{3\text{-}D\_QSAR\_Full\_}58$	0.87	0.737	4
$M_{3\text{-}D\_QSAR\_Full\_}59$	0.945	0.807	6
$M_{3\text{-}D\_QSAR\_Full\_}60$	0.945	0.765	7
$M_{3\text{-}D\_QSAR\_Full\_}61$	0.842	0.707	4
$M_{3\text{-}D\_QSAR\_Full\_}62$	0.843	0.722	4
$M_{3\text{-}D\_QSAR\_Full\_}63$	0.898	0.767	5
$M_{3\text{-}D\_QSAR\_Full\_}64$	0.9	0.716	5
$M_{3\text{-}D\_QSAR\_Full\_}65$	0.953	0.783	8
$M_{3\text{-}D\_QSAR\_Full\_}66$	0.884	0.764	4
$M_{3\text{-}D\_QSAR\_Full\_}67$	0.949	0.747	7
$M_{3\text{-}D\_QSAR\_Full\_}68$	0.943	0.759	6
$M_{3\text{-}D\_QSAR\_Full\_}69$	0.849	0.75	4
$M_{3\text{-}D\_QSAR\_Full\_}70$	0.923	0.729	6
$M_{3\text{-}D\_QSAR\_Full\_}71$	0.749	0.656	3
$M_{3\text{-}D\_QSAR\_Full\_}72$	0.802	0.721	3
$M_{3\text{-}D\_QSAR\_Full\_}73$	0.799	0.675	4
$M_{3\text{-}D\_QSAR\_Full\_}74$	0.948	0.756	8
$M_{3\text{-}D\_QSAR\_Full\_}75$	0.84	0.677	4
$M_{3\text{-}D\_QSAR\_Full\_}76$	0.878	0.67	4
$M_{3\text{-}D\_QSAR\_Full\_}77$	0.93	0.731	6
$M_{3\text{-}D\_QSAR\_Full\_}78$	0.586	0.45	2
$M_{3\text{-}D\_QSAR\_Full\_}79$	0.769	0.676	3
$M_{3\text{-}D\_QSAR\_Full\_}80$	0.978	0.814	8
$M_{3\text{-}D\_QSAR\_Full\_}81$	0.919	0.751	5
$M_{3\text{-}D\_QSAR\_Full\_}82$	0.916	0.762	5
$M_{3\text{-}D\_QSAR\_Full\_}83$	0.787	0.684	3
$M_{3\text{-}D\_QSAR\_Full\_}84$	0.919	0.751	5
$M_{3\text{-}D\_QSAR\_Full\_}85$	0.929	0.515	6
$M_{3\text{-}D\_QSAR\_Full\_}86$	0.852	0.709	4
$M_{3\text{-}D\_QSAR\_Full\_}87$	0.923	0.702	7
$M_{3\text{-}D\_QSAR\_Full\_}88$	0.756	0.652	3
$M_{3\text{-}D\_QSAR\_Full\_}89$	0.804	0.618	4
$M_{3\text{-}D\_QSAR\_Full\_}90$	0.84	0.728	3
$M_{3\text{-}D\_QSAR\_Full\_}91$	0.926	0.739	6
$M_{3\text{-}D\_QSAR\_Full\_}92$	0.902	0.715	5
$M_{3\text{-}D\_QSAR\_Full\_}93$	0.843	0.719	3
$M_{3\text{-}D\_QSAR\_Full\_}94$	0.738	0.702	2
$M_{3\text{-}D\_QSAR\_Full\_}95$	0.887	0.659	5
$M_{3\text{-}D\_QSAR\_Full\_}96$	0.822	0.644	4
$M_{3\text{-}D\_QSAR\_Full\_}97$	0.908	0.726	5
$M_{3\text{-}D\_QSAR\_Full\_}98$	0.893	0.759	5
$M_{3\text{-}D\_QSAR\_Full\_}99$	0.951	0.786	6
$M_{3\text{-}D\_QSAR\_Full\_}100$	0.768	0.656	3
$M_{3\text{-}D\_QSAR\_Full\_}101$	0.863	0.635	5
$M_{3\text{-}D\_QSAR\_Full\_}102$	0.929	0.511	6
$M_{3\text{-}D\_QSAR\_Full\_}103$	0.889	0.75	4
$M_{3\text{-}D\_QSAR\_Full\_}104$	0.966	0.641	8
$M_{3\text{-}D\_QSAR\_Full\_}105$	0.95	0.693	7
$M_{3\text{-}D\_QSAR\_Full\_}106$	0.923	0.648	6
$M_{3\text{-}D\_QSAR\_Full\_}107$	0.802	0.721	3
$M_{3\text{-}D\_QSAR\_Full\_}108$	0.971	0.675	8

Table S24. 3-D QSAR models coefficients obtained with the Full dataset.

Model ID	r^2_{cv}	q^2_{cv}	$ONPC_{cv}$
$M_{3\text{-}D_QSAR_Full}$_109	0.96	0.754	7
$M_{3\text{-}D_QSAR_Full}$_110	0.848	0.704	4
$M_{3\text{-}D_QSAR_Full}$_111	0.83	0.651	3
$M_{3\text{-}D_QSAR_Full}$_112	0.972	0.726	8
$M_{3\text{-}D_QSAR_Full}$_113	0.851	0.699	4
$M_{3\text{-}D_QSAR_Full}$_114	0.878	0.677	5
$M_{3\text{-}D_QSAR_Full}$_115	0.827	0.676	4
$M_{3\text{-}D_QSAR_Full}$_116	0.919	0.751	5
$M_{3\text{-}D_QSAR_Full}$_117	0.812	0.682	3
$M_{3\text{-}D_QSAR_Full}$_118	0.751	0.463	3
$M_{3\text{-}D_QSAR_Full}$_119	0.924	0.679	6
$M_{3\text{-}D_QSAR_Full}$_120	0.853	0.4	4
$M_{3\text{-}D_QSAR_Full}$_121	0.97	0.727	8
$M_{3\text{-}D_QSAR_Full}$_122	0.954	0.646	7
$M_{3\text{-}D_QSAR_Full}$_123	0.822	0.626	5
$M_{3\text{-}D_QSAR_Full}$_124	0.821	0.675	4
$M_{3\text{-}D_QSAR_Full}$_125	0.916	0.72	5
$M_{3\text{-}D_QSAR_Full}$_126	0.861	0.674	5
$M_{3\text{-}D_QSAR_Full}$_127	0.938	0.669	7
$M_{3\text{-}D_QSAR_Full}$_128	0.978	0.812	8
$M_{3\text{-}D_QSAR_Full}$_129	0.884	0.698	5
$M_{3\text{-}D_QSAR_Full}$_130	0.83	0.618	4
$M_{3\text{-}D_QSAR_Full}$_131	0.934	0.745	6
$M_{3\text{-}D_QSAR_Full}$_132	0.81	0.652	4
$M_{3\text{-}D_QSAR_Full}$_133	0.917	0.675	6
$M_{3\text{-}D_QSAR_Full}$_134	0.768	0.594	3
$M_{3\text{-}D_QSAR_Full}$_135	0.861	0.724	5
$M_{3\text{-}D_QSAR_Full}$_136	0.913	0.698	5
$M_{3\text{-}D_QSAR_Full}$_137	0.831	0.701	4
$M_{3\text{-}D_QSAR_Full}$_138	0.741	0.617	3
$M_{3\text{-}D_QSAR_Full}$_139	0.964	0.758	8
$M_{3\text{-}D_QSAR_Full}$_140	0.927	0.592	6
$M_{3\text{-}D_QSAR_Full}$_141	0.799	0.703	3
$M_{3\text{-}D_QSAR_Full}$_142	0.799	0.673	3
$M_{3\text{-}D_QSAR_Full}$_143	0.98	0.757	8
$M_{3\text{-}D_QSAR_Full}$_144	0.812	0.682	3
$M_{3\text{-}D_QSAR_Full}$_145	0.964	0.756	8
$M_{3\text{-}D_QSAR_Full}$_146	0.888	0.683	5
$M_{3\text{-}D_QSAR_Full}$_147	0.942	0.738	6
$M_{3\text{-}D_QSAR_Full}$_148	0.866	0.622	5
$M_{3\text{-}D_QSAR_Full}$_149	0.917	0.677	6
$M_{3\text{-}D_QSAR_Full}$_150	0.838	0.66	4
$M_{3\text{-}D_QSAR_Full}$_151	0.88	0.697	4
$M_{3\text{-}D_QSAR_Full}$_152	0.877	0.675	5
$M_{3\text{-}D_QSAR_Full}$_153	0.732	0.625	3
$M_{3\text{-}D_QSAR_Full}$_154	0.83	0.657	4
$M_{3\text{-}D_QSAR_Full}$_155	0.764	0.712	2
$M_{3\text{-}D_QSAR_Full}$_156	0.79	0.701	3
$M_{3\text{-}D_QSAR_Full}$_157	0.792	0.69	3
$M_{3\text{-}D_QSAR_Full}$_158	0.962	0.466	7
$M_{3\text{-}D_QSAR_Full}$_159	0.753	0.667	3
$M_{3\text{-}D_QSAR_Full}$_160	0.964	0.759	8
$M_{3\text{-}D_QSAR_Full}$_161	0.765	0.682	3
$M_{3\text{-}D_QSAR_Full}$_162	0.717	0.603	3

Table S24. 3-D QSAR models coefficients obtained with the Full dataset.

Model ID	r^2_{cv}	q^2_{cv}	$ONPC_{cv}$
$M_{3\text{-}D\_QSAR\_Full\_}163$	0.843	0.673	4
$M_{3\text{-}D\_QSAR\_Full\_}164$	0.91	0.77	5
$M_{3\text{-}D\_QSAR\_Full\_}165$	0.763	0.642	3
$M_{3\text{-}D\_QSAR\_Full\_}166$	0.825	0.704	4
$M_{3\text{-}D\_QSAR\_Full\_}167$	0.954	0.692	8
$M_{3\text{-}D\_QSAR\_Full\_}168$	0.681	0.617	2
$M_{3\text{-}D\_QSAR\_Full\_}169$	0.677	0.601	2
$M_{3\text{-}D\_QSAR\_Full\_}170$	0.822	0.627	4
$M_{3\text{-}D\_QSAR\_Full\_}171$	0.843	0.674	4
$M_{3\text{-}D\_QSAR\_Full\_}172$	0.826	0.653	4
$M_{3\text{-}D\_QSAR\_Full\_}173$	0.936	0.656	6
$M_{3\text{-}D\_QSAR\_Full\_}174$	0.872	0.743	4
$M_{3\text{-}D\_QSAR\_Full\_}175$	0.843	0.672	4
$M_{3\text{-}D\_QSAR\_Full\_}176$	0.88	0.629	5
$M_{3\text{-}D\_QSAR\_Full\_}177$	0.895	0.677	5
$M_{3\text{-}D\_QSAR\_Full\_}178$	0.788	0.656	3
$M_{3\text{-}D\_QSAR\_Full\_}179$	0.961	0.752	8
$M_{3\text{-}D\_QSAR\_Full\_}180$	0.905	0.684	5
$M_{3\text{-}D\_QSAR\_Full\_}181$	0.95	0.635	7
$M_{3\text{-}D\_QSAR\_Full\_}182$	0.791	0.682	3
$M_{3\text{-}D\_QSAR\_Full\_}183$	0.769	0.689	3
$M_{3\text{-}D\_QSAR\_Full\_}184$	0.681	0.617	2
$M_{3\text{-}D\_QSAR\_Full\_}185$	0.829	0.64	4
$M_{3\text{-}D\_QSAR\_Full\_}186$	0.717	0.604	3
$M_{3\text{-}D\_QSAR\_Full\_}187$	0.718	0.604	3
$M_{3\text{-}D\_QSAR\_Full\_}188$	0.651	0.573	2
$M_{3\text{-}D\_QSAR\_Full\_}189$	0.948	0.63	7
$M_{3\text{-}D\_QSAR\_Full\_}190$	0.818	0.726	3
$M_{3\text{-}D\_QSAR\_Full\_}191$	0.928	0.746	6
$M_{3\text{-}D\_QSAR\_Full\_}192$	0.783	0.643	3
$M_{3\text{-}D\_QSAR\_Full\_}193$	0.899	0.705	6
$M_{3\text{-}D\_QSAR\_Full\_}194$	0.811	0.714	3
$M_{3\text{-}D\_QSAR\_Full\_}195$	0.869	0.609	4
$M_{3\text{-}D\_QSAR\_Full\_}196$	0.593	0.424	2
$M_{3\text{-}D\_QSAR\_Full\_}197$	0.889	0.689	5
$M_{3\text{-}D\_QSAR\_Full\_}198$	0.895	0.727	5
$M_{3\text{-}D\_QSAR\_Full\_}199$	0.949	0.676	7
$M_{3\text{-}D\_QSAR\_Full\_}200$	0.795	0.679	3
$M_{3\text{-}D\_QSAR\_Full\_}201$	0.885	0.735	4
$M_{3\text{-}D\_QSAR\_Full\_}202$	0.92	0.684	6
$M_{3\text{-}D\_QSAR\_Full\_}203$	0.86	0.658	4
$M_{3\text{-}D\_QSAR\_Full\_}204$	0.876	0.736	4
$M_{3\text{-}D\_QSAR\_Full\_}205$	0.972	0.718	8
$M_{3\text{-}D\_QSAR\_Full\_}206$	0.88	0.675	5
$M_{3\text{-}D\_QSAR\_Full\_}207$	0.919	0.683	6
$M_{3\text{-}D\_QSAR\_Full\_}208$	0.756	0.672	3
$M_{3\text{-}D\_QSAR\_Full\_}209$	0.905	0.725	6
$M_{3\text{-}D\_QSAR\_Full\_}210$	0.881	0.676	5
$M_{3\text{-}D\_QSAR\_Full\_}211$	0.964	0.774	8
$M_{3\text{-}D\_QSAR\_Full\_}212$	0.879	0.742	4
$M_{3\text{-}D\_QSAR\_Full\_}213$	0.87	0.744	4
$M_{3\text{-}D\_QSAR\_Full\_}214$	0.933	0.789	6
$M_{3\text{-}D\_QSAR\_Full\_}215$	0.977	0.745	8
$M_{3\text{-}D\_QSAR\_Full\_}216$	0.773	0.74	2

Table S24. 3-D QSAR models coefficients obtained with the Full dataset.

Model ID	r^2_{cv}	q^2_{cv}	$ONPC_{cv}$
$M_{3\text{-}D\_QSAR\_Full\_}217$	0.858	0.731	4
$M_{3\text{-}D\_QSAR\_Full\_}218$	0.977	0.745	8
$M_{3\text{-}D\_QSAR\_Full\_}219$	0.917	0.7	6
$M_{3\text{-}D\_QSAR\_Full\_}220$	0.892	0.764	5
$M_{3\text{-}D\_QSAR\_Full\_}221$	0.858	0.731	4
$M_{3\text{-}D\_QSAR\_Full\_}222$	0.918	0.79	5
$M_{3\text{-}D\_QSAR\_Full\_}223$	0.939	0.711	7
$M_{3\text{-}D\_QSAR\_Full\_}224$	0.759	0.676	3
$M_{3\text{-}D\_QSAR\_Full\_}225$	0.775	0.719	3
$M_{3\text{-}D\_QSAR\_Full\_}226$	0.961	0.711	8
$M_{3\text{-}D\_QSAR\_Full\_}227$	0.937	0.76	6
$M_{3\text{-}D\_QSAR\_Full\_}228$	0.902	0.742	5
$M_{3\text{-}D\_QSAR\_Full\_}229$	0.95	0.775	7
$M_{3\text{-}D\_QSAR\_Full\_}230$	0.849	0.702	4
$M_{3\text{-}D\_QSAR\_Full\_}231$	0.9	0.731	5
$M_{3\text{-}D\_QSAR\_Full\_}232$	0.953	0.772	7
$M_{3\text{-}D\_QSAR\_Full\_}233$	0.757	0.683	3
$M_{3\text{-}D\_QSAR\_Full\_}234$	0.892	0.774	5
$M_{3\text{-}D\_QSAR\_Full\_}235$	0.937	0.759	6
$M_{3\text{-}D\_QSAR\_Full\_}236$	0.945	0.819	7
$M_{3\text{-}D\_QSAR\_Full\_}237$	0.96	0.772	7
$M_{3\text{-}D\_QSAR\_Full\_}238$	0.961	0.757	8
$M_{3\text{-}D\_QSAR\_Full\_}239$	0.81	0.724	3
$M_{3\text{-}D\_QSAR\_Full\_}240$	0.892	0.763	5
$M_{3\text{-}D\_QSAR\_Full\_}241$	0.971	0.647	8
$M_{3\text{-}D\_QSAR\_Full\_}242$	0.798	0.632	4
$M_{3\text{-}D\_QSAR\_Full\_}243$	0.769	0.539	3
$M_{3\text{-}D\_QSAR\_Full\_}244$	0.827	0.743	3
$M_{3\text{-}D\_QSAR\_Full\_}245$	0.773	0.686	3
$M_{3\text{-}D\_QSAR\_Full\_}246$	0.875	0.708	4
$M_{3\text{-}D\_QSAR\_Full\_}247$	0.838	0.664	4
$M_{3\text{-}D\_QSAR\_Full\_}248$	0.92	0.777	5
$M_{3\text{-}D\_QSAR\_Full\_}249$	0.805	0.672	3
$M_{3\text{-}D\_QSAR\_Full\_}250$	0.833	0.756	3
$M_{3\text{-}D\_QSAR\_Full\_}251$	0.903	0.677	5
$M_{3\text{-}D\_QSAR\_Full\_}252$	0.799	0.711	3
$M_{3\text{-}D\_QSAR\_Full\_}253$	0.798	0.71	3
$M_{3\text{-}D\_QSAR\_Full\_}254$	0.85	0.687	4
$M_{3\text{-}D\_QSAR\_Full\_}255$	0.751	0.6	3
$M_{3\text{-}D\_QSAR\_Full\_}256$	0.827	0.741	3
$M_{3\text{-}D\_QSAR\_Full\_}257$	0.799	0.633	4
$M_{3\text{-}D\_QSAR\_Full\_}258$	0.809	0.728	3
$M_{3\text{-}D\_QSAR\_Full\_}259$	0.836	0.6	4
$M_{3\text{-}D\_QSAR\_Full\_}260$	0.804	0.639	4
$M_{3\text{-}D\_QSAR\_Full\_}261$	0.854	0.523	4
$M_{3\text{-}D\_QSAR\_Full\_}262$	0.778	0.64	3
$M_{3\text{-}D\_QSAR\_Full\_}263$	0.731	0.621	3
$M_{3\text{-}D\_QSAR\_Full\_}264$	0.832	0.689	3
$M_{3\text{-}D\_QSAR\_Full\_}265$	0.711	0.588	3
$M_{3\text{-}D\_QSAR\_Full\_}266$	0.928	0.692	6
$M_{3\text{-}D\_QSAR\_Full\_}267$	0.848	0.652	4
$M_{3\text{-}D\_QSAR\_Full\_}268$	0.898	0.692	5
$M_{3\text{-}D\_QSAR\_Full\_}269$	0.92	0.766	5
$M_{3\text{-}D\_QSAR\_Full\_}270$	0.839	0.715	3

Table S24. 3-D QSAR models coefficients obtained with the Full dataset.

Model ID	r^2_{cv}	q^2_{cv}	$ONPC_{cv}$
$M_{3-D\_QSAR\_Full\_}271$	0.915	0.702	6
$M_{3-D\_QSAR\_Full\_}272$	0.572	0.462	2
$M_{3-D\_QSAR\_Full\_}273$	0.8	0.671	4
$M_{3-D\_QSAR\_Full\_}274$	0.865	0.667	5
$M_{3-D\_QSAR\_Full\_}275$	0.87	0.757	4
$M_{3-D\_QSAR\_Full\_}276$	0.672	0.55	2
$M_{3-D\_QSAR\_Full\_}277$	0.85	0.723	4
$M_{3-D\_QSAR\_Full\_}278$	0.915	0.703	6
$M_{3-D\_QSAR\_Full\_}279$	0.839	0.715	3
$M_{3-D\_QSAR\_Full\_}280$	0.842	0.715	4
$M_{3-D\_QSAR\_Full\_}281$	0.874	0.666	5
$M_{3-D\_QSAR\_Full\_}282$	0.734	0.665	3
$M_{3-D\_QSAR\_Full\_}283$	0.924	0.765	5
$M_{3-D\_QSAR\_Full\_}284$	0.837	0.734	3
$M_{3-D\_QSAR\_Full\_}285$	0.968	0.431	7
$M_{3-D\_QSAR\_Full\_}286$	0.783	0.711	3
$M_{3-D\_QSAR\_Full\_}287$	0.936	0.652	7
$M_{3-D\_QSAR\_Full\_}288$	0.85	0.7	4
$M_{3-D\_QSAR\_Full\_}289$	0.807	0.619	4
$M_{3-D\_QSAR\_Full\_}290$	0.947	0.699	7
$M_{3-D\_QSAR\_Full\_}291$	0.821	0.597	3
$M_{3-D\_QSAR\_Full\_}292$	0.914	0.702	6
$M_{3-D\_QSAR\_Full\_}293$	0.786	0.665	3
$M_{3-D\_QSAR\_Full\_}294$	0.822	0.732	3
$M_{3-D\_QSAR\_Full\_}295$	0.887	0.67	5
$M_{3-D\_QSAR\_Full\_}296$	0.875	0.672	5
$M_{3-D\_QSAR\_Full\_}297$	0.842	0.69	4
$M_{3-D\_QSAR\_Full\_}298$	0.86	0.711	4
$M_{3-D\_QSAR\_Full\_}299$	0.93	0.686	6
$M_{3-D\_QSAR\_Full\_}300$	0.888	0.652	5
$M_{3-D\_QSAR\_Full\_}301$	0.93	0.686	6
$M_{3-D\_QSAR\_Full\_}302$	0.826	0.751	3
$M_{3-D\_QSAR\_Full\_}303$	0.839	0.67	4
$M_{3-D\_QSAR\_Full\_}304$	0.912	0.751	5
$M_{3-D\_QSAR\_Full\_}305$	0.958	0.55	6
$M_{3-D\_QSAR\_Full\_}306$	0.942	0.737	6
$M_{3-D\_QSAR\_Full\_}307$	0.86	0.718	4
$M_{3-D\_QSAR\_Full\_}308$	0.883	0.736	4
$M_{3-D\_QSAR\_Full\_}309$	0.843	0.72	4
$M_{3-D\_QSAR\_Full\_}310$	0.955	0.717	7
$M_{3-D\_QSAR\_Full\_}311$	0.91	0.652	5
$M_{3-D\_QSAR\_Full\_}312$	0.804	0.722	3
$M_{3-D\_QSAR\_Full\_}313$	0.767	0.612	3
$M_{3-D\_QSAR\_Full\_}314$	0.773	0.67	3
$M_{3-D\_QSAR\_Full\_}315$	0.855	0.653	4
$M_{3-D\_QSAR\_Full\_}316$	0.766	0.664	3
$M_{3-D\_QSAR\_Full\_}317$	0.798	0.693	3
$M_{3-D\_QSAR\_Full\_}318$	0.862	0.714	4
$M_{3-D\_QSAR\_Full\_}319$	0.936	0.649	7
$M_{3-D\_QSAR\_Full\_}320$	0.844	0.49	4
$M_{3-D\_QSAR\_Full\_}321$	0.866	0.707	4
$M_{3-D\_QSAR\_Full\_}322$	0.879	0.642	5
$M_{3-D\_QSAR\_Full\_}323$	0.912	0.722	5
$M_{3-D\_QSAR\_Full\_}324$	0.855	0.715	4

Table S24. 3-D QSAR models coefficients obtained with the Full dataset.

Model ID	r^2_{cv}	q^2_{cv}	$ONPC_{cv}$
$M_{3\text{-}D\_QSAR\_Full\_}325$	0.955	0.705	7
$M_{3\text{-}D\_QSAR\_Full\_}326$	0.854	0.714	4
$M_{3\text{-}D\_QSAR\_Full\_}327$	0.892	0.694	5
$M_{3\text{-}D\_QSAR\_Full\_}328$	0.956	0.711	7
$M_{3\text{-}D\_QSAR\_Full\_}329$	0.953	0.81	7
$M_{3\text{-}D\_QSAR\_Full\_}330$	0.931	0.648	6
$M_{3\text{-}D\_QSAR\_Full\_}331$	0.93	0.721	6
$M_{3\text{-}D\_QSAR\_Full\_}332$	0.897	0.61	5
$M_{3\text{-}D\_QSAR\_Full\_}333$	0.75	0.677	3
$M_{3\text{-}D\_QSAR\_Full\_}334$	0.95	0.745	6
$M_{3\text{-}D\_QSAR\_Full\_}335$	0.931	0.645	6
$M_{3\text{-}D\_QSAR\_Full\_}336$	0.786	0.676	3
$M_{3\text{-}D\_QSAR\_Full\_}337$	0.957	0.716	7
$M_{3\text{-}D\_QSAR\_Full\_}338$	0.965	0.629	8
$M_{3\text{-}D\_QSAR\_Full\_}339$	0.885	0.746	4
$M_{3\text{-}D\_QSAR\_Full\_}340$	0.803	0.719	3
$M_{3\text{-}D\_QSAR\_Full\_}341$	0.947	0.681	8
$M_{3\text{-}D\_QSAR\_Full\_}342$	0.779	0.733	2
$M_{3\text{-}D\_QSAR\_Full\_}343$	0.738	0.515	3
$M_{3\text{-}D\_QSAR\_Full\_}344$	0.884	0.685	5
$M_{3\text{-}D\_QSAR\_Full\_}345$	0.575	0.45	2
$M_{3\text{-}D\_QSAR\_Full\_}346$	0.938	0.492	6
$M_{3\text{-}D\_QSAR\_Full\_}347$	0.931	0.645	6
$M_{3\text{-}D\_QSAR\_Full\_}348$	0.89	0.611	5
$M_{3\text{-}D\_QSAR\_Full\_}349$	0.976	0.638	8
$M_{3\text{-}D\_QSAR\_Full\_}350$	0.748	0.65	3
$M_{3\text{-}D\_QSAR\_Full\_}351$	0.936	0.589	6
$M_{3\text{-}D\_QSAR\_Full\_}352$	0.415	0.376	1
$M_{3\text{-}D\_QSAR\_Full\_}353$	0.892	0.697	5
$M_{3\text{-}D\_QSAR\_Full\_}354$	0.869	0.666	4
$M_{3\text{-}D\_QSAR\_Full\_}355$	0.891	0.647	5
$M_{3\text{-}D\_QSAR\_Full\_}356$	0.951	0.706	6
$M_{3\text{-}D\_QSAR\_Full\_}357$	0.961	0.628	8
$M_{3\text{-}D\_QSAR\_Full\_}358$	0.841	0.633	4
$M_{3\text{-}D\_QSAR\_Full\_}359$	0.81	0.667	4
$M_{3\text{-}D\_QSAR\_Full\_}360$	0.843	0.711	4
$M_{3\text{-}D\_QSAR\_Full\_}361$	0.906	0.695	5
$M_{3\text{-}D\_QSAR\_Full\_}362$	0.776	0.65	3
$M_{3\text{-}D\_QSAR\_Full\_}363$	0.729	0.672	2
$M_{3\text{-}D\_QSAR\_Full\_}364$	0.759	0.649	3
$M_{3\text{-}D\_QSAR\_Full\_}365$	0.978	0.745	8
$M_{3\text{-}D\_QSAR\_Full\_}366$	0.587	0.421	2
$M_{3\text{-}D\_QSAR\_Full\_}367$	0.908	0.717	6
$M_{3\text{-}D\_QSAR\_Full\_}368$	0.906	0.698	5
$M_{3\text{-}D\_QSAR\_Full\_}369$	0.902	0.617	5
$M_{3\text{-}D\_QSAR\_Full\_}370$	0.936	0.589	6
$M_{3\text{-}D\_QSAR\_Full\_}371$	0.876	0.742	4
$M_{3\text{-}D\_QSAR\_Full\_}372$	0.749	0.635	3
$M_{3\text{-}D\_QSAR\_Full\_}373$	0.763	0.641	3
$M_{3\text{-}D\_QSAR\_Full\_}374$	0.899	0.645	5
$M_{3\text{-}D\_QSAR\_Full\_}375$	0.957	0.742	6
$M_{3\text{-}D\_QSAR\_Full\_}376$	0.781	0.691	3
$M_{3\text{-}D\_QSAR\_Full\_}377$	0.963	0.734	8
$M_{3\text{-}D\_QSAR\_Full\_}378$	0.783	0.653	3

Table S24. 3-D QSAR models coefficients obtained with the Full dataset.

**Model ID**	r^2_{cv}	q^2_{cv}	$ONPC_{cv}$
$M_{3\text{-}D\_QSAR\_Full\_}379$	0.902	0.797	4
$M_{3\text{-}D\_QSAR\_Full\_}380$	0.894	0.771	4
$M_{3\text{-}D\_QSAR\_Full\_}381$	0.929	0.784	5
$M_{3\text{-}D\_QSAR\_Full\_}382$	0.827	0.689	4
$M_{3\text{-}D\_QSAR\_Full\_}383$	0.881	0.656	5
$M_{3\text{-}D\_QSAR\_Full\_}384$	0.927	0.679	6
$M_{3\text{-}D\_QSAR\_Full\_}385$	0.938	0.645	6
$M_{3\text{-}D\_QSAR\_Full\_}386$	0.881	0.656	5
$M_{3\text{-}D\_QSAR\_Full\_}387$	0.952	0.771	7
$M_{3\text{-}D\_QSAR\_Full\_}388$	0.878	0.645	5
$M_{3\text{-}D\_QSAR\_Full\_}389$	0.947	0.688	8
$M_{3\text{-}D\_QSAR\_Full\_}390$	0.931	0.755	7
$M_{3\text{-}D\_QSAR\_Full\_}391$	0.803	0.656	4
$M_{3\text{-}D\_QSAR\_Full\_}392$	0.902	0.797	4
$M_{3\text{-}D\_QSAR\_Full\_}393$	0.908	0.695	6
$M_{3\text{-}D\_QSAR\_Full\_}394$	0.758	0.678	3
$M_{3\text{-}D\_QSAR\_Full\_}395$	0.881	0.656	5
$M_{3\text{-}D\_QSAR\_Full\_}396$	0.896	0.671	5
$M_{3\text{-}D\_QSAR\_Full\_}397$	0.827	0.745	3
$M_{3\text{-}D\_QSAR\_Full\_}398$	0.83	0.748	3
$M_{3\text{-}D\_QSAR\_Full\_}399$	0.799	0.72	3
$M_{3\text{-}D\_QSAR\_Full\_}400$	0.758	0.678	3
$M_{3\text{-}D\_QSAR\_Full\_}401$	0.95	0.804	6
$M_{3\text{-}D\_QSAR\_Full\_}402$	0.862	0.78	3
$M_{3\text{-}D\_QSAR\_Full\_}403$	0.968	0.752	8
$M_{3\text{-}D\_QSAR\_Full\_}404$	0.815	0.697	3
$M_{3\text{-}D\_QSAR\_Full\_}405$	0.89	0.735	4
$M_{3\text{-}D\_QSAR\_Full\_}406$	0.865	0.713	4
$M_{3\text{-}D\_QSAR\_Full\_}407$	0.779	0.676	3
$M_{3\text{-}D\_QSAR\_Full\_}408$	0.879	0.646	5
$M_{3\text{-}D\_QSAR\_Full\_}409$	0.771	0.66	3
$M_{3\text{-}D\_QSAR\_Full\_}410$	0.882	0.592	4
$M_{3\text{-}D\_QSAR\_Full\_}411$	0.92	0.786	5
$M_{3\text{-}D\_QSAR\_Full\_}412$	0.865	0.713	4
$M_{3\text{-}D\_QSAR\_Full\_}413$	0.833	0.617	4
$M_{3\text{-}D\_QSAR\_Full\_}414$	0.742	0.501	3
$M_{3\text{-}D\_QSAR\_Full\_}415$	0.755	0.657	3
$M_{3\text{-}D\_QSAR\_Full\_}416$	0.965	0.745	6
$M_{3\text{-}D\_QSAR\_Full\_}417$	0.806	0.674	3
$M_{3\text{-}D\_QSAR\_Full\_}418$	0.851	0.616	4
$M_{3\text{-}D\_QSAR\_Full\_}419$	0.853	0.637	3
$M_{3\text{-}D\_QSAR\_Full\_}420$	0.798	0.716	3
$M_{3\text{-}D\_QSAR\_Full\_}421$	0.841	0.671	4
$M_{3\text{-}D\_QSAR\_Full\_}422$	0.804	0.632	4
$M_{3\text{-}D\_QSAR\_Full\_}423$	0.844	0.732	4
$M_{3\text{-}D\_QSAR\_Full\_}424$	0.903	0.7	5
$M_{3\text{-}D\_QSAR\_Full\_}425$	0.75	0.655	3
$M_{3\text{-}D\_QSAR\_Full\_}426$	0.813	0.728	3
$M_{3\text{-}D\_QSAR\_Full\_}427$	0.783	0.65	3
$M_{3\text{-}D\_QSAR\_Full\_}428$	0.844	0.731	4
$M_{3\text{-}D\_QSAR\_Full\_}429$	0.85	0.616	4
$M_{3\text{-}D\_QSAR\_Full\_}430$	0.789	0.685	3

Table S25. Details for $M_{3\text{-}D\_QSAR\_Full\_236}$ model

PC	r^2	SDEC	q^2_{cv}	SDEP $_{cv}$
1	0.45	0.64	0.43	0.65
2	0.73	0.45	0.69	0.49
3	0.83	0.36	0.76	0.42
4	0.88	0.30	0.79	0.40
5	0.92	0.25	0.81	0.38
6	0.93	0.24	0.81	0.38
7*	0.95	0.20	0.82	0.37
8	0.96	0.18	0.81	0.38

The star indicate the optimal number of PCs selected by a the max q^2_{cv} value.

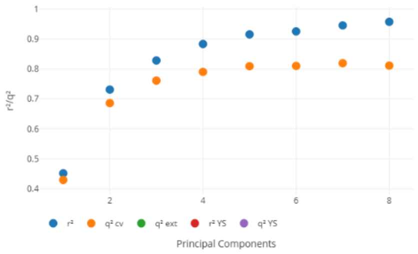

A

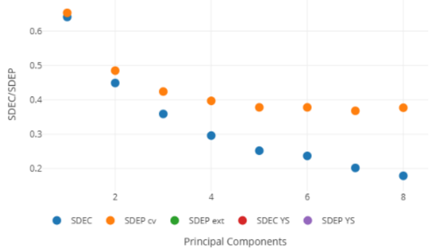

B

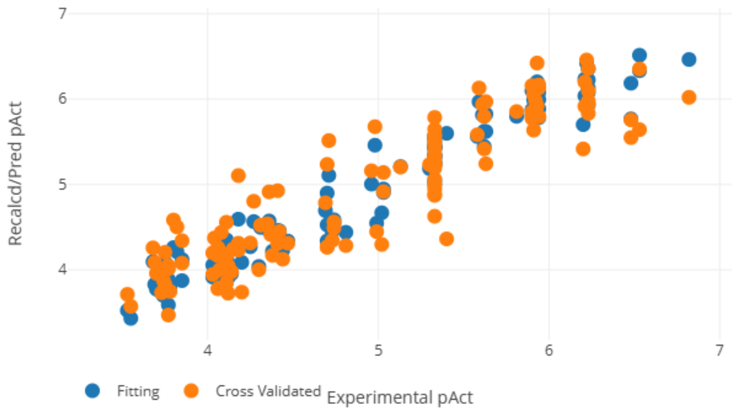

C

Figure S7. Full dataset $M_{3\text{-}D\_QSAR\_Full\_236}$ model: (A) r^2 and q^2_{cv} values in relation to number of PCs. (B) SDEC and SDEPcv in relation with the number of PC and the (C) experimental versus the recalculated/CV-predicted pMIC (pAct) values at 7 PCs. In blue, the r^2, SDEC, and experimental fitted values. In orange, the q^2_{cv}, $SDEP_{cv}$, and crossvalidated values.

Table S26. 3-D QSAR models coefficients obtained with the Splitted dataset

Dataset ID	r^2_{cv}	q^2_{cv}	$ONPC_{cv}$	r^2_{pred}	q^2_{pred}	$ONPC_{pred}$
$M_{3-D\_QSAR\_Splitted\_}1$	0.866	0.655	4	0.866	0.545	4
$M_{3-D\_QSAR\_Splitted\_}2$	0.831	0.76	3	0.831	0.728	3
$M_{3-D\_QSAR\_Splitted\_}3$	0.879	0.702	4	0.879	0.661	4
$M_{3-D\_QSAR\_Splitted\_}4$	0.823	0.747	3	0.89	0.759	4
$M_{3-D\_QSAR\_Splitted\_}5$	0.97	0.711	7	0.864	0.565	4
$M_{3-D\_QSAR\_Splitted\_}6$	0.725	0.635	2	0.887	0.62	4
$M_{3-D\_QSAR\_Splitted\_}7$	0.953	0.778	5	0.978	0.716	8
$M_{3-D\_QSAR\_Splitted\_}8$	0.965	0.686	7	0.913	0.744	5
$M_{3-D\_QSAR\_Splitted\_}9$	0.989	0.654	8	0.792	0.681	3
$M_{3-D\_QSAR\_Splitted\_}10$	0.915	0.677	5	0.879	0.623	4
$M_{3-D\_QSAR\_Splitted\_}11$	0.792	0.65	3	0.682	0.692	2
$M_{3-D\_QSAR\_Splitted\_}12$	0.791	0.633	3	0.701	0.528	2
$M_{3-D\_QSAR\_Splitted\_}13$	0.939	0.702	5	0.893	0.632	4
$M_{3-D\_QSAR\_Splitted\_}14$	0.866	0.779	3	0.866	0.749	3
$M_{3-D\_QSAR\_Splitted\_}15$	0.943	0.793	5	0.873	0.651	3
$M_{3-D\_QSAR\_Splitted\_}16$	0.987	0.762	8	0.949	0.741	5
$M_{3-D\_QSAR\_Splitted\_}17$	0.768	0.652	3	0.768	0.671	3
$M_{3-D\_QSAR\_Splitted\_}18$	0.911	0.746	5	0.829	0.682	3
$M_{3-D\_QSAR\_Splitted\_}19$	0.955	0.667	6	0.935	0.724	5
$M_{3-D\_QSAR\_Splitted\_}20$	0.796	0.732	3	0.933	0.645	5
$M_{3-D\_QSAR\_Splitted\_}21$	0.944	0.669	6	0.858	0.745	4
$M_{3-D\_QSAR\_Splitted\_}22$	0.955	0.75	7	0.967	0.748	8
$M_{3-D\_QSAR\_Splitted\_}23$	0.866	0.698	4	0.741	0.67	2
$M_{3-D\_QSAR\_Splitted\_}24$	0.884	0.668	4	0.809	0.671	3
$M_{3-D\_QSAR\_Splitted\_}25$	0.616	0.425	2	0.616	0.517	2
$M_{3-D\_QSAR\_Splitted\_}26$	0.929	0.742	5	0.891	0.676	4
$M_{3-D\_QSAR\_Splitted\_}27$	0.812	0.713	3	0.877	0.683	4
$M_{3-D\_QSAR\_Splitted\_}28$	0.917	0.771	5	0.844	0.699	3
$M_{3-D\_QSAR\_Splitted\_}29$	0.949	0.72	6	0.949	0.652	6
$M_{3-D\_QSAR\_Splitted\_}30$	0.78	0.683	3	0.78	0.737	3
$M_{3-D\_QSAR\_Splitted\_}31$	0.98	0.808	7	0.812	0.727	3
$M_{3-D\_QSAR\_Splitted\_}32$	0.95	0.799	5	0.964	0.733	6
$M_{3-D\_QSAR\_Splitted\_}33$	0.981	0.739	8	0.8	0.631	3
$M_{3-D\_QSAR\_Splitted\_}34$	0.813	0.681	3	0.904	0.622	5
$M_{3-D\_QSAR\_Splitted\_}35$	0.936	0.678	6	0.936	0.71	6
$M_{3-D\_QSAR\_Splitted\_}36$	0.972	0.595	8	0.867	0.65	4
$M_{3-D\_QSAR\_Splitted\_}37$	0.96	0.704	7	0.952	0.711	6
$M_{3-D\_QSAR\_Splitted\_}38$	0.946	0.766	5	0.946	0.692	5
$M_{3-D\_QSAR\_Splitted\_}39$	0.951	0.787	6	0.857	0.716	3
$M_{3-D\_QSAR\_Splitted\_}40$	0.714	0.667	2	0.959	0.715	7
$M_{3-D\_QSAR\_Splitted\_}41$	0.943	0.707	5	0.756	0.675	3
$M_{3-D\_QSAR\_Splitted\_}42$	0.953	0.778	5	0.978	0.718	8
$M_{3-D\_QSAR\_Splitted\_}43$	0.979	0.787	8	0.863	0.691	3
$M_{3-D\_QSAR\_Splitted\_}44$	0.934	0.753	5	0.836	0.671	3
$M_{3-D\_QSAR\_Splitted\_}45$	0.969	0.694	6	0.894	0.647	3
$M_{3-D\_QSAR\_Splitted\_}46$	0.938	0.797	5	0.787	0.753	2
$M_{3-D\_QSAR\_Splitted\_}47$	0.974	0.741	8	0.777	0.689	2
$M_{3-D\_QSAR\_Splitted\_}48$	0.965	0.727	7	0.777	0.59	3
$M_{3-D\_QSAR\_Splitted\_}49$	0.856	0.607	4	0.683	0.599	2
$M_{3-D\_QSAR\_Splitted\_}50$	0.934	0.753	5	0.836	0.671	3
$M_{3-D\_QSAR\_Splitted\_}51$	0.894	0.774	4	0.981	0.678	8
$M_{3-D\_QSAR\_Splitted\_}52$	0.897	0.692	5	0.897	0.643	5
$M_{3-D\_QSAR\_Splitted\_}53$	0.837	0.683	3	0.912	0.619	5

Table S26. 3-D QSAR models coefficients obtained with the Splitted dataset

Dataset ID	r^2_{cv}	q^2_{cv}	$ONPC_{cv}$	r^2_{pred}	q^2_{pred}	$ONPC_{pred}$
$M_{3\text{-}D\_QSAR\_Splitted\_}54$	0.866	0.779	3	0.866	0.749	3
$M_{3\text{-}D\_QSAR\_Splitted\_}55$	0.934	0.494	5	0.976	0.571	7
$M_{3\text{-}D\_QSAR\_Splitted\_}56$	0.842	0.707	3	0.842	0.645	3
$M_{3\text{-}D\_QSAR\_Splitted\_}57$	0.943	0.731	6	0.922	0.651	5
$M_{3\text{-}D\_QSAR\_Splitted\_}58$	0.946	0.761	5	0.849	0.697	3
$M_{3\text{-}D\_QSAR\_Splitted\_}59$	0.948	0.724	6	0.779	0.643	3
$M_{3\text{-}D\_QSAR\_Splitted\_}60$	0.829	0.743	3	0.723	0.619	2
$M_{3\text{-}D\_QSAR\_Splitted\_}61$	0.954	0.742	6	0.825	0.696	3
$M_{3\text{-}D\_QSAR\_Splitted\_}62$	0.914	0.763	4	0.914	0.712	4
$M_{3\text{-}D\_QSAR\_Splitted\_}63$	0.975	0.7	7	0.982	0.665	8
$M_{3\text{-}D\_QSAR\_Splitted\_}64$	0.973	0.776	7	0.913	0.745	4
$M_{3\text{-}D\_QSAR\_Splitted\_}65$	0.814	0.714	3	0.958	0.675	7
$M_{3\text{-}D\_QSAR\_Splitted\_}66$	0.83	0.755	3	0.734	0.645	2
$M_{3\text{-}D\_QSAR\_Splitted\_}67$	0.969	0.826	7	0.975	0.657	8
$M_{3\text{-}D\_QSAR\_Splitted\_}68$	0.943	0.699	6	0.892	0.75	4
$M_{3\text{-}D\_QSAR\_Splitted\_}69$	0.964	0.668	7	0.784	0.649	3
$M_{3\text{-}D\_QSAR\_Splitted\_}70$	0.838	0.669	3	0.941	0.735	6
$M_{3\text{-}D\_QSAR\_Splitted\_}71$	0.833	0.681	3	0.867	0.648	4
$M_{3\text{-}D\_QSAR\_Splitted\_}72$	0.965	0.7	7	0.786	0.666	3
$M_{3\text{-}D\_QSAR\_Splitted\_}73$	0.956	0.713	6	0.849	0.763	3
$M_{3\text{-}D\_QSAR\_Splitted\_}74$	0.83	0.755	3	0.734	0.645	2
$M_{3\text{-}D\_QSAR\_Splitted\_}75$	0.955	0.756	6	0.8	0.694	2
$M_{3\text{-}D\_QSAR\_Splitted\_}76$	0.972	0.595	8	0.867	0.65	4
$M_{3\text{-}D\_QSAR\_Splitted\_}77$	0.809	0.703	3	0.959	0.693	8
$M_{3\text{-}D\_QSAR\_Splitted\_}78$	0.74	0.635	3	0.84	0.705	4
$M_{3\text{-}D\_QSAR\_Splitted\_}79$	0.931	0.788	4	0.97	0.78	7
$M_{3\text{-}D\_QSAR\_Splitted\_}80$	0.973	0.809	7	0.909	0.76	4
$M_{3\text{-}D\_QSAR\_Splitted\_}81$	0.87	0.792	3	0.805	0.615	2
$M_{3\text{-}D\_QSAR\_Splitted\_}82$	0.935	0.766	5	0.758	0.684	2
$M_{3\text{-}D\_QSAR\_Splitted\_}83$	0.983	0.671	8	0.983	0.748	8
$M_{3\text{-}D\_QSAR\_Splitted\_}84$	0.989	0.654	8	0.792	0.681	3
$M_{3\text{-}D\_QSAR\_Splitted\_}85$	0.931	0.788	4	0.97	0.78	7
$M_{3\text{-}D\_QSAR\_Splitted\_}86$	0.949	0.72	6	0.949	0.652	6
$M_{3\text{-}D\_QSAR\_Splitted\_}87$	0.821	0.72	3	0.821	0.57	3
$M_{3\text{-}D\_QSAR\_Splitted\_}88$	0.818	0.623	3	0.818	0.616	3
$M_{3\text{-}D\_QSAR\_Splitted\_}89$	0.84	0.517	4	0.741	0.714	3
$M_{3\text{-}D\_QSAR\_Splitted\_}90$	0.973	0.792	7	0.95	0.703	5
$M_{3\text{-}D\_QSAR\_Splitted\_}91$	0.986	0.753	8	0.897	0.714	4
$M_{3\text{-}D\_QSAR\_Splitted\_}92$	0.871	0.745	4	0.871	0.657	4
$M_{3\text{-}D\_QSAR\_Splitted\_}93$	0.848	0.748	3	0.925	0.721	5
$M_{3\text{-}D\_QSAR\_Splitted\_}94$	0.94	0.693	5	0.7	0.656	2
$M_{3\text{-}D\_QSAR\_Splitted\_}95$	0.976	0.728	8	0.793	0.579	3
$M_{3\text{-}D\_QSAR\_Splitted\_}96$	0.796	0.732	3	0.933	0.645	5
$M_{3\text{-}D\_QSAR\_Splitted\_}97$	0.83	0.619	3	0.672	0.575	2
$M_{3\text{-}D\_QSAR\_Splitted\_}98$	0.98	0.738	8	0.659	0.711	2
$M_{3\text{-}D\_QSAR\_Splitted\_}99$	0.969	0.667	7	0.809	0.571	3
$M_{3\text{-}D\_QSAR\_Splitted\_}100$	0.867	0.792	3	0.91	0.644	4
$M_{3\text{-}D\_QSAR\_Splitted\_}101$	0.885	0.645	4	0.984	0.545	8
$M_{3\text{-}D\_QSAR\_Splitted\_}102$	0.884	0.723	4	0.884	0.767	4
$M_{3\text{-}D\_QSAR\_Splitted\_}103$	0.934	0.7	6	0.911	0.733	5
$M_{3\text{-}D\_QSAR\_Splitted\_}104$	0.941	0.8	5	0.913	0.717	4
$M_{3\text{-}D\_QSAR\_Splitted\_}105$	0.954	0.666	6	0.763	0.611	3
$M_{3\text{-}D\_QSAR\_Splitted\_}106$	0.937	0.711	5	0.871	0.638	3
$M_{3\text{-}D\_QSAR\_Splitted\_}107$	0.781	0.696	2	0.781	0.642	2

Table S26. 3-D QSAR models coefficients obtained with the Splitted dataset

Dataset ID	r^2_{cv}	q^2_{cv}	$ONPC_{cv}$	r^2_{pred}	q^2_{pred}	$ONPC_{pred}$
$M_{3\text{-}D_QSAR_Splitted}$_108	0.768	0.652	3	0.768	0.671	3
$M_{3\text{-}D_QSAR_Splitted}$_109	0.963	0.755	6	0.977	0.803	7
$M_{3\text{-}D_QSAR_Splitted}$_110	0.92	0.78	5	0.935	0.779	6
$M_{3\text{-}D_QSAR_Splitted}$_111	0.943	0.762	6	0.88	0.74	4
$M_{3\text{-}D_QSAR_Splitted}$_112	0.914	0.714	5	0.716	0.687	2
$M_{3\text{-}D_QSAR_Splitted}$_113	0.919	0.714	5	0.895	0.701	4
$M_{3\text{-}D_QSAR_Splitted}$_114	0.984	0.819	8	0.946	0.691	5
$M_{3\text{-}D_QSAR_Splitted}$_115	0.778	0.67	3	0.935	0.671	5
$M_{3\text{-}D_QSAR_Splitted}$_116	0.918	0.729	5	0.81	0.734	3
$M_{3\text{-}D_QSAR_Splitted}$_117	0.954	0.798	6	0.954	0.736	6
$M_{3\text{-}D_QSAR_Splitted}$_118	0.797	0.432	3	0.619	0.546	2
$M_{3\text{-}D_QSAR_Splitted}$_119	0.924	0.752	5	0.955	0.682	6
$M_{3\text{-}D_QSAR_Splitted}$_120	0.815	0.71	3	0.874	0.729	4
$M_{3\text{-}D_QSAR_Splitted}$_121	0.976	0.617	8	0.713	0.571	2
$M_{3\text{-}D_QSAR_Splitted}$_122	0.96	0.704	7	0.952	0.711	6
$M_{3\text{-}D_QSAR_Splitted}$_123	0.95	0.734	6	0.898	0.614	4
$M_{3\text{-}D_QSAR_Splitted}$_124	0.95	0.709	6	0.76	0.684	3
$M_{3\text{-}D_QSAR_Splitted}$_125	0.986	0.804	8	0.967	0.694	6
$M_{3\text{-}D_QSAR_Splitted}$_126	0.937	0.778	5	0.937	0.725	5
$M_{3\text{-}D_QSAR_Splitted}$_127	0.897	0.784	4	0.937	0.711	6
$M_{3\text{-}D_QSAR_Splitted}$_128	0.817	0.772	2	0.883	0.661	3
$M_{3\text{-}D_QSAR_Splitted}$_129	0.95	0.663	6	0.833	0.738	4
$M_{3\text{-}D_QSAR_Splitted}$_130	0.91	0.682	5	0.763	0.732	3
$M_{3\text{-}D_QSAR_Splitted}$_131	0.823	0.674	3	0.714	0.597	2
$M_{3\text{-}D_QSAR_Splitted}$_132	0.908	0.74	5	0.876	0.676	4
$M_{3\text{-}D_QSAR_Splitted}$_133	0.9	0.673	5	0.9	0.754	5
$M_{3\text{-}D_QSAR_Splitted}$_134	0.813	0.681	3	0.904	0.623	5
$M_{3\text{-}D_QSAR_Splitted}$_135	0.955	0.667	6	0.935	0.724	5
$M_{3\text{-}D_QSAR_Splitted}$_136	0.813	0.683	3	0.87	0.651	4
$M_{3\text{-}D_QSAR_Splitted}$_137	0.823	0.747	3	0.89	0.759	4
$M_{3\text{-}D_QSAR_Splitted}$_138	0.853	0.729	3	0.955	0.651	6
$M_{3\text{-}D_QSAR_Splitted}$_139	0.692	0.535	2	0.692	0.617	2
$M_{3\text{-}D_QSAR_Splitted}$_140	0.864	0.696	3	0.912	0.638	4
$M_{3\text{-}D_QSAR_Splitted}$_141	0.914	0.718	5	0.787	0.652	3
$M_{3\text{-}D_QSAR_Splitted}$_142	0.946	0.664	6	0.802	0.75	3
$M_{3\text{-}D_QSAR_Splitted}$_143	0.935	0.766	5	0.758	0.684	2
$M_{3\text{-}D_QSAR_Splitted}$_144	0.892	0.72	4	0.832	0.63	3
$M_{3\text{-}D_QSAR_Splitted}$_145	0.975	0.665	8	0.848	0.581	4
$M_{3\text{-}D_QSAR_Splitted}$_146	0.937	0.778	5	0.937	0.724	5
$M_{3\text{-}D_QSAR_Splitted}$_147	0.468	0.412	1	0.755	0.547	3
$M_{3\text{-}D_QSAR_Splitted}$_148	0.857	0.688	3	0.751	0.593	2
$M_{3\text{-}D_QSAR_Splitted}$_149	0.906	0.832	4	0.949	0.758	6
$M_{3\text{-}D_QSAR_Splitted}$_150	0.919	0.48	5	0.368	0.492	1
$M_{3\text{-}D_QSAR_Splitted}$_151	0.907	0.688	5	0.942	0.755	6
$M_{3\text{-}D_QSAR_Splitted}$_152	0.921	0.763	5	0.884	0.679	4
$M_{3\text{-}D_QSAR_Splitted}$_153	0.94	0.752	5	0.954	0.707	6
$M_{3\text{-}D_QSAR_Splitted}$_154	0.955	0.782	6	0.822	0.679	3
$M_{3\text{-}D_QSAR_Splitted}$_155	0.919	0.794	5	0.919	0.732	5
$M_{3\text{-}D_QSAR_Splitted}$_156	0.897	0.765	3	0.82	0.63	2
$M_{3\text{-}D_QSAR_Splitted}$_157	0.955	0.736	6	0.775	0.581	2
$M_{3\text{-}D_QSAR_Splitted}$_158	0.884	0.723	4	0.884	0.767	4
$M_{3\text{-}D_QSAR_Splitted}$_159	0.936	0.78	5	0.9	0.74	4
$M_{3\text{-}D_QSAR_Splitted}$_160	0.937	0.749	5	0.937	0.62	5
$M_{3\text{-}D_QSAR_Splitted}$_161	0.973	0.792	7	0.95	0.703	5

Table S26. 3-D QSAR models coefficients obtained with the Splitted dataset

Dataset ID	r^2_{cv}	q^2_{cv}	$ONPC_{cv}$	r^2_{pred}	q^2_{pred}	$ONPC_{pred}$
$M_{3-D_QSAR_Splitted}$_162	0.814	0.714	3	0.958	0.674	7
$M_{3-D_QSAR_Splitted}$_163	0.93	0.77	4	0.767	0.675	2
$M_{3-D_QSAR_Splitted}$_164	0.974	0.792	7	0.985	0.711	8
$M_{3-D_QSAR_Splitted}$_165	0.974	0.776	8	0.832	0.715	3
$M_{3-D_QSAR_Splitted}$_166	0.921	0.778	4	0.88	0.723	3
$M_{3-D_QSAR_Splitted}$_167	0.975	0.728	8	0.72	0.557	2
$M_{3-D_QSAR_Splitted}$_168	0.988	0.709	8	0.74	0.61	2
$M_{3-D_QSAR_Splitted}$_169	0.978	0.664	8	0.879	0.599	4
$M_{3-D_QSAR_Splitted}$_170	0.862	0.642	4	0.918	0.823	5
$M_{3-D_QSAR_Splitted}$_171	0.943	0.731	6	0.922	0.651	5
$M_{3-D_QSAR_Splitted}$_172	0.8	0.666	3	0.8	0.702	3
$M_{3-D_QSAR_Splitted}$_173	0.969	0.694	6	0.894	0.644	3
$M_{3-D_QSAR_Splitted}$_174	0.919	0.48	5	0.368	0.492	1
$M_{3-D_QSAR_Splitted}$_175	0.924	0.736	5	0.879	0.526	4
$M_{3-D_QSAR_Splitted}$_176	0.888	0.735	4	0.83	0.736	3
$M_{3-D_QSAR_Splitted}$_177	0.91	0.754	4	0.942	0.635	5
$M_{3-D_QSAR_Splitted}$_178	0.823	0.703	3	0.874	0.604	4
$M_{3-D_QSAR_Splitted}$_179	0.946	0.763	5	0.894	0.701	4
$M_{3-D_QSAR_Splitted}$_180	0.969	0.801	8	0.874	0.653	3
$M_{3-D_QSAR_Splitted}$_181	0.93	0.77	4	0.767	0.675	2
$M_{3-D_QSAR_Splitted}$_182	0.921	0.778	4	0.88	0.723	3
$M_{3-D_QSAR_Splitted}$_183	0.759	0.628	3	0.864	0.647	4
$M_{3-D_QSAR_Splitted}$_184	0.955	0.783	6	0.938	0.765	5
$M_{3-D_QSAR_Splitted}$_185	0.953	0.394	6	0.88	0.538	4
$M_{3-D_QSAR_Splitted}$_186	0.944	0.766	5	0.802	0.517	2
$M_{3-D_QSAR_Splitted}$_187	0.787	0.671	3	0.87	0.595	4
$M_{3-D_QSAR_Splitted}$_188	0.775	0.686	3	0.865	0.624	4
$M_{3-D_QSAR_Splitted}$_189	0.945	0.797	5	0.923	0.763	4
$M_{3-D_QSAR_Splitted}$_190	0.772	0.641	3	0.959	0.716	7
$M_{3-D_QSAR_Splitted}$_191	0.841	0.731	3	0.93	0.702	5
$M_{3-D_QSAR_Splitted}$_192	0.901	0.721	4	0.901	0.5	4
$M_{3-D_QSAR_Splitted}$_193	0.984	0.742	8	0.734	0.568	2
$M_{3-D_QSAR_Splitted}$_194	0.892	0.738	4	0.735	0.661	2
$M_{3-D_QSAR_Splitted}$_195	0.801	0.687	3	0.858	0.634	4
$M_{3-D_QSAR_Splitted}$_196	0.944	0.766	5	0.802	0.516	2
$M_{3-D_QSAR_Splitted}$_197	0.946	0.763	5	0.894	0.701	4
$M_{3-D_QSAR_Splitted}$_198	0.966	0.801	6	0.88	0.651	3
$M_{3-D_QSAR_Splitted}$_199	0.914	0.714	5	0.716	0.688	2
$M_{3-D_QSAR_Splitted}$_200	0.95	0.656	5	0.834	0.591	3
$M_{3-D_QSAR_Splitted}$_201	0.823	0.703	3	0.874	0.605	4
$M_{3-D_QSAR_Splitted}$_202	0.773	0.712	2	0.97	0.713	7
$M_{3-D_QSAR_Splitted}$_203	0.869	0.701	4	0.965	0.636	7
$M_{3-D_QSAR_Splitted}$_204	0.749	0.639	3	0.921	0.651	5
$M_{3-D_QSAR_Splitted}$_205	0.847	0.659	4	0.78	0.646	3
$M_{3-D_QSAR_Splitted}$_206	0.967	0.758	7	0.709	0.667	2
$M_{3-D_QSAR_Splitted}$_207	0.942	0.768	6	0.892	0.73	4
$M_{3-D_QSAR_Splitted}$_208	0.982	0.73	8	0.828	0.522	3
$M_{3-D_QSAR_Splitted}$_209	0.749	0.639	3	0.921	0.65	5
$M_{3-D_QSAR_Splitted}$_210	0.777	0.591	3	0.881	0.74	4
$M_{3-D_QSAR_Splitted}$_211	0.832	0.528	3	0.832	0.433	3
$M_{3-D_QSAR_Splitted}$_212	0.847	0.754	3	0.847	0.679	3
$M_{3-D_QSAR_Splitted}$_213	0.819	0.665	3	0.819	0.589	3
$M_{3-D_QSAR_Splitted}$_214	0.951	0.716	6	0.885	0.675	4
$M_{3-D_QSAR_Splitted}$_215	0.663	0.59	2	0.798	0.734	3

Table S26. 3-D QSAR models coefficients obtained with the Splitted dataset

Dataset ID	r^2_{cv}	q^2_{cv}	$ONPC_{cv}$	r^2_{pred}	q^2_{pred}	$ONPC_{pred}$
$M_{3-D_QSAR_Splitted}$_216	0.939	0.767	5	0.963	0.707	7
$M_{3-D_QSAR_Splitted}$_217	0.896	0.756	4	0.896	0.7	4
$M_{3-D_QSAR_Splitted}$_218	0.927	0.71	5	0.976	0.702	8
$M_{3-D_QSAR_Splitted}$_219	0.94	0.695	6	0.94	0.663	6
$M_{3-D_QSAR_Splitted}$_220	0.977	0.753	8	0.948	0.632	5
$M_{3-D_QSAR_Splitted}$_221	0.96	0.643	6	0.85	0.621	3
$M_{3-D_QSAR_Splitted}$_222	0.776	0.685	3	0.735	0.631	2
$M_{3-D_QSAR_Splitted}$_223	0.937	0.771	5	0.757	0.652	2
$M_{3-D_QSAR_Splitted}$_224	0.919	0.658	5	0.877	0.72	4
$M_{3-D_QSAR_Splitted}$_225	0.754	0.634	3	0.899	0.768	5
$M_{3-D_QSAR_Splitted}$_226	0.923	0.73	5	0.738	0.665	2
$M_{3-D_QSAR_Splitted}$_227	0.777	0.591	3	0.881	0.74	4
$M_{3-D_QSAR_Splitted}$_228	0.972	0.686	8	0.961	0.672	7
$M_{3-D_QSAR_Splitted}$_229	0.783	0.652	3	0.871	0.586	4
$M_{3-D_QSAR_Splitted}$_230	0.937	0.771	5	0.757	0.65	2
$M_{3-D_QSAR_Splitted}$_231	0.918	0.651	5	0.874	0.629	4
$M_{3-D_QSAR_Splitted}$_232	0.94	0.647	6	0.789	0.735	3
$M_{3-D_QSAR_Splitted}$_233	0.895	0.683	4	0.725	0.701	2
$M_{3-D_QSAR_Splitted}$_234	0.867	0.685	4	0.775	0.622	3
$M_{3-D_QSAR_Splitted}$_235	0.672	0.56	2	0.913	0.756	5
$M_{3-D_QSAR_Splitted}$_236	0.961	0.763	6	0.975	0.784	7
$M_{3-D_QSAR_Splitted}$_237	0.854	0.64	4	0.854	0.581	4
$M_{3-D_QSAR_Splitted}$_238	0.954	0.752	6	0.744	0.599	2
$M_{3-D_QSAR_Splitted}$_239	0.949	0.719	5	0.874	0.569	3
$M_{3-D_QSAR_Splitted}$_240	0.823	0.703	3	0.874	0.605	4
$M_{3-D_QSAR_Splitted}$_241	0.88	0.7	4	0.734	0.673	2
$M_{3-D_QSAR_Splitted}$_242	0.776	0.603	3	0.907	0.634	5
$M_{3-D_QSAR_Splitted}$_243	0.976	0.743	8	0.931	0.694	5
$M_{3-D_QSAR_Splitted}$_244	0.796	0.675	3	0.944	0.566	6
$M_{3-D_QSAR_Splitted}$_245	0.919	0.658	5	0.877	0.72	4
$M_{3-D_QSAR_Splitted}$_246	0.935	0.733	5	0.935	0.801	5
$M_{3-D_QSAR_Splitted}$_247	0.902	0.729	4	0.902	0.754	4
$M_{3-D_QSAR_Splitted}$_248	0.869	0.692	4	0.969	0.759	8
$M_{3-D_QSAR_Splitted}$_249	0.82	0.728	3	0.757	0.614	2
$M_{3-D_QSAR_Splitted}$_250	0.942	0.458	5	0.723	0.431	2
$M_{3-D_QSAR_Splitted}$_251	0.981	0.729	7	0.861	0.697	3
$M_{3-D_QSAR_Splitted}$_252	0.91	0.769	4	0.975	0.649	7
$M_{3-D_QSAR_Splitted}$_253	0.897	0.768	4	0.897	0.638	4
$M_{3-D_QSAR_Splitted}$_254	0.832	0.494	3	0.973	0.418	7
$M_{3-D_QSAR_Splitted}$_255	0.832	0.692	3	0.926	0.695	5
$M_{3-D_QSAR_Splitted}$_256	0.973	0.734	8	0.828	0.587	3
$M_{3-D_QSAR_Splitted}$_257	0.672	0.56	2	0.913	0.755	5
$M_{3-D_QSAR_Splitted}$_258	0.8	0.691	3	0.852	0.683	4
$M_{3-D_QSAR_Splitted}$_259	0.884	0.693	4	0.807	0.716	3
$M_{3-D_QSAR_Splitted}$_260	0.982	0.73	8	0.828	0.528	3
$M_{3-D_QSAR_Splitted}$_261	0.971	0.667	7	0.684	0.685	2
$M_{3-D_QSAR_Splitted}$_262	0.989	0.728	8	0.955	0.718	5
$M_{3-D_QSAR_Splitted}$_263	0.867	0.685	4	0.775	0.625	3
$M_{3-D_QSAR_Splitted}$_264	0.865	0.591	4	0.796	0.621	3
$M_{3-D_QSAR_Splitted}$_265	0.925	0.735	5	0.829	0.628	3
$M_{3-D_QSAR_Splitted}$_266	0.947	0.666	5	0.908	0.747	4
$M_{3-D_QSAR_Splitted}$_267	0.867	0.685	4	0.775	0.625	3
$M_{3-D_QSAR_Splitted}$_268	0.669	0.521	2	0.887	0.493	4
$M_{3-D_QSAR_Splitted}$_269	0.669	0.521	2	0.887	0.494	4

Table S26. 3-D QSAR models coefficients obtained with the Splitted dataset

Dataset ID	r^2_{cv}	q^2_{cv}	ONPCcv	r^2_{pred}	q^2_{pred}	ONPCpred
$M_{3-D_QSAR_Splitted}$_270	0.973	0.773	8	0.832	0.676	3
$M_{3-D_QSAR_Splitted}$_271	0.916	0.648	4	0.97	0.601	8
$M_{3-D_QSAR_Splitted}$_272	0.897	0.743	4	0.897	0.653	4
$M_{3-D_QSAR_Splitted}$_273	0.919	0.671	5	0.919	0.464	5
$M_{3-D_QSAR_Splitted}$_274	0.93	0.707	4	0.93	0.586	4
$M_{3-D_QSAR_Splitted}$_275	0.925	0.667	5	0.686	0.668	2
$M_{3-D_QSAR_Splitted}$_276	0.846	0.757	3	0.889	0.734	4
$M_{3-D_QSAR_Splitted}$_277	0.766	0.67	3	0.673	0.657	2
$M_{3-D_QSAR_Splitted}$_278	0.754	0.634	3	0.899	0.768	5
$M_{3-D_QSAR_Splitted}$_279	0.87	0.634	4	0.818	0.675	3
$M_{3-D_QSAR_Splitted}$_280	0.847	0.748	3	0.847	0.735	3
$M_{3-D_QSAR_Splitted}$_281	0.849	0.722	3	0.888	0.708	4
$M_{3-D_QSAR_Splitted}$_282	0.932	0.687	5	0.887	0.643	4
$M_{3-D_QSAR_Splitted}$_283	0.895	0.604	4	0.895	0.667	4
$M_{3-D_QSAR_Splitted}$_284	0.865	0.656	4	0.865	0.625	4
$M_{3-D_QSAR_Splitted}$_285	0.855	0.591	4	0.689	0.567	2
$M_{3-D_QSAR_Splitted}$_286	0.821	0.709	3	0.886	0.681	4
$M_{3-D_QSAR_Splitted}$_287	0.924	0.573	5	0.777	0.563	3
$M_{3-D_QSAR_Splitted}$_288	0.768	0.633	3	0.877	0.617	4
$M_{3-D_QSAR_Splitted}$_289	0.742	0.556	3	0.809	0.663	4
$M_{3-D_QSAR_Splitted}$_290	0.987	0.683	8	0.802	0.546	3
$M_{3-D_QSAR_Splitted}$_291	0.839	0.722	3	0.968	0.613	7
$M_{3-D_QSAR_Splitted}$_292	0.987	0.435	8	0.807	0.618	3
$M_{3-D_QSAR_Splitted}$_293	0.579	0.437	2	0.898	0.608	4
$M_{3-D_QSAR_Splitted}$_294	0.971	0.61	6	0.451	0.538	1
$M_{3-D_QSAR_Splitted}$_295	0.96	0.816	5	0.774	0.679	2
$M_{3-D_QSAR_Splitted}$_296	0.882	0.678	4	0.882	0.664	4
$M_{3-D_QSAR_Splitted}$_297	0.937	0.677	5	0.793	0.678	3
$M_{3-D_QSAR_Splitted}$_298	0.847	0.733	3	0.847	0.692	3
$M_{3-D_QSAR_Splitted}$_299	0.96	0.782	6	0.788	0.683	2
$M_{3-D_QSAR_Splitted}$_300	0.951	0.702	6	0.767	0.605	3
$M_{3-D_QSAR_Splitted}$_301	0.924	0.724	5	0.883	0.623	4
$M_{3-D_QSAR_Splitted}$_302	0.914	0.523	4	0.831	0.631	3
$M_{3-D_QSAR_Splitted}$_303	0.885	0.7	4	0.885	0.708	4
$M_{3-D_QSAR_Splitted}$_304	0.856	0.732	3	0.856	0.682	3
$M_{3-D_QSAR_Splitted}$_305	0.977	0.743	8	0.787	0.632	3
$M_{3-D_QSAR_Splitted}$_306	0.921	0.649	5	0.765	0.691	3
$M_{3-D_QSAR_Splitted}$_307	0.884	0.727	4	0.884	0.654	4
$M_{3-D_QSAR_Splitted}$_308	0.975	0.741	8	0.93	0.728	5
$M_{3-D_QSAR_Splitted}$_309	0.847	0.675	4	0.847	0.565	4
$M_{3-D_QSAR_Splitted}$_310	0.954	0.721	6	0.93	0.676	5
$M_{3-D_QSAR_Splitted}$_311	0.98	0.758	8	0.824	0.664	3
$M_{3-D_QSAR_Splitted}$_312	0.98	0.758	8	0.824	0.664	3
$M_{3-D_QSAR_Splitted}$_313	0.861	0.676	4	0.784	0.687	3
$M_{3-D_QSAR_Splitted}$_314	0.963	0.738	6	0.724	0.589	2
$M_{3-D_QSAR_Splitted}$_315	0.917	0.676	5	0.836	0.696	3
$M_{3-D_QSAR_Splitted}$_316	0.975	0.773	7	0.827	0.6	3
$M_{3-D_QSAR_Splitted}$_317	0.766	0.696	2	0.903	0.635	4
$M_{3-D_QSAR_Splitted}$_318	0.977	0.743	8	0.787	0.632	3
$M_{3-D_QSAR_Splitted}$_319	0.964	0.722	7	0.804	0.688	3
$M_{3-D_QSAR_Splitted}$_320	0.905	0.752	4	0.856	0.647	3
$M_{3-D_QSAR_Splitted}$_321	0.959	0.71	6	0.891	0.618	4
$M_{3-D_QSAR_Splitted}$_322	0.981	0.76	8	0.889	0.645	4
$M_{3-D_QSAR_Splitted}$_323	0.861	0.676	4	0.784	0.68	3

Table S26. 3-D QSAR models coefficients obtained with the Splitted dataset

Dataset ID	r^2_{cv}	q^2_{cv}	$ONPC_{cv}$	r^2_{pred}	q^2_{pred}	$ONPC_{pred}$
$M_{3\text{-}D_QSAR_Splitted}$_324	0.944	0.625	6	0.879	0.671	4
$M_{3\text{-}D_QSAR_Splitted}$_325	0.827	0.618	4	0.88	0.612	5
$M_{3\text{-}D_QSAR_Splitted}$_326	0.989	0.603	8	0.938	0.645	5
$M_{3\text{-}D_QSAR_Splitted}$_327	0.98	0.768	8	0.924	0.585	5
$M_{3\text{-}D_QSAR_Splitted}$_328	0.944	0.625	6	0.879	0.672	4
$M_{3\text{-}D_QSAR_Splitted}$_329	0.952	0.695	6	0.934	0.725	5
$M_{3\text{-}D_QSAR_Splitted}$_330	0.971	0.768	6	0.925	0.65	4
$M_{3\text{-}D_QSAR_Splitted}$_331	0.987	0.73	8	0.723	0.649	2
$M_{3\text{-}D_QSAR_Splitted}$_332	0.849	0.738	3	0.981	0.687	8
$M_{3\text{-}D_QSAR_Splitted}$_333	0.843	0.727	3	0.843	0.68	3
$M_{3\text{-}D_QSAR_Splitted}$_334	0.887	0.715	4	0.922	0.656	5
$M_{3\text{-}D_QSAR_Splitted}$_335	0.98	0.768	8	0.924	0.585	5
$M_{3\text{-}D_QSAR_Splitted}$_336	0.936	0.653	5	0.936	0.699	5
$M_{3\text{-}D_QSAR_Splitted}$_337	0.887	0.715	4	0.922	0.658	5
$M_{3\text{-}D_QSAR_Splitted}$_338	0.919	0.753	4	0.949	0.563	5
$M_{3\text{-}D_QSAR_Splitted}$_339	0.884	0.73	4	0.937	0.605	5
$M_{3\text{-}D_QSAR_Splitted}$_340	0.953	0.7	6	0.801	0.605	3
$M_{3\text{-}D_QSAR_Splitted}$_341	0.965	0.469	6	0.965	0.636	6
$M_{3\text{-}D_QSAR_Splitted}$_342	0.895	0.709	4	0.928	0.703	5
$M_{3\text{-}D_QSAR_Splitted}$_343	0.719	0.656	2	0.826	0.612	3
$M_{3\text{-}D_QSAR_Splitted}$_344	0.893	0.622	4	0.893	0.719	4
$M_{3\text{-}D_QSAR_Splitted}$_345	0.751	0.69	2	0.901	0.49	4
$M_{3\text{-}D_QSAR_Splitted}$_346	0.994	0.449	8	0.422	0.524	1
$M_{3\text{-}D_QSAR_Splitted}$_347	0.821	0.603	3	0.984	0.624	8
$M_{3\text{-}D_QSAR_Splitted}$_348	0.973	0.798	6	0.86	0.591	3
$M_{3\text{-}D_QSAR_Splitted}$_349	0.852	0.744	3	0.852	0.627	3
$M_{3\text{-}D_QSAR_Splitted}$_350	0.794	0.655	3	0.868	0.623	4
$M_{3\text{-}D_QSAR_Splitted}$_351	0.905	0.761	4	0.905	0.697	4
$M_{3\text{-}D_QSAR_Splitted}$_352	0.668	0.548	2	0.978	0.619	8
$M_{3\text{-}D_QSAR_Splitted}$_353	0.719	0.603	3	0.919	0.643	6
$M_{3\text{-}D_QSAR_Splitted}$_354	0.827	0.728	3	0.827	0.667	3
$M_{3\text{-}D_QSAR_Splitted}$_355	0.882	0.696	4	0.938	0.609	6
$M_{3\text{-}D_QSAR_Splitted}$_356	0.967	0.512	6	0.942	0.529	5
$M_{3\text{-}D_QSAR_Splitted}$_357	0.891	0.664	4	0.891	0.639	4
$M_{3\text{-}D_QSAR_Splitted}$_358	0.952	0.65	6	0.934	0.763	5
$M_{3\text{-}D_QSAR_Splitted}$_359	0.992	0.781	8	0.992	0.61	8
$M_{3\text{-}D_QSAR_Splitted}$_360	0.863	0.717	4	0.863	0.742	4
$M_{3\text{-}D_QSAR_Splitted}$_361	0.878	0.639	4	0.818	0.676	3
$M_{3\text{-}D_QSAR_Splitted}$_362	0.962	0.694	6	0.973	0.642	7
$M_{3\text{-}D_QSAR_Splitted}$_363	0.827	0.728	3	0.827	0.668	3
$M_{3\text{-}D_QSAR_Splitted}$_364	0.979	0.655	7	0.968	0.524	6
$M_{3\text{-}D_QSAR_Splitted}$_365	0.841	0.722	3	0.841	0.718	3
$M_{3\text{-}D_QSAR_Splitted}$_366	0.827	0.728	3	0.827	0.669	3
$M_{3\text{-}D_QSAR_Splitted}$_367	0.863	0.771	3	0.863	0.774	3
$M_{3\text{-}D_QSAR_Splitted}$_368	0.818	0.676	3	0.935	0.627	5
$M_{3\text{-}D_QSAR_Splitted}$_369	0.9	0.639	5	0.635	0.547	2
$M_{3\text{-}D_QSAR_Splitted}$_370	0.99	0.73	8	0.9	0.696	4
$M_{3\text{-}D_QSAR_Splitted}$_371	0.882	0.696	4	0.938	0.609	6
$M_{3\text{-}D_QSAR_Splitted}$_372	0.9	0.639	5	0.635	0.546	2
$M_{3\text{-}D_QSAR_Splitted}$_373	0.863	0.717	4	0.863	0.742	4
$M_{3\text{-}D_QSAR_Splitted}$_374	0.897	0.745	4	0.86	0.654	3
$M_{3\text{-}D_QSAR_Splitted}$_375	0.891	0.664	4	0.891	0.637	4
$M_{3\text{-}D_QSAR_Splitted}$_376	0.837	0.672	3	0.979	0.664	8
$M_{3\text{-}D_QSAR_Splitted}$_377	0.885	0.636	4	0.916	0.695	5

Table S26. 3-D QSAR models coefficients obtained with the Splitted dataset

Dataset ID	$r^2{}_{cv}$	$q^2{}_{cv}$	$ONPC_{cv}$	$r^2{}_{pred}$	$q^2{}_{pred}$	$ONPC_{pred}$
$M_{3\text{-}D_QSAR_Splitted}$_378	0.825	0.588	3	0.915	0.569	5
$M_{3\text{-}D_QSAR_Splitted}$_379	0.861	0.72	3	0.443	0.476	1
$M_{3\text{-}D_QSAR_Splitted}$_380	0.979	0.521	7	0.799	0.422	3
$M_{3\text{-}D_QSAR_Splitted}$_381	0.942	0.729	5	0.978	0.773	8
$M_{3\text{-}D_QSAR_Splitted}$_382	0.884	0.685	4	0.804	0.586	3
$M_{3\text{-}D_QSAR_Splitted}$_383	0.975	0.688	8	0.727	0.587	2
$M_{3\text{-}D_QSAR_Splitted}$_384	0.814	0.633	3	0.814	0.647	3
$M_{3\text{-}D_QSAR_Splitted}$_385	0.842	0.733	3	0.842	0.522	3
$M_{3\text{-}D_QSAR_Splitted}$_386	0.87	0.64	4	0.87	0.639	4
$M_{3\text{-}D_QSAR_Splitted}$_387	0.903	0.779	4	0.903	0.646	4
$M_{3\text{-}D_QSAR_Splitted}$_388	0.945	0.6	6	0.911	0.619	5
$M_{3\text{-}D_QSAR_Splitted}$_389	0.965	0.694	7	0.93	0.616	5
$M_{3\text{-}D_QSAR_Splitted}$_390	0.952	0.786	6	0.875	0.732	3
$M_{3\text{-}D_QSAR_Splitted}$_391	0.839	0.672	4	0.907	0.661	5
$M_{3\text{-}D_QSAR_Splitted}$_392	0.75	0.527	3	0.962	0.745	7
$M_{3\text{-}D_QSAR_Splitted}$_393	0.562	0.436	2	0.768	0.628	3
$M_{3\text{-}D_QSAR_Splitted}$_394	0.883	0.692	4	0.838	0.71	3
$M_{3\text{-}D_QSAR_Splitted}$_395	0.739	0.493	3	0.589	0.598	2
$M_{3\text{-}D_QSAR_Splitted}$_396	0.873	0.649	4	0.873	0.642	4
$M_{3\text{-}D_QSAR_Splitted}$_397	0.932	0.708	5	0.777	0.579	3
$M_{3\text{-}D_QSAR_Splitted}$_398	0.984	0.732	7	0.719	0.613	2
$M_{3\text{-}D_QSAR_Splitted}$_399	0.887	0.731	4	0.887	0.688	4
$M_{3\text{-}D_QSAR_Splitted}$_400	0.938	0.797	5	0.766	0.709	2
$M_{3\text{-}D_QSAR_Splitted}$_401	0.954	0.813	5	0.864	0.681	3
$M_{3\text{-}D_QSAR_Splitted}$_402	0.954	0.709	6	0.916	0.639	5
$M_{3\text{-}D_QSAR_Splitted}$_403	0.804	0.687	3	0.804	0.675	3
$M_{3\text{-}D_QSAR_Splitted}$_404	0.952	0.772	6	0.93	0.684	5
$M_{3\text{-}D_QSAR_Splitted}$_405	0.963	0.749	6	0.772	0.703	2
$M_{3\text{-}D_QSAR_Splitted}$_406	0.927	0.715	5	0.848	0.568	3
$M_{3\text{-}D_QSAR_Splitted}$_407	0.779	0.68	3	0.872	0.635	4
$M_{3\text{-}D_QSAR_Splitted}$_408	0.958	0.783	6	0.869	0.673	3
$M_{3\text{-}D_QSAR_Splitted}$_409	0.967	0.793	6	0.845	0.594	3
$M_{3\text{-}D_QSAR_Splitted}$_410	0.89	0.736	4	0.89	0.674	4
$M_{3\text{-}D_QSAR_Splitted}$_411	0.965	0.578	7	0.869	0.551	4
$M_{3\text{-}D_QSAR_Splitted}$_412	0.938	0.791	5	0.813	0.661	3
$M_{3\text{-}D_QSAR_Splitted}$_413	0.904	0.7	5	0.868	0.739	4
$M_{3\text{-}D_QSAR_Splitted}$_414	0.882	0.688	4	0.816	0.587	3
$M_{3\text{-}D_QSAR_Splitted}$_415	0.903	0.592	5	0.85	0.605	4
$M_{3\text{-}D_QSAR_Splitted}$_416	0.961	0.703	6	0.782	0.583	3
$M_{3\text{-}D_QSAR_Splitted}$_417	0.869	0.811	3	0.933	0.716	5
$M_{3\text{-}D_QSAR_Splitted}$_418	0.954	0.711	6	0.832	0.57	3
$M_{3\text{-}D_QSAR_Splitted}$_419	0.986	0.768	8	0.979	0.681	7
$M_{3\text{-}D_QSAR_Splitted}$_420	0.95	0.719	6	0.779	0.653	2
$M_{3\text{-}D_QSAR_Splitted}$_421	0.801	0.646	3	0.679	0.653	2
$M_{3\text{-}D_QSAR_Splitted}$_422	0.95	0.719	6	0.779	0.655	2
$M_{3\text{-}D_QSAR_Splitted}$_423	0.956	0.723	6	0.801	0.615	3
$M_{3\text{-}D_QSAR_Splitted}$_424	0.779	0.68	3	0.872	0.633	4
$M_{3\text{-}D_QSAR_Splitted}$_425	0.764	0.649	3	0.927	0.667	6
$M_{3\text{-}D_QSAR_Splitted}$_426	0.947	0.714	6	0.913	0.781	5
$M_{3\text{-}D_QSAR_Splitted}$_427	0.873	0.649	4	0.873	0.641	4
$M_{3\text{-}D_QSAR_Splitted}$_428	0.821	0.692	3	0.821	0.685	3
$M_{3\text{-}D_QSAR_Splitted}$_429	0.944	0.72	5	0.859	0.605	3
$M_{3\text{-}D_QSAR_Splitted}$_430	0.986	0.768	8	0.979	0.683	7
$M_{3\text{-}D_QSAR_Splitted}$_431	0.831	0.623	3	0.873	0.566	4

Table S26. 3-D QSAR models coefficients obtained with the Splitted dataset

Dataset ID	r^2_{cv}	q^2_{cv}	$ONPC_{cv}$	r^2_{pred}	q^2_{pred}	$ONPC_{pred}$
$M_{3\text{-}D\_QSAR\_Splitted\_}432$	0.954	0.709	6	0.916	0.642	5
$M_{3\text{-}D\_QSAR\_Splitted\_}433$	0.975	0.688	8	0.727	0.591	2
$M_{3\text{-}D\_QSAR\_Splitted\_}434$	0.94	0.736	5	0.74	0.611	2
$M_{3\text{-}D\_QSAR\_Splitted\_}435$	0.869	0.811	3	0.933	0.716	5

Table S27. Details for $M_{3\text{-}D\_QSAR\_Splitted\_170}$ model

PC	r^2	SDEC	q^2_{cv}	$SDEP_{cv}$	q^2_{pred}	$SDEP_{pred}$
1	0.36	0.68	0.33	0.70	0.47	0.65
2	0.64	0.51	0.57	0.56	0.65	0.53
3	0.79	0.39	0.64	0.51	0.71	0.48
4	0.86	0.32	0.64	0.51	0.76	0.44
5*	0.92	0.24	0.64	0.52	0.82	0.38
6	0.94	0.21	0.62	0.53	0.82	0.38
7	0.96	0.17	0.61	0.54	0.82	0.38
8	0.97	0.14	0.57	0.56	0.81	0.39

The star indicate the optimal number of PCs selected by a the max q^2_{pred} value.

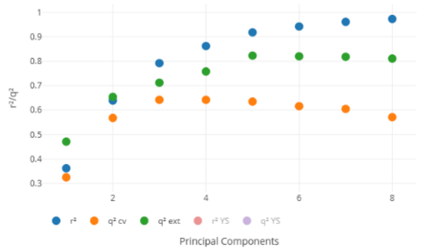

A

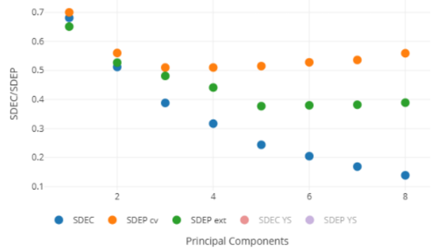

B

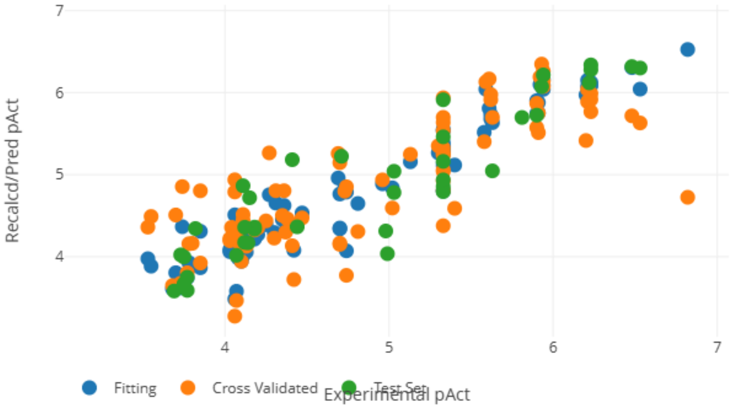

C

Figure S8. Full dataset $M_{3\text{-}D\_QSAR\_Splitted\_170}$ model: (A) r^2, q^2_{cv} and q^2_{pred} values in relation to number of PCs. (B) SDEC, $SDEP_{cv}$ and $SDEP_{pred}$ in relation with the number of PC and the (C) experimental versus the recalculated/CV-predicted/Test Set predicted pMIC (pAct) values at 4 PCs. In blue, the r^2, SDEC, and experimental fitted values. In orange, the q^2_{cv}, $SDEP_{cv,}$ and crossvalidated values. In green the q^2_{pred}, $SDEP_{pred}$ and Test Set predicted values.

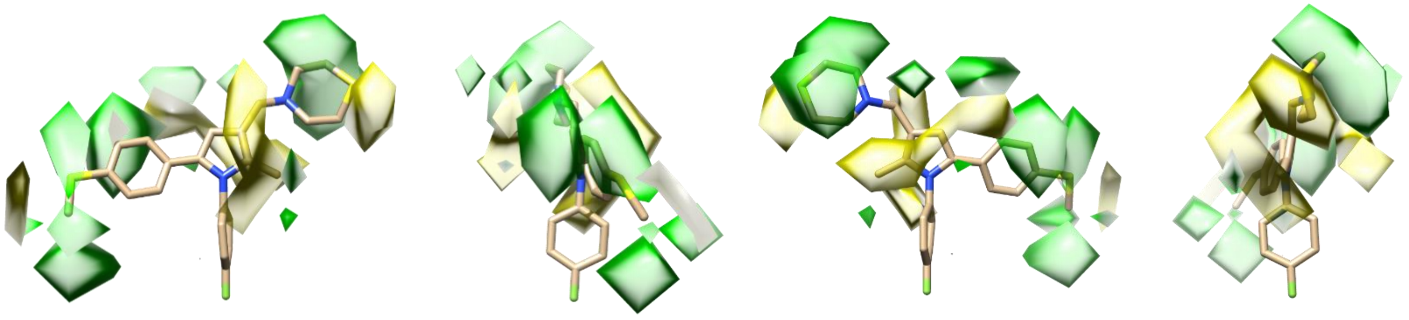

A B C D

Average Activity Contribution (AAC) Plots for the STE field

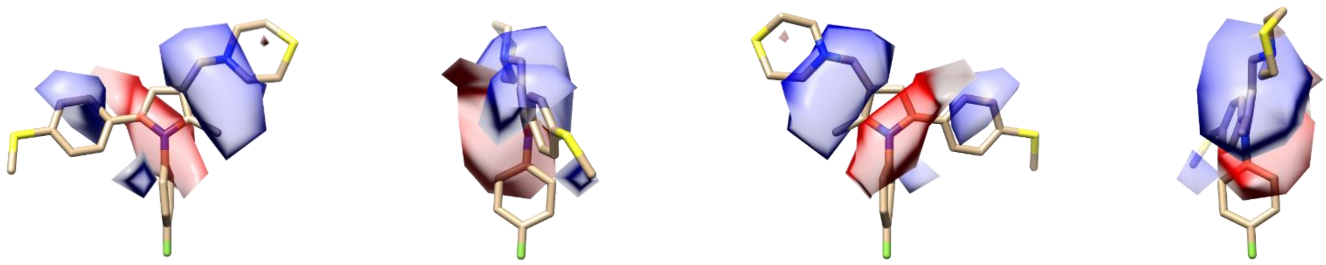

A B C D

Average Activity Contribution (AAC) Plots for the ELE field

Figure S9. AAC plots relative to the $M_{3\text{-}D\_QSAR\_Splitted\_170}$ model. Four images are given for the STE and ELE fields. From left to right each image is a 90° rotated view of the same object. BMC2010_12, the most potent compound of the dataset, is depicted along with the AAC. Images were generated with the 1.18 version of UCSF Chimera software.

CoMFA map analysis of the $M_{3\text{-}D\_QSAR\_Splitted\_170}$ model

The CoMFA maps, both electrostatic (ELE) and steric (STE), provide critical insights into how the most potent molecule interacts with its environment. By analyzing both maps together, we can identify regions where structural modifications can enhance activity while avoiding detrimental effects.

The thiomorpholine sulfur atom is near red regions, indicating that introducing negatively charged groups could reduce activity. This suggests that the sulfur atom’s partial negative charge may contribute negatively to electrostatic interactions if not balanced. Furthermore, the sulfur atom is near STE negative yellow regions, suggesting that replacing it with bulky groups would lead to steric hindrance with negative effect to the potency. It could be interesting a classical isosteric replcement with nitrogen, oxygen or a methylene. The sulfur atom presents a challenge due to its proximity to unfavorable regions in both maps. While it may contribute to other interactions (e.g., hydrogen bonding), its presence requires careful management. Avoiding bulky or highly electronegative groups near the sulfur atom is crucial to prevent reducing activity through either electrostatic repulsion or steric hindrance.

Blue regions are aligned with the thiomorpholine nitrogen atom, indicating that a positive charged group enhances activity. This suggests that the nitrogen atom could play a crucial role in favorable electrostatic interactions.

For the STE, the nitrogen atom is also positioned within green regions, showing that its bulkiness and the atom attache to it contributes positively to steric interactions. This indicates that the size and shape ot the atoms’ aggregate around the thiomorpholine nitrogen are well-suited for a positive contribute to the activity. Thus, the nitrogen atom is a dual-functional feature, contributing positively to both electrostatic and steric interactions. Its alignment with favorable regions in both maps underscores its importance for maintaining high potency. Future modifications should aim to preserve or enhance this region by introducing substituents that maintain or increase its positive charge and bulk.

The aromatic ring attached at pyrrole position 5 align with blue regions, suggesting that its electron density enhances electrostatic interactions. This indicates that the aromatic system contributes positively to stabilizing interactions. Moreover the phenyl moiety is close to green polyhedra, indicating that its bulk is important for the antitubercular activity. The C5-pyrrole aromatic ring seems strategically positioned and extending bulky

substituents (e.g., phenyl, cyclohexyl) or electron-rich groups (e.g., methoxy, amino) from these regions could further improve activity by leveraging both types of interactions.
The N1-Pyrrole phenyl group is near red regions, indicating that placing charged groups in this area could reduce activity, thus the insertion of substituent on this aromatic moiety should be selected carefully. This part of the molecule is also near yellow regions, suggesting that introducing bulky groups in this area would reduce activity.

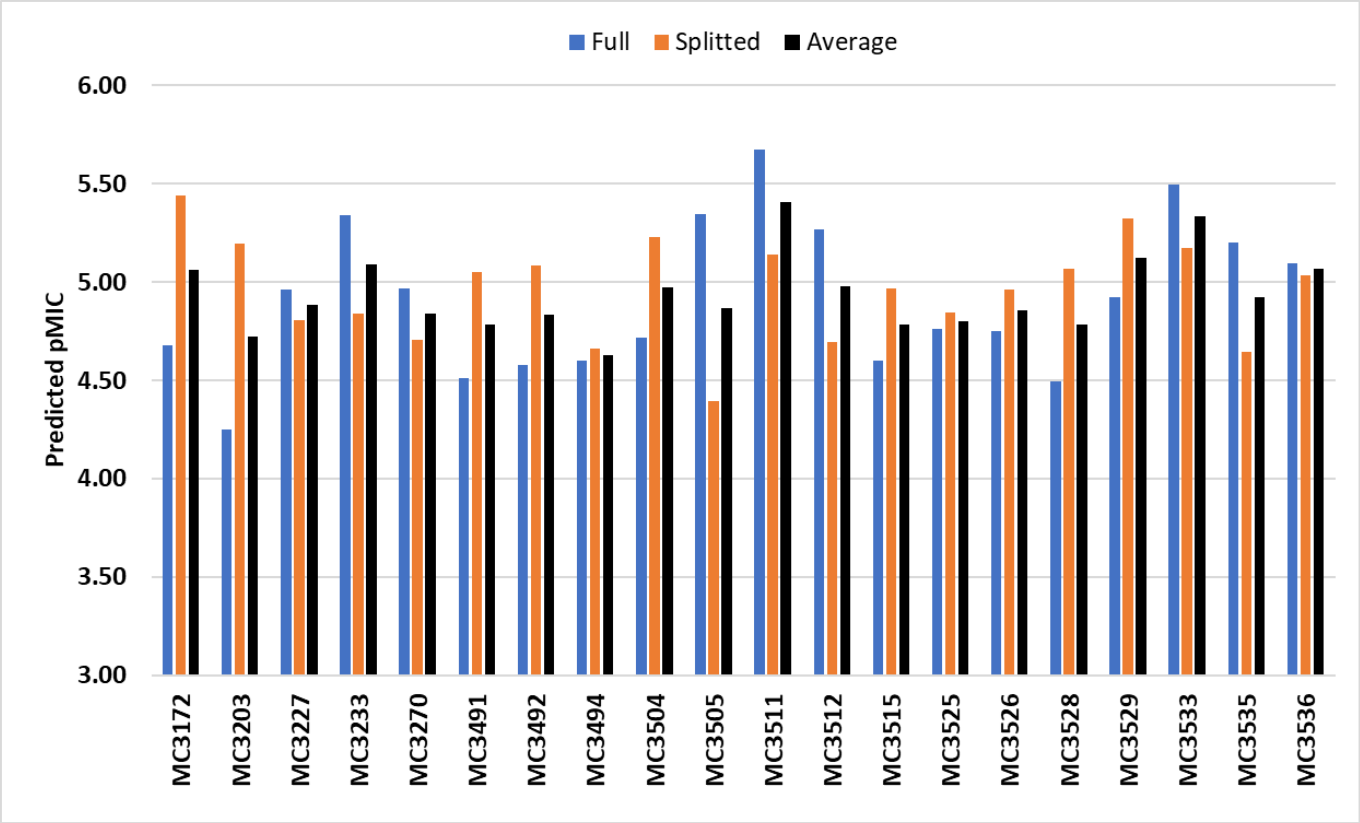

C

Figure S10. pMIC prediction on the under synthesizing compounds with the two final Py-CoMFA models.

Table S28. Re-Docking and Cross-Docking RMSD values.

Docking Program	Scoring Function	Experimental Conformation Re-Docking (ECRD)								
		6AJG	6AJH	6AJI	6AJJ	7C2M	7C2N	7WNX	ADA %	Average
plants	chemplp	0.99	0.42	2.02	1.74	0.40	0.91	1.02	92.86	1.07
	PLP95	1.39	0.40	1.78	1.73	0.70	0.90	1.93	100.00	1.26
	PLP	1.39	0.42	1.99	1.74	0.40	0.91	1.42	100.00	1.18
smina	AD4	9.72	7.80	0.62	2.35	4.66	0.54	1.64	50.00	3.90
	Vina	2.34	2.24	0.61	2.27	1.15	0.43	1.10	78.57	1.45
	Vinardo	2.32	1.44	0.51	2.26	1.18	0.42	0.88	85.71	1.29
		**Random Conformation Re-Docking (RCRD)**								
		6AJG	6AJH	6AJI	6AJJ	7C2M	7C2N	7WNX	ADA %	Average
plants	chemplp	1.59	0.48	1.20	2.22	0.83	1.84	1.54	92.86	1.39
	PLP95	2.21	0.48	1.22	2.16	1.02	1.81	1.97	85.71	1.55
	PLP	1.70	0.48	1.30	2.62	0.86	2.43	1.85	85.71	1.61
smina	AD4	9.72	10.12	1.48	2.52	4.70	2.48	1.67	42.86	4.67
	Vina	2.36	2.21	0.64	2.58	1.14	2.47	1.41	71.43	1.83
	Vinardo	2.38	1.25	0.55	2.46	1.20	1.78	1.40	85.71	1.57
		**Experimental Conformation Cross-Docking (ECCD)**								
		6AJG	6AJH	6AJI	6AJJ	7C2M	7C2N	7WNX	ADA %	Average
plants	chemplp	1.14	1.08	1.61	1.75	0.50	1.64	3.05	85.71	1.54
	PLP95	2.01	1.12	1.87	1.73	1.84	1.55	2.96	85.71	1.87
	PLP	1.73	1.10	1.81	1.74	1.84	1.10	3.03	85.71	1.76
smina	AD4	2.30	2.97	1.48	2.28	2.43	1.39	3.13	57.14	2.28
	Vina	2.25	2.82	1.84	2.25	1.34	1.00	2.89	71.43	2.06
	Vinardo	2.21	2.71	1.29	2.25	1.51	0.90	2.85	71.43	1.96
		**Random Conformation Cross-Docking (RCCD)**								
		6AJG	6AJH	6AJI	6AJJ	7C2M	7C2N	7WNX	ADA %	Average
plants	chemplp	1.84	1.10	7.81	2.80	1.54	2.40	2.95	64.29	2.92
	PLP95	1.87	1.01	1.37	2.74	1.90	1.83	2.75	85.71	1.92
	PLP	1.85	1.09	1.87	2.74	1.67	2.50	3.24	71.43	2.14
smina	AD4	1.96	1.44	1.33	4.48	2.43	1.85	3.22	64.29	2.39
	Vina	2.30	2.73	1.36	2.84	1.37	2.53	2.86	64.29	2.28
	Vinardo	1.96	1.23	1.35	2.50	1.50	1.85	2.84	85.71	1.89

Table S29. SB 3-D QSAR models for the ligands extracted from the aligned complexes with MmpL3 protein.

Model ID	r^2	q^2cv	ONPC	Probe	Charge Model	Min Sigma	Grid Spacing	Grid Extension	Diel Const	CutOff	# Level
$M_{SB_3\text{-}D_QSAR}$_1	0.99	0.52	3	C.3	gasteiger	2.00	2	5	8	30	0
$M_{SB_3\text{-}D_QSAR}$_2	0.95	0.42	2	N.4	qeq	0.40	2.1	5	6	11	3
$M_{SB_3\text{-}D_QSAR}$_3	1.00	0.46	4	C.3.H3	gasteiger	1.10	1.8	5	41	19	0
$M_{SB_3\text{-}D_QSAR}$_4	0.96	0.34	2	I	eem	1.70	1.4	7	4	4	3
$M_{SB_3\text{-}D_QSAR}$_5	0.99	0.62	2	OH2	eem2015hn	2.60	1.4	8	40	13	3
$M_{SB_3\text{-}D_QSAR}$_6	0.96	0.36	2	H.P	eem2015ha	0.90	2.5	3	59	24	1
$M_{SB_3\text{-}D_QSAR}$_7	0.93	0.33	2	O.3	eem2015hn	2.30	3.4	4	7	19	4
$M_{SB_3\text{-}D_QSAR}$_8	1.00	0.41	3	F	qtpie	1.90	1.1	9	10	29	2
$M_{SB_3\text{-}D_QSAR}$_9	0.96	0.46	2	OH	eem2015ha	0.80	1.4	4	9	48	3
$M_{SB_3\text{-}D_QSAR}$_10	0.99	0.47	3	H.P	openbabel	0.80	1.3	9	50	32	4
$M_{SB_3\text{-}D_QSAR}$_11	1.00	0.52	4	O.3	mmff94	1.60	2.3	4	64	18	2
$M_{SB_3\text{-}D_QSAR}$_12	0.96	0.47	2	C.3.H3	eem2015bn	1.50	1.6	9	64	38	0
$M_{SB_3\text{-}D_QSAR}$_13	0.99	0.42	3	OH2	eem2015hn	1.20	2.9	6	42	43	1
$M_{SB_3\text{-}D_QSAR}$_14	0.00	-0.36	1	OH	openbabel	1.70	1	9	64	1	2
$M_{SB_3\text{-}D_QSAR}$_15	1.00	0.26	5	I	qeq	2.40	2.9	5	69	30	2
$M_{SB_3\text{-}D_QSAR}$_16	0.99	0.44	3	OH	eem2015hn	1.10	1	7	62	26	2
$M_{SB_3\text{-}D_QSAR}$_17	0.15	-0.98	1	H.P	eem	1.80	1.7	8	33	1	2
$M_{SB_3\text{-}D_QSAR}$_18	0.97	0.19	2	O.3	qeq	2.40	1.2	8	1	25	1
$M_{SB_3\text{-}D_QSAR}$_19	1.00	0.48	3	O.3	eem2015ha	0.70	1.4	5	49	13	1
$M_{SB_3\text{-}D_QSAR}$_20	0.95	0.51	2	S.o	eem2015ha	1.90	2.1	5	47	44	3
$M_{SB_3\text{-}D_QSAR}$_21	1.00	0.46	3	N.4	mmff94	0.80	2.9	4	61	39	0
$M_{SB_3\text{-}D_QSAR}$_22	0.97	0.43	2	Cl	eem2015bn	0.10	3.1	6	66	50	1
$M_{SB_3\text{-}D_QSAR}$_23	1.00	0.53	4	C.3.H2	eem2015ha	0.30	3.2	7	32	10	1
$M_{SB_3\text{-}D_QSAR}$_24	1.00	0.61	3	OH2	eem	2.30	2.4	8	76	41	1
$M_{SB_3\text{-}D_QSAR}$_25	0.99	0.48	3	C.3.H3	eem2015hn	0.80	1.9	10	23	13	0
$M_{SB_3\text{-}D_QSAR}$_26	0.98	0.56	3	F	openbabel	0.90	2.8	5	37	21	2
$M_{SB_3\text{-}D_QSAR}$_27	0.99	0.53	3	C.3	mmff94	1.30	1.5	8	26	42	4
$M_{SB_3\text{-}D_QSAR}$_28	0.99	0.37	3	S.3	eem2015ha	2.40	2.6	9	75	17	1
$M_{SB_3\text{-}D_QSAR}$_29	0.94	0.44	2	I	mmff94	1.50	2.9	9	24	25	2
$M_{SB_3\text{-}D_QSAR}$_30	0.99	0.51	3	Br	eem	1.90	3.1	10	24	41	0
$M_{SB_3\text{-}D_QSAR}$_31	1.00	0.48	6	N.4	eem2015bm	1.20	1.8	8	38	50	4
$M_{SB_3\text{-}D_QSAR}$_32	0.99	0.48	3	C.3.H2	eem2015hn	1.10	1.5	3	40	49	3
$M_{SB_3\text{-}D_QSAR}$_33	1.00	0.47	4	C.3	eem2015hm	0.70	3.3	7	46	7	2
$M_{SB_3\text{-}D_QSAR}$_34	0.92	0.49	2	OH2	eem2015ha	0.50	3.3	4	29	44	2
$M_{SB_3\text{-}D_QSAR}$_35	0.97	0.39	2	OH	eem2015hm	1.10	2.3	4	11	38	0
$M_{SB_3\text{-}D_QSAR}$_36	0.97	0.38	2	C.3.H1	eem2015bm	2.20	1.3	4	56	25	1
$M_{SB_3\text{-}D_QSAR}$_37	0.99	0.54	3	C.3.H3	eem	1.90	2.7	4	77	28	1
$M_{SB_3\text{-}D_QSAR}$_38	1.00	0.44	3	C.3	eem2015ba	1.20	2.3	6	75	15	1
$M_{SB_3\text{-}D_QSAR}$_39	1.00	0.53	2	Na	qtpie	1.00	1.7	3	75	4	0
$M_{SB_3\text{-}D_QSAR}$_40	0.97	0.48	2	OH	eem2015ba	2.60	1.9	5	41	32	3
$M_{SB_3\text{-}D_QSAR}$_41	0.99	0.42	3	Na	eem2015hn	1.30	1.3	8	73	49	2
$M_{SB_3\text{-}D_QSAR}$_42	1.00	0.51	4	S.3	eem2015hm	1.30	2.8	3	18	38	2
$M_{SB_3\text{-}D_QSAR}$_43	1.00	0.48	7	S.o	eem2015ha	0.10	2.2	4	69	17	2
$M_{SB_3\text{-}D_QSAR}$_44	0.96	0.47	2	O.3	eem2015bn	1.20	2.6	7	79	19	3
$M_{SB_3\text{-}D_QSAR}$_45	0.99	0.43	3	F	eem2015hn	1.00	1	8	23	29	0
$M_{SB_3\text{-}D_QSAR}$_46	0.97	0.49	2	C.3.H3	eem2015bn	2.80	2.4	5	73	20	0
$M_{SB_3\text{-}D_QSAR}$_47	0.97	0.37	2	O.3	eem	0.40	3	6	34	16	0
$M_{SB_3\text{-}D_QSAR}$_48	1.00	0.54	3	OH2	eem2015bn	2.10	1.5	5	16	11	1
$M_{SB_3\text{-}D_QSAR}$_49	1.00	0.40	3	O.3	eem2015hm	2.60	1	7	33	33	4
$M_{SB_3\text{-}D_QSAR}$_50	1.00	0.59	8	F	eem2015bn	2.80	2.7	3	50	25	4
$M_{SB_3\text{-}D_QSAR}$_51	0.99	0.44	3	H	eem2015ba	1.70	1.4	10	13	50	0
$M_{SB_3\text{-}D_QSAR}$_52	0.99	0.43	3	OH2	eem2015ba	0.80	2.2	3	4	42	2
$M_{SB_3\text{-}D_QSAR}$_53	1.00	0.53	4	H.P	eem2015hm	0.80	2.8	5	68	47	4
$M_{SB_3\text{-}D_QSAR}$_54	0.99	0.50	3	OH	eem	2.40	1.3	3	22	37	2
$M_{SB_3\text{-}D_QSAR}$_55	0.99	0.57	3	OH	gasteiger	1.40	3.4	7	37	39	2
$M_{SB_3\text{-}D_QSAR}$_56	0.96	0.33	2	H	qeq	1.00	3.2	10	45	13	2
$M_{SB_3\text{-}D_QSAR}$_57	1.00	0.26	3	C.3.H1	qtpie	2.00	2.4	6	12	49	0
$M_{SB_3\text{-}D_QSAR}$_58	0.99	0.47	3	N.4	eem2015ha	0.70	1	8	43	32	2
$M_{SB_3\text{-}D_QSAR}$_59	0.99	0.73	2	C.3.H3	gasteiger	2.30	1.4	4	9	3	4

Table S29. SB 3-D QSAR models for the ligands extracted from the aligned complexes with MmpL3 protein.

Model ID	r^2	q^2cv	ONPC	Probe	Charge Model	Min Sigma	Grid Spacing	Grid Extension	Diel Const	CutOff	# Level
$M_{SB_3\text{-}D_QSAR}$_60	1.00	0.63	5	C.3	openbabel	1.50	3.4	8	23	40	4
$M_{SB_3\text{-}D_QSAR}$_61	1.00	0.41	3	H	eem2015bn	0.80	1	10	44	33	3
$M_{SB_3\text{-}D_QSAR}$_62	0.96	0.51	2	OH	qtpie	1.60	3.5	7	6	10	1
$M_{SB_3\text{-}D_QSAR}$_63	0.99	0.43	3	F	mmff94	2.00	1.5	3	67	17	1
$M_{SB_3\text{-}D_QSAR}$_64	0.97	0.46	2	Na	eem2015bm	0.10	2.2	10	18	17	4
$M_{SB_3\text{-}D_QSAR}$_65	0.98	0.52	3	N.4	eem2015bn	1.00	2.5	9	75	39	0
$M_{SB_3\text{-}D_QSAR}$_66	1.00	0.46	5	F	gasteiger	2.10	2.5	8	65	28	4
$M_{SB_3\text{-}D_QSAR}$_67	0.99	0.31	3	I	eem2015bn	0.30	2.6	3	23	47	0
$M_{SB_3\text{-}D_QSAR}$_68	0.99	0.43	3	C.3	eem	1.40	2.2	8	22	25	0
$M_{SB_3\text{-}D_QSAR}$_69	1.00	0.73	5	Na	eem2015bm	3.00	3.1	5	13	10	0
$M_{SB_3\text{-}D_QSAR}$_70	0.98	0.29	3	C.3.H1	eem2015ba	0.90	2.6	9	19	41	2
$M_{SB_3\text{-}D_QSAR}$_71	1.00	0.53	3	I	eem2015bm	1.80	2	3	60	8	1
$M_{SB_3\text{-}D_QSAR}$_72	1.00	0.38	3	H.P	eem2015bm	0.60	1	10	70	18	4
$M_{SB_3\text{-}D_QSAR}$_73	0.99	0.53	3	C.3.H2	eem	2.60	2	6	19	49	4
$M_{SB_3\text{-}D_QSAR}$_74	0.97	0.43	2	I	eem2015hn	0.20	3.4	3	4	19	1
$M_{SB_3\text{-}D_QSAR}$_75	0.95	0.28	2	H	eem2015bm	0.40	2.7	3	3	7	3
$M_{SB_3\text{-}D_QSAR}$_76	1.00	0.38	3	N.4	qeq	2.00	3	8	15	26	4
$M_{SB_3\text{-}D_QSAR}$_77	0.96	0.47	2	O.3	eem2015ha	1.40	1.2	4	64	41	1
$M_{SB_3\text{-}D_QSAR}$_78	1.00	0.32	4	C.3	qeq	1.10	1.3	4	57	49	0
$M_{SB_3\text{-}D_QSAR}$_79	0.99	0.50	3	Br	eem	2.20	1.2	6	66	29	4
$M_{SB_3\text{-}D_QSAR}$_80	0.99	0.07	3	O.3	eem2015hn	2.00	1.6	6	28	2	3
$M_{SB_3\text{-}D_QSAR}$_81	1.00	0.45	4	I	eem2015ba	1.80	3.4	10	49	15	0
$M_{SB_3\text{-}D_QSAR}$_82	0.99	0.54	3	S.3	mmff94	0.10	1.1	6	10	22	3
$M_{SB_3\text{-}D_QSAR}$_83	0.99	0.58	3	C.3	gasteiger	3.00	2	7	75	15	3
$M_{SB_3\text{-}D_QSAR}$_84	0.99	0.58	3	C.3	eem2015hn	1.80	2.5	9	70	42	4
$M_{SB_3\text{-}D_QSAR}$_85	0.97	0.36	2	O.3	eem2015hm	0.20	3.5	3	9	6	2
$M_{SB_3\text{-}D_QSAR}$_86	0.95	0.38	2	S.o	eem2015bn	2.80	3.4	5	39	32	2
$M_{SB_3\text{-}D_QSAR}$_87	1.00	0.36	4	F	qeq	0.30	1	4	67	12	4
$M_{SB_3\text{-}D_QSAR}$_88	0.95	0.52	2	C.3	eem2015ba	2.40	2.1	7	32	26	0
$M_{SB_3\text{-}D_QSAR}$_89	0.98	0.56	2	C.3	eem2015hn	1.90	1.2	8	60	11	2
$M_{SB_3\text{-}D_QSAR}$_90	1.00	0.45	4	H	mmff94	1.30	1.2	4	79	43	4
$M_{SB_3\text{-}D_QSAR}$_91	0.96	0.57	2	O.3	mmff94	2.40	3.5	7	66	27	1
$M_{SB_3\text{-}D_QSAR}$_92	0.94	0.42	2	N.4	eem2015hm	0.20	3.5	7	36	33	3
$M_{SB_3\text{-}D_QSAR}$_93	1.00	0.54	6	C.3	eem	0.80	1.7	8	41	20	3
$M_{SB_3\text{-}D_QSAR}$_94	0.96	0.21	2	C.3.H2	eem2015ha	1.40	3.2	3	3	6	4
$M_{SB_3\text{-}D_QSAR}$_95	1.00	0.47	3	H.P	mmff94	1.10	1.8	4	10	48	2
$M_{SB_3\text{-}D_QSAR}$_96	0.99	0.49	3	Br	eem2015bm	1.40	3.1	3	53	38	2
$M_{SB_3\text{-}D_QSAR}$_97	0.99	0.56	3	F	eem	0.20	2.1	6	41	20	0
$M_{SB_3\text{-}D_QSAR}$_98	0.97	0.56	2	I	gasteiger	2.60	3	8	16	10	4
$M_{SB_3\text{-}D_QSAR}$_99	0.95	0.42	2	H	eem2015bm	1.00	3.3	7	9	3	2
$M_{SB_3\text{-}D_QSAR}$_100	1.00	0.54	3	S.o	eem2015bm	1.70	2.8	8	34	20	0
$M_{SB_3\text{-}D_QSAR}$_101	0.97	0.46	2	C.3.H1	eem2015ha	2.40	1.6	8	5	10	3
$M_{SB_3\text{-}D_QSAR}$_102	1.00	0.45	3	F	eem2015bn	1.40	2.8	8	51	45	2
$M_{SB_3\text{-}D_QSAR}$_103	1.00	0.57	4	C.3	openbabel	0.50	3.2	4	40	34	0
$M_{SB_3\text{-}D_QSAR}$_104	1.00	0.32	5	I	eem2015ha	0.60	3.1	6	45	33	0
$M_{SB_3\text{-}D_QSAR}$_105	1.00	0.48	3	S.o	qeq	1.70	2.2	3	37	45	1
$M_{SB_3\text{-}D_QSAR}$_106	1.00	0.45	4	H	eem2015hm	0.30	2.7	10	10	4	1
$M_{SB_3\text{-}D_QSAR}$_107	1.00	0.58	3	I	eem2015hm	2.10	1.5	9	38	17	3
$M_{SB_3\text{-}D_QSAR}$_108	0.96	0.38	2	C.3.H1	eem2015hm	0.60	3	6	34	23	0
$M_{SB_3\text{-}D_QSAR}$_109	0.99	0.45	3	C.3.H3	eem2015hm	1.70	1.3	7	57	31	4
$M_{SB_3\text{-}D_QSAR}$_110	0.99	0.52	3	OH2	mmff94	0.50	1.2	3	32	44	2
$M_{SB_3\text{-}D_QSAR}$_111	0.95	0.38	2	N.4	eem2015hn	1.20	3.4	10	4	25	0
$M_{SB_3\text{-}D_QSAR}$_112	1.00	0.47	5	OH2	eem2015ha	0.70	1.1	6	69	22	1
$M_{SB_3\text{-}D_QSAR}$_113	1.00	0.26	4	C.3.H1	qtpie	2.40	1.5	3	25	5	2
$M_{SB_3\text{-}D_QSAR}$_114	0.98	0.58	2	F	eem2015hn	2.80	2.6	8	23	19	1
$M_{SB_3\text{-}D_QSAR}$_115	1.00	0.55	3	Br	eem2015bm	0.60	2.7	3	53	39	4
$M_{SB_3\text{-}D_QSAR}$_116	0.99	0.48	3	S.o	eem2015bm	1.80	2	9	68	29	2
$M_{SB_3\text{-}D_QSAR}$_117	1.00	0.30	4	Br	qeq	1.50	3.1	4	42	36	4
$M_{SB_3\text{-}D_QSAR}$_118	0.99	0.53	3	O.3	eem2015ha	1.30	3.3	6	4	7	0

Table S29. SB 3-D QSAR models for the ligands extracted from the aligned complexes with MmpL3 protein.

Model ID	r^2	q^2cv	ONPC	Probe	Charge Model	Min Sigma	Grid Spacing	Grid Extension	Diel Const	CutOff	# Level
$M_{SB_3\text{-}D_QSAR_}$119	0.96	0.39	2	OH	eem2015hn	0.60	3.5	3	33	31	1
$M_{SB_3\text{-}D_QSAR_}$120	0.99	0.49	3	C.3.H2	openbabel	3.00	2.1	7	13	14	4
$M_{SB_3\text{-}D_QSAR_}$121	0.99	0.52	3	I	eem2015hn	0.20	1.3	9	18	16	1
$M_{SB_3\text{-}D_QSAR_}$122	0.99	0.50	3	O.3	eem2015ba	0.40	2.5	6	44	4	0
$M_{SB_3\text{-}D_QSAR_}$123	1.00	0.51	4	S.3	eem2015ha	3.00	2	10	77	30	4
$M_{SB_3\text{-}D_QSAR_}$124	1.00	0.45	2	S.o	qeq	1.90	1.1	6	71	24	1
$M_{SB_3\text{-}D_QSAR_}$125	0.94	0.49	2	OH2	eem2015ba	1.60	2.1	7	71	14	1
$M_{SB_3\text{-}D_QSAR_}$126	0.99	0.51	3	Br	gasteiger	2.00	1.3	5	10	21	0
$M_{SB_3\text{-}D_QSAR_}$127	0.97	0.59	2	F	qtpie	1.40	3.5	7	18	5	3
$M_{SB_3\text{-}D_QSAR_}$128	0.99	0.49	3	C.3.H2	eem2015bm	2.40	3	8	38	44	2
$M_{SB_3\text{-}D_QSAR_}$129	1.00	0.33	6	C.3.H1	qtpie	1.00	2.6	4	5	40	3
$M_{SB_3\text{-}D_QSAR_}$130	1.00	0.57	4	S.3	eem2015hn	1.90	3.5	4	72	46	2
$M_{SB_3\text{-}D_QSAR_}$131	0.99	0.40	3	I	eem2015ha	1.50	2.7	6	58	30	0
$M_{SB_3\text{-}D_QSAR_}$132	0.99	0.48	3	H.P	gasteiger	2.10	1.2	6	45	39	4
$M_{SB_3\text{-}D_QSAR_}$133	0.99	0.41	3	S.o	eem2015hm	2.20	1.3	4	6	48	0
$M_{SB_3\text{-}D_QSAR_}$134	0.99	0.42	3	C.3	eem2015bn	1.40	1.7	6	5	21	4
$M_{SB_3\text{-}D_QSAR_}$135	0.98	0.44	3	N.4	eem	0.20	2.9	5	60	17	3
$M_{SB_3\text{-}D_QSAR_}$136	0.99	0.39	3	Na	qeq	1.60	3.2	9	4	20	1
$M_{SB_3\text{-}D_QSAR_}$137	0.99	0.46	3	H.P	eem2015ha	0.60	1.6	9	9	23	4
$M_{SB_3\text{-}D_QSAR_}$138	1.00	0.47	7	C.3.H1	eem2015bm	2.70	2.2	3	35	18	2
$M_{SB_3\text{-}D_QSAR_}$139	1.00	0.47	4	N.4	qeq	1.60	2.4	9	18	16	2
$M_{SB_3\text{-}D_QSAR_}$140	1.00	0.55	4	H.P	eem2015ba	1.80	2.8	5	57	49	0
$M_{SB_3\text{-}D_QSAR_}$141	0.99	0.48	3	OH	eem2015ba	1.20	1.5	4	12	23	0
$M_{SB_3\text{-}D_QSAR_}$142	1.00	0.52	2	H.P	qtpie	2.10	1.4	9	20	13	3
$M_{SB_3\text{-}D_QSAR_}$143	0.99	0.45	3	N.4	eem2015bm	1.30	1	10	7	34	3
$M_{SB_3\text{-}D_QSAR_}$144	0.99	0.54	3	C.3.H2	eem	0.50	2	9	21	9	0
$M_{SB_3\text{-}D_QSAR_}$145	0.99	0.54	3	I	eem2015hn	1.80	2.6	4	80	41	3
$M_{SB_3\text{-}D_QSAR_}$146	0.99	0.48	3	O.3	eem2015ha	1.50	2	6	26	13	0
$M_{SB_3\text{-}D_QSAR_}$147	0.99	0.48	3	C.3.H3	mmff94	1.70	1.9	6	79	27	2
$M_{SB_3\text{-}D_QSAR_}$148	0.99	0.51	3	N.4	mmff94	2.80	2.4	8	35	48	0
$M_{SB_3\text{-}D_QSAR_}$149	0.99	0.50	3	Na	eem2015ba	2.10	1.6	9	30	30	1
$M_{SB_3\text{-}D_QSAR_}$150	0.98	0.46	3	Br	openbabel	3.00	2.3	7	13	44	2
$M_{SB_3\text{-}D_QSAR_}$151	1.00	0.56	4	N.4	openbabel	1.90	1.9	7	15	27	4
$M_{SB_3\text{-}D_QSAR_}$152	0.98	0.59	2	N.4	eem	2.50	1.5	10	16	9	3
$M_{SB_3\text{-}D_QSAR_}$153	1.00	0.47	3	OH	eem2015hm	3.00	2.3	7	26	21	3
$M_{SB_3\text{-}D_QSAR_}$154	0.99	0.43	3	H	eem2015bm	0.70	1.3	7	32	22	3
$M_{SB_3\text{-}D_QSAR_}$155	0.96	0.46	2	Cl	eem2015bn	0.80	1.6	5	9	29	1
$M_{SB_3\text{-}D_QSAR_}$156	0.99	0.62	2	O.3	eem2015ba	1.50	2	9	16	4	3
$M_{SB_3\text{-}D_QSAR_}$157	0.99	0.54	3	C.3.H3	eem	0.40	2.3	5	73	28	2
$M_{SB_3\text{-}D_QSAR_}$158	0.99	0.58	2	C.3	eem	2.50	1.8	4	25	9	4
$M_{SB_3\text{-}D_QSAR_}$159	0.95	0.47	2	S.3	eem2015hm	3.00	2.6	10	23	49	4
$M_{SB_3\text{-}D_QSAR_}$160	1.00	0.44	3	Na	eem2015hn	1.80	1.5	6	26	43	4
$M_{SB_3\text{-}D_QSAR_}$161	0.99	0.47	3	F	eem2015hm	2.10	1.8	9	51	48	2
$M_{SB_3\text{-}D_QSAR_}$162	0.99	0.50	3	Br	gasteiger	0.60	1.1	6	40	15	3
$M_{SB_3\text{-}D_QSAR_}$163	0.98	0.49	3	Br	eem	1.20	2.9	10	75	12	4
$M_{SB_3\text{-}D_QSAR_}$164	0.98	0.24	2	C.3.H3	qeq	2.50	2.3	10	73	35	3
$M_{SB_3\text{-}D_QSAR_}$165	0.99	0.43	3	C.3.H2	eem	0.50	2.7	5	40	30	3
$M_{SB_3\text{-}D_QSAR_}$166	0.99	0.43	3	Cl	qeq	1.50	1.2	6	2	32	0
$M_{SB_3\text{-}D_QSAR_}$167	0.99	0.44	3	S.o	eem2015bn	1.40	1.1	5	15	25	3
$M_{SB_3\text{-}D_QSAR_}$168	1.00	0.44	5	H.P	gasteiger	2.00	3	7	18	48	3
$M_{SB_3\text{-}D_QSAR_}$169	0.00	-0.36	1	O.3	eem2015ba	2.90	2.6	8	66	2	1
$M_{SB_3\text{-}D_QSAR_}$170	1.00	0.60	5	F	qeq	1.80	3.4	8	18	9	4
$M_{SB_3\text{-}D_QSAR_}$171	0.99	0.50	3	C.3.H1	eem2015bn	2.30	1.4	6	76	17	4
$M_{SB_3\text{-}D_QSAR_}$172	1.00	0.47	4	Cl	mmff94	2.20	2.7	3	50	32	0
$M_{SB_3\text{-}D_QSAR_}$173	0.99	0.47	3	N.4	eem2015bm	1.70	1.4	4	30	32	4
$M_{SB_3\text{-}D_QSAR_}$174	0.99	0.51	3	H	eem2015bn	0.10	2.1	8	62	36	2
$M_{SB_3\text{-}D_QSAR_}$175	1.00	0.65	3	Na	eem2015hn	2.20	2.5	9	11	6	4
$M_{SB_3\text{-}D_QSAR_}$176	0.99	0.51	3	I	eem	0.20	2.6	10	77	31	2
$M_{SB_3\text{-}D_QSAR_}$177	0.94	0.46	2	OH	eem2015hn	2.50	3.3	4	49	39	3

Table S29. SB 3-D QSAR models for the ligands extracted from the aligned complexes with MmpL3 protein.

Model ID	r^2	q^2cv	ONPC	Probe	Charge Model	Min Sigma	Grid Spacing	Grid Extension	Diel Const	CutOff	# Level
$M_{SB\_3-D\_QSAR\_}178$	1.00	0.57	3	Br	eem2015hm	2.40	2.4	7	52	21	1
$M_{SB\_3-D\_QSAR\_}179$	0.99	0.38	3	OH2	mmff94	1.10	3.5	8	64	50	2
$M_{SB\_3-D\_QSAR\_}180$	0.99	0.52	3	C.3.H1	mmff94	0.60	1.9	4	32	49	1
$M_{SB\_3-D\_QSAR\_}181$	0.99	0.44	3	O.3	qeq	0.20	3.5	7	69	11	2
$M_{SB\_3-D\_QSAR\_}182$	0.99	0.58	3	S.3	eem2015bm	0.30	2.9	10	7	43	1
$M_{SB\_3-D\_QSAR\_}183$	0.96	0.47	2	Br	qtpie	1.00	3.5	9	28	13	3
$M_{SB\_3-D\_QSAR\_}184$	0.94	0.46	2	OH2	openbabel	1.70	2.5	7	45	40	2
$M_{SB\_3-D\_QSAR\_}185$	0.99	0.49	3	H.P	gasteiger	1.50	1.6	8	53	49	3
$M_{SB\_3-D\_QSAR\_}186$	0.96	0.47	2	Cl	eem2015hn	2.20	2.6	7	33	33	2
$M_{SB\_3-D\_QSAR\_}187$	0.98	0.39	2	Na	eem2015hm	1.80	3.3	10	27	47	2
$M_{SB\_3-D\_QSAR\_}188$	0.99	0.51	3	OH2	openbabel	0.40	1.1	10	32	8	2
$M_{SB\_3-D\_QSAR\_}189$	1.00	0.44	6	OH	qeq	0.70	1.7	9	74	18	2
$M_{SB\_3-D\_QSAR\_}190$	0.99	0.64	3	Cl	qtpie	1.90	3.3	10	3	1	4
$M_{SB\_3-D\_QSAR\_}191$	1.00	0.46	7	Cl	qeq	1.20	1.8	10	22	37	1
$M_{SB\_3-D\_QSAR\_}192$	1.00	0.55	4	OH2	eem2015bm	2.10	2.4	9	52	45	0
$M_{SB\_3-D\_QSAR\_}193$	0.99	0.45	3	C.3.H1	eem2015bm	1.20	1.6	10	61	31	2
$M_{SB\_3-D\_QSAR\_}194$	0.97	0.41	2	H	eem2015hn	1.50	3.4	5	9	4	2
$M_{SB\_3-D\_QSAR\_}195$	0.98	0.56	2	S.o	eem2015ha	2.00	1.9	3	57	9	4
$M_{SB\_3-D\_QSAR\_}196$	0.98	0.52	3	F	eem2015bm	0.40	3.5	4	62	28	4
$M_{SB\_3-D\_QSAR\_}197$	1.00	0.40	5	S.o	qtpie	2.70	1.4	7	19	21	1
$M_{SB\_3-D\_QSAR\_}198$	1.00	0.42	4	F	qtpie	2.60	3.4	8	74	36	1
$M_{SB\_3-D\_QSAR\_}199$	1.00	0.48	6	OH	openbabel	1.20	2.6	4	41	33	4
$M_{SB\_3-D\_QSAR\_}200$	0.99	0.50	3	OH2	eem2015hn	1.90	2	9	22	27	4
$M_{SB\_3-D\_QSAR\_}201$	0.99	0.42	3	OH	eem2015hn	0.80	2.2	6	46	15	3
$M_{SB\_3-D\_QSAR\_}202$	1.00	0.50	3	Cl	eem2015hm	0.90	1.2	10	50	8	2
$M_{SB\_3-D\_QSAR\_}203$	0.97	0.39	2	C.3.H2	eem2015ba	0.10	2.2	10	58	1	2
$M_{SB\_3-D\_QSAR\_}204$	1.00	0.46	3	Cl	qtpie	1.00	2.1	10	33	26	3
$M_{SB\_3-D\_QSAR\_}205$	0.99	0.45	3	Na	openbabel	1.40	2	3	46	30	4

Table S30. Details for the $M_{SB_3\text{-}D_QSAR}$_1 model

PC	r^2	SDEC	q^2_{cv}	SDEP $_{cv}$
1	0.47	0.71	-0.15	1.05
2	0.96	0.20	0.48	0.71
3*	0.99	0.10	0.52	0.68

The star indicate the optimal number of PCs selected by a the max q^2_{cv} value.

Table S31. Details for the $M_{SB_3\text{-}D_QSAR}$_59 model

PC	r^2	SDEC	q^2_{cv}	SDEP $_{cv}$
1	0.88	0.34	0.64	0.59
2	0.99	0.10	0.73	0.51
3*	1.00	0.02	0.72	0.52

The star indicate the optimal number of PCs selected by a the max q^2_{cv} value.

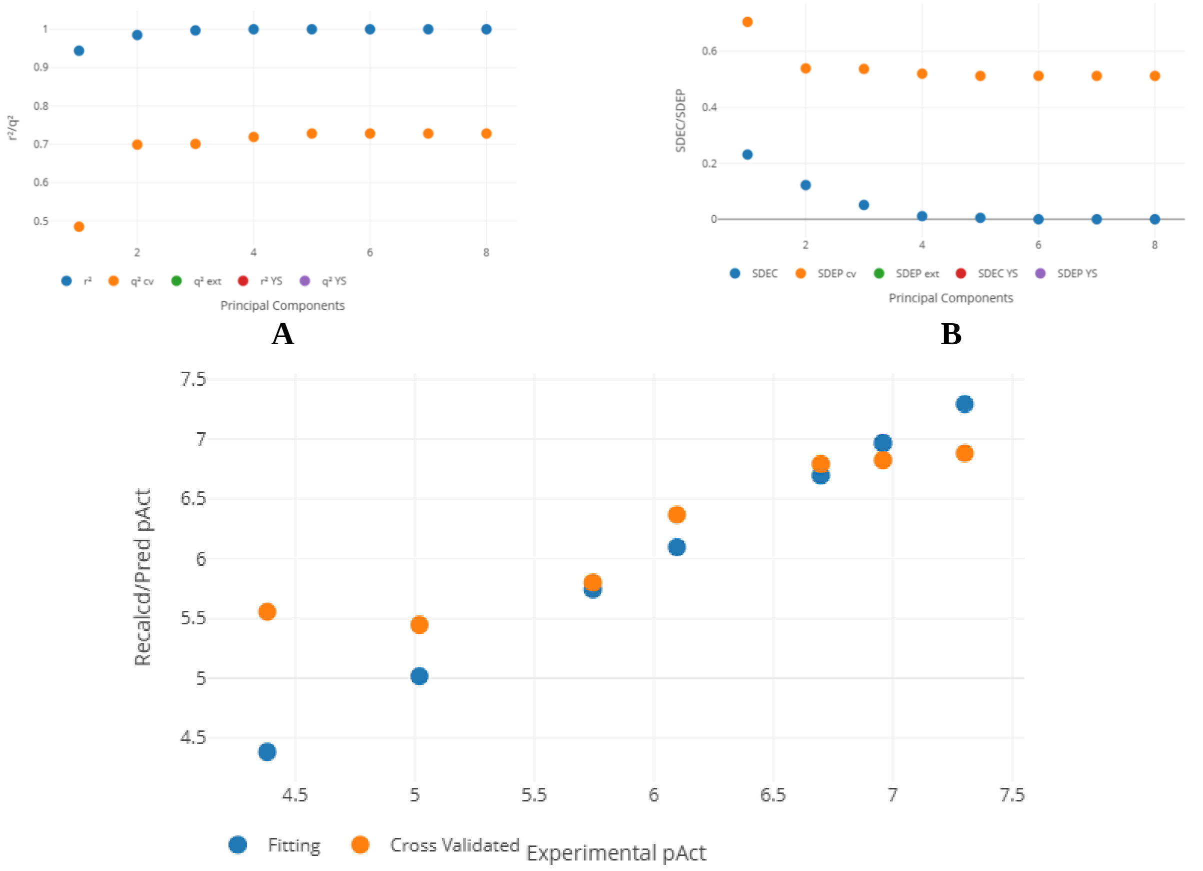

C

Figure S11. Model $M_{SB_3\text{-}D_QSAR}$_59: (A) r^2 and q^2_{cv} values in relation to number of PCs. (B) SDEC and $SDEP_{cv}$ in relation with the number of PC and the (C) experimental versus the recalculated/CV-predicted pMIC (pAct) values at 2 PCs. In blue, the r^2, SDEC, and experimental fitted values. In orange, the q^2_{cv}, $SDEP_{cv}$, and crossvalidated values.

Table S32. Statistical coefficients for the COMBINE models $M_{COMBINE\_1}$- $M_{COMBINE\_7}$

#	PDB Code	ONPC	r^2	SDEC	q^2_{cv}	SDEP
$M_{COMBINE\_1}$	6AJG	1	0.62	0.61	0.11	0.93
$M_{COMBINE\_2}$	6AJH	2	0.97	0.16	0.50	0.70
$M_{COMBINE\_3}$	6AJI	1	0.55	0.66	0.11	0.93
$M_{COMBINE\_4}$	6AJJ	1	0.91	0.29	0.62	0.61
$M_{COMBINE\_5}$	7C2M	1	0.75	0.49	0.34	0.80
$M_{COMBINE\_6}$	7C2N	2	0.99	0.09	0.13	0.91
$M_{COMBINE\_7}$	7WNX	1	0.77	0.47	0.44	0.73
$M_{COMBINE\_4SA}$	6AJJ	2	0.97	0.16	0.91	0.30

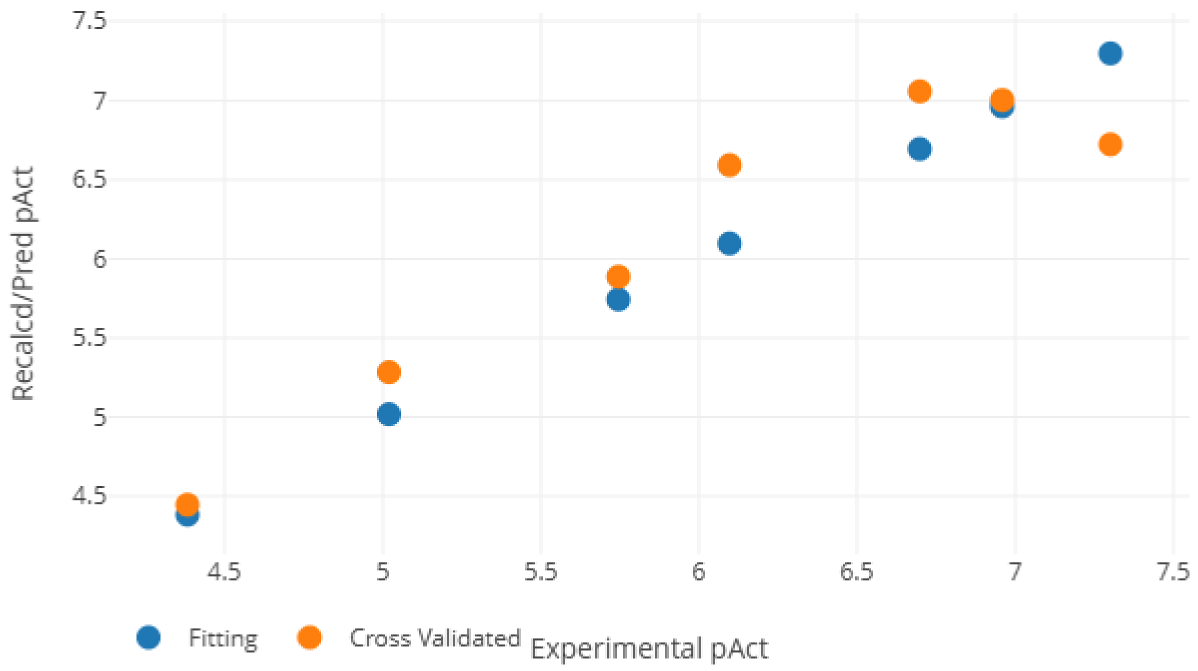

Figure S12. Model $M_{COMBINE\_4SA}$: experimental versus the recalculated/CV-predicted pMIC (pAct) values at 2 PCs. In blue, the experimental vs fitted values. In orange, the experimental vs crossvalidated values.

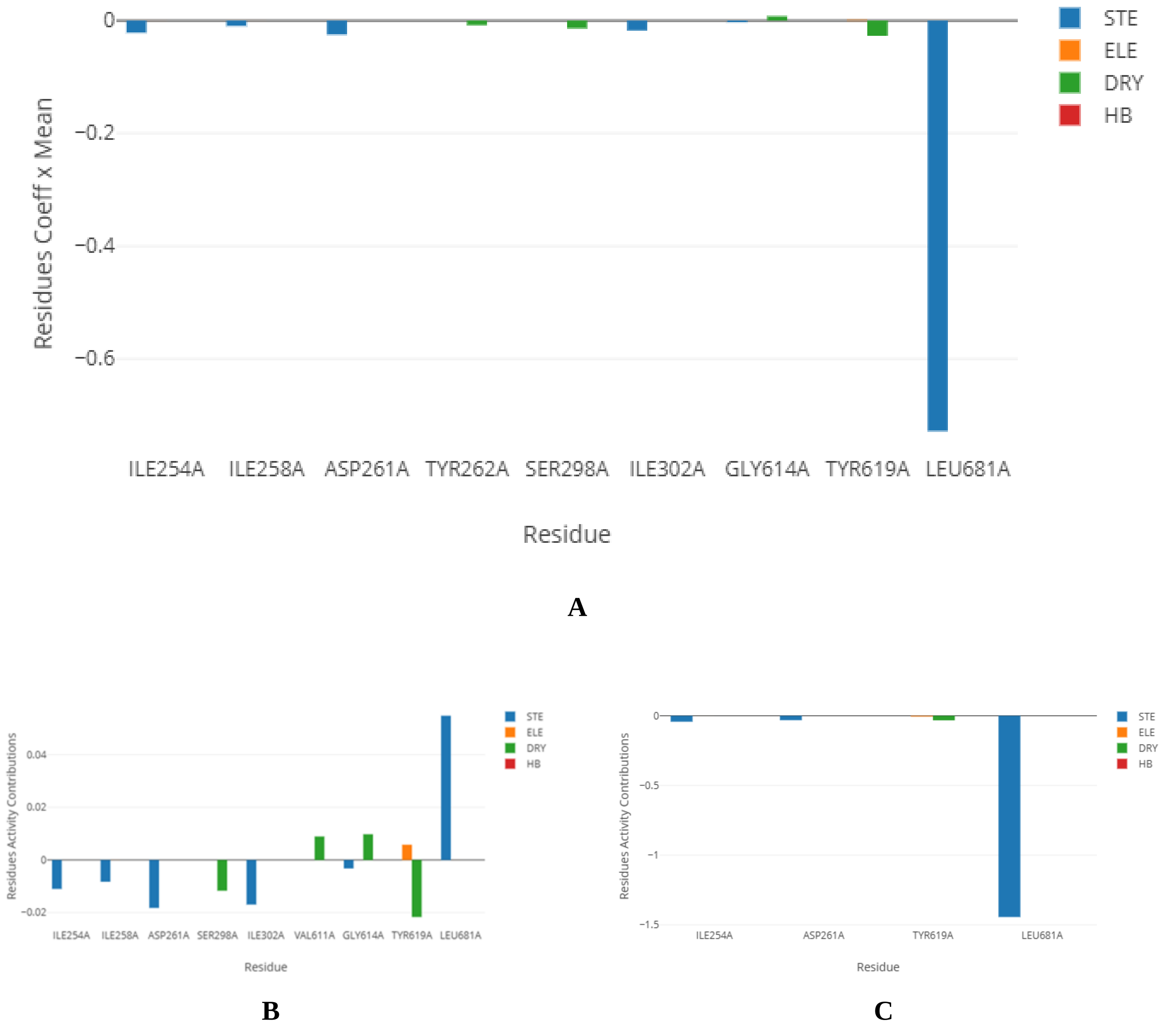

Figure S13. Model M$_{COMBINE}$_4$_{SA}$: (A) AAC plot; (B) AC plot for the 7CM2 compound, AC plot for the 7WNX compounds.

Table S33. SB 3-D QSAR models from ligands minimized into the Mmpl3 protein obtained from the 6AJJ pdb code.

Model ID	r^2	q^2cv	ONPC	Probe	Charge Model	Min Sigma	Grid Spacing	Grid Extension	Diel Const	CutOff	# Level
$M_{SB\_3-D\_QSAR\_}1_{MIN}$	0.90	0.36	2	C.3	gasteiger	2.0	2.0	5	8	30	0
$M_{SB\_3-D\_QSAR\_}2_{MIN}$	0.92	0.45	2	I	qeq	1.9	3.3	10	47	27	3
$M_{SB\_3-D\_QSAR\_}3_{MIN}$	0.90	0.41	2	Cl	gasteiger	2.0	3.2	3	45	45	0
$M_{SB\_3-D\_QSAR\_}4_{MIN}$	0.98	0.37	2	Cl	qeq	2.3	1.9	4	48	13	2
$M_{SB\_3-D\_QSAR\_}5_{MIN}$	0.93	0.49	2	N.4	eem2015bm	2.9	3.0	7	7	8	1
$M_{SB\_3-D\_QSAR\_}6_{MIN}$	0.89	0.39	2	C.3.H1	eem	0.5	3.3	7	69	24	0
$M_{SB\_3-D\_QSAR\_}7_{MIN}$	0.89	0.40	2	S.o	eem2015ha	1.0	3.4	7	18	37	2
$M_{SB\_3-D\_QSAR\_}8_{MIN}$	0.93	0.42	2	F	eem2015ha	1.5	1.2	8	36	47	0
$M_{SB\_3-D\_QSAR\_}9_{MIN}$	0.93	0.35	2	C.3.H1	eem2015bn	1.6	1.8	10	2	36	2
$M_{SB\_3-D\_QSAR\_}10_{MIN}$	1.00	0.06	3	H	eem2015hm	0.1	3.5	3	24	20	2
$M_{SB\_3-D\_QSAR\_}11_{MIN}$	0.85	0.16	2	O.3	openbabel	1.6	2.7	7	45	45	3
$M_{SB\_3-D\_QSAR\_}12_{MIN}$	0.92	0.40	2	Br	eem2015ha	2.0	1.5	5	43	46	1
$M_{SB\_3-D\_QSAR\_}13_{MIN}$	1.00	0.54	3	S.3	qtpie	0.7	1.5	10	45	3	1
$M_{SB\_3-D\_QSAR\_}14_{MIN}$	0.92	0.31	2	C.3.H3	qeq	0.2	2.3	8	25	30	4
$M_{SB\_3-D\_QSAR\_}15_{MIN}$	1.00	0.61	3	H	eem2015bn	1.3	2.7	9	64	7	0
$M_{SB\_3-D\_QSAR\_}16_{MIN}$	0.99	0.39	3	C.3.H3	eem	1.4	1.5	8	76	44	0
$M_{SB\_3-D\_QSAR\_}17_{MIN}$	0.99	0.63	2	O.3	eem2015ha	1.8	2.3	9	39	6	2
$M_{SB\_3-D\_QSAR\_}18_{MIN}$	0.94	0.47	2	Cl	eem	1.1	2.6	9	70	7	4
$M_{SB\_3-D\_QSAR\_}19_{MIN}$	0.91	0.35	2	N.4	openbabel	2.3	1.5	10	43	40	2
$M_{SB\_3-D\_QSAR\_}20_{MIN}$	0.98	0.17	3	O.3	openbabel	2.5	1.1	3	51	6	0
$M_{SB\_3-D\_QSAR\_}21_{MIN}$	0.89	0.29	2	S.o	gasteiger	2.5	2.9	6	61	47	0
$M_{SB\_3-D\_QSAR\_}22_{MIN}$	0.93	0.47	2	C.3.H1	eem	0.6	2.1	7	70	9	0
$M_{SB\_3-D\_QSAR\_}23_{MIN}$	0.93	0.42	2	OH2	eem2015bm	0.1	1.6	5	30	8	1
$M_{SB\_3-D\_QSAR\_}24_{MIN}$	1.00	0.36	3	F	qtpie	0.3	1.6	7	27	40	4
$M_{SB\_3-D\_QSAR\_}25_{MIN}$	1.00	0.75	3	F	qtpie	1.4	2.5	6	69	27	0
$M_{SB\_3-D\_QSAR\_}26_{MIN}$	0.93	0.38	2	H.P	eem2015bn	0.6	2.1	9	6	11	0
$M_{SB\_3-D\_QSAR\_}27_{MIN}$	0.94	0.46	2	N.4	gasteiger	0.1	2.8	3	32	37	1
$M_{SB\_3-D\_QSAR\_}28_{MIN}$	0.90	0.19	2	F	qeq	0.6	2.4	10	29	26	2
$M_{SB\_3-D\_QSAR\_}29_{MIN}$	0.89	0.41	2	H.P	eem2015ha	0.5	2.0	6	37	15	4
$M_{SB\_3-D\_QSAR\_}30_{MIN}$	0.92	0.38	2	C.3.H1	mmff94	0.3	2.9	3	18	34	3
$M_{SB\_3-D\_QSAR\_}31_{MIN}$	0.99	0.65	3	OH	openbabel	1.6	3.0	5	30	5	2
$M_{SB\_3-D\_QSAR\_}32_{MIN}$	0.92	0.41	2	F	mmff94	2.1	1.4	7	60	25	4
$M_{SB\_3-D\_QSAR\_}33_{MIN}$	0.99	0.38	3	Cl	qeq	0.5	2.0	4	29	12	1
$M_{SB\_3-D\_QSAR\_}34_{MIN}$	0.95	0.49	2	C.3.H3	qtpie	1.3	1.4	9	30	9	4
$M_{SB\_3-D\_QSAR\_}35_{MIN}$	0.92	0.45	2	H.P	openbabel	2.5	3.0	5	44	8	0
$M_{SB\_3-D\_QSAR\_}36_{MIN}$	0.99	0.51	3	OH2	eem	2.2	1.9	7	75	23	2
$M_{SB\_3-D\_QSAR\_}37_{MIN}$	0.94	0.34	2	H	eem2015bm	3.0	2.4	8	56	25	3
$M_{SB\_3-D\_QSAR\_}38_{MIN}$	0.99	0.42	3	C.3.H2	eem	0.5	1.2	7	63	34	1
$M_{SB\_3-D\_QSAR\_}39_{MIN}$	0.91	0.39	2	C.3.H2	qeq	0.2	2.4	8	3	17	3
$M_{SB\_3-D\_QSAR\_}40_{MIN}$	0.99	0.48	3	S.o	eem2015hm	1.8	3.1	4	18	17	1
$M_{SB\_3-D\_QSAR\_}41_{MIN}$	0.91	0.42	2	OH2	mmff94	2.6	2.7	9	17	20	4
$M_{SB\_3-D\_QSAR\_}42_{MIN}$	0.91	0.39	2	H.P	mmff94	0.5	1.6	8	59	41	2
$M_{SB\_3-D\_QSAR\_}43_{MIN}$	0.92	0.41	2	C.3.H2	mmff94	0.3	2.6	4	44	40	2
$M_{SB\_3-D\_QSAR\_}44_{MIN}$	0.90	0.29	2	OH	eem2015ba	1.0	3.1	9	48	31	2
$M_{SB\_3-D\_QSAR\_}45_{MIN}$	0.95	0.49	2	H	qtpie	2.4	1.8	8	9	20	1
$M_{SB\_3-D\_QSAR\_}46_{MIN}$	0.86	0.20	2	C.3.H1	eem2015ha	1.9	2.6	5	43	41	2
$M_{SB\_3-D\_QSAR\_}47_{MIN}$	0.89	0.33	2	Na	eem2015ha	2.0	2.6	5	57	28	2
$M_{SB\_3-D\_QSAR\_}48_{MIN}$	0.87	0.36	2	C.3.H3	gasteiger	0.7	3.3	5	42	11	0
$M_{SB\_3-D\_QSAR\_}49_{MIN}$	0.93	0.36	2	C.3.H3	eem2015bn	0.8	1.8	6	51	12	1
$M_{SB\_3-D\_QSAR\_}50_{MIN}$	0.91	0.34	2	C.3.H2	eem2015bn	1.4	2.0	10	78	21	1
$M_{SB\_3-D\_QSAR\_}51_{MIN}$	0.92	0.35	2	Na	eem2015ba	2.7	2.5	9	2	31	3
$M_{SB\_3-D\_QSAR\_}52_{MIN}$	1.00	0.43	3	S.3	eem2015bm	2.8	2.3	6	33	43	4
$M_{SB\_3-D\_QSAR\_}53_{MIN}$	0.91	0.30	2	C.3	openbabel	1.8	2.7	4	23	43	0
$M_{SB\_3-D\_QSAR\_}54_{MIN}$	0.87	0.24	2	C.3	gasteiger	2.7	2.7	7	78	47	1
$M_{SB\_3-D\_QSAR\_}55_{MIN}$	1.00	0.53	3	I	eem2015hn	3.0	1.5	4	29	14	2
$M_{SB\_3-D\_QSAR\_}56_{MIN}$	0.91	0.38	2	C.3	mmff94	0.8	1.9	5	2	49	3
$M_{SB\_3-D\_QSAR\_}57_{MIN}$	0.93	0.39	2	H.P	qtpie	1.7	3.0	5	76	45	2
$M_{SB\_3-D\_QSAR\_}58_{MIN}$	0.99	0.40	3	Na	eem	0.1	2.3	3	13	49	1

Table S33. SB 3-D QSAR models from ligands minimized into the Mmpl3 protein obtained from the 6AJJ pdb code.

$M_{SB\_3-D\_QSAR}\_59_{MIN}$	0.94	0.36	2	S.3	eem2015hm	0.2	1.5	9	40	34	1
$M_{SB\_3-D\_QSAR}\_60_{MIN}$	1.00	0.43	3	H.P	eem2015bn	1.1	3.5	4	25	38	0
$M_{SB\_3-D\_QSAR}\_61_{MIN}$	0.90	0.40	2	I	eem2015hm	2.3	2.0	7	30	25	3
$M_{SB\_3-D\_QSAR}\_62_{MIN}$	0.88	0.33	2	N.4	eem2015bn	1.6	2.5	8	68	27	0
$M_{SB\_3-D\_QSAR}\_63_{MIN}$	0.91	0.38	2	OH2	openbabel	0.5	1.4	10	46	41	3
$M_{SB\_3-D\_QSAR}\_64_{MIN}$	0.99	0.60	3	H.P	mmff94	0.2	3.2	4	44	34	2
$M_{SB\_3-D\_QSAR}\_65_{MIN}$	0.99	0.37	3	C.3.H3	qtpie	1.1	2.8	8	6	12	3
$M_{SB\_3-D\_QSAR}\_66_{MIN}$	0.92	0.41	2	C.3.H1	eem2015ha	1.2	2.8	8	54	47	3
$M_{SB\_3-D\_QSAR}\_67_{MIN}$	0.92	0.33	2	H	openbabel	2.2	1.5	4	40	35	1
$M_{SB\_3-D\_QSAR}\_68_{MIN}$	0.94	0.38	2	Na	eem2015bn	0.7	1.3	4	26	9	4
$M_{SB\_3-D\_QSAR}\_69_{MIN}$	0.92	0.37	2	H.P	openbabel	2.1	1.3	7	27	28	3
$M_{SB\_3-D\_QSAR}\_70_{MIN}$	0.99	0.42	3	N.4	eem2015bn	1.9	1.3	4	47	47	0
$M_{SB\_3-D\_QSAR}\_71_{MIN}$	0.92	0.33	2	Cl	eem2015bm	0.4	1.8	9	69	38	1
$M_{SB\_3-D\_QSAR}\_72_{MIN}$	0.91	0.38	2	C.3	openbabel	1.2	2.6	8	71	47	0
$M_{SB\_3-D\_QSAR}\_73_{MIN}$	0.92	0.41	2	O.3	qtpie	1.4	2.3	8	22	34	4
$M_{SB\_3-D\_QSAR}\_74_{MIN}$	0.90	0.28	2	OH2	eem2015hm	2.0	2.2	9	6	45	4
$M_{SB\_3-D\_QSAR}\_75_{MIN}$	0.99	0.62	3	H.P	eem2015ha	3.0	3.0	9	71	40	4
$M_{SB\_3-D\_QSAR}\_76_{MIN}$	0.91	0.33	2	Cl	eem2015bn	1.9	3.2	4	47	41	1
$M_{SB\_3-D\_QSAR}\_77_{MIN}$	0.92	0.32	2	I	eem2015bm	0.4	2.3	7	74	31	0
$M_{SB\_3-D\_QSAR}\_78_{MIN}$	1.00	0.57	3	I	qeq	0.9	3.0	8	34	11	3
$M_{SB\_3-D\_QSAR}\_79_{MIN}$	1.00	0.73	3	H	eem2015hn	1.3	3.2	5	42	3	3
$M_{SB\_3-D\_QSAR}\_80_{MIN}$	0.98	0.51	3	I	eem2015hn	2.0	3.3	7	35	45	1
$M_{SB\_3-D\_QSAR}\_81_{MIN}$	0.93	0.36	2	Br	eem2015bn	0.4	1.4	10	19	31	4
$M_{SB\_3-D\_QSAR}\_82_{MIN}$	0.92	0.38	2	OH	qtpie	0.5	2.9	5	35	39	2
$M_{SB\_3-D\_QSAR}\_83_{MIN}$	0.90	0.67	1	H.P	qeq	1.2	1.5	7	35	1	3
$M_{SB\_3-D\_QSAR}\_84_{MIN}$	1.00	0.48	3	H.P	qeq	1.7	3.2	6	26	45	4
$M_{SB\_3-D\_QSAR}\_85_{MIN}$	0.92	0.43	2	Cl	gasteiger	2.6	1.2	6	74	10	0
$M_{SB\_3-D\_QSAR}\_86_{MIN}$	0.91	0.36	2	H.P	gasteiger	2.6	1.5	3	75	43	2
$M_{SB\_3-D\_QSAR}\_87_{MIN}$	0.93	0.43	2	C.3	eem2015ha	1.3	2.1	10	80	14	3
$M_{SB\_3-D\_QSAR}\_88_{MIN}$	0.99	0.63	2	Na	eem2015hm	2.2	1.2	5	66	8	1
$M_{SB\_3-D\_QSAR}\_89_{MIN}$	0.92	0.39	2	I	eem2015ha	1.6	1.4	4	4	27	0
$M_{SB\_3-D\_QSAR}\_90_{MIN}$	1.00	0.59	3	OH2	eem2015bm	1.9	1.3	6	27	5	3
$M_{SB\_3-D\_QSAR}\_91_{MIN}$	0.94	0.64	1	Br	gasteiger	0.6	2.1	5	38	1	3
$M_{SB\_3-D\_QSAR}\_92_{MIN}$	1.00	0.41	3	I	qeq	1.9	1.5	5	51	35	1
$M_{SB\_3-D\_QSAR}\_93_{MIN}$	0.90	0.41	2	N.4	eem2015ba	0.8	2.0	6	46	41	3
$M_{SB\_3-D\_QSAR}\_94_{MIN}$	0.93	0.35	2	I	eem2015ba	0.3	1.7	9	32	37	0
$M_{SB\_3-D\_QSAR}\_95_{MIN}$	0.92	0.43	2	Na	eem2015hm	0.6	2.3	9	74	31	4
$M_{SB\_3-D\_QSAR}\_96_{MIN}$	0.92	0.38	2	S.o	eem2015ha	1.5	2.8	8	45	29	0
$M_{SB\_3-D\_QSAR}\_97_{MIN}$	1.00	0.75	2	C.3.H2	eem2015hm	1.3	2.1	9	51	1	4
$M_{SB\_3-D\_QSAR}\_98_{MIN}$	0.94	0.39	2	N.4	eem2015bn	0.9	1.0	8	11	34	4
$M_{SB\_3-D\_QSAR}\_99_{MIN}$	0.84	0.11	2	H.P	gasteiger	1.0	3.5	10	23	40	1
$M_{SB\_3-D\_QSAR}\_100_{MIN}$	0.92	0.36	2	Na	eem2015ha	2.4	3.3	4	68	48	1
$M_{SB\_3-D\_QSAR}\_101_{MIN}$	1.00	0.56	3	C.3	eem2015hn	2.9	2.5	6	42	12	3
$M_{SB\_3-D\_QSAR}\_102_{MIN}$	0.91	0.28	2	C.3.H1	eem2015ba	1.1	2.2	6	79	16	2
$M_{SB\_3-D\_QSAR}\_103_{MIN}$	0.96	0.49	2	O.3	eem2015hn	2.3	1.9	10	69	24	2
$M_{SB\_3-D\_QSAR}\_104_{MIN}$	0.99	0.20	3	N.4	eem2015bn	1.3	3.5	10	38	24	0
$M_{SB\_3-D\_QSAR}\_105_{MIN}$	0.90	0.49	1	H	gasteiger	2.1	1.7	6	20	5	0
$M_{SB\_3-D\_QSAR}\_106_{MIN}$	0.99	0.37	3	S.o	eem	0.9	1.2	7	62	26	2
$M_{SB\_3-D\_QSAR}\_107_{MIN}$	0.89	0.38	2	Cl	mmff94	0.3	3.2	4	49	8	0
$M_{SB\_3-D\_QSAR}\_108_{MIN}$	0.91	0.32	2	I	mmff94	0.1	2.2	5	31	33	3
$M_{SB\_3-D\_QSAR}\_109_{MIN}$	0.99	0.56	3	I	openbabel	2.9	3.3	3	55	14	1
$M_{SB\_3-D\_QSAR}\_110_{MIN}$	0.99	0.55	3	C.3.H3	qeq	0.8	3.3	4	14	29	0
$M_{SB\_3-D\_QSAR}\_111_{MIN}$	0.99	0.52	3	Br	eem	2.2	2.2	8	78	15	4
$M_{SB\_3-D\_QSAR}\_112_{MIN}$	0.72	0.36	1	I	eem2015ha	1.8	3.1	5	62	2	4
$M_{SB\_3-D\_QSAR}\_113_{MIN}$	0.90	0.28	2	S.3	eem2015ba	0.8	3.0	9	3	46	3
$M_{SB\_3-D\_QSAR}\_114_{MIN}$	0.94	0.28	2	C.3	qeq	0.2	1.3	6	50	41	1
$M_{SB\_3-D\_QSAR}\_115_{MIN}$	0.82	0.36	1	H	qtpie	2.0	2.2	3	57	21	0
$M_{SB\_3-D\_QSAR}\_116_{MIN}$	0.85	0.22	2	OH	openbabel	2.2	3.1	6	53	50	2
$M_{SB\_3-D\_QSAR}\_117_{MIN}$	0.92	0.34	2	OH	qeq	0.2	3.5	7	12	10	1

Table S33. SB 3-D QSAR models from ligands minimized into the Mmpl3 protein obtained from the 6AJJ pdb code.

$M_{SB\_3\text{-}D\_QSAR\_}118_{MIN}$	1.00	0.68	3	O.3	eem2015hn	1.0	3.5	4	60	4	2
$M_{SB\_3\text{-}D\_QSAR\_}119_{MIN}$	0.96	0.51	2	S.o	eem2015hm	3.0	2.2	4	42	13	3
$M_{SB\_3\text{-}D\_QSAR\_}120_{MIN}$	0.99	0.34	3	C.3.H3	eem	1.5	1.2	6	45	36	3
$M_{SB\_3\text{-}D\_QSAR\_}121_{MIN}$	0.00	-0.36	1	OH	eem2015hm	2.5	1.2	4	29	1	3
$M_{SB\_3\text{-}D\_QSAR\_}122_{MIN}$	0.90	0.35	2	C.3.H3	openbabel	2.5	1.4	4	45	36	3
$M_{SB\_3\text{-}D\_QSAR\_}123_{MIN}$	0.94	0.37	2	C.3.H2	eem2015ha	2.7	1.0	5	2	24	4
$M_{SB\_3\text{-}D\_QSAR\_}124_{MIN}$	1.00	0.55	3	Br	eem2015hn	2.8	2.4	5	56	20	4
$M_{SB\_3\text{-}D\_QSAR\_}125_{MIN}$	0.93	0.41	2	C.3.H3	qtpie	0.7	1.8	9	77	36	0
$M_{SB\_3\text{-}D\_QSAR\_}126_{MIN}$	1.00	0.55	3	I	eem2015hn	1.5	2.9	6	26	10	0
$M_{SB\_3\text{-}D\_QSAR\_}127_{MIN}$	0.94	0.43	2	F	eem2015bm	1.4	1.7	10	64	49	3
$M_{SB\_3\text{-}D\_QSAR\_}128_{MIN}$	0.96	0.58	2	N.4	eem	1.9	2.9	8	25	18	4
$M_{SB\_3\text{-}D\_QSAR\_}129_{MIN}$	0.90	0.17	2	C.3.H2	openbabel	0.3	2.9	7	10	30	2
$M_{SB\_3\text{-}D\_QSAR\_}130_{MIN}$	0.91	0.33	2	S.o	gasteiger	0.6	2.6	3	53	22	0
$M_{SB\_3\text{-}D\_QSAR\_}131_{MIN}$	0.94	0.42	2	N.4	eem2015bn	2.1	2.5	5	5	10	4
$M_{SB\_3\text{-}D\_QSAR\_}132_{MIN}$	0.94	0.28	2	C.3.H3	qtpie	2.3	1.9	8	41	32	0
$M_{SB\_3\text{-}D\_QSAR\_}133_{MIN}$	0.95	0.31	2	C.3	qeq	2.0	2.5	8	35	31	0
$M_{SB\_3\text{-}D\_QSAR\_}134_{MIN}$	0.00	-0.36	1	Cl	eem	2.2	3.2	5	78	2	1
$M_{SB\_3\text{-}D\_QSAR\_}135_{MIN}$	0.93	0.32	2	N.4	eem2015bm	0.3	2.2	6	25	14	0
$M_{SB\_3\text{-}D\_QSAR\_}136_{MIN}$	0.93	0.38	2	H	eem2015ha	1.2	1.7	9	2	36	2
$M_{SB\_3\text{-}D\_QSAR\_}137_{MIN}$	0.95	0.44	2	I	qeq	1.7	1.2	3	40	28	2
$M_{SB\_3\text{-}D\_QSAR\_}138_{MIN}$	0.87	0.13	2	Cl	mmff94	0.2	3.5	3	52	40	0
$M_{SB\_3\text{-}D\_QSAR\_}139_{MIN}$	1.00	0.46	3	OH	gasteiger	1.3	3.5	10	39	8	3
$M_{SB\_3\text{-}D\_QSAR\_}140_{MIN}$	1.00	0.48	3	O.3	eem2015hm	1.2	2.8	6	72	43	2
$M_{SB\_3\text{-}D\_QSAR\_}141_{MIN}$	0.91	0.36	2	F	mmff94	1.3	2.2	6	30	29	1
$M_{SB\_3\text{-}D\_QSAR\_}142_{MIN}$	0.92	0.41	2	C.3.H3	mmff94	1.5	1.3	3	25	33	1
$M_{SB\_3\text{-}D\_QSAR\_}143_{MIN}$	0.91	0.39	2	N.4	mmff94	1.5	2.8	7	28	50	1
$M_{SB\_3\text{-}D\_QSAR\_}144_{MIN}$	0.93	0.39	2	C.3	eem2015ha	1.0	1.7	9	53	44	4
$M_{SB\_3\text{-}D\_QSAR\_}145_{MIN}$	0.00	-0.36	1	S.o	eem2015hm	2.4	3.4	5	70	2	3
$M_{SB\_3\text{-}D\_QSAR\_}146_{MIN}$	0.92	0.34	2	OH2	eem2015bn	2.4	1.0	10	51	30	1
$M_{SB\_3\text{-}D\_QSAR\_}147_{MIN}$	0.91	0.37	2	Na	mmff94	1.1	1.2	6	47	35	4
$M_{SB\_3\text{-}D\_QSAR\_}148_{MIN}$	0.91	0.35	2	S.o	qtpie	0.3	3.5	8	10	4	2
$M_{SB\_3\text{-}D\_QSAR\_}149_{MIN}$	0.99	0.27	3	H.P	qeq	0.8	3.0	6	12	50	4
$M_{SB\_3\text{-}D\_QSAR\_}150_{MIN}$	0.99	0.36	3	OH	eem	2.1	1.0	5	22	41	1
$M_{SB\_3\text{-}D\_QSAR\_}151_{MIN}$	0.95	0.51	2	H	eem2015hm	1.7	1.2	5	66	11	4
$M_{SB\_3\text{-}D\_QSAR\_}152_{MIN}$	0.90	0.40	2	N.4	eem2015ha	0.8	2.0	7	49	46	1
$M_{SB\_3\text{-}D\_QSAR\_}153_{MIN}$	1.00	0.35	3	O.3	eem2015hn	0.3	1.5	10	61	18	3
$M_{SB\_3\text{-}D\_QSAR\_}154_{MIN}$	0.99	0.47	3	I	gasteiger	1.5	3.2	6	15	11	0
$M_{SB\_3\text{-}D\_QSAR\_}155_{MIN}$	0.91	0.40	2	C.3.H3	eem2015hm	0.7	3.0	6	49	3	1
$M_{SB\_3\text{-}D\_QSAR\_}156_{MIN}$	0.90	0.38	2	I	gasteiger	0.3	3.5	8	60	41	4
$M_{SB\_3\text{-}D\_QSAR\_}157_{MIN}$	0.99	0.40	3	O.3	qtpie	1.3	2.0	7	28	36	3
$M_{SB\_3\text{-}D\_QSAR\_}158_{MIN}$	1.00	0.65	3	O.3	eem2015hn	2.9	1.7	10	75	16	1
$M_{SB\_3\text{-}D\_QSAR\_}159_{MIN}$	0.95	0.49	2	Cl	qeq	2.6	2.7	8	61	28	0
$M_{SB\_3\text{-}D\_QSAR\_}160_{MIN}$	1.00	0.43	3	H.P	eem2015bm	1.9	1.6	10	33	27	1
$M_{SB\_3\text{-}D\_QSAR\_}161_{MIN}$	1.00	0.46	3	Cl	eem2015hm	2.4	1.2	4	39	17	1
$M_{SB\_3\text{-}D\_QSAR\_}162_{MIN}$	0.00	-0.36	1	C.3.H2	eem2015bm	1.8	3.0	8	63	1	2
$M_{SB\_3\text{-}D\_QSAR\_}163_{MIN}$	0.94	0.40	2	H.P	eem2015bm	2.7	1.5	6	24	27	0
$M_{SB\_3\text{-}D\_QSAR\_}164_{MIN}$	0.86	0.19	2	H.P	eem2015ba	0.3	3.5	10	30	33	4
$M_{SB\_3\text{-}D\_QSAR\_}165_{MIN}$	0.91	0.42	2	N.4	eem2015bm	1.5	2.6	10	73	24	1
$M_{SB\_3\text{-}D\_QSAR\_}166_{MIN}$	0.92	0.35	2	C.3.H3	eem2015hn	1.9	1.8	9	5	48	4
$M_{SB\_3\text{-}D\_QSAR\_}167_{MIN}$	0.88	0.33	2	C.3	gasteiger	1.6	3.3	4	63	33	3
$M_{SB\_3\text{-}D\_QSAR\_}168_{MIN}$	0.96	0.33	2	OH2	qeq	1.1	2.2	8	1	9	0
$M_{SB\_3\text{-}D\_QSAR\_}169_{MIN}$	0.89	0.39	2	S.3	openbabel	2.1	3.0	10	21	45	4
$M_{SB\_3\text{-}D\_QSAR\_}170_{MIN}$	0.91	0.41	2	OH	eem2015ba	0.2	1.7	5	9	43	3
$M_{SB\_3\text{-}D\_QSAR\_}171_{MIN}$	0.91	0.27	2	C.3.H1	eem2015ba	2.5	3.3	3	14	32	3
$M_{SB\_3\text{-}D\_QSAR\_}172_{MIN}$	0.95	0.35	2	Cl	qeq	1.5	2.6	6	76	17	4
$M_{SB\_3\text{-}D\_QSAR\_}173_{MIN}$	0.85	0.21	3	H	eem2015hm	1.9	2.6	10	38	2	0
$M_{SB\_3\text{-}D\_QSAR\_}174_{MIN}$	0.88	0.39	2	Cl	openbabel	2.8	3.3	7	65	31	3
$M_{SB\_3\text{-}D\_QSAR\_}175_{MIN}$	0.98	0.47	3	H.P	gasteiger	2.5	2.7	8	53	25	3
$M_{SB\_3\text{-}D\_QSAR\_}176_{MIN}$	0.94	0.40	2	H.P	mmff94	0.6	3.5	7	10	9	0

Table S33. SB 3-D QSAR models from ligands minimized into the Mmpl3 protein obtained from the 6AJJ pdb code.

$M_{SB\_3-D\_QSAR}\_177_{MIN}$	0.93	0.34	2	OH	eem2015bm	0.7	3.2	4	9	45	2
$M_{SB\_3-D\_QSAR}\_178_{MIN}$	0.89	0.38	2	Na	gasteiger	2.9	2.5	8	66	25	1
$M_{SB\_3-D\_QSAR}\_179_{MIN}$	1.00	0.55	2	F	qtpie	2.8	1.6	9	23	11	3
$M_{SB\_3-D\_QSAR}\_180_{MIN}$	0.94	0.41	2	C.3.H3	qtpie	2.9	1.7	4	4	30	4
$M_{SB\_3-D\_QSAR}\_181_{MIN}$	0.90	0.24	2	F	eem2015hn	2.1	2.9	9	72	24	1
$M_{SB\_3-D\_QSAR}\_182_{MIN}$	0.99	0.43	3	I	eem2015hm	0.4	3.0	5	62	25	4
$M_{SB\_3-D\_QSAR}\_183_{MIN}$	0.94	0.43	2	H.P	mmff94	1.9	1.3	3	26	31	4
$M_{SB\_3-D\_QSAR}\_184_{MIN}$	1.00	0.53	3	Na	mmff94	1.5	2.5	9	46	34	4
$M_{SB\_3-D\_QSAR}\_185_{MIN}$	0.90	0.41	2	F	eem2015ba	0.3	2.0	9	46	20	2
$M_{SB\_3-D\_QSAR}\_186_{MIN}$	0.94	0.41	2	F	qtpie	0.7	1.1	5	17	43	4
$M_{SB\_3-D\_QSAR}\_187_{MIN}$	0.99	0.42	3	C.3.H1	eem	1.2	1.5	3	35	11	3
$M_{SB\_3-D\_QSAR}\_188_{MIN}$	0.92	0.40	2	OH2	eem2015ha	0.8	1.5	4	62	43	2
$M_{SB\_3-D\_QSAR}\_189_{MIN}$	0.98	0.25	3	F	eem2015bm	1.1	2.4	6	34	31	1
$M_{SB\_3-D\_QSAR}\_190_{MIN}$	0.91	0.31	2	S.o	qtpie	0.2	3.4	9	73	32	1
$M_{SB\_3-D\_QSAR}\_191_{MIN}$	0.99	0.60	3	Cl	eem2015bn	2.3	2.8	5	41	7	0
$M_{SB\_3-D\_QSAR}\_192_{MIN}$	0.93	0.38	2	C.3	eem2015ha	2.2	1.4	4	6	12	4
$M_{SB\_3-D\_QSAR}\_193_{MIN}$	0.60	-0.05	1	C.3.H1	qtpie	1.7	3.5	3	27	25	3
$M_{SB\_3-D\_QSAR}\_194_{MIN}$	1.00	0.62	3	I	eem2015hm	1.4	1.1	8	77	3	1
$M_{SB\_3-D\_QSAR}\_195_{MIN}$	0.98	0.59	2	C.3.H3	qeq	1.8	2.1	5	54	12	0
$M_{SB\_3-D\_QSAR}\_196_{MIN}$	1.00	0.47	3	C.3	eem2015ha	1.3	2.3	10	67	10	1
$M_{SB\_3-D\_QSAR}\_197_{MIN}$	0.94	0.39	2	H.P	eem2015hm	1.9	3.0	8	35	42	3
$M_{SB\_3-D\_QSAR}\_198_{MIN}$	0.92	0.42	2	N.4	eem2015hn	0.7	2.7	9	75	17	0
$M_{SB\_3-D\_QSAR}\_199_{MIN}$	0.92	0.33	2	S.3	eem2015hm	2.7	1.8	4	30	43	3
$M_{SB\_3-D\_QSAR}\_200_{MIN}$	0.89	0.26	2	O.3	eem2015ba	2.6	2.7	7	3	30	1
$M_{SB\_3-D\_QSAR}\_201_{MIN}$	0.93	0.38	2	S.o	eem2015hn	1.9	1.7	8	41	43	1

A

B

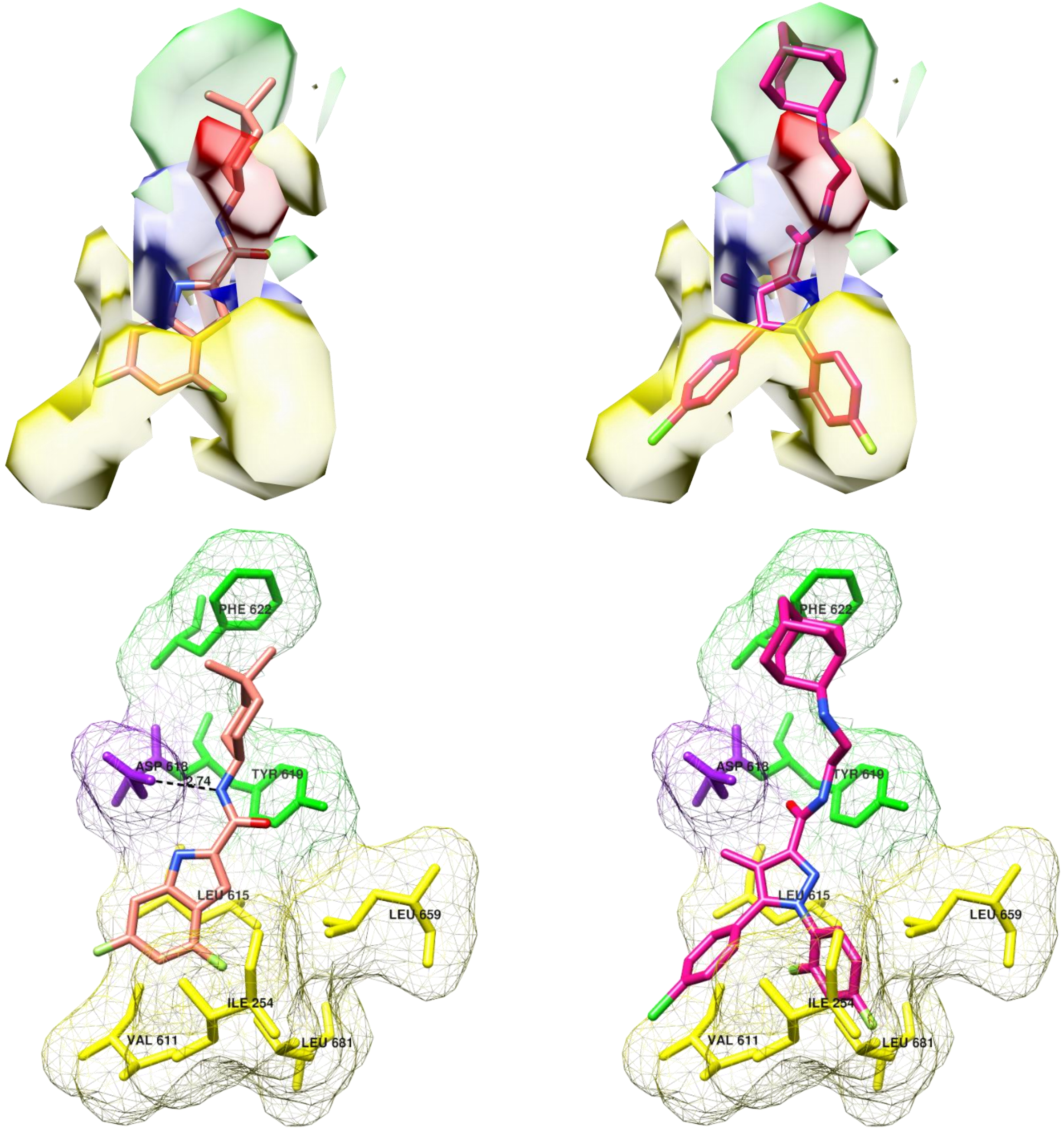

X **Y**

Figure S14. Graphical view of (row A) AAC values from CoMFA model $M_{SB\_3\text{-}D\_QSAR\_}97_{MIN}$ and (row B) COMBINE $M_{COMBINE\_}4$ model. Residues are colored as described in the main text. $AAC_{COMBINE}$ values for the hydrogen bonding (HB) the residues were colored in purple. The most potent compound (6C2M) is also reported in column X and one of the less potent (7WNX) in column Y. The hydrogen bonding distance of 2.74 Å between 6C2M amide nitrogen and Asp619 carboxylic side chain is also depicted in panel A/X.

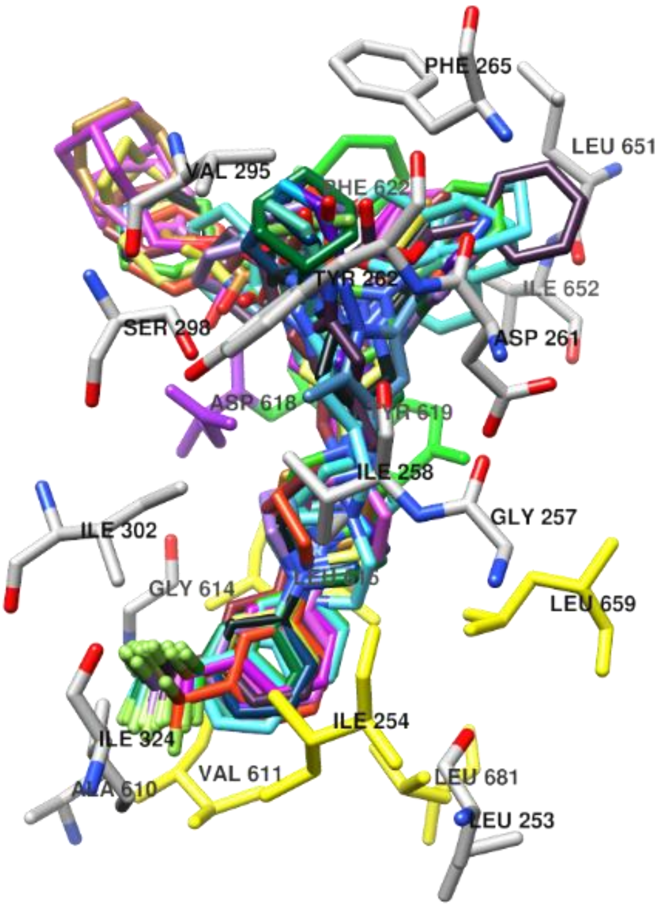

A

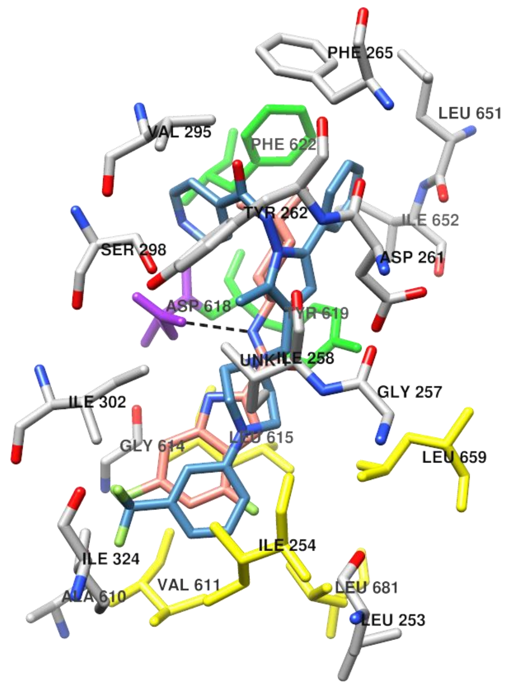

B

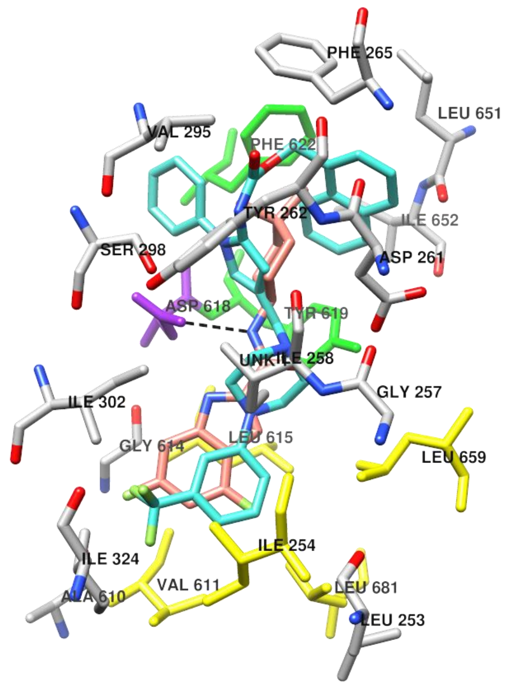

C

Figure S15. (A) Overall binding mode of all designed derivatives **2a-j** and **3a-i**. (B) Docked conformation of Sudoterb (Steel Blue) overlapped to 6C2M (pink). (C) Docked conformation of **3h** (Turquoise) overlapped to 6C2M (pink).

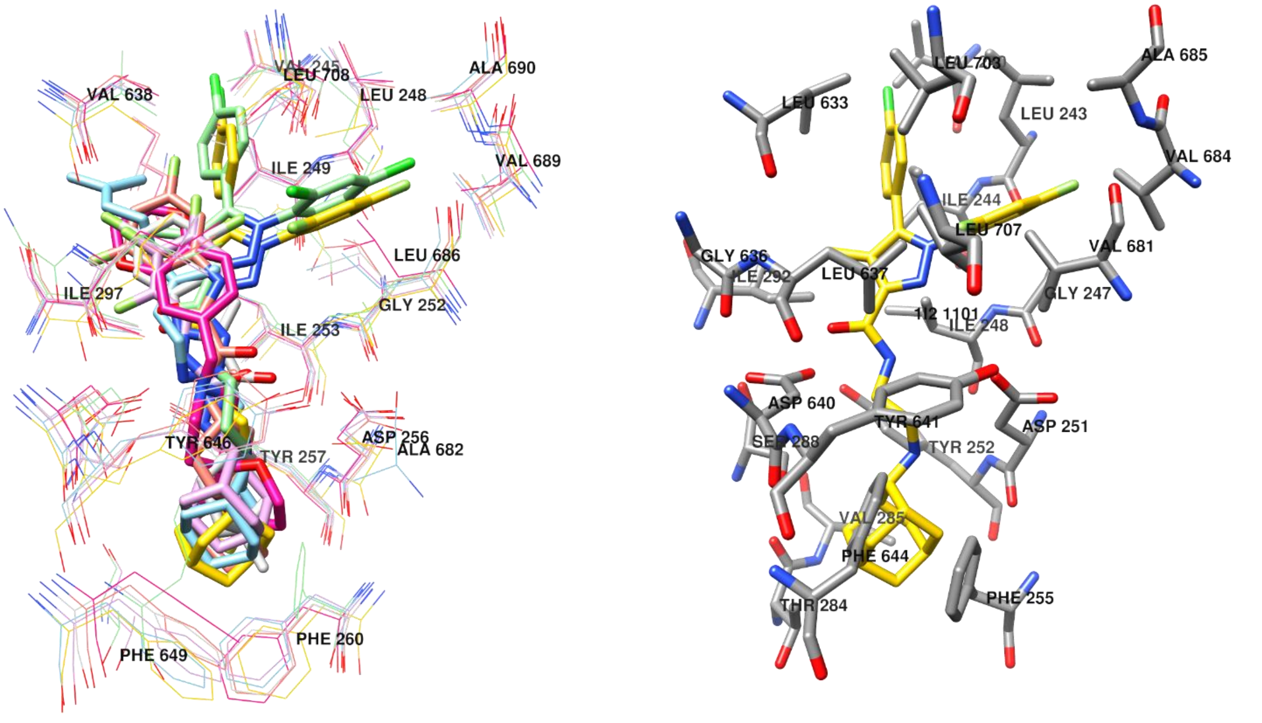

A **B**

Figure S16. A: Binding sites of the 6AJG, 6AJH, 6AJI, 6AJJ, 7C2M, 7C2N and 7WNX *M. smegmatis* MmpL3 complexes. B: binding site of the *M. tuberculosis* (7NVH, gray colored carbon atoms) with overlapped ligand extracted from the 7WNX complex (yellow colored carbon atoms).
Structures in A and B were superimposed with the matchmaker utility of UCSF Chimera. To note that the ligand display several steric clushes with the protein and there is no room to place it. Several residue sidechains are rotate towards the ligand cavity to prevent any possible molecular docking.

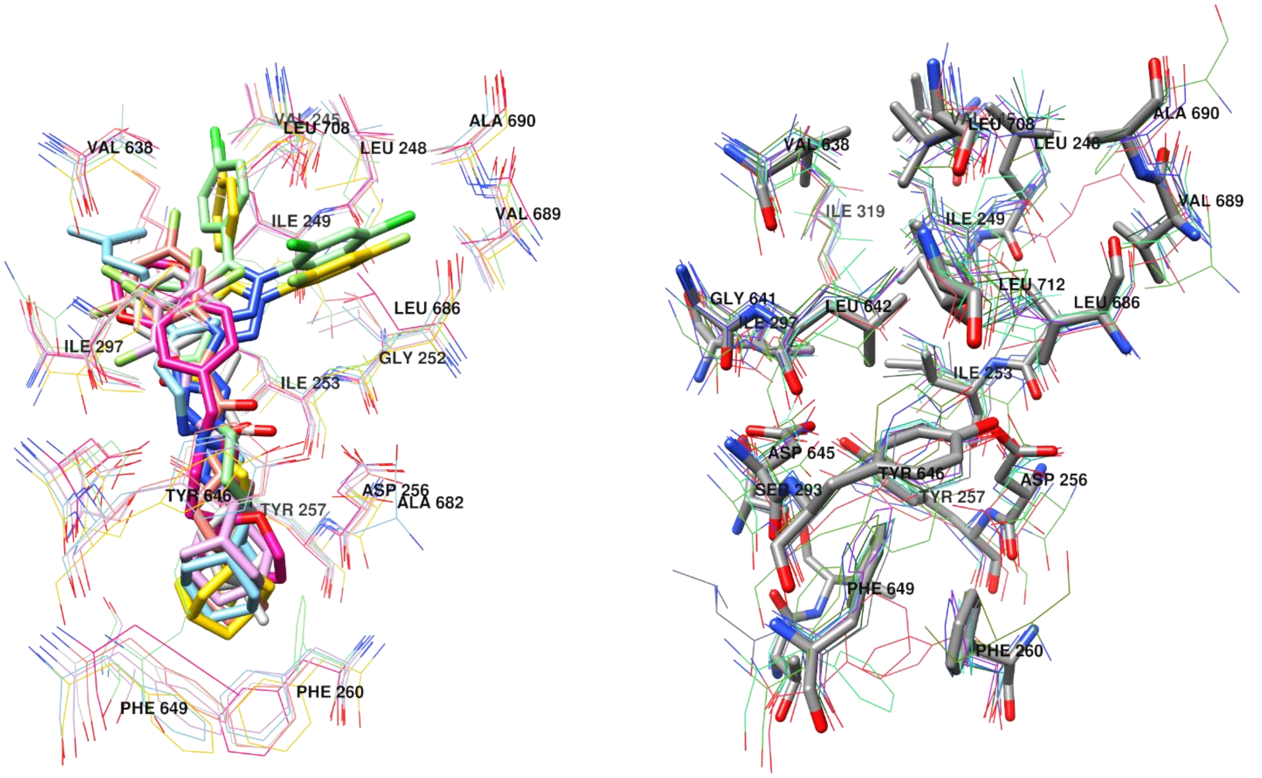

A **B**

Figure S17. Comparison of 6AJG, 6AJH, 6AJI, 6AJJ, 7C2M, 7C2N and 7WNX *M. smegmatis* MmpL3 complexes binding sites (A) to unbound *M. smegmatis* MmpL3 proteins, overlapped to that of *M. tuberculosis* (7NVH, gray colored carbon atoms) (B). Note that sidechains of Phe249 and Phe250 act like a gate being closed in the unbound proteins. In the complexes the two benzyl group are rotate away from the ligands.

Table S34. Chemical and physical properties of compounds **2a-j**

**Cpd**	**Lab. code**	**R**	**mp (°C)**	**Yield (%)**
**2a**	**MC 3494**	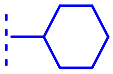	oil	70
**2b**	**MC 3504**	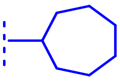	oil	85
**2c**	**MC 3525**	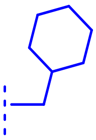	66-68[a]	60
**2d**	**MC 3528**	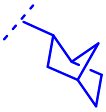	149-151[b]	50
**2e**	**MC 3529**	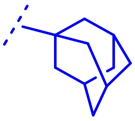	oil	78
**2f**	**MC 3491**	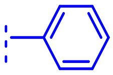	oil	90
**2g**	**MC 3203**	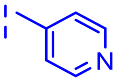	oil	50
**2h**	**MC 3492**	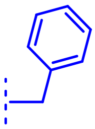	oil	75
**2i**	**MC 3515**	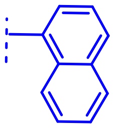	oil	75
**2j**	**MC 3511**	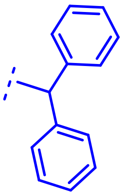	150-152[b]	80

Crystallisation solvent: a) cyclohexane; b) benzene

Table S35. Chemical and physical properties of compounds **3a-i**

Cpd	Lab. code	R	mp (°C)	Yield (%)
**3a**	**MC 3270**	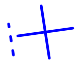	175-177[c]	74
**3b**	**MC 3526**	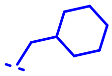	oil	75
**3c**	**MC 3505**	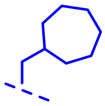	oil	82
**3d**	**MC 3533**	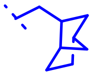	oil	89
**3e**	**MC 3535**	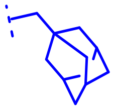	oil	83
**3f**	**MC 3233**	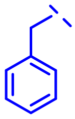	45-47[a]	57
**3g**	**MC 3227**	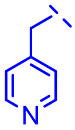	58-60[b]	62
**3h**	**MC 3536**	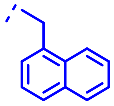	oil	69
**3i**	**MC 3512**	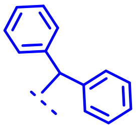	oil	77

Crystallisation solvent: a) hexane; b) cyclohexane; c) benzene

Table S36. Chemical and physical properties of intermediate compound **9**

Cpd	Molecular Structure	mp (°C)	Yield (%)
**6**	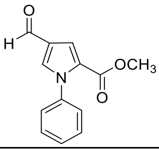	oil	70
**7**	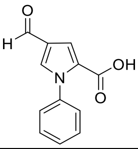	209-211[a]	88

Crystallization solvent: a) acetonitrile

Table S37. Chemical and physical properties of intermediate compounds **8a-j**

Cpd	Molecular Structure	mp (°C)	Yield (%)
**8a**	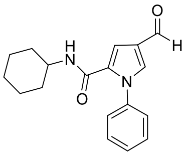	oil	82
**8b**	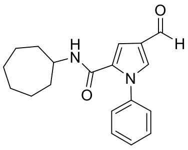	oil	75
**8c**	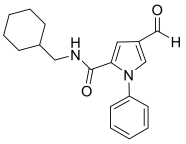	oil	87
**8d**	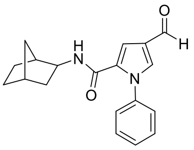	oil	71
**8e**	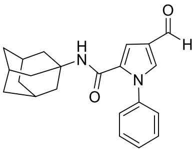	170-172[a]	65
**8f**	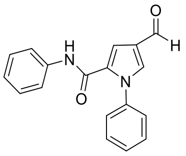	oil	78
**8g**	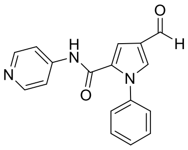	>259[b]	59
**8h**	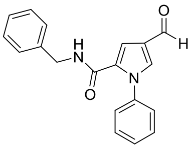	oil	73
**8i**	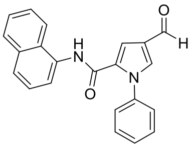	167-170[a]	60
**8j**	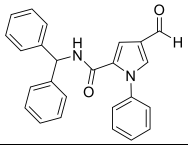	169-171[a]	68

Crystallisation solvent: a) benzene/acetonitrile; b) acetonitrile/methanol

Table S38. Chemical and physical properties of intermediate compounds **9a-i**

Cpd	Molecular Structure	mp (°C)	Yield (%)
**9a**	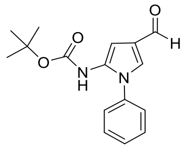	163-165[a]	67
**9b**	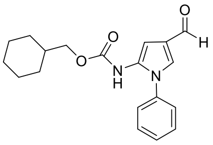	oil	69
**9c**	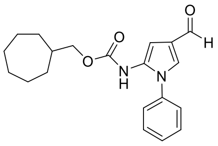	oil	71
**9d**	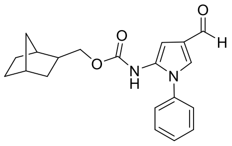	oil	75
**9e**	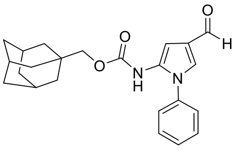	oil	77
**9f**	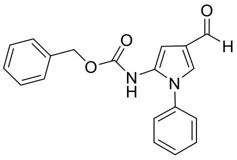	oil	80
**9g**	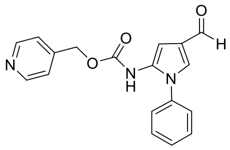	oil	51
**9h**	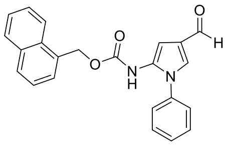	156-158[a]	77
**9i**	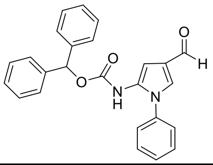	oil	54

Crystallization solvent: a) benzene

Table S39. Elementl analysis of compounds **2a-j** and **3a-i**

Cpd	Cpd	calculated				found			
		C	H	N	O	C	H	N	O
**2a**	**MC 3494**	68.22	6.51	10.97	3.13	68.17	6.52	11.01	3.14
**2b**	**MC 3504**	68.68	6.72	10.68	3.05	68.73	6.70	10.70	3.04
**2c**	**MC 3525**	68.68	6.72	10.68	3.05	68.59	6.73	10.64	3.06
**2d**	**MC 3528**	68.95	6.36	10.72	3.06	69.01	6.34	10.69	3.05
**2e**	**MC 3529**	70.44	6.63	9.96	2.84	70.36	6.65	9.98	2.83
**2f**	**MC 3491**	69.03	5.39	11.10	3.17	69.15	5.37	11.14	3.16
**2g**	**MC 3203**	66.52	5.18	13.85	3.16	66.58	5.20	13.89	3.17
**2h**	**MC 3492**	69.48	5.64	10.80	3.09	69.57	5.66	10.77	3.10
**2i**	**MC 3515**	71.47	5.27	10.10	2.88	71.54	5.28	10.13	2.89
**2j**	**MC 3511**	72.71	5.59	9.42	2.69	72.81	5.57	9.45	2.70
**3a**	**MC 3270**	64.79	6.24	11.19	6.39	64.89	6.22	11.16	6.40
**3b**	**MC 3526**	66.65	6.53	10.36	5.92	66.54	6.55	10.39	5.91
**3c**	**MC 3505**	67.13	6.72	10.10	5.77	67.02	6.70	10.14	5.76
**3d**	**MC 3533**	67.37	6.38	10.14	5.79	67.41	6.40	10.17	5.80
**3e**	**MC 3535**	68.90	6.63	9.45	5.40	68.97	6.61	9.41	5.39
**3f**	**MC 3233**	67.40	5.47	10.48	5.99	67.46	5.45	10.50	6.01
**3g**	**MC 3227**	65.04	5.27	13.08	5.97	64.93	5.26	13.05	5.98
**3h**	**MC 3536**	69.85	5.34	9.58	5.47	69.99	5.33	9.55	5.48
**3i**	**MC 3512**	70.81	5.45	9.17	5.24	70.75	5.46	9.13	5.23

^{1}H NMR spectral data of compounds 5-7, 8f, 3a-j, 4a-i.

(5) Methyl 1-phenyl-1*H*-pyrrole-2-carboxylate

Yield: 90% white solid. ^{1}H-NMR (CDCl$_3$) δ 3.75 (s, 3H, COOC*H$_3$*), 6.31-6.33 (t, 1H, pyrrole proton), 6.97-6.98 (m, 1H, pyrrole proton), 7.12-7.14 (m,1H, pyrrole proton), 7.25-7.35 (m, 5H, aromatic protons).

(6) Methyl 4-formyl-1-phenyl-1*H*-pyrrol-2-carboxylate

Yield: 80% white solid. 1H-NMR (CDCl$_3$) δ 3.75 (s, 3H, COOC*H$_3$*), 7.34-7.36 (m, 2H, pyrrole proton), 7.49-7.55 (m, 5H, aromatic protons), 9.88 (s, 1H, *CHO*).

(7) 4-Formyl-1-phenyl-1*H*-pyrrole-2-carboxylic acid

Yield 78 %: yellow solid. ^{1}H-NMR (CDCl$_3$) δ 7.31-7.32 (m, 1H, pyrrole proton), 7.41-7.54 (m, 5H, aromatic protons), 8.00 (m, 1H, pyrrole proton) 9.88 (s, 1H, *CHO*), 12.68 (s, 1H, COO*H*).

(8f) 4-Formyl-N,1-diphenyl-1*H*-pyrrol-2-carboxyamide

Yield:78%, oil. ^{1}H-NMR (CDCl$_3$) δ 7.10-7.15 (t, 1H, aromatic proton), 7.26-7.36 (m, 4H, aromatic protons), 7.39-7.44 (m, 1H, pyrrole proton) 7.45-7.50 (m, 5H, aromatic protons), 7.55-7.59 (m, 1H, pyrrole proton), 8.4 (s, 1H, CON*H*Ph) 9.9 (s, 1H, *CHO*).

(9f) Benzyl (4-formyl-1-phenyl-1*H*-pyrrol-2-yl)carbamate

Yield: 80% oil. ^{1}H-NMR (CDCl$_3$) δ 4.12-4.16 (s, 1H, OCON*H*), 5.13 (s, 2H, *CH$_2$*), 6.2 (s, 1H, pyrrole proton), 6.74 (s, 1H, pyrrole proton) 7.23-7.5 (m, 10H, aromatic protons), 9.8 (s, 1H, *CHO*).

(2a, MC3494) *N*-Cyclohexyl-1-phenyl-4-((4-(3-(trifluoromethyl)phenyl)piperazin-1-yl)methyl)-1*H*-pyrrole-2-carboxamide

Mp: oil; yield: 70%; ^{1}H-NMR (CDCl$_3$) δ 1.07-1.19 (m, 3H, cyclohexyl protons), 1.26-1.38 (m, 2H, cyclohexyl protons), 1.56-1.69 (m, 3H, cyclohexyl protons), 1.84-1.94 (m, 2H, cyclohexyl protons), 2.65-2.72 (m, 4H, piperazine protons), 3.24-3.33 (m, 4H, piperazine protons), 3.52 (s, 2H, piperazine-C*H$_2$*-pyrrole), 3.78-3.86 (m, 1H, cyclohexyl proton, 5.51 (d, 1H, CON*H*), 6.77 (s, 1H, pyrrole proton), 6.86 (s, 1H, pyrrole proton), 7.07-7.14 (m, 3H, aromatic protons), 7.34-7.54 (m, 6H, aromatic protons).

(2b, MC3504) *N*-Cycloheptyl-1-phenyl-4-((4-(3-(trifluoromethyl)phenyl)piperazin-1-yl)methyl)-1*H*-pyrrole-2-carboxamide

Mp: oil; yield: 85%; ^{1}H-NMR ($CDCl_3$) δ 1.26-1.62 (m, 10H, cycloheptyl protons), 1.87-1.91 (m, 2H, cycloheptyl protons), 2.64-2.71 (m, 4H, piperazine protons), 3.24-3.33 (m, 4H, piperazine protons), 3.50 (s, 2H, piperazine-C*H₂*-pyrrole), 3.96-4.04 (m, 1H, cycloheptyl proton, 5.56 (d, 1H, CON*H*), 6.76 (s, 1H, pyrrole proton), 6.85 (s, 1H, pyrrole proton), 7.07-7.13 (m, 3H, aromatic protons), 7.34-7.47 (m, 6H, aromatic protons).

(2c, MC3525) *N*-(Cyclohexylmethyl)-1-phenyl-4-((4-(3-(trifluoromethyl)phenyl)piperazin-1-yl)methyl)-1*H*-pyrrole-2-carboxamide

Mp: 66-68 °C; yield: 60%; recryst. solv: cyclohexane, ^{1}H-NMR (CDCl3) δ 0.83-1.01 (m, 4H, cyclohexyl protons), 1.09-1.37 (m, 4H, cyclohexyl protons), 1.41-1.82 (m, 3H, cyclohexyl protons), 2.63-2.72 (m, 4H, piperazine protons), 3.15 (t, 2H, -CH_2-NHCO), 3.23-3.33 (m, 4H, piperazine protons), 3.52 (s, 2H, piperazine-CH_2-pyrrole), 5.69 (t, 1H, CONH), 6.78 (s, 1H, pyrrole proton), 6.86 (s, 1H, pyrrole proton), 7.07-7.13 (m, 3H, aromatic protons), 7.29-7.47 (m, 6H, aromatic protons).

(2d, MC3528) *N*-(bicyclo[2.2.1]heptan-2-yl)-1-phenyl-4-((4-(3-(trifluoromethyl)phenyl)piperazin-1-yl)methyl)-1*H*-pyrrole-2-carboxamide

Mp: 149-151 °C; yield: 50%; recryst. solv.: benzene. ^{1}H-NMR ($CDCl_3$) δ 1.01-1.32 (m, 7H, norbornane protons), 1.75-1.80 (m, 1H, norbornane proton), 2.16-2.23 (m, 2H, norbornane proton), 2.68-2-71 (m, 4H, piperazine protons), 3.26-3.32 (m, 4H, piperazine protons), 3.50 (s, 2H, piperazine-CH_2-pyrrole), 3.72-3.79 (m, 1H, norbornane proton), 5.39-5.44 (m, 1H, CONH), 6.77 (s, 1H, pyrrole proton), 6.84 (s, 1H, pyrrole proton), 7.07-7.13 (m, 3H, aromatic protons,), 7.34-7.48 (m, 6H, aromatic protons).

(2e, MC3529) *N*-(Adamantan-1-yl)-1-phenyl-4-((4-(3-(trifluoromethyl)phenyl)piperazin-1-yl)methyl)-1*H*-pyrrole-2-carboxamide

Mp: oil; yield: 78%; ^{1}H-NMR ($CDCl_3$) δ 1.27-1.30 (m, 2H, adamantane protons), 1.90-2.00 (m, 6H, adamantane proton), 2.05-2.10 (m, 7H, adamantane proton), 2.67-2-71 (m, 4H, piperazine protons), 3.26-3.36 (m, 4H, piperazine protons), 3.51 (s, 2H, piperazine-CH_2-pyrrole), 5.35 (s, 1H, CONH), 6.72 (s, 1H, pyrrole proton), 6.83 (s, 1H, pyrrole proton), 7.07-7.14 (m, 3H, aromatic protons), 7.33-7.48 (m, 6H, aromatic protons).

(2f, MC3491) *N*,1-Diphenyl-4-((4-(3-(trifluoromethyl)phenyl)piperazin-1-yl)methyl)-1*H*-pyrrole-2-carboxamide

Mp: oil; yield: 90%; ^{1}H-NMR ($CDCl_3$) δ 2.69-2.72 (m, 4H, piperazine protons), 3.29-3.32 (m, 4H, piperazine protons), 3.54 (s, 2H, piperazine-CH_2-pyrrole), 6.94 (d, 2H, aromatic protons), 7.08-7.16 (m, 4H, CON*H*, pyrrole, and aromatic protons), 7.29-7.49 (m, 11H, aromatic protons).

(2g, MC3203) 1-Phenyl-*N*-(pyridin-4-yl)-4-((4-(3-(trifluoromethyl)phenyl)piperazin-1-yl)methyl)-1*H*-pyrrole-2-carboxamide

Mp: oil; yield: 50%; ^{1}H-NMR ($CDCl_3$) δ 2.68-2.74 (m, 4H, piperazine protons), 3.26-3.32 (m, 4H, piperazine protons), 3.55 (s, 2H, piperazine-CH_2-pyrrole), 6.99 (s, 2H, aromatic protons), 7.06-7.17 (m, 3H, CON*H*, pyrrole protons), 7.36-7.40 (m, 5H, aromatic and pyridine protons), 7.44-7.50 (m, 3H, aromatic protons), 7.63 (s, 1H, aromatic proton), 8.45 (s, 2H, pyridine protons).

(2h, MC3492) *N*-Benzyl-1-phenyl-4-((4-(3-(trifluoromethyl)phenyl)piperazin-1-yl)methyl)-1*H*-pyrrole-2-carboxamide

Mp: oil; yield: 75%; ^{1}H-NMR ($CDCl_3$) δ 2.65-2.69 (m, 4H, piperazine protons), 3.26-3.29 (m, 4H, piperazine protons), 3.50 (s, 2H, piperazine-CH_2-pyrrole), 4.49 (d, 2H, -CH_2-NHCO), 5.95 (t, 1H, CON*H*), 6.77 (s, 1H, pyrrole proton), 6.88 (s, 1H, pyrrole proton), 7.06-7.13 (m, 3H, aromatic protons), 7.24-7.45 (m, 11H, aromatic protons).

(2i, MC3515) *N*-(Naphthalen-1-yl)-1-phenyl-4-((4-(3-(trifluoromethyl)phenyl)piperazin-1-yl)methyl)-1*H*-pyrrole-2-carboxamide

Mp: oil; yield: 75%; ^{1}H-NMR ($CDCl_3$) δ 2.69-2.73 (m, 4H, piperazine protons), 3.28-3.33 (m, 4H, piperazine protons), 3.60 (s, 2H, piperazine-CH_2-pyrrole), 7.00 (s, 1H, pyrrole proton), 7.08-7.16 (m, 4H, pyrrole and aromatic protons, CON*H*), 7.35-7.47 (m, 10H, aromatic protons), 7.65-7.68 (m, 1H, aromatic proton), 7.85-7.88 (m, 2H, aromatic protons), 8.05-8.08 (m, 1H, aromatic proton).

(2j, MC3511) *N*-Benzhydryl-1-phenyl-4-((4-(3-(trifluoromethyl)phenyl)piperazin-1-yl)methyl)-1*H*-pyrrole-2-carboxamide

Mp: 150-152 °C; yield: 80%; recryst. solv.: benzene. ^{1}H-NMR (CDCl3) δ 2.63-2.75 (m, 4H, piperazine protons), 3.22-3.29 (m, 4H, piperazine protons), 3.53 (s, 2H, piperazine-CH_2-pyrrole), 6.19-6.25 (d, 1H, Ph-CH-Ph), 6.28-6.33 (m, 1H, CONH), 6.86—6.91 (m, 2H, pyrrole and aromatic protons), 7.08-7.45 (m, 19H, pyrrole, and aromatic protons).

(3a, MC3270) *tert*-Butyl (1-phenyl-4-((4-(3-(trifluoromethyl)phenyl)piperazin-1-yl)methyl)-1*H*-pyrrol-2-yl)carbamate

Mp: 175-177 °C; yield: 74%; recryst. solv.: benzene. ^{1}H-NMR (CDCl$_3$) δ 1.41 (s, 9H, OC$(CH_3)_3$), 2.69-2.72 (m, 4H, piperazine protons), 3.27-3.30 (m, 4H, piperazine protons), 3.51 (s, 2H, piperazine-CH_2-pyrrole), 5.98 (s, 1H, pyrrole proton), 6.21 (s, 1H, CON*H*), 6.67 (s, 1H, pyrrole proton), 7.07-7.09 (m, 2H, aromatic protons), 7.12 (s, 1H, aromatic proton), 7.33-7.39 (m, 4H, aromatic protons), 7.44-7.49 (m, 2H, aromatic protons).

(3b, MC3526) Cyclohexylmethyl (1-phenyl-4-((4-(3-(trifluoromethyl)phenyl)piperazin-1-yl)methyl)-1*H*-pyrrol-2-yl)carbamate

Mp: oil; yield: 75%; ^{1}H-NMR (CDCl$_3$) δ 0.88-0.99 (m, 4H, cyclohexyl protons), 1.15-1.32 (m, 4H, cyclohexyl protons), 1.51-1.80 (m, 3H, cyclohexyl protons), 2.65-2.75 (m, 4H, piperazine protons), 3.22-3.32 (m, 4H, piperazine protons), 3.52 (s, 2H, piperazine-CH_2-pyrrole), 3.88-3.95 (d, 2H, O-CH_2-CH), 6.18-6.25 (m, 2H, CON*H*, pyrrole proton), 6.68 (s, 1H, pyrrole proton), 7.05-7.13 (m, 3H, aromatic protons), 7.33-7.47 (m,6H, aromatic protons).

(3c, MC3505) Cycloheptylmethyl (1-phenyl-4-((4-(3-(trifluoromethyl)phenyl)piperazin-1-yl)methyl)-1*H*-pyrrol-2-yl)carbamate

Mp: oil; yield: 82%; ^{1}H-NMR (CDCl$_3$) δ 1.26 (m, 1H, cycloheptyl proton), 1.40-1.76 (m, 12H, cycloheptyl protons), 2.64-2.75 (m, 4H, piperazine protons), 3.24-3.33 (m, 4H, piperazine protons), 3.51 (s, 2H, piperazine-CH_2-pyrrole), 3.84-3.92 (m, 2H, -O-CH_2-CH$(CH_2)_6$, 6.17-6.28 (m, 2H, CON*H*, pyrrole proton), 6.68 (s, 1H, pyrrole proton), 7.06-7.15 (m, 3H, aromatic protons), 7.33-7.51 (m, 6H, aromatic protons).

(3d, MC3533) (Bicyclo[2.2.1]heptan-2-yl)methyl (1-phenyl-4-((4-(3-(trifluoromethyl) phenyl)piperazin-1-yl)methyl)-1*H*-pyrrol-2-yl)carbamate

Mp: oil; yield: 89%; ^{1}H-NMR (CDCl$_3$) δ 1.14-1.56 (m, 6H, norbornane protons), 1.65-1.72 (m, 1H, norbornane proton), 2.16-2.23 (m, 4H, norbornane protons, -O-CH_2-norborane), 2.68-2-76 (m, 4H, piperazine protons), 3.23-3.32 (m, 4H, piperazine protons), 3.52 (s, 2H, piperazine-CH_2-pyrrole), 3.91-3.99 (m, 1H, norbornane proton), 4.09-4.18 (m, 1H, norbornane proton), 6.14-6.28 (m, 2H, CON*H*, pyrrole proton), 6.69 (s, 1H, pyrrole proton), 7.07-7.14 (m, 3H, aromatic protons), 7.34-7.48 (m, 6H, aromatic protons).

(3e, MC3535) (Adamantan-1-yl)methyl (1-phenyl-4-((4-(3-(trifluoromethyl)phenyl) piperazin-1-yl)methyl)-1*H*-pyrrol-2-yl)carbamate

Mp: oil; yield: 83%; ^{1}H-NMR (CDCl$_3$) δ 1.20-1.40 (m, 2H, adamantane protons), 1.56-1.84 (m, 10H, adamantane proton), 1.90-2.05 (m, 3H, adamantane protons,), 2.65-2-76 (m, 4H, piperazine protons), 3.25-3.32 (m, 4H, piperazine protons), 3.53 (s, 2H, piperazine-CH_2-pyrrole), 3.71 (s, 2H,-O-CH_2-adamantane), 6.09-6.28 (m, 2H, CON*H*, pyrrole proton), 6.70 (s, 1H, pyrrole proton), 7.03-7.15 (m, 3H, aromatic protons), 7.30-7.38 (m, 4H, aromatic protons), 7.42-7.48 (m, 2H, aromatic protons).

(3f, MC3233) Benzyl (1-phenyl-4-((4-(3-(trifluoromethyl)phenyl)piperazin-1-yl)methyl)-1*H*-pyrrol-2-yl)carbamate

Mp: 45-47 °C; yield: 57%; recryst. solv.: *n*-hexane. ^{1}H-NMR (CDCl$_3$) δ 2.64-2.73 (m, 4H, piperazine protons), 3.25-3.34 (m, 4H, piperazine protons), 3.52 (s, 2H, piperazine-CH_2-pyrrole), 5.31 (s, 2H, -O-CH_2-Ar), 6.21-6.30 (m, 2H, CON*H*, pyrrole proton), 6.68 (s, 1H, pyrrole proton), 7.05-7.13 (m, 3H, aromatic protons), 7.33-7.47 (m,11H, aromatic protons).

(3g, MC3227) Pyridin-4-ylmethyl (1-phenyl-4-((4-(3-(trifluoromethyl)phenyl)piperazin-1-yl)methyl)-1*H*-pyrrol-2-yl)carbamate

Mp: 58-60 °C; yield: 62%; recryst. solv.: cyclohexane. ^{1}H-NMR (CDCl$_3$) δ 2.64-2.70 (m, 4H, piperazine protons), 3.25-3.30 (m, 4H, piperazine protons), 3.53 (s, 2H, piperazine-CH_2-pyrrole), 5.14 (s, 2H, -CH_2-OCONH-), 6.25-6.30 (m, 2H, CON*H*, pyrrole proton), 6.71 (s, 1H, pyrrole proton), 7.06-7.17 (m, 4H, aromatic protons), 7.28-7.45 (m, 7H, aromatic and pyridine protons), 8.54 (s, 2H, pyridine protons).

(3h, MC3536) Naphthalen-1-ylmethyl (1-phenyl-4-((4-(3-(trifluoromethyl)phenyl) piperazin-1-yl)methyl)-1*H*-pyrrol-2-yl)carbamate

Mp: oil; yield: 69%; ^{1}H-NMR ($CDCl_3$) δ 2.53-2.70 (m, 4H, piperazine protons), 3.15-3.33 (m, 4H, piperazine protons), 3.48 (s, 2H, piperazine-CH_2-pyrrole), 5.62 (s, 2H, Ar-O-CH_2-), 6.27 (s, 1H, pyrrole proton),6.58-6.68 (m, 2H, CON*H*, pyrrole proton), 7.07-7.70 (m,13H, aromatic protons), 7.84-8.10 (m, 3H, aromatic protons).

(3i, MC3512) Benzhydryl (1-phenyl-4-((4-(3-(trifluoromethyl)phenyl)piperazin-1-yl)methyl)-1*H*-pyrrol-2-yl)carbamate

Mp: oil; yield: 77%; ^{1}H-NMR ($CDCl_3$) δ 2.66-2.72 (m, 4H, piperazine protons), 3.27-3.28 (m, 4H, piperazine protons), 3.55 (s, 2H, piperazine-CH_2-pyrrole, 6.26 (s, 1H, Ph-C*H*-Ph), 6.69 (s, 1H, pyrrole proton), 6.83 (s, 1H, pyrrole proton), 7.06-7.16 (m, 3H, aromatic protons), 7.19-7.41 (m, 17H, aromatic protons, CON*H*).

References

1. Kumar, A.; Karkara, B. B.; Panda, G., Novel candidates in the clinical development pipeline for TB drug development and their synthetic approaches. *Chem Biol Drug Des* **2021,** *98* (5), 787-827.
2. Bhakta, S.; Scalacci, N.; Maitra, A.; Brown, A. K.; Dasugari, S.; Evangelopoulos, D.; McHugh, T. D.; Mortazavi, P. N.; Twist, A.; Petricci, E.; Manetti, F.; Castagnolo, D., Design and Synthesis of 1-((1,5-Bis(4-chlorophenyl)-2-methyl-1H-pyrrol-3-yl)methyl)-4-methylpiperazine (BM212) and N-Adamantan-2-yl-N′-((E)-3,7-dimethylocta-2,6-dienyl)ethane-1,2-diamine (SQ109) Pyrrole Hybrid Derivatives: Discovery of Potent Antitubercular Agents Effective against Multidrug-Resistant Mycobacteria. *Journal of Medicinal Chemistry* **2016,** *59* (6), 2780-2793.
3. Biava, M.; Porretta, G. C.; Poce, G.; Battilocchio, C.; Alfonso, S.; De Logu, A.; Serra, N.; Manetti, F.; Botta, M., Identification of a novel pyrrole derivative endowed with antimycobacterial activity and protection index comparable to that of the current antitubercular drugs streptomycin and rifampin. *Bioorg Med Chem* **2010,** *18* (22), 8076-84.
4. Castagnolo, D.; Manetti, F.; Radi, M.; Bechi, B.; Pagano, M.; De Logu, A.; Meleddu, R.; Saddi, M.; Botta, M., Synthesis, biological evaluation, and SAR study of novel pyrazole analogues as inhibitors of Mycobacterium tuberculosis: Part 2. Synthesis of rigid pyrazolones. *Bioorganic & Medicinal Chemistry* **2009,** *17* (15), 5716-5721.
5. Castagnolo, D.; De Logu, A.; Radi, M.; Bechi, B.; Manetti, F.; Magnani, M.; Supino, S.; Meleddu, R.; Chisu, L.; Botta, M., Synthesis, biological evaluation and SAR study of novel pyrazole analogues as inhibitors of Mycobacterium tuberculosis. *Bioorganic & Medicinal Chemistry* **2008,** *16* (18), 8587-8591.
6. Manetti, F.; Magnani, M.; Castagnolo, D.; Passalacqua, L.; Botta, M.; Corelli, F.; Saddi, M.; Deidda, D.; De Logu, A., Ligand-based virtual screening, parallel solution-phase and microwave-assisted synthesis as tools to identify and synthesize new inhibitors of mycobacterium tuberculosis. *ChemMedChem* **2006,** *1* (9), 973-89.
7. Taminau, J.; Thijs, G.; De Winter, H., Pharao: pharmacophore alignment and optimization. *J Mol Graph Model* **2008,** *27* (2), 161-9.
8. Kawabata, T.; Nakamura, H., 3D flexible alignment using 2D maximum common substructure: dependence of prediction accuracy on target-reference chemical similarity. *J Chem Inf Model* **2014,** *54* (7), 1850-63.
9. Mirabello, C.; Wallner, B., InterLig: improved ligand-based virtual screening using topologically independent structural alignments. *Bioinformatics* **2020,** *36* (10), 3266-3267.
10. Landrum, G., RDKit: Open-Source Cheminformatics Software. **2016**.
11. Vainio, M. J.; Puranen, J. S.; Johnson, M. S., ShaEP: molecular overlay based on shape and electrostatic potential. *J Chem Inf Model* **2009,** *49* (2), 492-502.
12. Grant, J. A.; Pickup, B. T., A Gaussian Description of Molecular Shape. *The Journal of Physical Chemistry* **1995,** *99* (11), 3503-3510.
